# Differential expression of small RNAs from *Burkholderia thailandensis* in response to varying environmental and stress conditions

**DOI:** 10.1186/1471-2164-15-385

**Published:** 2014-05-19

**Authors:** Chris J Stubben, Sofiya N Micheva-Viteva, Yulin Shou, Sarah K Buddenborg, John M Dunbar, Elizabeth Hong-Geller

**Affiliations:** Bioscience Division, Los Alamos National Laboratory, Los Alamos, NM 87544 USA

**Keywords:** Small RNAs, *Burkholderia*, Microarray, Stress conditions, Bacterial adaptation, Gene expression

## Abstract

**Background:**

Bacterial small RNAs (sRNAs) regulate gene expression by base-pairing with downstream target mRNAs to attenuate translation of mRNA into protein at the post-transcriptional level. In response to specific environmental changes, sRNAs can modulate the expression levels of target genes, thus enabling adaptation of cellular physiology.

**Results:**

We profiled sRNA expression in the Gram-negative bacteria *Burkholderia thailandensis* cultured under 54 distinct growth conditions using a *Burkholderia*-specific microarray that contains probe sets to all intergenic regions greater than 90 bases. We identified 38 novel sRNAs and performed experimental validation on five sRNAs that play a role in adaptation of *Burkholderia* to cell stressors. In particular, the *trans*-encoded BTH_s1 and s39 exhibited differential expression profiles dependent on growth phase and cell stimuli, such as antibiotics and serum. Furthermore, knockdown of the highly-expressed BTH_s39 by antisense transcripts reduced *B. thailandensis* cell growth and attenuated host immune response upon infection, indicating that BTH_s39 functions in bacterial metabolism and adaptation to the host. In addition, expression of *cis*-encoded BTH_s13 and s19 found in the 5′ untranslated regions of their cognate genes correlated with tight regulation of gene transcript levels. This sRNA-mediated downregulation of gene expression may be a conserved mechanism of post-transcriptional gene dosage control.

**Conclusions:**

These studies provide a broad analysis of differential *Burkholderia* sRNA expression profiles and illustrate the complexity of bacterial gene regulation in response to different environmental stress conditions.

**Electronic supplementary material:**

The online version of this article (doi:10.1186/1471-2164-15-385) contains supplementary material, which is available to authorized users.

## Background

The discovery of small RNAs (sRNAs) as ubiquitous regulators of gene expression in the last decade represents fertile ground for scientific advances in both fundamental biology and potential applications in medicine. Bacterial sRNAs vary in length from ~50-450 nucleotides and are generally encoded in the intergenic regions (IGRs) of DNA [1, 2]. The majority of sRNAs function as negative regulators by base-pairing with the 5′ untranslated region (UTR) of target mRNAs to prevent translation by sterically blocking the ribosome binding site and reducing the stability of the mRNA. Bacterial sRNAs hybridize in short discontinuous stretches of sequence with limited complementarity, and are thus able to base pair with multiple mRNAs to regulate a complex network of genes that underpin diverse cellular behaviors. The post-transcriptional regulator and RNA chaperone Hfq has been shown to facilitate base pairing between bacterial sRNAs and mRNA targets [3].

sRNAs modulate the expression levels of target genes in response to specific environmental changes. For example, the *Escherichia coli* sRNA, RyhB, inhibits expression of ~18 proteins that play a role in iron acquisition under iron-limiting conditions to ensure that scarce iron is available for more essential cell processes [4]. Another sRNA, SgrS, is expressed under metabolic stress conditions when *E. coli* is unable to metabolize intracellular phosphorylated sugars [5]. In addition, bacterial sRNAs have been found to regulate expression of virulence genes in a variety of pathogens during host infection, including *Staphylococcus aureus*, *Pseudomonas aeruginosa*, and *Salmonella typhimurium*[6, 7].

The first known sRNAs, primarily from *E. coli,* were identified fortuitously by the direct detection of highly abundant RNAs (e.g. 4.5S RNA, RNaseP RNA) or in the context of protein-focused studies (e.g. CsrB and OxyS RNAs) [8]. In the last decade, global analytical approaches, such as gene expression microarrays and deep sequencing, have begun to systematically reveal sRNA populations in a wide variety of bacteria, including *Listeria monocytogenes*[9], *Listeria pneumophila*[10], *Yersinia pseudotuberculosis*[11], and *S. typhimurium*[12]. These analyses have generated hundreds of sRNA candidates that are actively being investigated to determine their functional activities. However, these studies have generally limited sRNA expression profiling to one or a few different bacterial growth conditions. Given that sRNA expression is highly dependent on a specific environment, we sought to profile differential sRNA expression levels in response to a wider variety of growth conditions and cell stimuli.

We examined sRNA expression profiles in the Gram-negative bacterial genus, *Burkholderia,* which encompasses ~60 species that exhibit a wide range of biological functions, including pathogenicity, bioremediation, and nitrogen fixation. The two best-characterized species, *B. pseudomallei* and *B. mallei*, the causative agents of human melioidosis and equine glanders, respectively, are categorized as Category B biothreat agents by the CDC. We designed and constructed a microarray that contains probe sets to all IGRs longer than 90 bases using genome annotations from *B. thailandensis*, a closely-related attenuated species to *B. pseudomallei. B. thailandensis* was cultured under 54 distinct growth conditions that varied media, temperature, salt, pH, nutrient limitations, and several poisons, such as antibiotics and ethanol. From this analysis, we identified 38 novel sRNAs, 20 of which are also present in *B. pseudomallei* and *B. mallei*. Experimental validation suggested that some sRNAs play a role in adaptation of *Burkholderia* to antibiotic exposure and survival in a host-specific environment.

## Results

### Differential *B. thailandensis* sRNA expression profiles in response to stress

We obtained 162 gene expression profiles from *B. thailandensis* cultured in 54 distinct growth conditions to identify novel sRNAs that regulate stress adaptation. The expression profiles were obtained using a custom Affymetrix microarray containing probes to most *B. thailandensis* genes and all IGRs greater than 90 bases. The majority of profiles (101/162) were from four time course studies measuring changes in temperature, pH, salt and phosphate concentrations (Table 1). The remaining 61 experiments included a wide range of conditions assessing nutrient limitations, deprivation of oxygen, nitrogen, sulfur, or magnesium, and several poisons such as antibiotics, ethanol, peroxide, and salicylate. We analyzed the 2908 probe sets targeting the IGRs in the four time course studies and used the following criteria to identify candidate sRNAs: (1) probes that did not have similar fold changes and expression levels as the immediate flanking genes, (2) two or more probes forming a single peak spanning a minimum of 30 bases, and (3) overlapping sRNA and transcription terminator predictions.

**Table 1:** ***Burkholderia*** **growth conditions for microarray analysis**

Time course experiments						
ID	Medium	Conditions	OD600	Time pt	Time from t0	Label
1	Nutrient broth	25 C	1	0		temp t0
2	Nutrient broth	37 C	0.65	1	20m	temp t1
3	Nutrient broth	37 C	1	2	63m	temp t2
4	Nutrient broth	37 C	1.25	3	78m	temp t3
5	Nutrient broth	37 C	1.5	4	96m	temp t4
6	Nutrient broth	pH 9	0.6	0		pH t0
7	Nutrient broth	pH 4	0.7	1	15m	pH t1
8	Nutrient broth	pH 4.5	1	2	15h	pH t2
9	Nutrient broth	pH 4.5	1.2	3	15.6h	pH t3
10	Nutrient broth	pH 5+	1.6	4	15.3h	pH t4
11	Nutrient broth	no salt	0.6	0		salt t0
12	Nutrient broth	350mM NaCl	0.7	1	15m	salt t1
13	Nutrient broth	350mM NaCl	1	2	107m	salt t2
14	Nutrient broth	350mM NaCl	1.2	3	147m	salt t3
15	Nutrient broth	350mM NaCl	1.5	4	201m	salt t4
16	M9 succinate	80mM PO4	1	0		PO4 t0
17	M9 succinate	40mM PO4	0.6	1	15m	PO4 t1
18	M9 succinate	40mM PO4	0.9	2	97m	PO4 t2
19	M9 succinate	40mM PO4	1.2	3	160m	PO4 t3
20	M9 succinate	20mM PO4	0.6	4	188m	PO4 t4
21	M9 succinate	20mM PO4	0.6	5	363m	PO4 t5
Five replicates for all conditions, except samples at pH 4.5 with 3 replicates.						
**Single time point experiments**						
**ID**	**Medium**	**Temp**	**Replicates**	**Description**	**Label**	
22	M9 succinate	16	4	temp 16 C	16°C	
23	M9 succinate	37	4	temp 37 C	37°C	
24	M9 succinate	37	1	70 mM phosphate	PO4	
25	M9 succinate	37	1	40 mM phosphate	low PO4	
26	M9 succinate	37	1	anaerobic	N	
27	M9 succinate	37	1	anaerobic + CO2	N + CO2	
28	M9 succinate	37	1	nitrogen-limited	low N	
29	M9 succinate	37	1	sulfur-limited	low sulfur	
30	M9 succinate	37	1	solid media	solid	
31	M9 succinate	37	1	50 mM Mg++	high Mg	
32	M9 succinate	37	1	5 uM Mg++	low Mg	
33	M9 succinate	37	1	0.07g/L EDTA	EDTA	
34	M9 succinate	37	1	0.07g/L EDTA with nutrient-limit	EDTA/limit	
35	M9 succinate	37	1	+0.1% phenol	phenol	
36	M9 succinate	37	1	+200 ppm Bleach	bleach	
37	M9 succinate	37	1	+5% EtOH	ethanol	
38	M9 glucose	25	1	M9-glucose	glucose	
39	M9 galactose	37	1	M9-galactose	galactose	
40	M9 proline	37	1	M9-proline	proline	
41	Luria broth	25	4	temp 25 C	25°C	
42	Luria broth	37	4	temp 37C	37°C	
43	Luria broth	41	4	temp 41 C	42°C	
44	Luria broth	37	4	pH 9	pH 9	
45	Luria broth	37	4	pH 5	pH 5	
46	Luria broth	37	4	80mM Peroxide	H_2_0_2_	
47	Luria broth	37	2	0.5M NaCl	NaCl	
48	Calf serum	37	4	CS infusion, temp 37 C	37°C	
49	Calf serum	37	1	antibiotics	antibiotics	
50	Calf serum	37	1	antibiotics + 0.07g/L EDTA	antibiotics + EDTA	
51	Calf serum	37	1	aspirin	salicylate	
52	Brain heart	37	1	solid media	solid	
53	Tryptic soy	37	1	kan mutant	kan mutant	
54	Tryptic soy	37	1	wt	wt	

The antibiotics treatment consisted of carbenicillin (100 μg/ml), chloramphenicol (30 μg/ml), erythromycin (200 μg/ml, and kanamycin (50 μg/ml). The EDTA concentration of 0.07g/L is standard usage for chelation therapy. The kan mutant (ID53) is a spontaneous *B. thailandensis* mutant isolated in response to a sublethal exposure to kanamycin.

**Figure 1:**
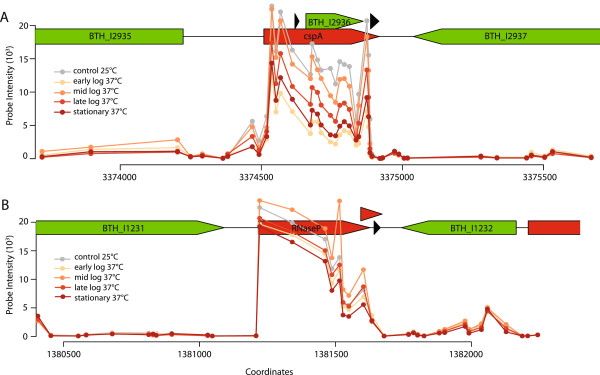
**Expression plots of** ***cis-*** **(cspA) and** ***trans*** **-encoded (RNaseP) sRNAs from** ***B. thailandensis*** **based on microarray analysis. (A)** CspA is a sRNA prediction from Rfam (red) and resides upstream of predicted gene BTH_I2936. CspA expression was downregulated at 37°C (yellow to red-labeled expression profiles) compared to 25°C (grey expression profile). Predicted genes and terminators are shown in green and black triangles, respectively. **(B)** RNaseP is a predicted sRNA from Rfam (red). RNaseP was expressed independently from flanking genes at high levels in response to practically all conditions tested.

We observed the presense of both *cis*-encoded and *trans*-encoded sRNAs (Figure 1). Many of the top-ranked IGRs exhibited similar expression levels to a flanking gene, suggesting potential *cis*-encoded regulation, as seen for the thermoregulator *cspA*, which resides upstream of BTH_I2936 (Figure 1A, Rfam database and plots # 2, 3, 5, 6, 9 in Additional file 1). However, *Burkholderia* genomes have a high GC content and inconsistent gene start predictions [13]. Over half the genes in the *B. thailandensis* genome have an alternate start site in other gene models. Therefore, many of the *cis*-encoded candidate sRNAs may be false positives, representing mRNA from genes for which the translation start site should be extended upstream. Given this difficulty in predicting gene start sites and 5′ UTRs, we excluded the majority of *cis*-encoded sRNAs from further study, except for candidate sRNAs (e.g. BTH_s13 and s19) that were characterized by a single peak upstream from a gene with little or no expression.

**Figure 2:**
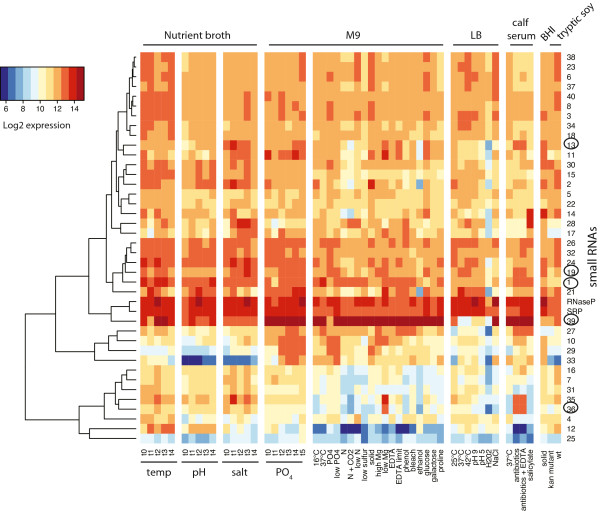
**Differential expression of 40 sRNAs identified from microarray analysis.** This heatmap depicts differential expression of 38 novel and 2 known sRNAs across all 54 different growth conditions listed at the bottom (described in Table 1). The first 21 columns were time course experiments used for the initial sRNA identification. Different types of media for *B. thailandensis* growth are denoted at the top of the heatmap. sRNAs that were further validated are circled on the right. The sRNAs were clustered using Canberra distance and complete linkage clustering and expression levels are depicted using a log2 scale.

We identified 38 novel sRNAs and two known sRNAs, the trans-encoded ribonuclease P (RNaseP, Figure 1B) and signal recognition particle (SRP), that matched our search criteria (Table 2). The expression profiles of all 40 sRNAs are displayed in a cluster heatmap (Figure 2) and in individual genome plots using probe data from the four time-course experiments (Additional file 2). The wide range of responses to the 54 experimental conditions illustrates the complexity of bacterial gene regulation in *B. thailandensis*. Many sRNAs were differentially expressed in response to a mixture of antibiotics to which *Burkholderia* is naturally resistant (sub-lethal concentrations of carbenicillin, chloramphenicol, erythromycin and kanamycin) in calf serum (Table 2 and Figure 2). In particular, nine sRNAs (BTH_s10, s13, s16, s19, s21, s27, s29, s35, and s36) were markedly upregulated with fold changes ranging from 1.7 to 4.5 (log 2), whereas BTH_s12 was downregulated (-3.9 fold change, log 2). Other stress conditions, such as anaerobiosis (CO_2_ atmosphere) and exposure to ethanol or peroxide (H_2_O_2_), downregulated specific sRNAs, but did not modulate expression profiles of other sRNAs. BTH_s13 exhibited reduced expression in response to all three of these stimuli, suggesting that BTH_s13 plays a role in microbial adaptation to these stressors.

**Table 2:** ***B. thailandensis*** **sRNAs identified from microarray analysis of expression in IGRs**

				Expressed region			Fold change	BLAST
sRNA	Chr	5′ Locus	Genes	Length	First probe	Last probe	Antibiotic	distribution
BTH_s1	1	BTH_I0225	><	151	259648	259799	0.45	Btpm
BTH_s2	1	BTH_I0498	<>	147	553257	553404	0.74	Btpm
BTH_s3	1	BTH_I0572	><	50	652705	652755	−0.56	Bt
BTH_s4	1	BTH_I0629	<>	77	725000	725077	−1.53	Bt
BTH_s5	1	BTH_I0805	><	72	919535	919607	−0.72	Bt
BTH_s6	1	BTH_I0981	><	92	1114896	1114988	−0.83	Bt
BTH_s7	1	BTH_I0986	>>	187	1123855	1124042	0.34	Bt
BTH_s8	1	BTH_I1002	<<	57	1139418	1139475	−0.09	Bt
RnaseP	1	BTH_I1231	><	413	1381221	1381634	−0.03	conserved
BTH_s10	1	BTH_I1420	>>	50	1603625	1603675	1.79	Bt
BTH_s11	1	BTH_I1430	>>	304	1614533	1614837	−0.31	Btpm
BTH_s12	1	BTH_I1458	><	30	1655354	1655384	−3.91	Btpm
BTH_s13	1	BTH_I1526	<>	283	1728308	1728591	1.98	Btpm+
BTH_s14	1	BTH_I1552	<>	263	1754006	1754269	−1.13	Btpm
BTH_s15	1	BTH_I1641	>>	161	1850688	1850849	−0.44	Btpm+
BTH_s16	1	BTH_I1756	<>	122	1967019	1967141	1.98	Btpm
BTH_s17	1	BTH_I1826	>>	184	2052202	2052386	−0.48	Btpm
BTH_s18	1	BTH_I1973	><	29	2237631	2237660	−0.60	Bt
BTH_s19	1	BTH_I2094	<>	127	2372778	2372905	1.84	Btpm+
small SRP	1	BTH_I2218	<>	14	2494993	2495007	−0.55	conserved
BTH_s21	1	BTH_I2685	>>	51	3066928	3066979	1.83	Bt
BTH_s22	1	BTH_I2764	><	34	3174991	3175025	0.02	Bt
BTH_s23	1	BTH_I2791	><	194	3207088	3207282	−0.97	Bt
BTH_s24	1	BTH_I2908	>>	67	3344035	3344102	0.51	Btpm+
BTH_s25	1	BTH_I3195	<>	54	3642110	3642164	−1.32	Btpm+
BTH_s26	2	BTH_II0111	<>	59	126516	126575	−0.05	Bt
BTH_s27	2	BTH_II0236	>>	88	287804	287892	1.70	Btpm
BTH_s28	2	BTH_II0378	>>	230	448466	448696	−0.39	Btp
BTH_s29	2	BTH_II0540	>>	169	641739	641908	1.94	Btpm
BTH_s30	2	BTH_II0674	>>	128	789481	789609	0.00	Btpm+
BTH_s31	2	BTH_II0686	>>	143	803355	803498	−1.24	Btpm+
BTH_s32	2	BTH_II0954	<<	38	1133460	1133498	0.08	Bt
BTH_s33	2	BTH_II1164	><	52	1355194	1355246	−0.29	Btpm
BTH_s34	2	BTH_II1180	><	113	1380009	1380122	−0.95	Bt
BTH_s35	2	BTH_II1417	>>	133	1672484	1672617	3.91	Btpm
BTH_s36	2	BTH_II1508	>>	104	1778103	1778207	4.57	Btpm
BTH_s37	2	BTH_II1685	<<	99	2043861	2043960	−0.71	Bt
BTH_s38	2	BTH_II1727	>>	183	2091083	2091266	−0.97	Bt
BTH_s39	2	BTH_II2030	><	70	2477704	2477774	0.16	Btpm+
BTH_s40	2	BTH_II2171	><	107	2670574	2670681	−0.29	Bt

Fold change in response to antibiotics is depicted in Log 2. For BLAST distribution, Bt – found only in *B. thailandensis*; Btp – found in *B. thailandensis* and *B. pseudomallei*; Btpm – found in *B. thailandensis, B. pseudomallei*, and *B. mallei*; Btpm + - found in *B. thailandensis, B. pseudomallei, B. mallei*, and at least one other additional *Burkholderia* species; and conserved – found in all 16 *Burkholderia* strains tested, including *B. thailandensis, B. pseudomallei, B. mallei, B. phytofirmans, B. phymatum, B. phenoliruptrix, B. xenovorans, B. rhizoxinica, B. gladioli, B. glumae, B. vietnamiensis, B ambifaria, B. cenocepacia, B multivorans*, and *Burkholderia sp.*

All the sRNA sequences were blasted against *Burkholderia* genomes from 16 different species to determine their phyogenetic distribution (Table 2). Of the 40 sRNAs, 17 sRNAs were unique to *B. thailandensis.* BTH_s28 was present in both *B. thailandensis* and *B. pseudomallei*. Twelve sRNAs were found in *B. thailandensis* and the two pathogenic strains *B. pseudomallei* and *B. mallei*. Eight other sRNAs were found in other *Burkholderia* species, in addition to *B. thailandensis* and the two pathogenic strains, and only the two known sRNAs, RNaseP and SRP, were present in all *Burkholderia* strains analyzed. We chose to further validate five novel sRNAs, including the *trans*-encoded BTH_s1, s36, and s39, and the *cis*-encoded BTH_s13 and s19, based on their presence in pathogenic *Burkholderia* and/or higher expression in response to antibiotic treatment (Table 2 and Figure 2).

### *Trans*-encoded sRNAs in *Burkholderia*

**Figure 3:**
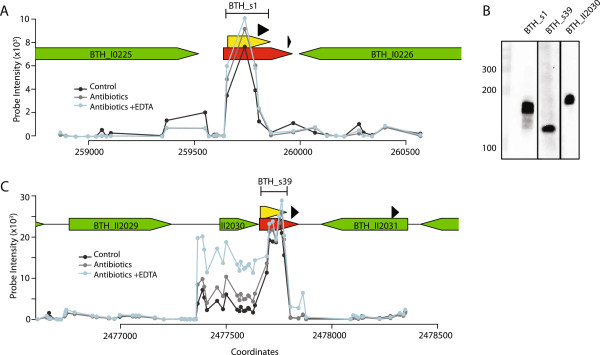
**Genome mapping and expression analysis of** ***trans*** **-encoded BTH_s1 and BTH_s39. (A)** BTH_s1 overlaps with a sRNA prediction from Sipht (red) in the IGR between BTH_I0225 and BTH_I0226 (green). The yellow arrowhead bar maps the sequence boundaries defined by RACE. Probe intensities for BTH_s1 expression in *B. thailandensis* treated with (1) an antibiotics mixture containing sublethal concentrations of carbenicillin, chloramphenicol, erythromycin, and kanamycin (grey line), (2) the antibiotics mixure and 0.7 gm/L EDTA, a standard dosage for chelation therapy (blue line) or (3) untreated control (black line) are depicted. Predicted terminators are denoted as black triangles. **(B)** Northern blot of BTH_s1, BTH_s39, and BTH_I2030 demonstrates that these three genes are expressed as independent single transcripts. The size of the transcripts was determined on a 15% denaturing polyacrylamide gel using a biotinylated RNA ladder. **(C)** BTH_s39 overlaps with a sRNA prediction from Sipht (red) in the IGR between BTH_I2030 and BTH_I2031 (green). The yellow arrowhead bar maps the sequence boundaries defined by RACE. Probe intensities for BTH_s39 expression in *B. thailandensis* for the three different conditions and predicted terminators are denoted as described in **(A)**.

The *trans*-encoded BTH_s1 and BTH_s39 exhibited modestly higher expression levels in response to antibiotic exposure compared to untreated control cells and were expressed independently from their flanking genes (Figure 3). We observed a single major transcript for BTH_s1 using Northern blot analysis in exponentially-growing bacteria (Figure 3B). BTH_s39 was mapped to the 3′ UTR of a conserved protein-coding gene, BTH_II2030, based on prediction of a transcription terminator sequence immediately following BTH_s39 (Figure 3C). However, we demonstrated that BTH_s39 was processed as an independent transcript from BTH_II2030 by Northern blot (Figure 3B). Using rapid amplification of cDNA ends (RACE), we determined the size of BTH_s1 and BTH_s39 to be 205 and 130 nt, respectively, and mapped the genomic coordinates. (Figure 3A and C, yellow arrowhead bars and Additional file 3) Based on gene expression analysis using our *B. thailandensis* microarray, we determined that the expression profiles of BTH_s39 and BTH_II2030 are similar under some growth conditions, such as phosphate starvation and exposure to antibiotics, but significantly differ in response to other stress factors, such as elevated temperature (41°C) or acidic environment (pH 4), suggesting that expression for both transcripts is highly regulated, but can be divergent (data not shown). Importantly, BTH_s39 shares >90% sequence similarity across a wide range of *Burkholderia* species, while BTH_II2030 is conserved only between *B. thailandensis*, *B. pseudomallei*, and *B. mallei*, suggesting that BTH_s39 regulatory function is highly conserved.

**Figure 4:**
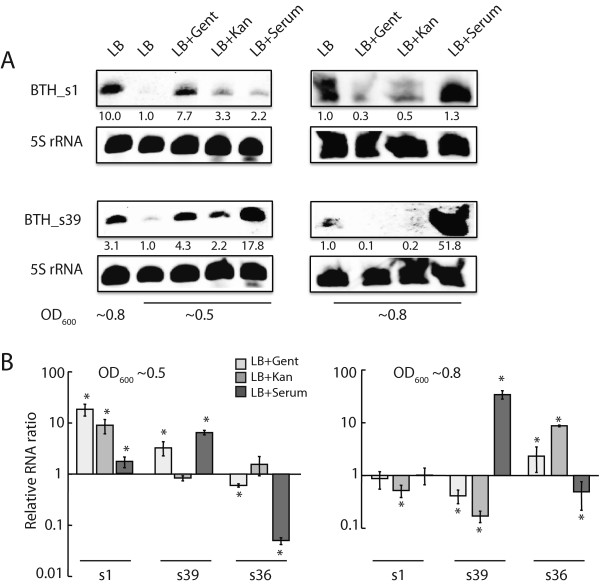
**Expression profiles of** ***trans*** **-encoded sRNAs in response to antibiotics and serum. (A)** Northern blot analysis was performed on 6 μg total RNA isolated from *Bt* CDC272 grown at 37°C to mid-exponential (OD_600_ ~ 0.5, left panel) or early stationary phase (OD_600_ ~ 0.8, right panel) in LB or LB containing 300 μg/ml gentamycin (LB + Gent), 200 μg/ml kanamycin (LB + Kan), or 20% bovine serum (LB + Serum). At OD_600_ ~ 0.5 and ~0.8, the fold change in BTH_s1 and s39 transcript levels (displayed under the RNA bands) was determined as the ratio of sRNA normalized to 5S rRNA for each experimental condition, compared to growth in LB. To evaluate the effect of signal normalization between independent probe annealing experiments, the left panel includes RNA samples from bacteria grown in LB to OD_600_ ~ 0.8 (left panel, first lane) for comparison to transcript levels at OD_600_ ~ 0.5. One representative of four Northern blots performed on RNA isolated from independent experiments is shown. **(B)** Two-step RT-PCR analysis was performed on total RNA isolated from *Bt* CDC272 grown at 37°C to OD_600_ ~ 0.5 (left plot) and OD_600_ ~ 0.8 (right plot). The relative RNA levels are presented as fold change in BTH_s1, s39, and s36 expression levels between LB + treatment compared relative to LB alone. For each sample, the sRNA levels were normalized to 5S rRNA. The amount of antibiotic and serum used was as described in **(A)**. The data represents the average and standard deviation from three independent experiments performed in duplicate. The “*” denotes statistical significance (p < 0.05) between sRNA expression in *B. thailandensis* grown in LB + treatment compared to LB alone.

Both BTH_s1 and BTH_s39 were expressed at low levels in exponentially-growing (OD ~0.5) *B. thailandensis* and reached maximal levels at stationary phase (OD ~0.8) (Figure 4A). Growth of the *B. thailandensis* CDC2721121 strain is inhibited by gentamycin at >300 μg/ml and kanamycin at >200 μg/ml. When exposed to sublethal concentrations of both antibiotics, BTH_s1 and BTH_s39 levels were upregulated in exponentially-growing bacteria, but downregulated in bacteria at stationary phase. These results were further validated by qPCR (Figure 4B), although BTH_s39 exhibited little change in expression in response to kanamycin at exponential phase using this method. A third trans-encoded sRNA, BTH_s36, displayed a different expression profile from BTH_s1 and BTH_s39, with relatively little change in response to antibiotics during exponential growth, but significantly increased expression at stationary phase. In addition to antibiotics, we also stimulated *B. thailandensis* in LB media containing serum and observed strong induction of BTH_s39 and significant reduction of BTH_s36 in both growth phases, which are reversed expression profiles to that seen with antibiotic treatment especially at stationary phase (Figure 4A and B). These data suggest that BTH_s39 and BTH_s36 have opposing regulatory functions in bacterial adaptation to antibiotics and host serum.

**Figure 5:**
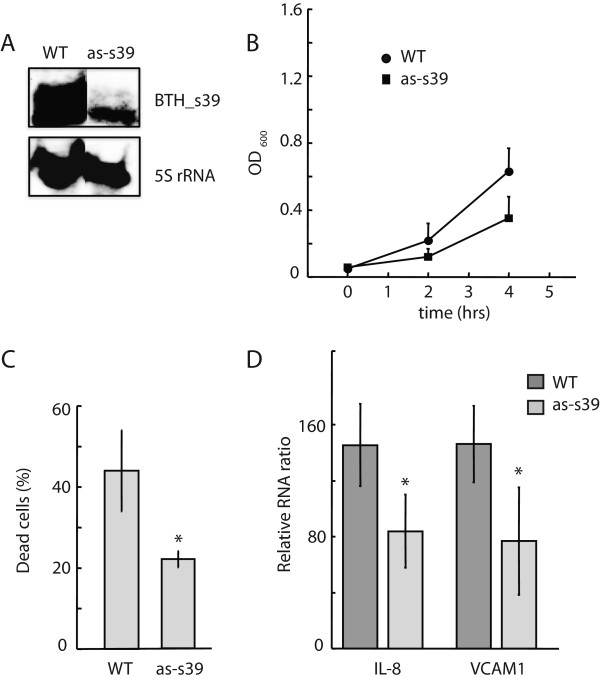
**Knock-down of BTH_s39 inhibits** ***B. thailandensis*** **growth and attenuates host response. (A)** The efficiency of BTH_s39 transcript reduction was evaluated by Northern blot analysis of RNA isolated from wild type *Bt* CDC272 (WT) and from a bacterial clone expressing anti-sense to BTH_s39 transcript (as-s39) grown in LB containing 10% serum. 10 μg total RNA was loaded per sample and 5S rRNA was used as a reference control. **(B)** Growth curves of *Bt* CDC272 (WT) and the as-s39 knock-down strain cultured in RPMI containing 10% serum. Bacterial culture densities (OD_600_) were measured at 2h intervals. The average values of four independent culture samples per bacterial strain are depicted. **(C)** The virulence of wild type *Bt* CDC272 (WT) and the as-s39 knock-down strain was evaluated by infection of THP-1 cells at MOI 10 for two hours, followed by addition of media containing 250 μg/ml kanamycin. The percentage (%) of dead cells was determined 24 hrs post-infection by comparison of total ATP in *Burkholderia*-infected versus uninfected THP-1 cells. The average and standard deviation values were obtained from three independent experiments. The ‘*’ denotes statistical significance (p < 0.05) in % dead cells between the as-s39 knock-down strain compared to WT. **(D)** The effect of BTH_s39 downregulation on the ability of *Bt* CDC272 to elicit an immune response was measured by RT-PCR. THP-1 cells were collected 5 h post infection with *Bt* CDC272 (WT) and the as-s39 knock-down strain at MOI 10. VCAM1 and IL-8 transcript levels were determined by Taqman qPCR using total RNA. The RNA levels are presented as fold change versus untreated THP1 control samples. Data is shown from three independent infection experiments performed in duplicate. The ‘*’ denotes statistical significance (p < 0.05) in IL-8 and VCAM1 expression between the WT and as-s39 knock-down strains.

Based on the high expression levels of BTH_s39 in response to multiple stimuli (Figure 2), we expected that *Burkholderia* defective for BTH_s39 expression may exhibit physiological changes in stress response or virulence. To examine the effect of a BTH_s39 knockdown in *Burkholderia*, we overexpressed an antisense transcript to BTH_s39 and isolated bacterial clones with ~85% reduction in BTH_s39 levels (Figure 5A). BTH_s39-defective *B. thailandensis* grew at a slower rate compared to a wild-type strain in host media containing serum (Figure 5B). We also observed that infection of THP-1 macrophage cells with the BTH_s39-defective strain led to increased host survival compared to infection with the wild-type strain (Figure 5C). Finally, THP-1 cells infected with the BTH_s39-defective strain expressed only half of the chemokine IL-8 and adhesion molecule ICAM-1 transcript levels compared to THP-1 cells infected with the wild-type strain (Figure 5D). Taken together, these data demonstrate that BTH_s39 is required for pathogen adaptation to a host-specific environment containing serum. Exposure to serum has been shown to stimulate the expression of bacterial genes that impact the host immune response [14, 15].

### sRNAs encoded in the 5′UTR of target genes

**Figure 6:**
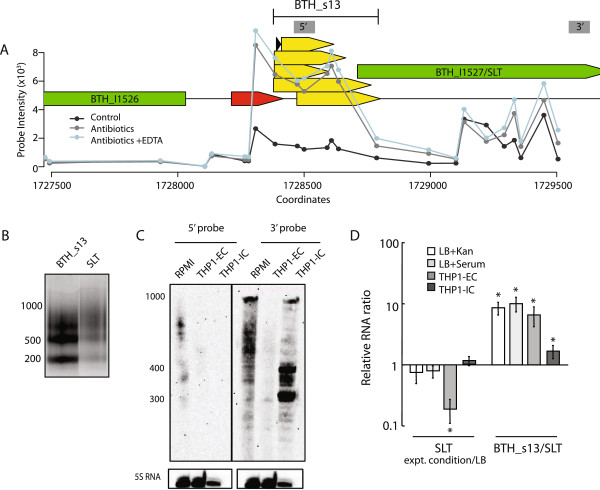
**The** ***cis*** **-encoded BTH_s13 controls BTH_I1527/SLT gene expression by transcript degradation. (A)** BTH_s13 is adjacent to a sRNA prediction from Sipht (red) in the IGR between BTH_I1526 and BTH_I1527/SLT (green). Probe intensities for BTH_s13 expression in *B. thailandensis* treated with the three conditions described in Figure 3A are depicted. The yellow arrowhead bars map the different BTH_s13 sequences obtained by RACE. The grey boxes labeled 5’ and 3’ denote the probe sites used in the Northern blots shown in **C**. **(B)** PCR products were generated by applying RACE to the same cDNA template using primers specific to BTH_s13 and SLT. **(C)** Total RNA (15 μg) was isolated from *Bt* CDC272 grown in host medium (RPMI 10% serum) or derived from the extracellular (THP1-EC) or intracellular bacterial fraction (THP1-IC) of THP-1 infected cells. The Northern blots were derived from a single Northern blot consecutively processed with probes to 5’ BTH_s13, 3’ region of the BTH_I1527/SLT gene, and the 5S rRNA as a loading control. A representative of three independent experiments is shown. **(D)** Total RNA was isolated from *Bt* CDC272 cultured in LB, LB+Kan or LB+Serum, and from the THP1-EC and THP1-IC fractions of THP-1 infected cells. The relative RNA levels of SLT are presented as fold change of SLT transcript detected with the 3’ primer set and were calculated as the transcript ratio between each treatment relative to growth in LB. The BTH_s13/SLT ratio is derived from the transcript levels detected with primer sets corresponding to the 5’/BTH_s13 and the 3’ region of SLT for each condition. The average and standard deviation from three independent experiments perfomed in duplicate are presented. The “*” denotes statistical significance (p<0.05) for SLT expression in *B. thailandensis* grown in LB + treatment compared to LB alone or for BTH_s13 expression compared to SLT.

**Figure 7:**
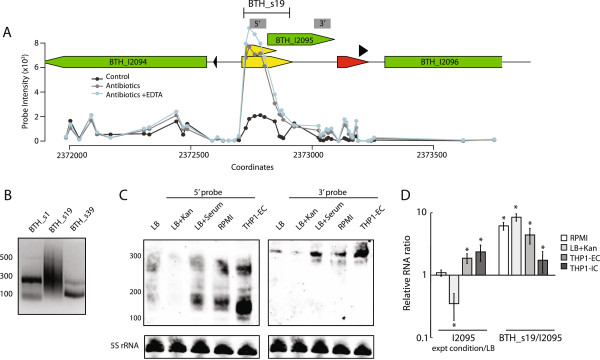
**The** ***cis*** **-encoded BTH_s19 regulates BTH_I2095 gene expression by transcript degradation. (A)** BTH_s19 is located in the IGR between BTH_I2094 and BTH_I2095 (green). Probe intensities for BTH_s19 expression in *Bt* CDC272 treated with the three conditions described in Figure 3A are depicted. The yellow arrowhead bars map the different BTH_s19 sequences obtained by RACE. The grey boxes labeled 5’ and 3’ denote the primers used in Northern blots in Figure 7B. **(B)** PCR products were generated by applying RACE to the same cDNA template using primers specific to BTH_s1, s19, and s39. **(C)** Total RNA (20μg) was isolated from *Bt* CDC272 grown in LB, LB+Kan or LB+Serum, host media (RPMI), or total RNA derived from the THP1-EC fraction of THP-1 infected cells. Results are from a single Northern blot consecutively processed with probes to 5’/BTH_s19 and 3’ region of the BTH_I2095 gene, and 5S rRNA, and is a representative of three independent experiments. **(D)** qPCR was performed on total RNA isolated from *Bt* CDC272 cultured in LB, host medium with 10% serum (RPMI), LB containing 200 μg/ml kanamycin (LB+Kan), and from the extracellular (THP1-EC) and the intracellular fraction (THP1-IC) of THP-1 infected cells. The relative RNA levels are presented as fold change of BTH_I2095 detected with the 3’ primers and were calculated as transcript ratio between each treatment relative to bacteria grown in LB. The BTH_s19/I2095 ratio is derived from the transcript levels detected with primer sets corresponding to the 5’/BTH_s19 and the 3’ region of BTH_I2095 within each condition. The average and standard deviation from three independent experiments perfomed in duplicate are presented. The “*” denotes statistical significance (p<0.05) for BTH_I2095 expression in *B. thailandensis* grown in LB + treatment compared to growth in LB or for BTH_s19 expression compared to BTH_I2095.

During analysis of sRNA sequences, we noted that several IGRs contained potential cis-encoded sRNAs in the 5′ UTR. We selected two sRNAs, BTH_s13 and BTH_s19, that were characterized by a single peak immediately upstream from their poorly-expressed cognate protein-coding genes, BTH_I1527 and BTH_I2095, respectively (Figures 6A and 7A). Both BTH_s13 and BTH_s19 expression were up-regulated >3-fold when *B. thailandensis* was exposed to the antibiotics mixture. We validated the predicted genomic coordinates of BTH_s13 and BTH_s19 using RACE and observed that the resultant PCR products exhibited heterogeneous migration, in contrast to a single predominant band that we observed for BTH_s1 and BTH_s39 (Figures 6B and 7B). Sequencing of the PCR products revealed that BTH_s13 and sBTH_19 were expressed as multiple subspecies of processed transcripts (Figures 6A and 7A, yellow arrowhead bars, and Additional file 3), ranging from 209 to 400 and 133 to 223 nucleotides, respectively. Several of the sRNA subspecies extended into their cognate downstream coding gene by >100nt, suggesting that the sRNA and downstream gene may be expressed as one transcript that subsequently undergoes degradation and/or cleavage by site-specific enzymatic RNA processing as a mechanism for gene downregulation.

The gene immediately downstream of BTH_s13, BTH_I1527, encodes soluble lytic transglycosylase (SLT), an enzyme that cleaves the glycosidic linkage between N-acetylmuramoyl and N-acetylglucosaminyl residues within peptidoglycan in the bacterial cell wall to create space for the insertion of membrane-spanning structures, such as flagella and the Type III secretion system, during cell division [16]. Given that the cell wall is essential for bacterial viability, we expect expression of SLT to require stringent regulation. From RACE analysis, we also observed heterogeneous migration of SLT transcripts (Figure 6B), similar to what we observed for BTH_s13, suggesting that SLT transcripts are also degraded and short-lived. Both BTH_s13 (5′ probe) and SLT (3′ probe) transcripts undergo degradation when *B. thailandensis* is grown in conditioned host media (RPMI), as demonstrated by Northern blot (Figure 6C). To further investigate BTH_s13 expression during pathogenesis, we infected THP-1 macrophages with *B. thailandensis* separated into two fractions: extracellular (THP1-EC) bacteria that remained in the medium, and phagocytosed intracellular (THP1-IC) pathogen released by detergent-induced host cell lysis. Interestingly, the IC fraction exhibited higher levels of full-length SLT (1000nt) and 300-400nt transcript fragments, compared to barely detectable SLT transcript in the EC fraction, suggesting that SLT expression is needed for intracellular survival and proliferation during infection. For extracellular bacteria, cell wall fortification is likely required to resist microbicidal peptides produced by macrophages, thus leading to significant reduction of SLT expression.

By qPCR, we observed that the levels of BTH_s13 were ~10-fold higher than the SLT transcripts in bacteria grown in kanamycin (LB + Kan), serum-containing media (LB + Serum), or the EC fraction (Figure 6D). In the IC fraction however, the increase in SLT transcript accumulation was offset by a ~4-fold decrease in the BTH_s13 levels as compared to the EC fraction or bacteria grown in host media. In sum, our results suggest that BTH_s13 regulates SLT gene expression on a posttranscriptional level to fine tune SLT protein accumulation.

We also investigated BTH_s19, located in the 5′UTR of the coding gene BTH_I2095. From RACE analysis, we recovered two BTH_s19 subspecies that extended into the 5′ end of BTH_I2095 by either +70 or +150nt. (Figure 7, yellow arrowheads and Additional file 3) The RACE and Northern blot data collectively indicated that full-length BTH_s19 transcripts (~300nt) were expressed in *B. thailandensis* grown in LB during log phase and in response to serum and conditioned host media (RPMI), but were subjected to rapid degradation (<200nt) (Figure 7C, 5′ probe). The relatively low level of full-length transcript may be indicative of tight gene regulation, suggesting that BTH_I2095 encodes a protein whose accumulation may burden the cell. We also observed that BTH_s19 and BTH_I2095 transcript levels were markedly suppressed when *B. thailandensis* was cultured in LB containing inhibitory concentrations of kanamycin (200 μg/ml), suggesting a role in adaptation to antibiotic exposure (Figure 7C). This role was further underscored by the significant accumulation of BTH_s19 transcripts in bacteria grown under suboptimal antibiotic concentrations (Figure 7A). Unlike BTH_s13, BTH_s19 expression was up-regulated in the EC fraction of *B. thailandensis* compared to bacteria cultured in LB or RPMI (Figure 7C). By qPCR, we also observed that BTH_s19 accumulation was relatively reduced in the IC fraction compared to levels of BTH_I2095 transcripts (Figure 7D). Taken together our data on *cis*-encoded BTH_s13 and BTH_s19 present a conserved mechanism of post-transcriptional gene dosage control by destabilization of downstream cognate mRNAs.

Notably, alignment of BTH_s13 and BTH_s19 sequences across pathogenic *Burkholderia* indicated a higher sequence identity (>95%) compared to their cognate downstream coding genes, BTH_I1527/SLT and BTH_I2095 (~92% and ~94% sequence identity respectively), suggesting that these sRNAs may have conserved regulatory functions.

### Prediction of sRNA targets using CopraRNA

To identify potential target genes for *B. thailandensis* sRNAs, we used the Comparative Prediction Algorithm for sRNA Targets (CopraRNA) on the experimentally-validated sRNAs [17] (Table 3). The top targets for both *cis*-encoded sRNAs, BTH_s13 and s19, were correctly identified as their respective downstream genes. Interestingly, the number of targets under the q-value threshold for the *cis*-acting sRNAs BTH_s13 and s19 (43 and 52 targets, respectively) was larger compared to the *trans*-encoded sRNAs BTH_s1, s36, and s39 (5, 4, and 13 targets, respectively). Many of these predictions may be valid targets. For example, the second target prediction of BTH_s19 is the osmolarity response regulator *ompR* (BTH_I2094), which is coded on the opposite strand 171 bases upstream. Given that the predicted binding site is at position -199 to -153, it is likely that BTH_s19 is an antisense regulator of the 5′ UTR of *ompR* as well. For trans-encoded sRNAs, there is little correlation between the genomic location of the sRNA and its cognate mRNA targets. The top targets for the trans-encoded BTH_s1 and s39 were phage proteins with multiple copies on three or more genomic islands. BTH_s39 is found immediately downstream of BTH_II2030 in the 3′ UTR region, but this gene was not found in the list of top 100 potential targets for BTH_s39, further confirming the independence of the transcript as seen by Northern blot (Figure 3). Genes regulating key pathways for energy production, phosphorus metabolism, and oxidative stress were found amongst the top ten predicted targets for BTH_s39, which is consistent with the s39 expression profile (Figure 2).

**Table 3:** **Target predictions with q-value < 0.5 from CopraRNA and details of the top significant matches**

sRNA#	Total	Target	q-value	Energy	sRNA	mRNA	Definition
1	5	BTH_I0117	0.03528	−23.340	164..191	−56..-27	gp31
		BTH_I1738	0.04661	−24.158	159..185	5..29	hypothetical protein
		BTH_II0675	0.04661	−22.376	164..192	−79..-54	aspartate-semialdehyde dehydrogenase Asd
		BTH_II1093	0.07311	−15.108	159..185	−170..-140	Ser/Thr protein phosphatase
		BTH_I2063	0.07311	−20.982	163..189	15..47	L D-carboxypeptidase A LdcA
13	43	BTH_I1527	0.00000	−10.280	273..298	−69..-47	transglycosylase SLT domain-containing protein
		BTH_I1319	0.00341	−24.103	133..193	−128..-73	tRNA delta(2)-isopentenylpyrophosphate transferase MiaA
		BTH_II1420	0.04465	−13.661	250..260	8..18	hypothetical protein
		BTH_I0403	0.04465	−17.257	201..238	−32..16	hypothetical protein
		BTH_I0216	0.05922	−13.297	50..88	−43..1	ATP-dependent protease domain-containing protein
		BTH_I0248	0.06071	−13.886	159..190	−43..-14	flagellar rod assembly protein/muramidase FlgJ
		BTH_I0386	0.09037	−15.127	175..210	−158..-122	lipoyl synthase LipA
		BTH_I1901	0.09529	−12.224	386..423	36..77	phosphoesterase
		BTH_II1524	0.19532	−11.366	298..319	−65..-46	acetyltransferase
		BTH_II0792	0.28030	−13.522	159..200	−64..-28	endo-1 4-D-glucanase
19	52	BTH_I2095	0.00000	−15.900	60..73	−53..-40	hypothetical protein
		BTH_I2094	0.00002	−63.187	1..47	−199..-153	osmolarity response regulator OmpR
		BTH_I0740	0.10640	−22.588	16..86	16..96	3-deoxy-manno-octulosonate cytidylyltransferase KdsB
		BTH_II0267	0.10640	−21.664	179..222	−56..-10	rhsD protein
		BTH_I1820	0.10640	−23.421	16..86	16..94	histidine ammonia-lyase HutH
		BTH_I3024	0.12057	−28.055	123..217	−9..86	shikimate kinase AroK
		BTH_II0267	0.14266	−21.664	179..222	−56..-10	rhsD protein
		BTH_II2237	0.14266	−18.893	61..77	−176..-160	MmgE/PrpD family protein
		BTH_I2472	0.14266	−18.732	28..82	−63..-1	ribose operon repressor RbsR
		BTH_I2817	0.16248	−17.933	177..220	−197..-156	short chain dehydrogenase
36	4	BTH_II1509	0.00117	−14.418	67..87	−25..-4	MgtC family protein
		BTH_I0673	0.43725	−40.259	19..86	−186..-115	DNA-binding response regulator
		BTH_II1525	0.43725	−28.046	51..87	−88..-52	transporter
		BTH_I3260	0.43725	−17.695	20..87	−135..-78	ATP-dependent DNA helicase Rep
39	13	BTH_I3274	0.00865	−13.184	87..121	−160..-130	phage integrase family site specific recombinase
		BTH_I0763	0.05339	−12.897	61..98	−132..-97	ATP-dependent Clp protease ATP-binding subunit ClpA
		BTH_I1300	0.11325	−8.769	115..122	64..71	DNA repair protein RecN
		BTH_I1594	0.21103	−11.949	82..105	63..86	cold-shock domain-contain protein
		BTH_I3232	0.21103	−9.560	117..125	54..62	phage integrase family site specific recombinase
		BTH_I1062	0.26265	−17.617	67..83	47..64	NADH dehydrogenase subunit B
		BTH_I0425	0.26265	−13.815	87..121	−109..-70	hypothetical protein
		BTH_I2768	0.28525	−8.492	61..83	69..94	phosphate regulon transcriptional regulatory protein PhoB
		BTH_I1282	0.38899	−9.640	4..12	−83..-75	catalase/peroxidase HPI
		BTH_II0671	0.48561	−12.524	112..127	27..46	bkd operon transcriptional regulator

## Discussion

In this study, we identified 40 *B. thailandensis* sRNAs that are differentially expressed in response to 54 distinct growth and stimulatory conditions. The sRNAs included highly-expressed sRNAs in practically all growth conditions (e.g. BTH_s39, RNaseP, and SRP), poorly-expressed sRNAs (e.g. BTH_s25 and s12), and stress-induced sRNAs specifically regulated in response to toxic stimuli (e.g. BTH_s13 and s36). Of the 38 novel sRNAs, 20 displayed high sequence conservation between *B. thailandensis* and the two pathogenic strains, *B. pseudomallei* and *B. mallei*. The five sRNAs we characterized in-depth were in this category. In particular, BTH_s39 is one of the most highly-expressed sRNAs in cells exposed to environmental stress factors and may play a conserved essential role similar to the two highly-expressed known sRNAs, RNaseP and SRP. Indeed, knockdown of BTH_s39 expression by antisense transcripts led to inhibition of *B. thailandensis* growth in a host-specific environment and attenuation of host cell death and cytokine expression upon host infection (Figure 5), indicating that BTH_s39 functions both in adaptation to environmental growth and pathogenicity mechanisms.

sRNAs have been identified in studies of other *Burkholderia* species. Deep sequencing of *Burkholderia cenocepacia* grown in two different conditions, a soil environment and an infection model, led to identification of thirteen novel sRNAs [18]. Twelve of these sRNAs were specifically induced in soil, suggesting that these sRNAs modulate bacterial survival under suboptimal growth conditions. A recent comprehensive transcriptomics study of *B. pseudomallei* grown under 82 environmental conditions was performed using whole genome tiling [19]. Of 766 condition-dependent sRNAs identified, 150 shared high sequence identity and chromosomal synteny with *B. thailandensis.* Similar to the *B. cenocepacia* study, relatively few (<10) sRNAs were associated with host infection, although media supplemented with host factors stimulated expression of a higher number of sRNAs (~50 upregulated and ~50 downregulated). These differences in sRNA expression levels under host conditions may stem from competing stress factors in the host environment, such as low O_2_ or iron levels, change in carbon source, and exposure to microbicidal agents. Furthermore, the comparatively low abundance of bacteria-derived sRNAs compared to the host-derived RNA population can confound detection of less abundant sRNAs required for bacterial adaptation to the host. Moreover, sRNAs with high sequence conservation between bacterial species may not follow the same expression pattern under equal growth conditions due to distinct genetic backgrounds.

There are still challenges to be addressed in the discovery and classification of sRNAs. In many cases, slight discrepancies in growth conditions can result in markedly different sRNA expression profiles by independent research groups [20]. Many filtering criteria exclude sRNAs <100 bp. In the case of BTH_s39, we found that the most abundant transcript was 95 bp. BTH_s39 was not identified by the *B. pseudomallei* transcriptome analysis, which had excluded RNA transcripts <110 bp [19]. Other studies exclude RNA transcripts located in the 5′ or 3′ UTRs of annotated genes, assuming that these RNAs were *cis* regulatory elements of the flanking gene. We demonstrated that BTH_s39 was expressed independently of its upstream flanking gene BTH_II2030 under various growth conditions. We included these transcripts since they can resist degradation and accumulate in bacteria, and thus have the potential to exert regulatory control not only on their flanking genes, but also target other *trans*-encoded transcripts.

Although the predicted sRNA targets from the CopraRNA target identification analysis remain to be experimentally validated, it was surprising to identify more potential targets for the *cis*-acting BTH_s13 and BTH_s19 sRNAs compared to the *trans*-acting BTH_s1 and BTH_s39. Another distinctive feature of the *cis*-acting sRNAs characterized in our study was the degree of their sequence conservation (>95%) across pathogenic *Burkholderia*, which was greater than that of their flanking cognate genes. We demonstrated that the processing of BTH_s13 and BTH_s19 caused degradation of the downstream gene, and the ratio between the sRNAs located in the 5′UTR versus the transcript levels of the downstream coding gene was always >3 (Figures 6 and 7). These observations indicate that the *cis*-acting sRNAs are likely to be co-transcribed with their flanking cognate gene, and sRNA processing executes post-transcriptional gene dosage control.

The sequence conservation of BTH_s13 and BTH_s19 between several *Burkholderia* species suggests that these sRNAs play an essential regulatory role. One possible function of these sRNAs is the riboswitch, a highly-conserved family of *cis*-regulatory RNAs by both sequence and structure, that responds to changes in intracellular concentrations of metabolites and secondary messengers to attenuate transcription/translation by competing with either a terminator stem-loop or a Shine-Delgarno element [21]. To date, only the *glmS* riboswitch is known to undergo ligand binding-induced self-cleavage that destabilizes the mRNA in which it resides, leading to mRNA degradation by RNase J1 [22]. We are currently investigating whether BTH_s13 and BTH_s19 can function as riboswitches via binding to specific metabolites or secondary messengers to protect bacteria from accumulation of cytotoxic transcripts [23].

We also observed that BTH_I2095, the downstream cognate gene of BTH_s19, is in close proximity to genes activated by oxidative stress. BTH_I2095 is located immediately downstream of the two component system RisS/RisA, which has been linked to *Bordetella* pathogenicity and is optimally expressed in the intracellular niche [24]. Genes that encode for antioxidant protein (BTH_I2092), alkyl hydroperoxide reductase D (BTH_I2091), and transcription-repair coupling factor (BTH_I2088) are located further upstream of RisS. These genes are induced upon exposure to antibiotics, oxidizing agents (H_2_O_2_), iron/manganese depletion, or ionizing radiation [25–27]. We found that BTH_s19 accumulated in *B. thailandensis* exposed to a sub-lethal dose of kanamycin included in a cocktail of antibiotics to which the bacteria is naturally resistant (Figure 7A), whereas BTH_s19 levels were significantly reduced in bacteria exposed to inhibitory concentrations of kanamycin (Figure 7B-C). These data support a role for BTH_s19 in mediating antibiotic resistance.

The downstream cognate gene of BTH_s13, SLT, functions in bacterial cell wall remodeling during cell division and the formation of macromolecule virulence complexes such as the secretion systems, T3SS and T6SS [28]. In this study, we demonstrated that SLT transcription was inhibited when bacteria was exposed to conditioned host medium, which contains antibacterial peptides, indicating that SLT expression is downregulated when bacterial cell walls need to be fortified in order to resist microbicidal peptides produced by immune cells (Figure 6). In contrast, SLT levels were relatively higher in intracellular bacteria, which correlates with a need for cell wall remodeling upon T6SS formation and secretion of virulence proteins, to support intracellular survival and proliferation*.* The T6SSs were identified as major virulence factors essential for intracellular growth, actin polymerization, and formation of multi-nucleate giant cells [29, 30]. Expression of T6SS gene clusters in pathogenic *Burkholderia* was found to be limited exclusively to intracellular bacteria [31]. Interestingly, we observed inhibition of 5′ UTR degradation (lower ratio of BTH_s13 to SLT transcript) in intracellular bacteria, suggesting that BTH_s13 may serve as a post-transcriptional regulator of SLT to control production of an autolytic enzyme in response to specific growth conditions.

## Conclusions

These studies provide a broad analysis of differential *Burkholderia* sRNA expression profiles and illustrate the complexity of bacterial gene regulation in response to different environmental stress conditions. Given that a specific sRNA profile is expressed only in response to specific stressors, it may be the case that the current list of annotated sRNAs represents only a small fraction of the total sRNA repertoire that can possibly be expressed. We characterized trans-encoded sRNAs, BTH_s1 and s39, that exhibited differential expression profiles dependent on growth phase and cell stimuli. The downstream mRNA targets for most bacterial trans-encoded sRNAs remain unknown. We expect that bioinformatics approaches such as the CopraRNA algorithm can downselect candidate mRNA targets for follow-on experimentation. We are currently validating the mRNA targets for specific sRNAs predicted by CopraRNA. We have also found that cis-encoded sRNAs, such as BTH_s13 and s19, may be expressed as single transcripts in tandem with their cognate downstream genes and subsequently undergo RNA degradation. This mechanism represents a novel RNA-mediated strategy for post-transcriptional gene dosage to tightly control expression of cognate downstream genes.

We expect that further functional and mechanistic studies of regulatory RNAs will reveal additional mechanisms of gene regulation. Our lab has recently performed single molecule fluorescence *in situ* hybridization (smFISH) on a novel *Y. pestis* sRNA, YOP_s8, implicated in virulence, to quantify precise sRNA copy number and model dynamics of sRNA expression in response to a temperature shift [32]. A comprehensive approach to analyze sRNA function and dynamics will provide a better mechanistic understanding of the complexity of sRNA-mediated gene expression in response to different niche stressors.

## Methods

### Microarray study

We conducted 162 independent experiments on *B. thailandensis* E264 using 54 distinct growth conditions. Experiments were performed in four classes of media: nutrient broth, M9-minimal media, Luria broth, and various host conditions such as brain-heart infusion, calf serum, or tryptic soy broth. There were 101 experiments in four time course arrays measuring temperature increase, pH drop, salt increase, and phosphate starvation (Table 1). Each condition was grown at 37°C, except the initial temperature experiment at 25°C, and replicated five times, except at pH 4.5 (three replicates). Six of the experimental replicates from this group were excluded from the analysis due to outliers in quality assessment. In order to survey a diverse range of environmental and stress conditions, another 10 replicated and 23 unreplicated treatments were also tested (Table 1). For conditions ID49 and ID50, *B. thailandensis* was grown in the presence of a mixture of antibiotics, including carbenicillin (100 μg/ml), chloramphenicol (30 μg/ml), erythromycin (200 μg/ml, and kanamycin (50 μg/ml). Total bacterial RNA for each experimental condition was extracted using the TRIzol method.

A custom Affymetrix microarray was designed using *Burkholderia thailandensis* strain E264 gene annotations from Pathema [33]. The array included 8711 probe sets targeting 5557 protein-coding genes and 2908 IGRs longer than 90 bases. The probes within the IGRs were designed to detect expression on the plus strand. The Pathema annotations are included in Additional file 4. The microarray experiments were performed by the Genomics and Microarray Core at the University of Colorado, Denver. The Affymetrix CEL files were loaded into R [34] using the simpleaffy package [35]. Quality was assessed using plots of normalized unscaled standard error and relative log expression. The microarray data were corrected for background noise with the RMA algorithm and replicates were normalized using the quantile method. The perfect match intensities were averaged across replicates for all 95303 probes and 54 growth conditions. Additional file 5 contains R scripts and additional plots detailing the microarray analysis.

### sRNA identification

sRNA candidates were initially identified using the probe data from the four time course experiments (Table 1). The IGRs were sorted by the range between the probe with the lowest intensity and the probe with the second highest intensity (to avoid spikes caused by single probes with high expression levels). The resulting probe patterns and genome features from the top 500 IGRs were then plotted in R to identify candidate sRNAs (see Additional file 1 for plots from the first 100 IGRs).

The following genome features, 5634 CDS, 12 rRNAs and 58 tRNAs collected from Pathema (Additional file 4), were included in the plots. All four gene prediction programs at NCBI (ftp.ncbi.nih.gov/genomes/Bacteria/Burkholderia_thailandensis_E264_uid58081) were checked to add new features (i.e. those without an exact match to an existing feature) in the following priority order: RefSeq, Prodigal, Genemark, and Glimmer (1011, 1083, 1726 and 1814 additional annotations, respectively). Non-coding sRNAs were added from publicly-available databases, including Rfam [36], Sipht [37] and NAPP [38]. There are 28 new sRNA families in Rfam, 449 small RNA predictions from SIPHT and 409 predictions from NAPP. Finally, we used TransTermHP [39] to predict 4098 regions with rho-independent transcription terminators (using version 2.0 with --all-context and –p expterm.dat options).

The following color schemes were used in the genome plots: protein-coding regions (green), ribosomal RNAs and genes (blue), tRNAs (orange), non-coding sRNAs (red), and terminators (black). The Pathema genes were labeled using the BTH_I and BTH_II locus tag designations representing chromosome I and II. The RefSeq genes with alternate start sites were labeled using the same locus tag and an “R” suffix. The Prodigal, Genemark, and Glimmer genes were marked with unique ids and prod, gmrk, and glim prefixes. The plot lines from the 21 conditions in the four time course experiments were colored by increasingly darker shades of red, gray, green and blue to represent the temperature increase, salt increase, pH drop and phosphate starvation, respectively, in Additional files 1 and 2. In order to visualize the impacts of remapping probe sets to new gene predictions, the probes on the plus strand were marked by circles and probes on the minus strand with triangles.

The sRNA sequences (see Additional file 6) were compared against 120 *Burkholderia* genomes downloaded on Oct. 19, 2012 using blastn without filters or soft-masking. Results with >60% coverage were divided into four categories for down-selection: (1) unique to *B. thailandensis*, (2) present in pathogenic *B. pseudomallei* group*,* (3) found in many *Burkholderia* species, and (4) conserved in all 16 *Burkholderia* species.

### Quantitative RT-PCR

For the experimental validation studies, we used *B. thailandensis* CDC2721121, a clinical isolate that shares 99.4% similarity of 16sRNA gene sequence with the environmental E264 strain [40]. Due to genomic acquisition of the capsular polysaccharide cluster, *Bt* CDC272 exhibits several *B. pseudomallei*-like phenotypes, such as colony wrinkling, resistance to human complement binding, and survival within macrophages [41]. Total RNA was isolated from *Bt* CDC272 by incubation of cells in 100 μl TE buffer containing 1000 U lysozyme for 15 min at room temperature, followed by application of the miRNeasy kit (QIAGEN). Contaminating genomic DNA was removed by two consecutive incubations using the DNA-free DNase Treatment and Removal kit (Applied Biosystems). Resultant RNA concentrations were quantified using a ND 1000 Nano-drop spectrophotometer. cDNA was synthesized using 2 μg total RNA and the miRCURY LNA Universal RT kit (Exiqon) and diluted (1:5) in nuclease-free water (Ambion). Three μl of cDNA were incubated with *Power* SYBR Green PCR Mastermix (Applied Biosystems) and transcript-specific forward and reverse primers (final concentration 600 pmol) for each RT-PCR reaction. The RT-PCR was performed using an ABI 7500 Fast System (Applied Biosystems) with the following cycling conditions: 5 min denaturation step at 95°C followed by 40 amplification cycles, consisting of 15 s at 95°C and 60 s at 58°C. Gene transcript levels between different experimental conditions were compared to bacterial strains grown in standard LB media at 37°C with aeration at equal cell density (OD_600_) and were normalized to the 5S rRNA levels. For the s13 and s19 qPCR analysis, we also applied a second internal control, the newly discovered s3, which is not as abundant as 5S rRNA, and whose expression levels remain unchanged in many experimental conditions including exposure to antibiotic. Samples were run in triplicate and relative RNA ratios were calculated using the ΔΔC_T_ method. The primer sequences of sRNAs of interest and the internal control gene 5S rRNA are listed in Additional file 7. RT-PCR products were cloned into the pGEMT vector and sequenced to validate sequences.

### Preparation of extracellular and intracellular bacterial fractions

THP-1 cells (2×10^7^) were cultured in RPMI supplemented with 10% fetal bovine serum (RPMI-10) and pretreated with 100 nM PMA 72h prior to infection. Media was replaced 48 h post-PMA treatment. Overnight cultures of *Bt* CDC272 were diluted to OD_600_ ~ 0.2 and incubated at 37°C for 2h to obtain bacteria in the exponential growth phase. Differentiated THP-1 cells were infected with *B. thailandensis* at MOI 20. For control samples, an equal amount of bacteria was used to inoculate 20 ml RPMI-10 media without host cells under static conditions at 37°C and 5% CO_2_. At 3 h post-infection, the conditioned media was collected from experiment and control samples and subjected to two sequential centrifugation steps at 1,200 rpm for 2 min to remove carry over non-adherent host cells, followed by 4,000 rpm for 10 min to pellet extracellular bacteria. Bacterial pellets were treated with 0.1% Triton in 1×PBS for 2 min to lyse carryover host cells, washed in 10 ml PBS, and collected by centrifugation at 4,000 rpm for 10 min. Bacterial pellets were resuspended in 200 μl of TES (10 mM TrisHCl, pH 7.5, 1 mM EDTA, and 100 mM NaCl) buffer containing 10 μl of Ready-Lyse Lysozyme solution and incubated for 15 min at room temperature. Total RNA was isolated using TRIzol reagent (1 ml per 10^7^ bacterial cells), phenol/chloroform extraction, and isopropanol precipitation from the aqueous fraction.

To obtain the intracellular bacteria fraction, infected THP-1 cells were collected after removal of conditioned media containing extracellular bacteria, washed with 20 ml PBS five times, and further incubated in fresh RPMI-10 for 3 additional hrs (total 6 h exposure to bacteria). THP-1 cells were collected by centrifugation and incubated in 10 ml TES buffer containing 10 mg/ml lysozyme for 30 min at 37°C and 5% CO_2_ to weaken the cell wall of the extracellular bacteria. THP-1 cells were then washed with PBS, incubated in 0.1% Triton for 5 minutes, and subjected to two centrifugation steps, 2 min at 1,200 rpm and 10 min at 4,000 rpm to collect bacteria associated with host cells. Total RNA was isolated as described above.

### Generation of BTH_s39 knockdown *in Bt* CDC272

The BTH_s39 sequence was synthesized via PCR using *Bt* CDC272 genomic DNA and the following forward and reverse primers: 5′GTATTGTGGGGACCACCTCT3′ and 5′AAGCGGCTTGGCTTGCTGCAACGGCT3′. PCR products were cloned into pGEMT and sequenced. Clones with anti-sense orientation to BTH_s39 that were flanked by PstI on the 5′ and SphI on the 3′ were used as donors to generate inserts for the pMo168 expression vector (Addgene plasmid 27389). The anti-sense to BTH_s39 sequence (as-s39) was cloned in the 3′UTR of the XylE reporter gene expressed from a constitutive tacI promoter [42]. The pMo168-as-s39 vector was electroporated into *Bt* CDC272 cells, and clones were isolated on LB agar plates containing 200 μg/ml kanamycin. Bacterial clones carrying the expression vector were validated using 0.5 M pyrocatechol administered drop wise on the colonies. Expression of the antisense s39 transcript was validated using Northern blot with a sense BTH_s39 biotinylated probe: 5′-BiosgTAGGCATTAGCCAGCCACAACGGCT3′. The efficiency of *Burkholderia*-induced killing of THP-1 cells by the BTH_s39-deficient strain was determined using the Cell Titer Glo kit (Promega). VCAM1 and IL-8 transcript levels were determined by Taqman qPCR using total RNA.

### Northern blot analysis

The size and expression levels of candidate sRNAs were assessed by Northern blot analysis using total RNA isolated from *Bt* CDC272. RNA samples were separated on 6% TBE-Urea polyacrylamide gels by electrophoresis. A poly-A tailed RNA ladder (0.1-2 Kb RNA Ladder, Life Technologies) was run alongside the samples to determine the size of detected transcripts. RNA was electro-transferred and UV cross-linked to a Hybond-N Plus membrane (Amersham, GE Healthcare Life Sciences), air-dried at 80°C for 30 min, and probed with biotinylated DNA oligonucleotides (Integrated DNA Technologies) complementary to the candidate sRNA or the poly-A ladder. Hybridization proceeded overnight in ULTRAhyb buffer (Ambion, Life Technologies) at 45°C, and signal was detected using the Pierce Chemiluminescent Nucleic Acid Detection Module (Thermo Scientific) and the ChemiDoc XRL imaging system (Bio-Rad). Following analysis, membranes were stripped by incubation in boiling water for 5 min and hybridized to a 5S rRNA probe. Expression was estimated relative to the 5S rRNA based on band intensity analysis using the ChemiDoc XRL software.

### Mapping of sRNA 5′- and 3′-ends using rapid amplification of c-DNA Ends (RACE)

The precise size and genomic coordinates of candidate sRNAs were determined by RACE using circularized RNA. Total RNA was isolated from *Bt* CDC272 at exponential phase (OD_600_ ~ 0.5). 500 ng RNA treated with the DNA-free DNase Treatment and Removal kit (Applied Biosystems) were incubated with 40U of T4 RNA ligase I (New England Biolabs) overnight at 17°C in a 25 μl total volume containing 1× RNA ligase buffer, 8% DMSO, 10 U of RNase Inhibitor, and RNase-free water. The circularized RNA was purified using the Minelute RNA kit (QIAGEN) and subjected to RT-PCR using specific outward primers (Additional file 7) and the SuperScript One-Step RT-PCR kit (Invitrogen). RT-PCR products were separated on 1.5% TBE agarose gels, and specific bands were extracted, cloned into the pGEMT vector, and sequenced. Sequences were compared to the *B. thailandensis* E264 genome.

### Prediction of sRNA targets

We predicted sRNA targets using the Comparative Prediction Algorithm for sRNA Targets (CopraRNA) server [17]. Since CopraRNA utilizes phylogenetic information from an extended model of sRNA–target evolution, we analyzed the five sRNAs chosen for experimental validation since they were also present in *B. pseudomallei* and *B. mallei*. We applied Blastn at NCBI to find sRNA homologs in five or more *Burkholderia* strains as input and used the default settings on the website to extract interactions from -200 to 100 of the start codon.

### Availability of supporting data

The microarray data set supporting the results of this article is available in the NCBI Gene Expression Omnibus database under accession number GSE56502 and can be accessed at http://www.ncbi.nlm.nih.gov/geo/query/acc.cgi?acc=GSE56502.

## Electronic supplementary material

Additional file 1: **Expression plots from IGRs.** The top 100 IGRs were sorted by the range between the probe with the lowest intensity and the probe with the second highest intensity using probe data from the 21 distinct conditions in the four time course arrays. Plot colors and labels are described in the Methods. (PDF 7 MB)

Additional file 2: **Expression plots of sRNAs.** Expression plots are depicted of 40 sRNA candidates using probe data from 21 distinct conditions in the four time course arrays. Plot colors and labels are described in the Methods. (PDF 2 MB)

Additional file 3: **sRNA sequences obtained from RACE.** RACE sequences obtained from sequencing of BTH_s1, s13, s19, s27, s36, and s39. (DOCX 23 KB)

Additional file 4: **R code for microarray analysis.** R code to load *B. thailandensis* arrays is described using the simpleaffy package, quality assessment plots, background correction, and summarization. (ZIP 323 KB)

Additional file 5: ***B. thailandensis*** **annotations from Pathema.** Pathema gene annotations downloaded on Sep 20, 2012 from the Pathosystems Resource Intergration Center (http://www.patricbrc.org). (DOCX 195 KB)

Additional file 6: **FASTA file of sRNA sequences.** FASTA file of 40 sRNA sequences identified in intergenic regions. (ZIP 3 KB)

Additional file 7: **Primer sequences.** The primer sequences for sRNAs and the internal control gene 5S rRNA for Northern blots and qPCR. (PPTX 80 KB)

## Abbreviations

EC: Extracellular
IC: Intracellular
IGR: Intergenic region
PAMP: Pathogen-associated molecular pattern
PCR: Polymerase chain reaction
RACE: Rapid amplification of cDNA ends
SLT: Soluble lytic transglycosylase
sRNAs: small RNAs
UTR: Untranslated region.
