# Supplementary figures and images for: Differential expression of small RNAs from Burkholderia thailandensis in response to varying environmental and stress conditions
Source: BMC Genomics. 2014 May 19;15(1):385. doi: 10.1186/1471-2164-15-385 (PMC4035088; doi:10.1186/1471-2164-15-385)

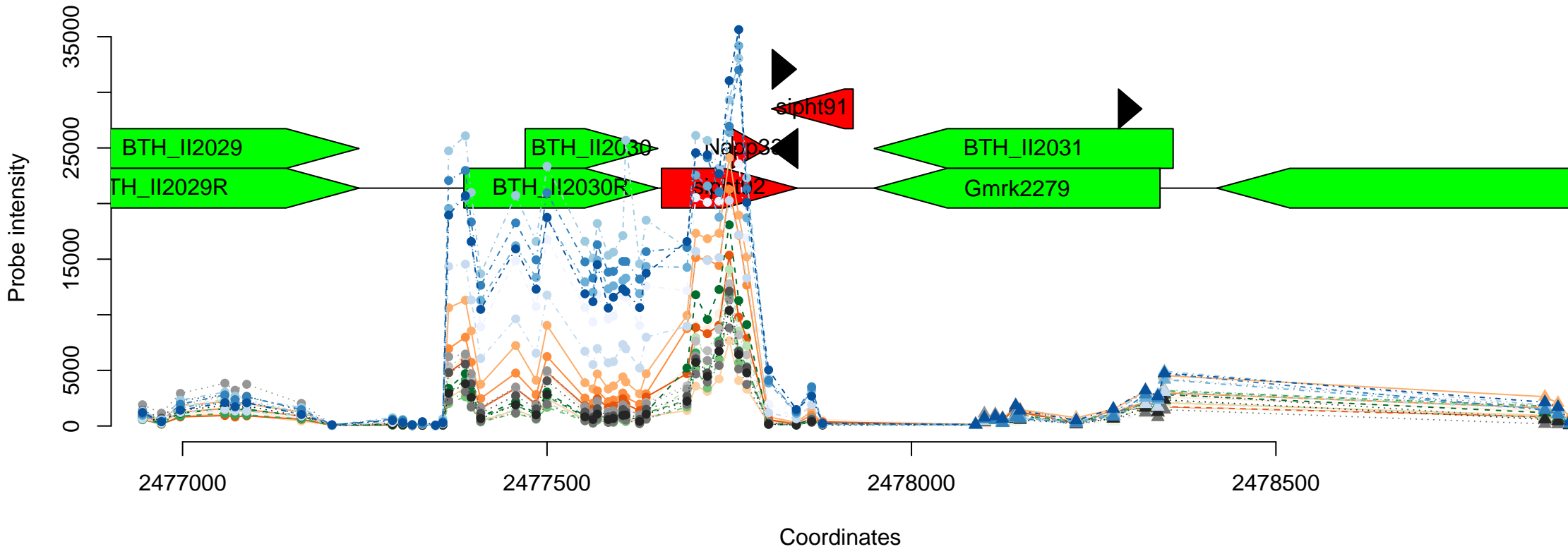

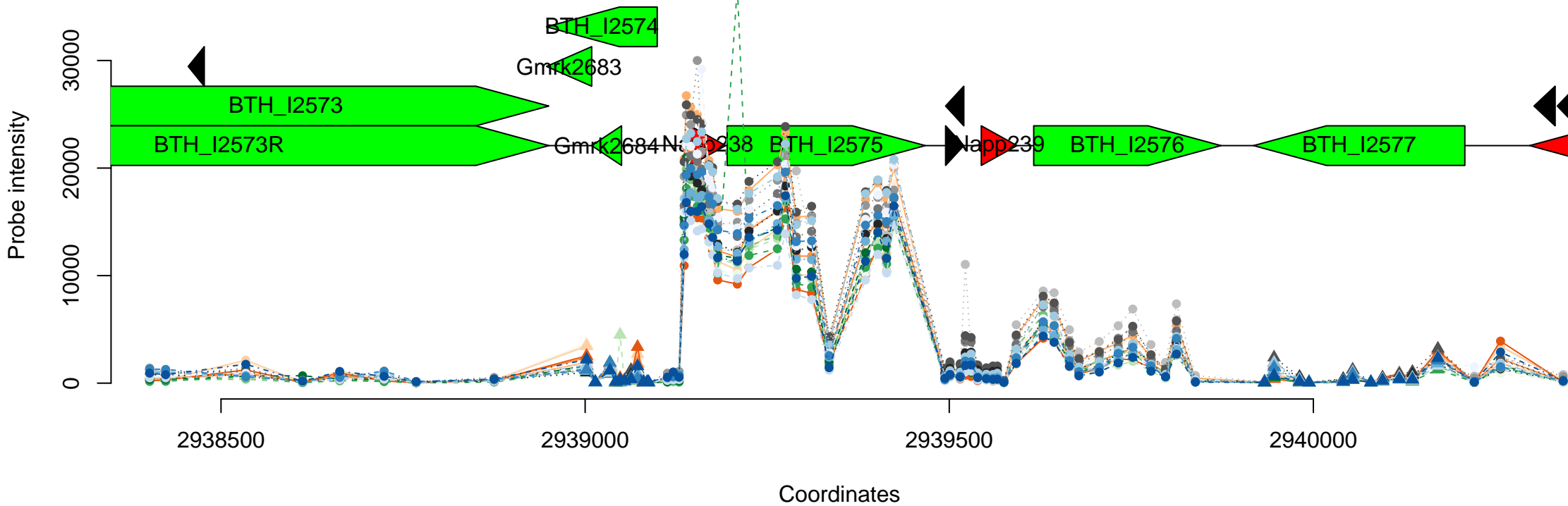

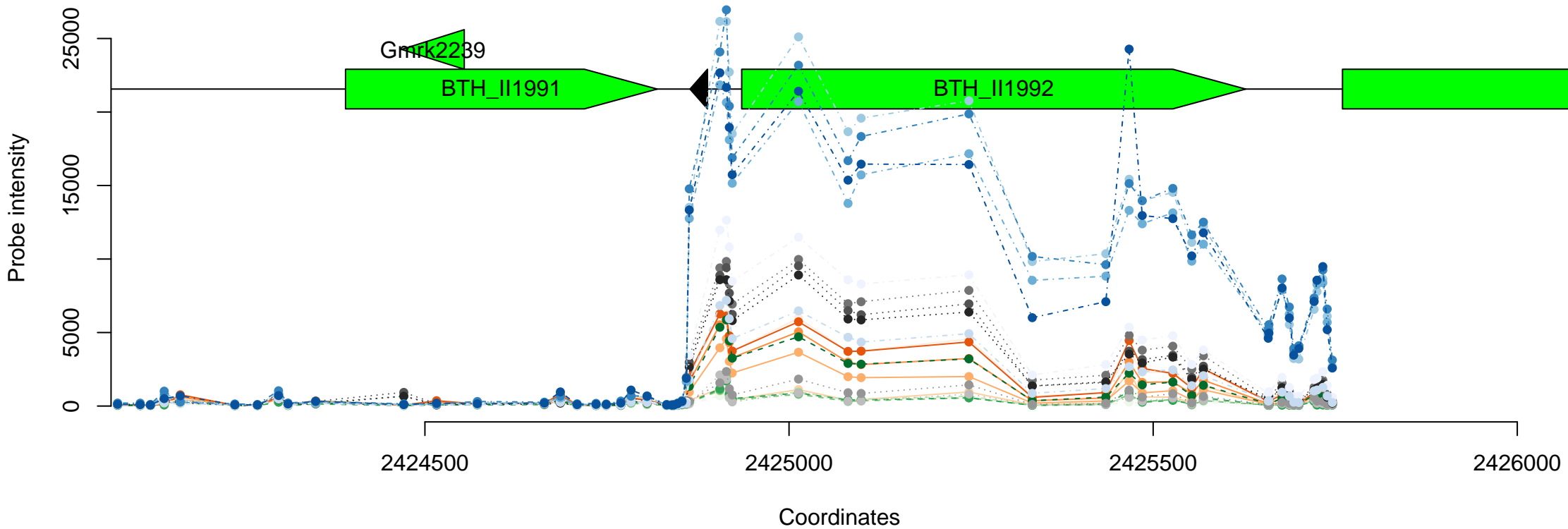

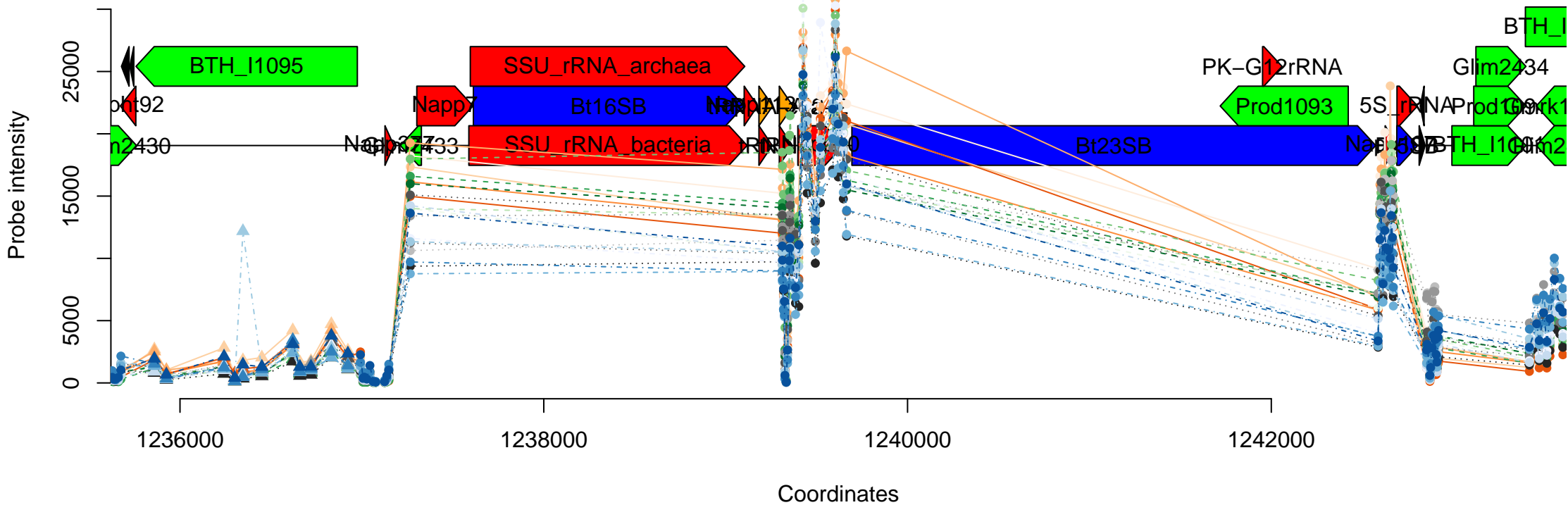

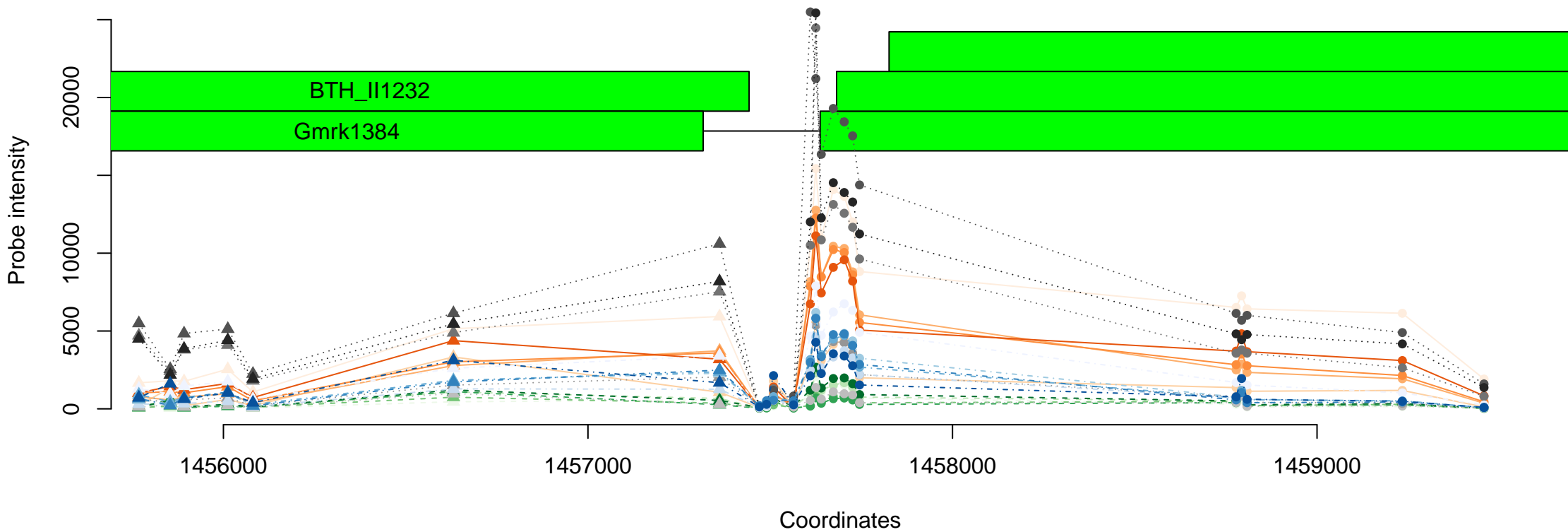

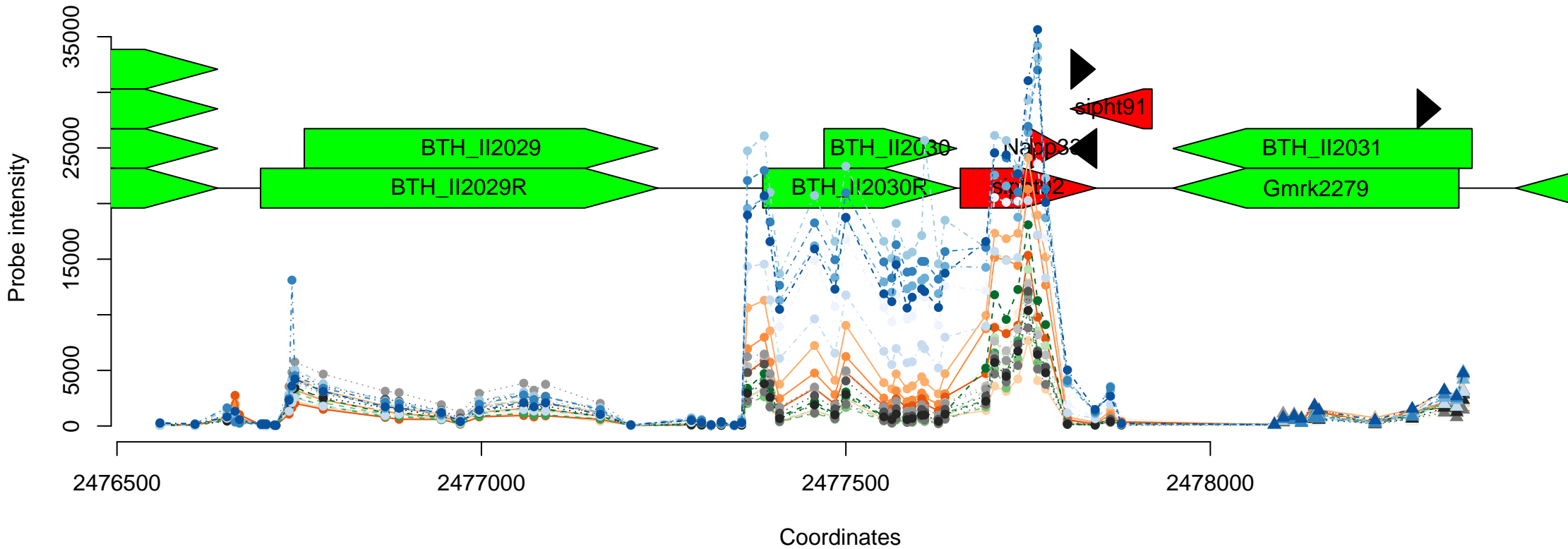

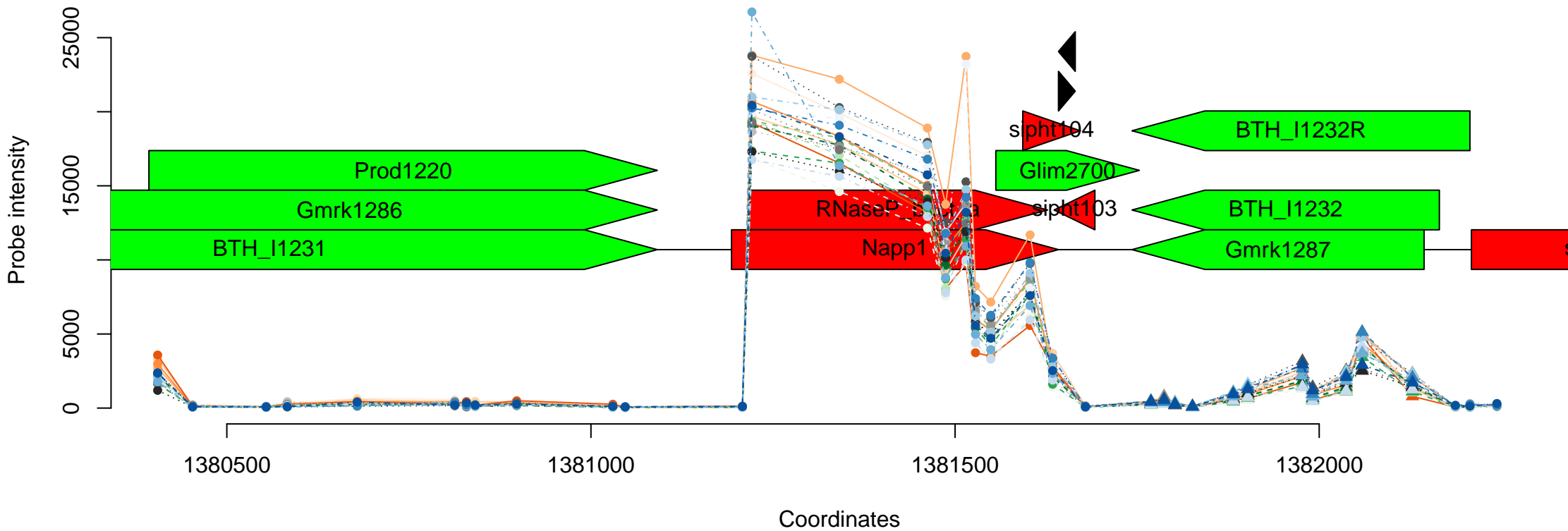

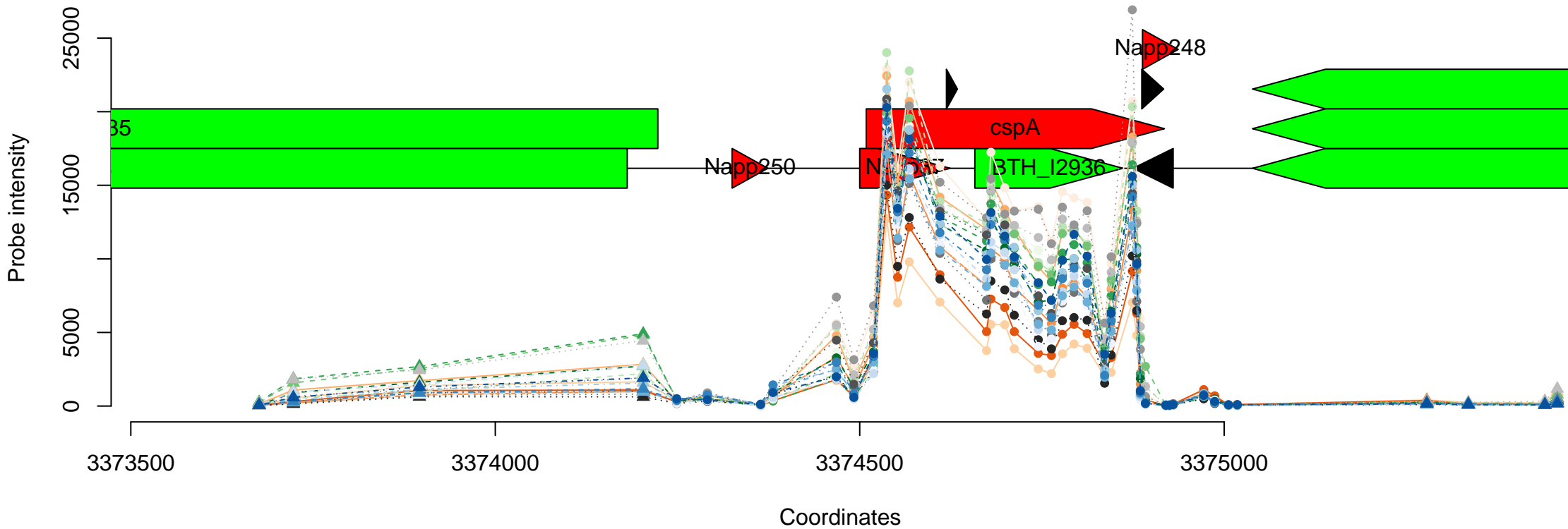

Probe intensity

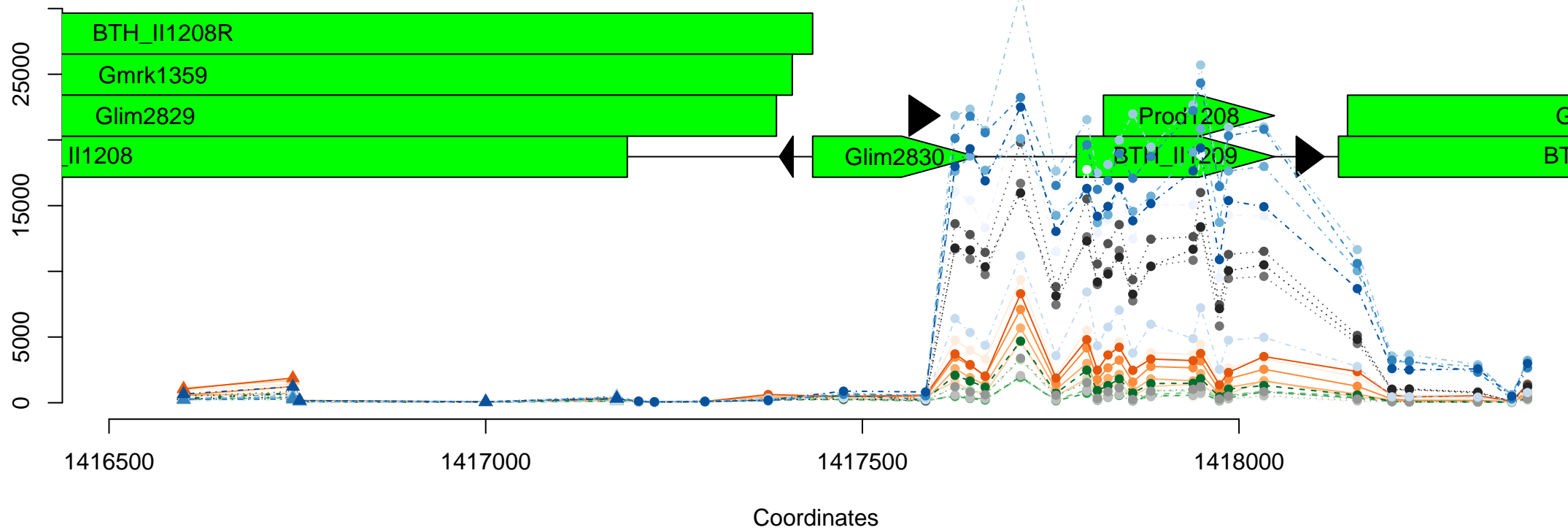

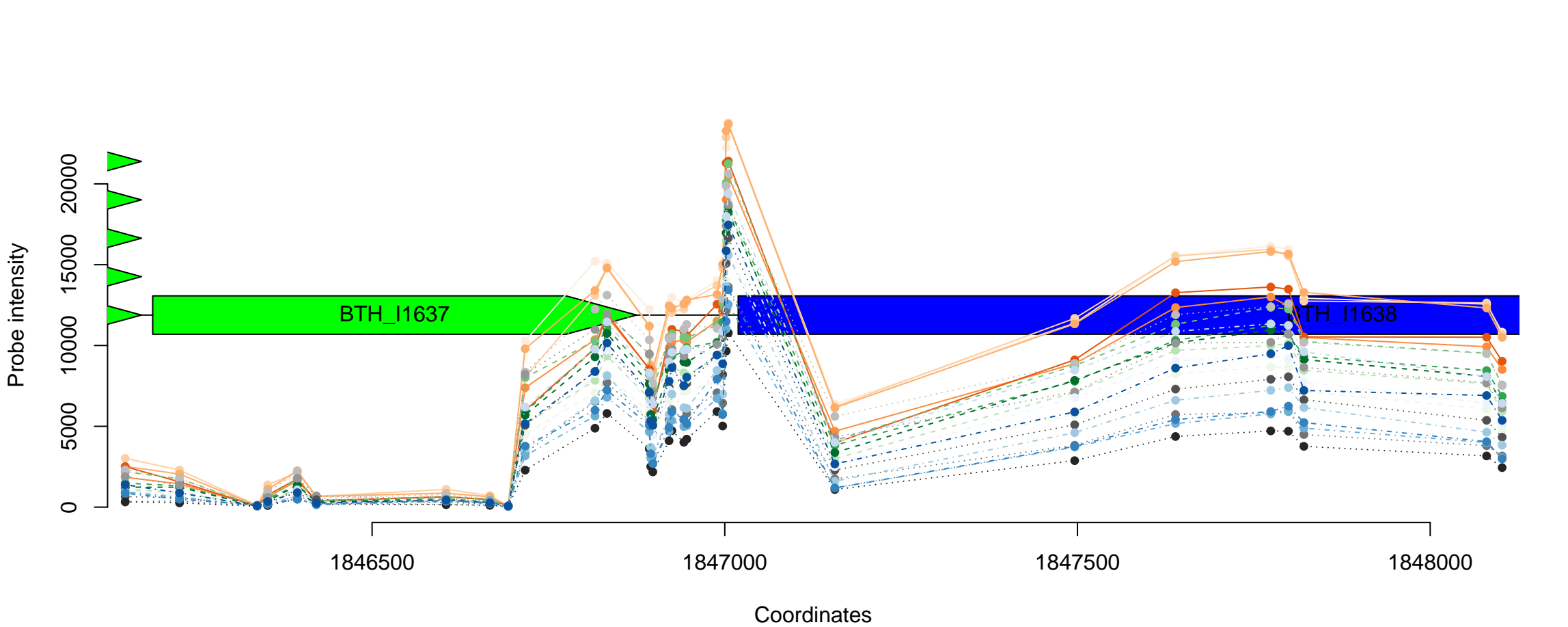

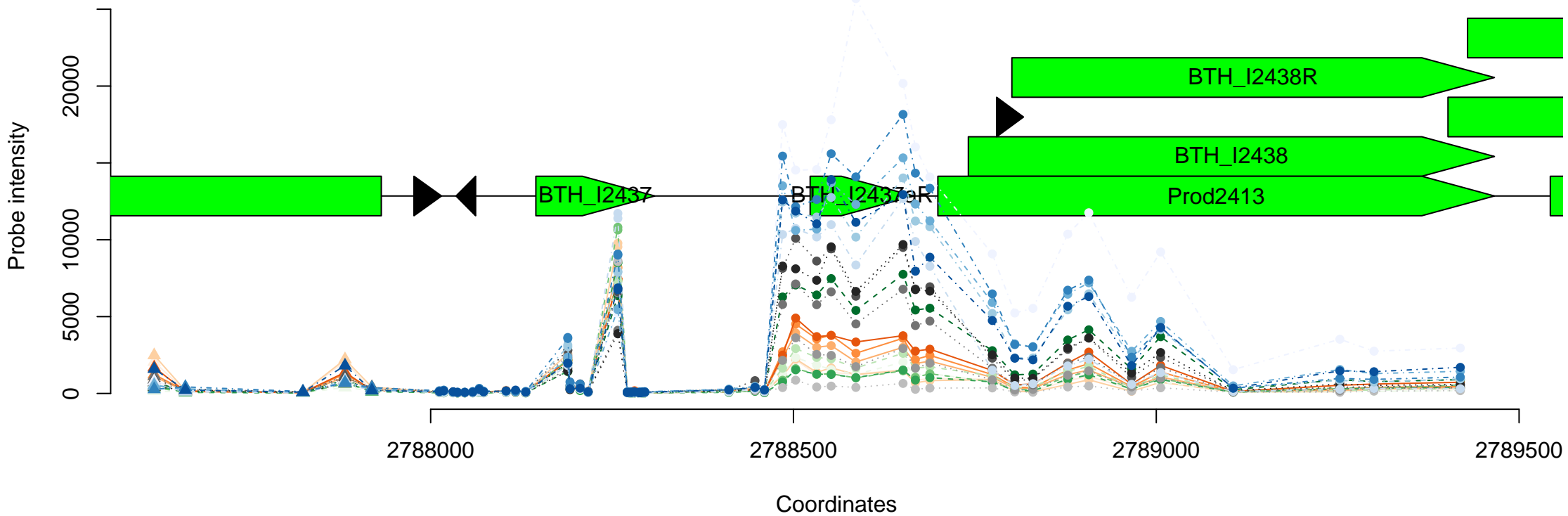

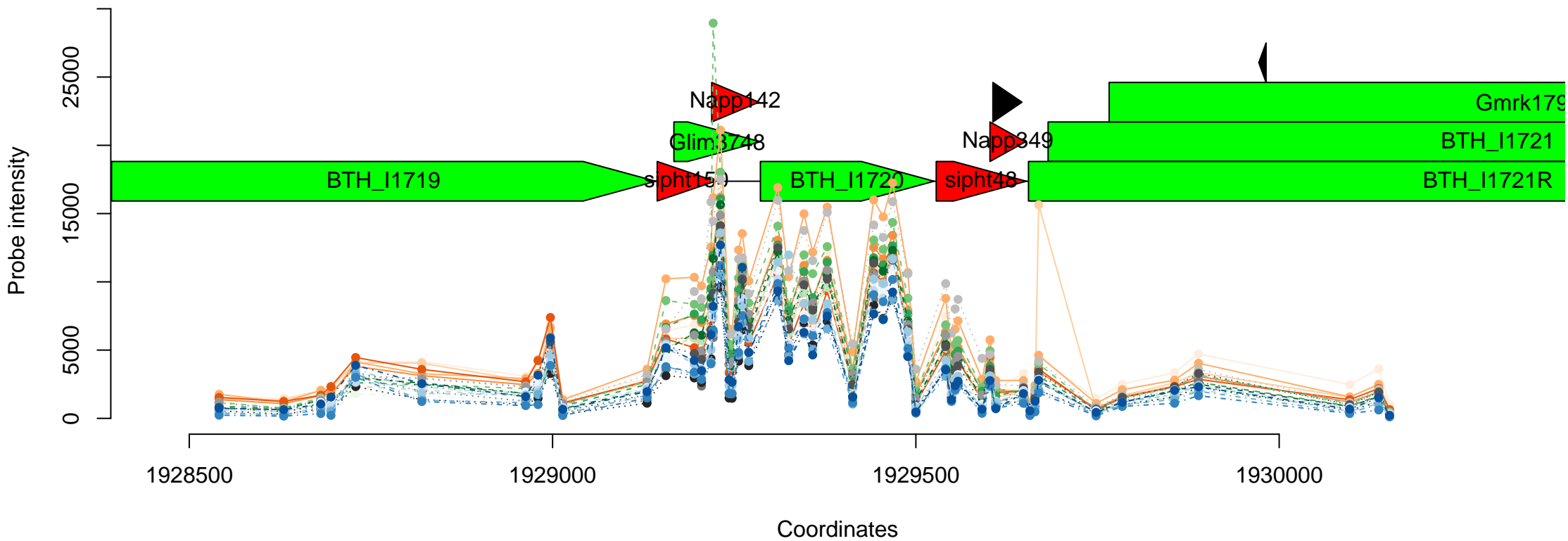

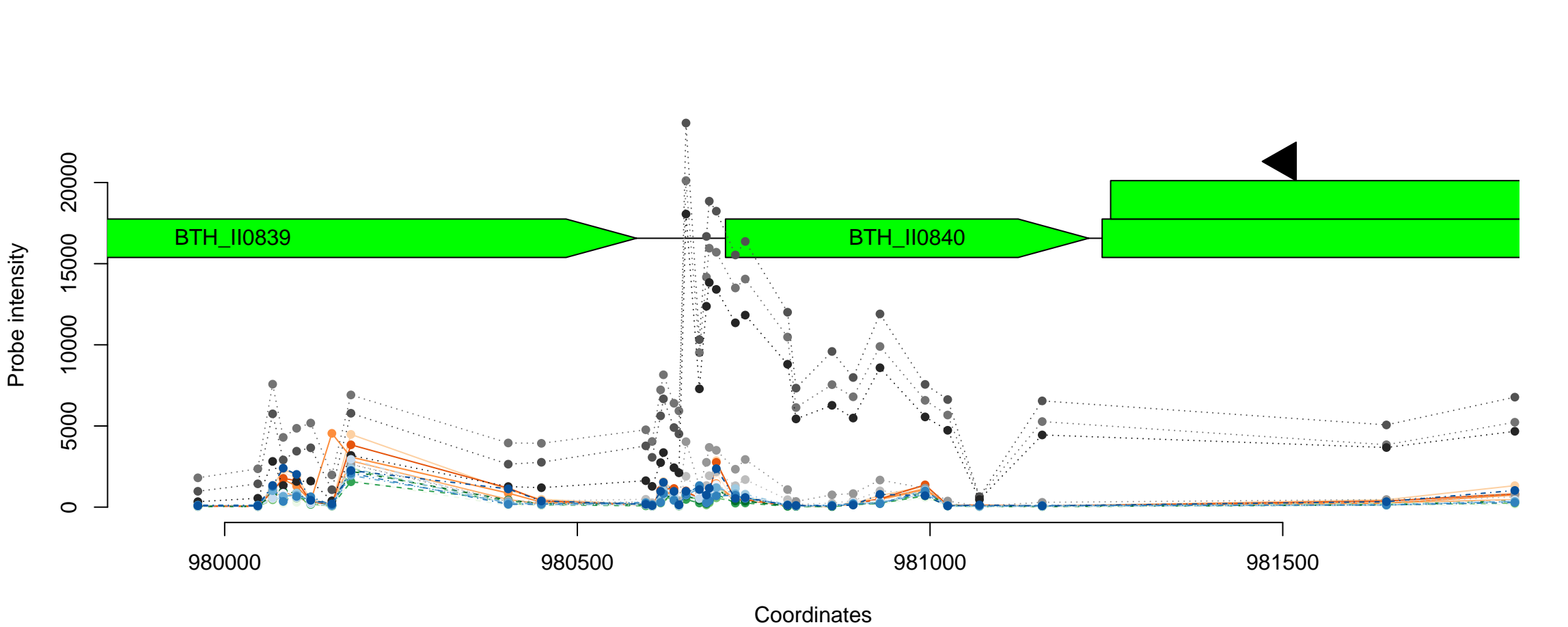

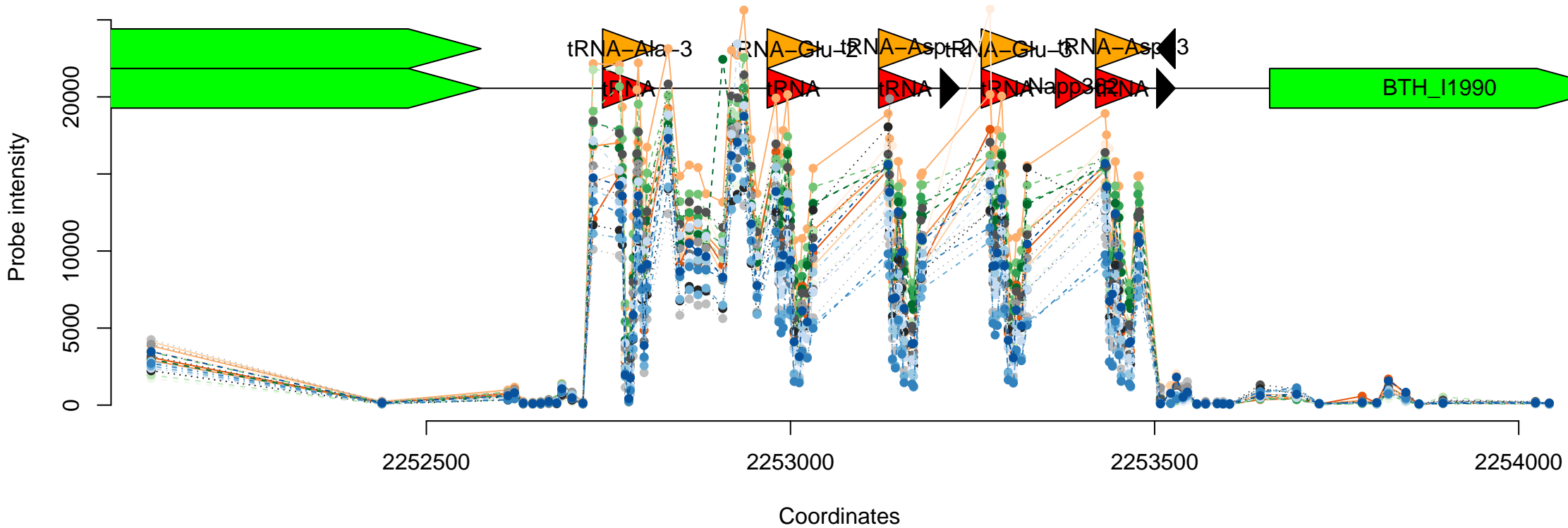

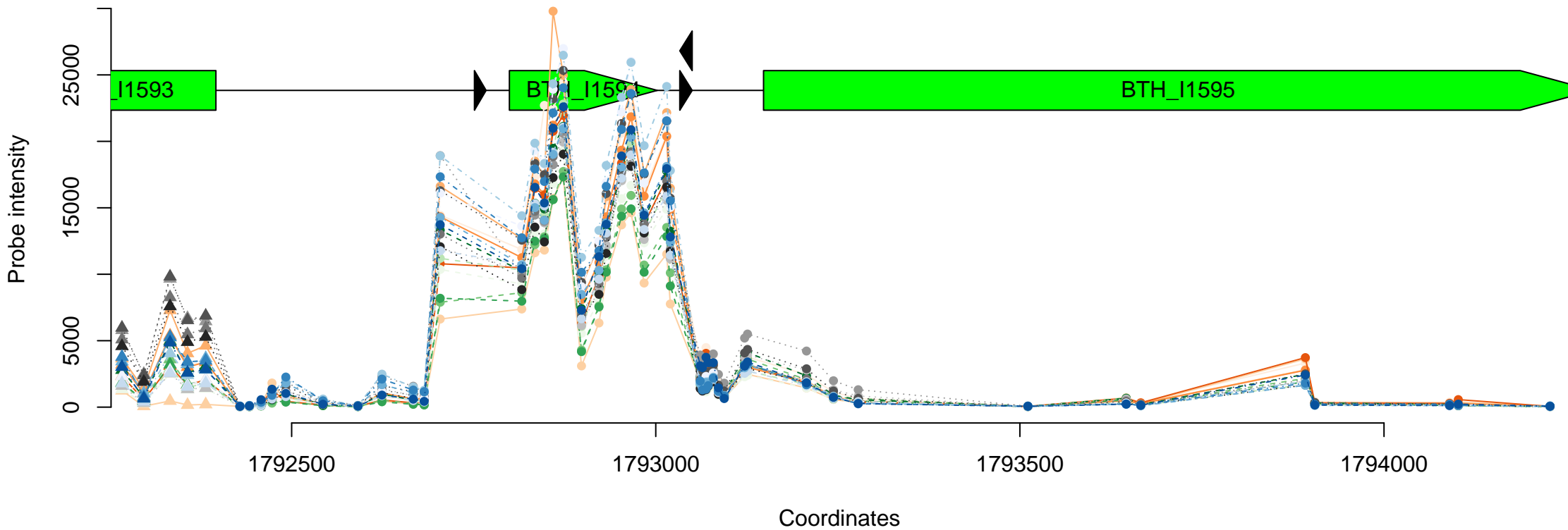

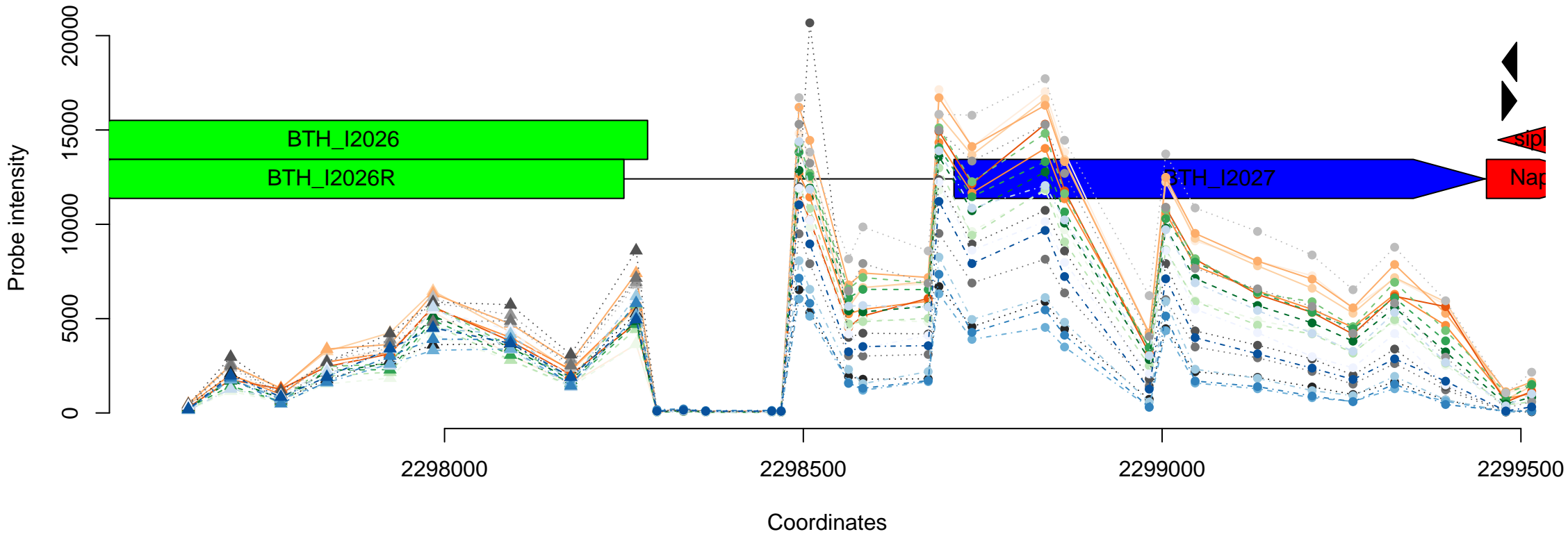

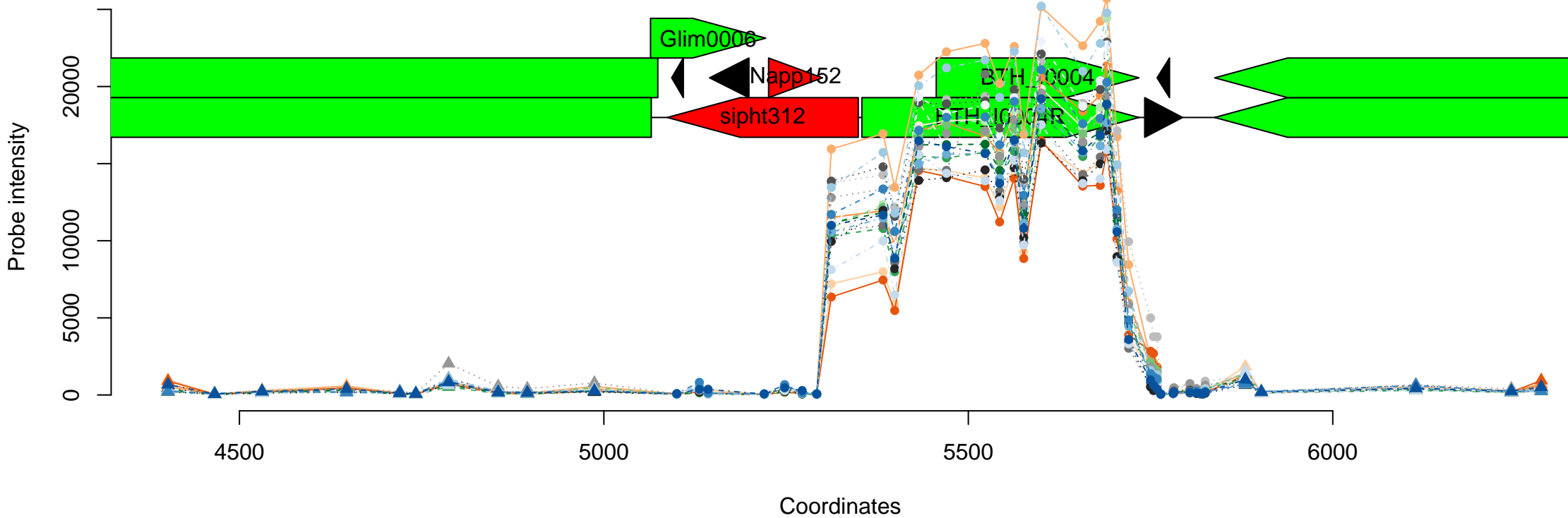

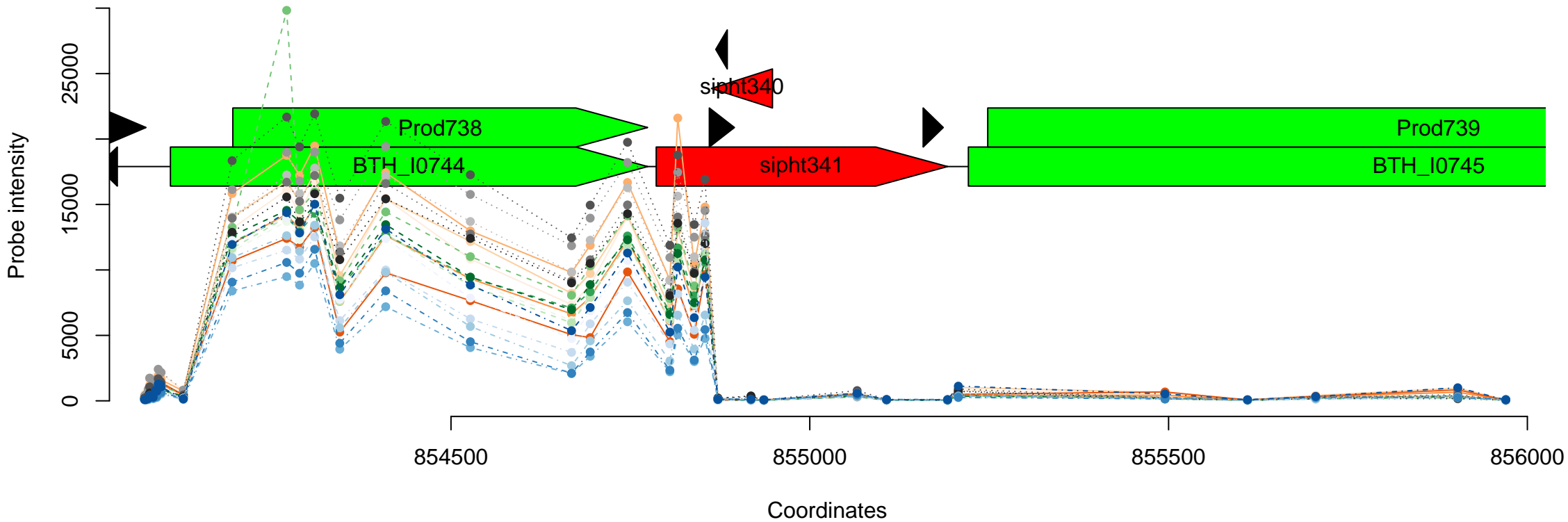

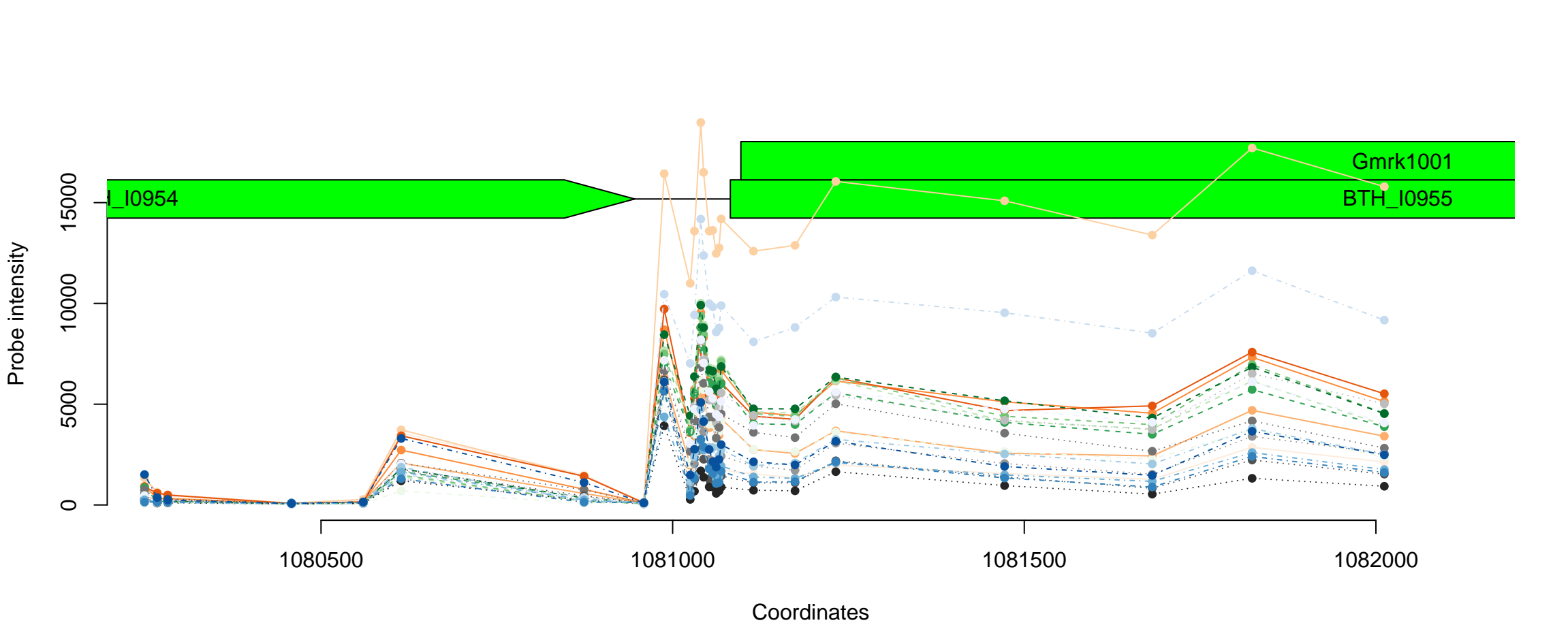

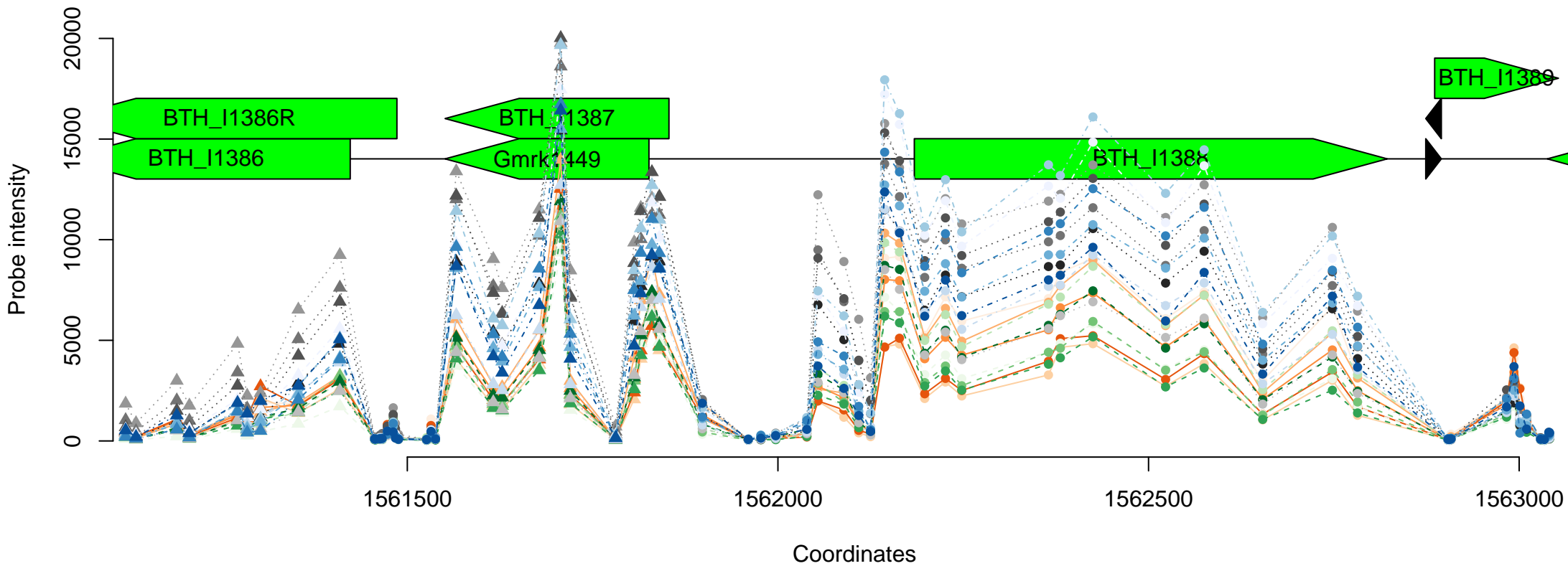

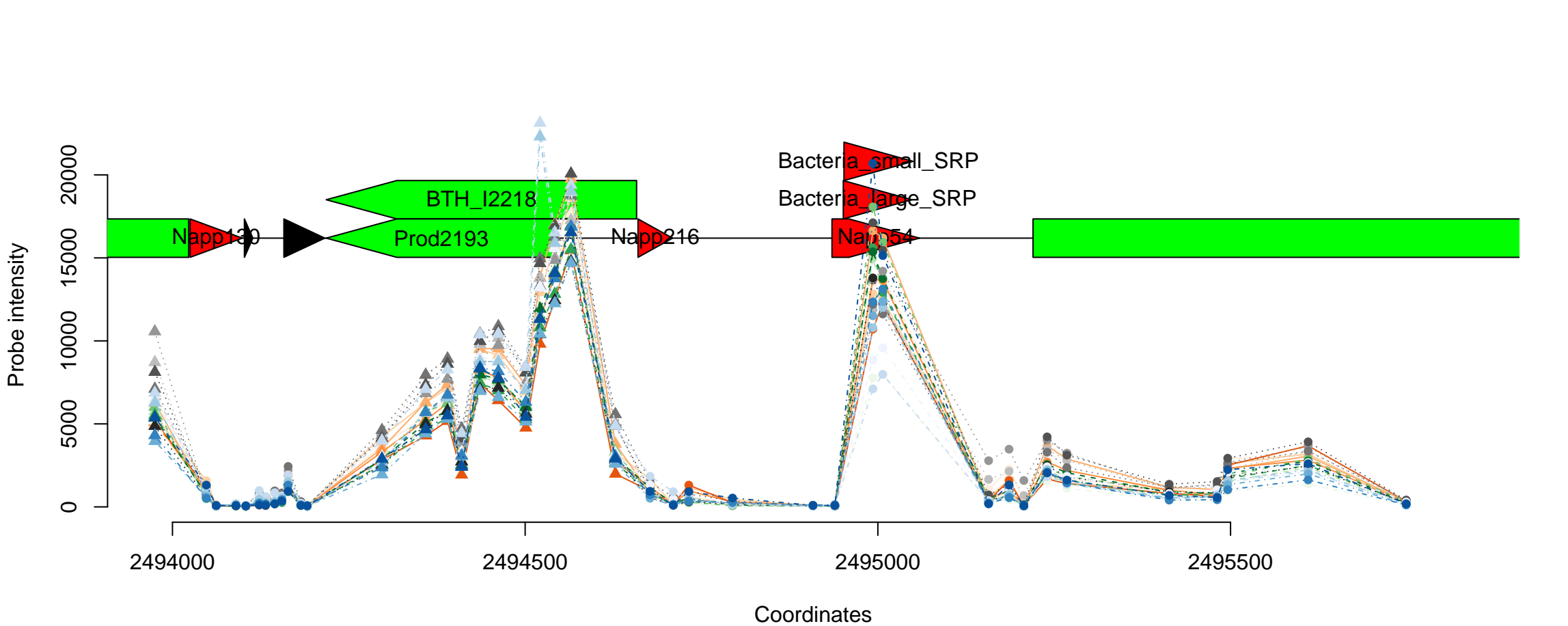

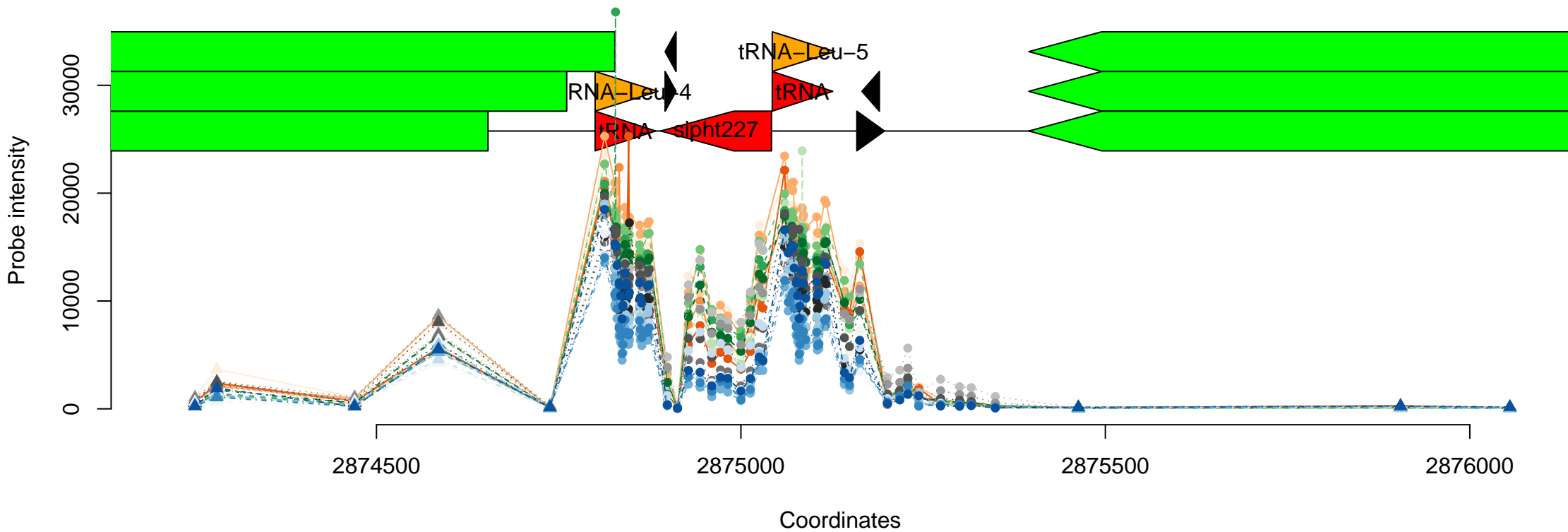

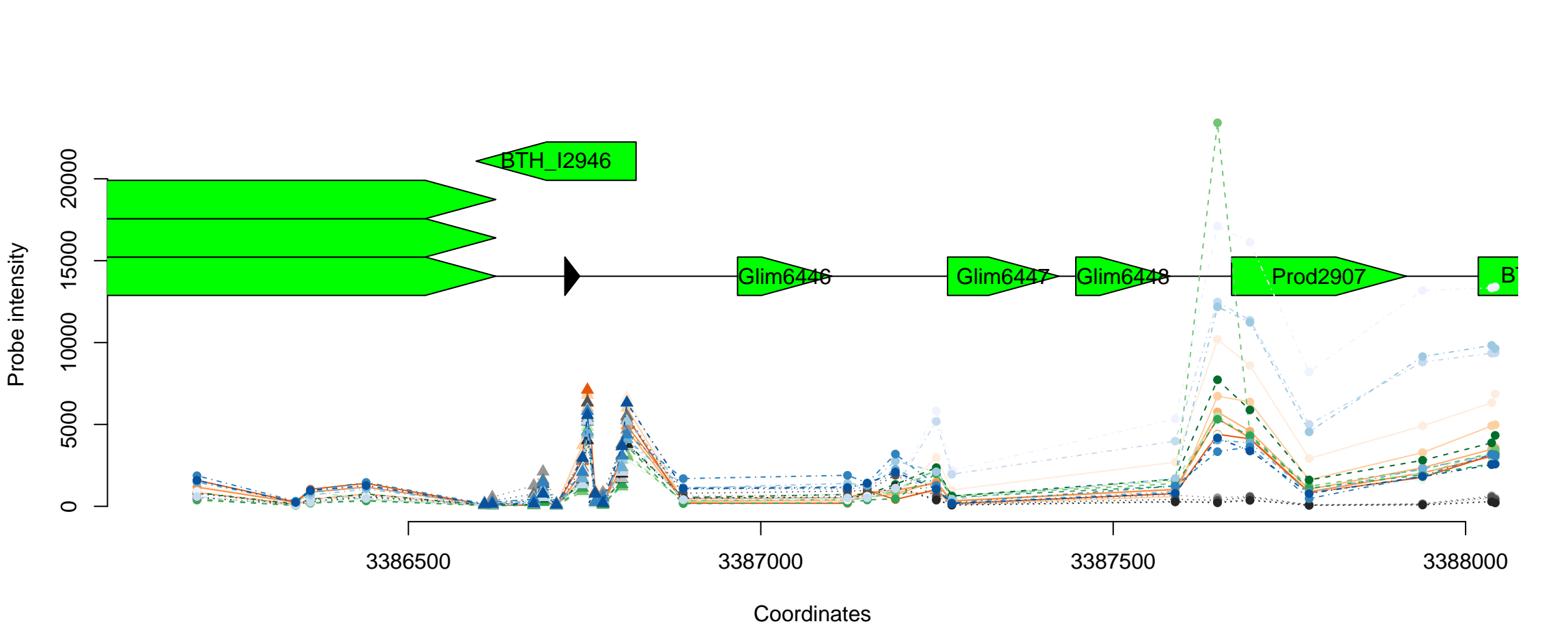

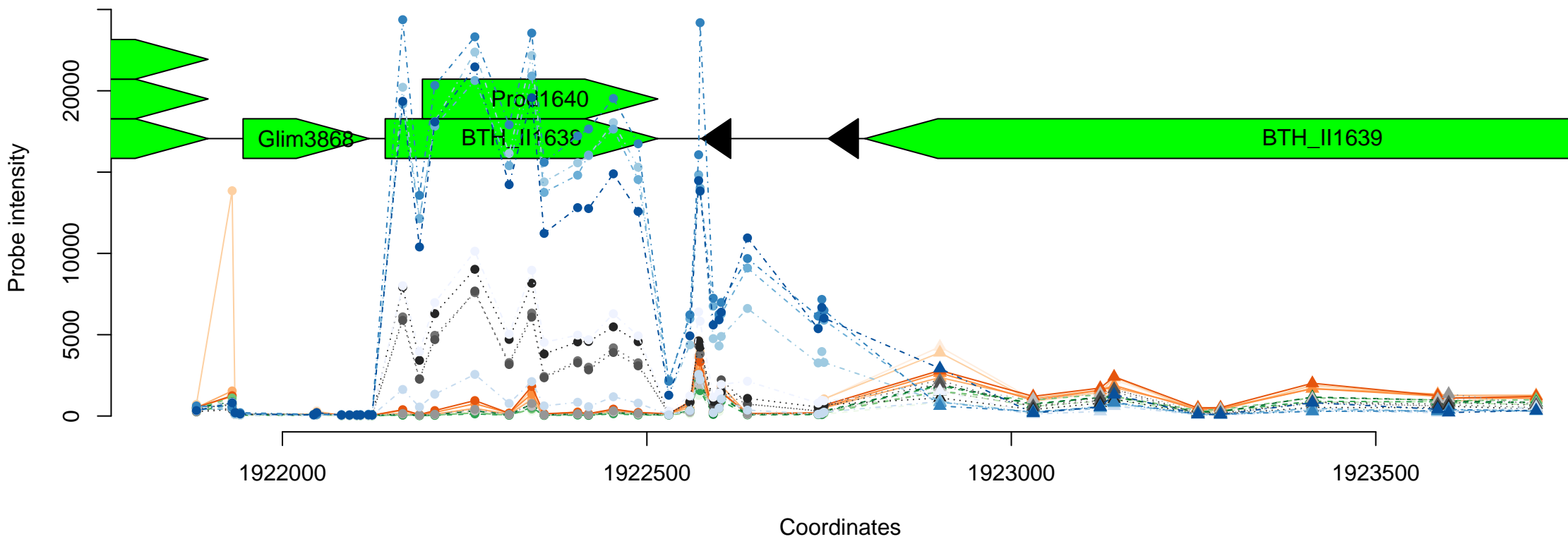

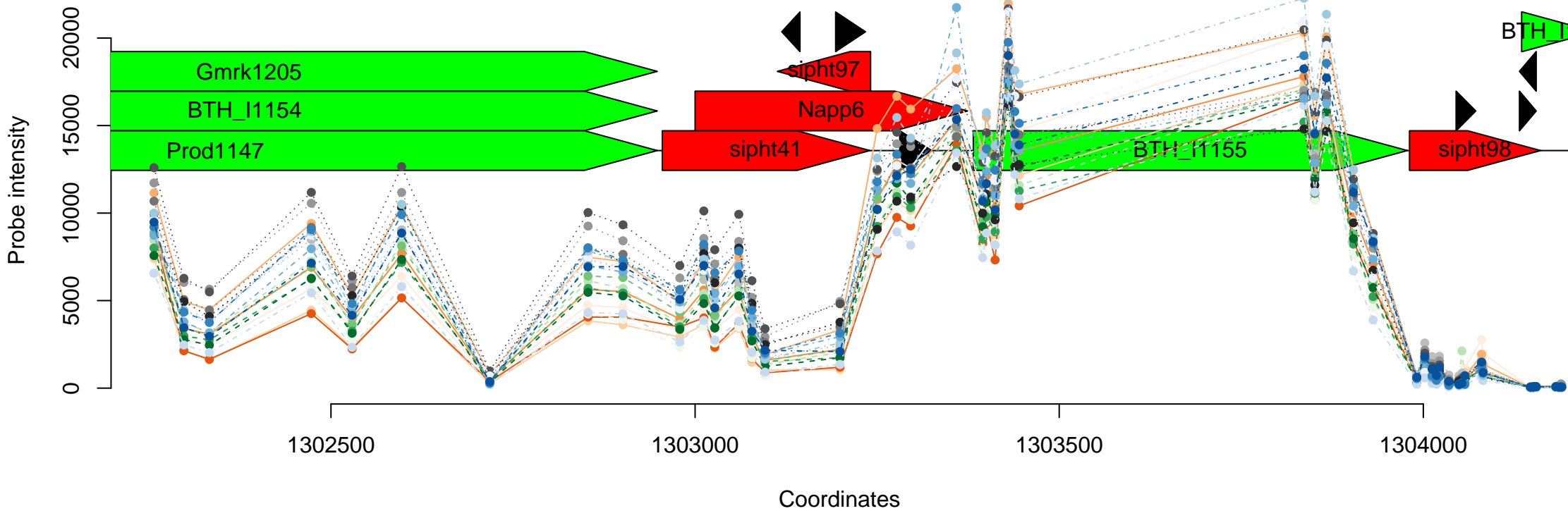

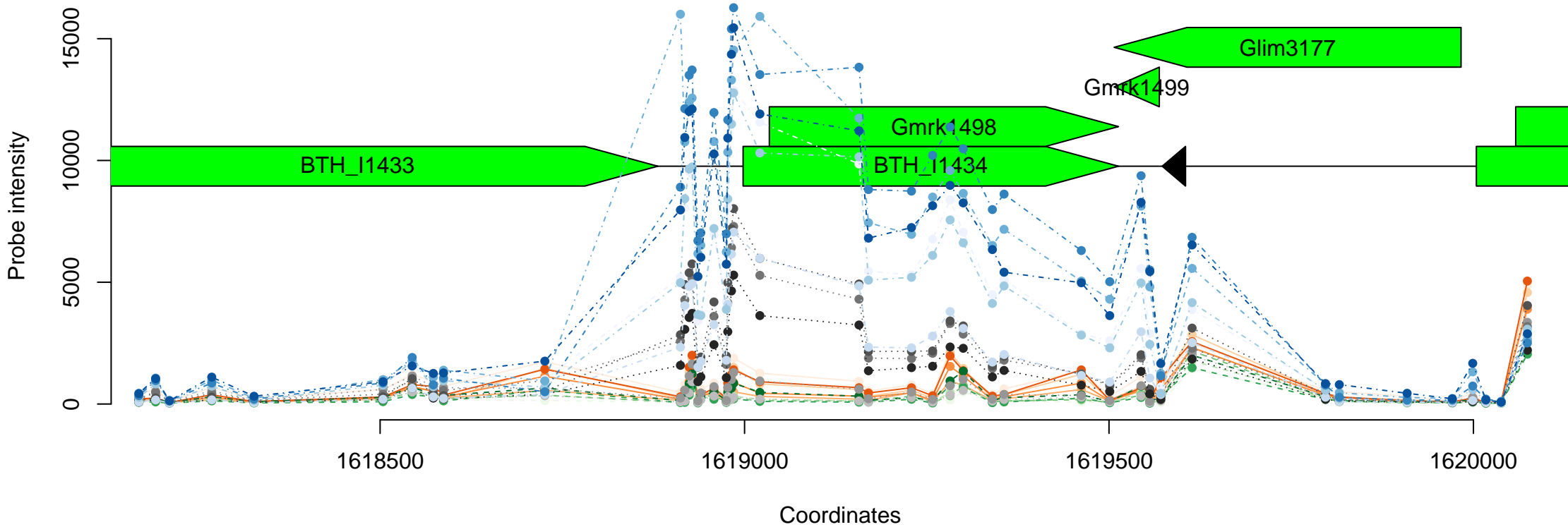

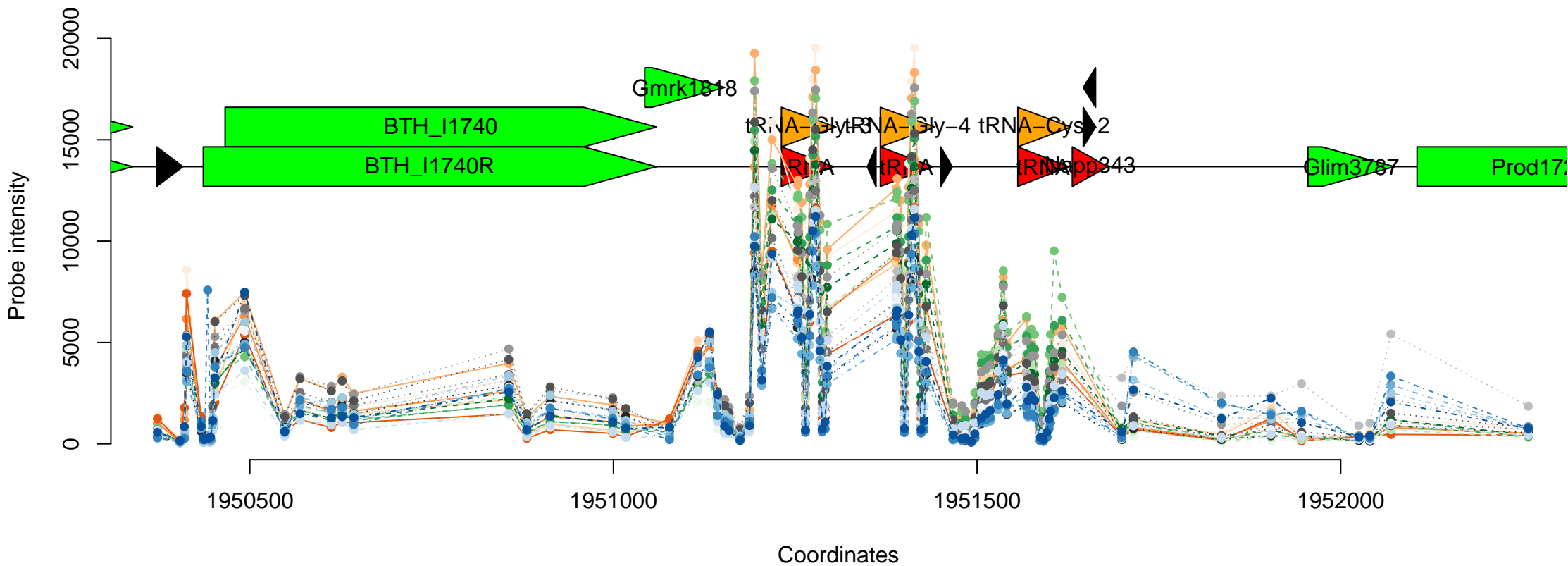

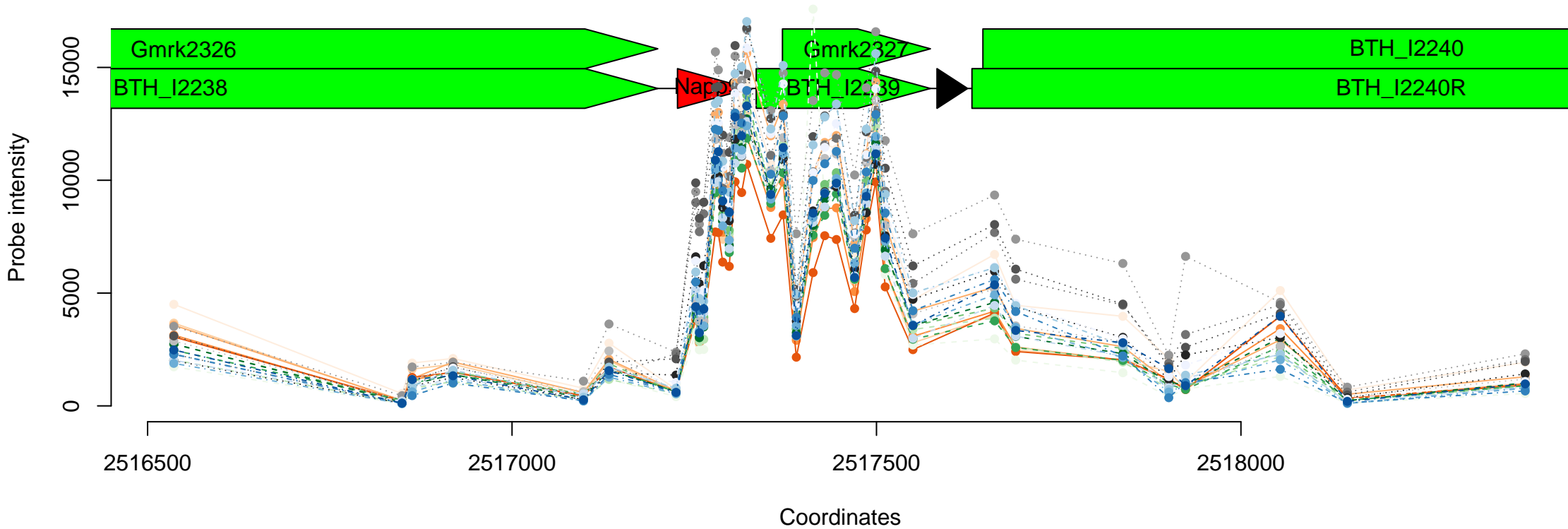

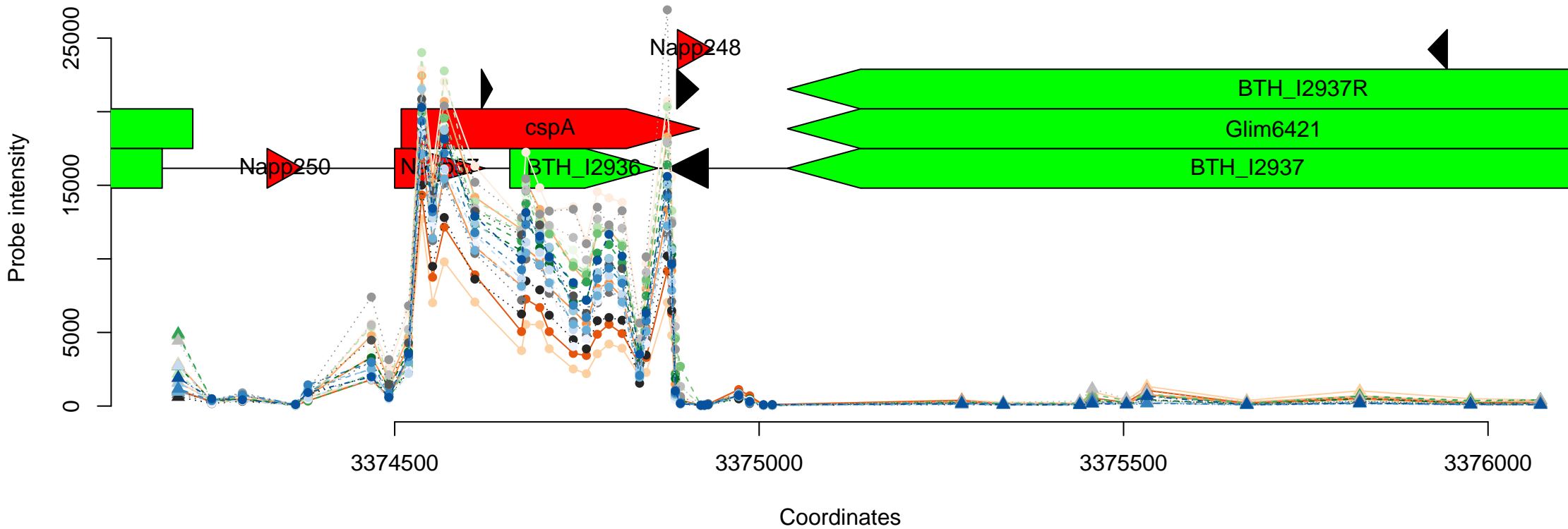

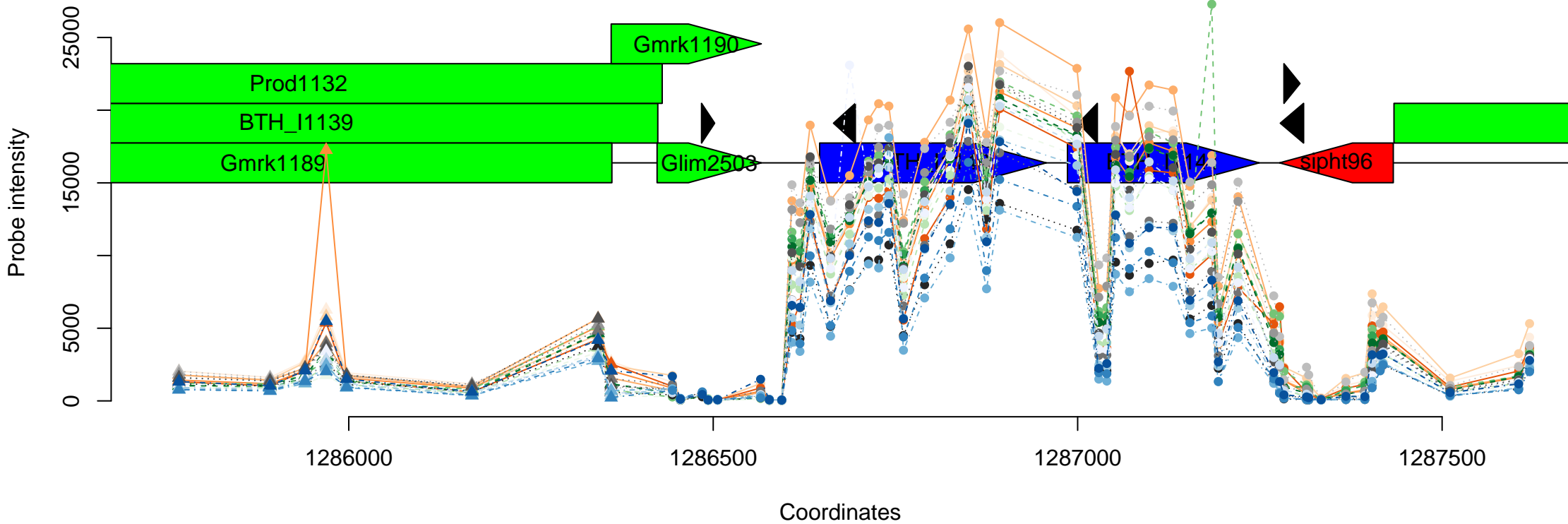

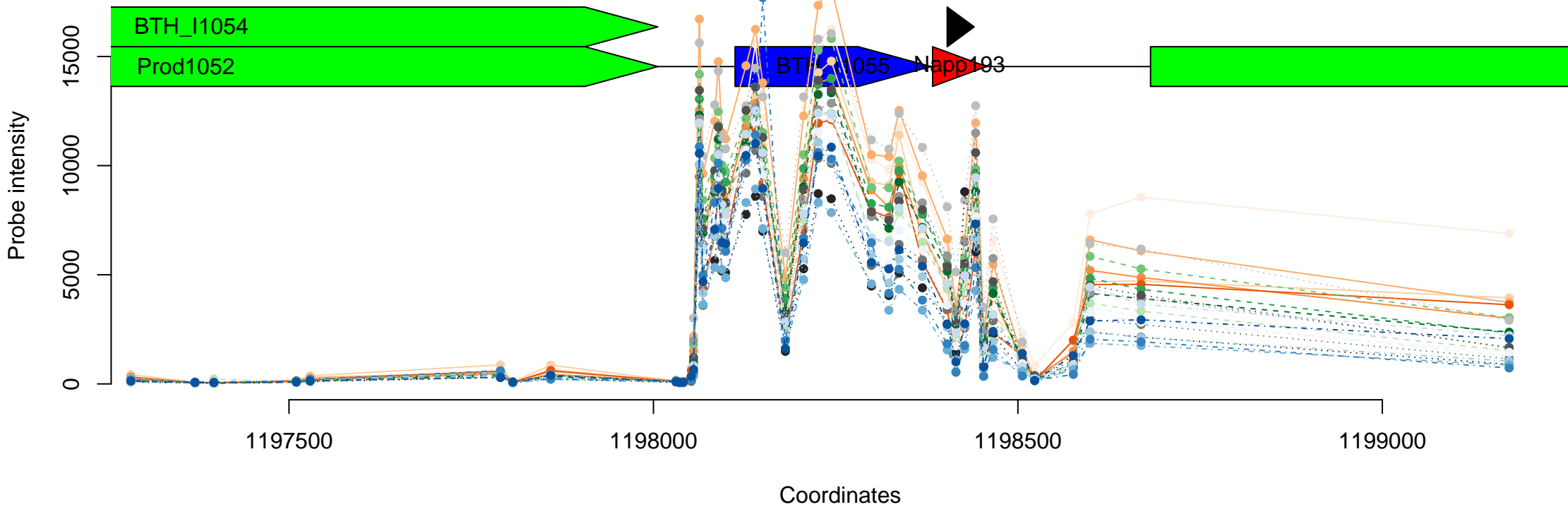

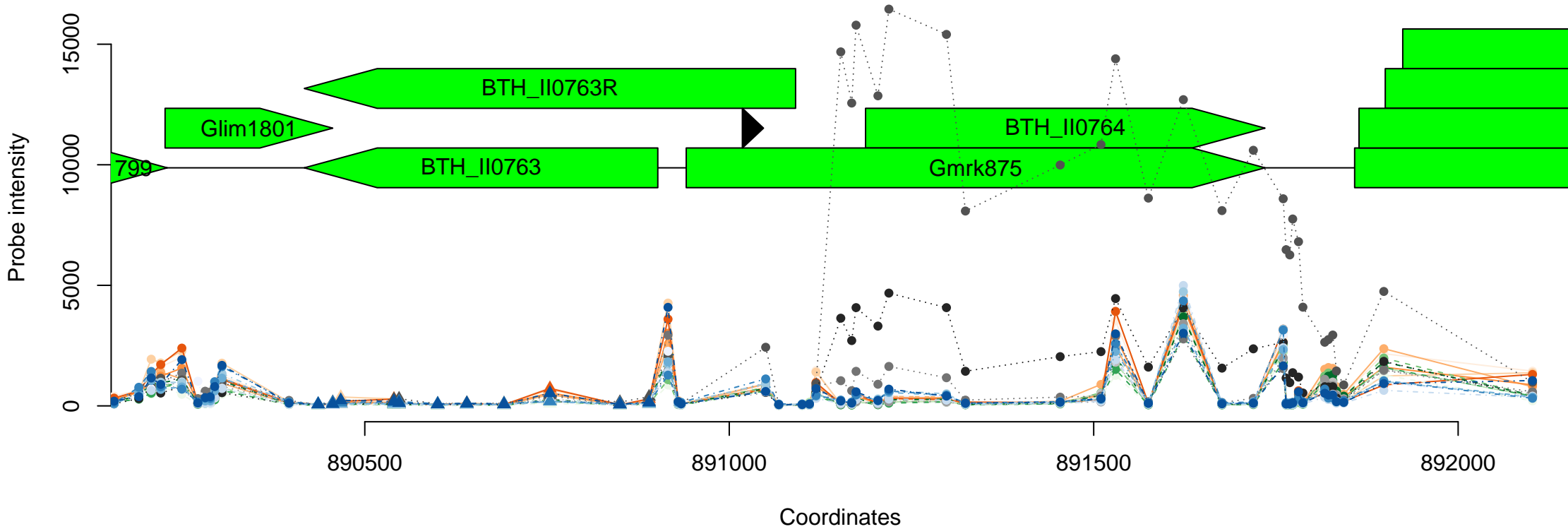

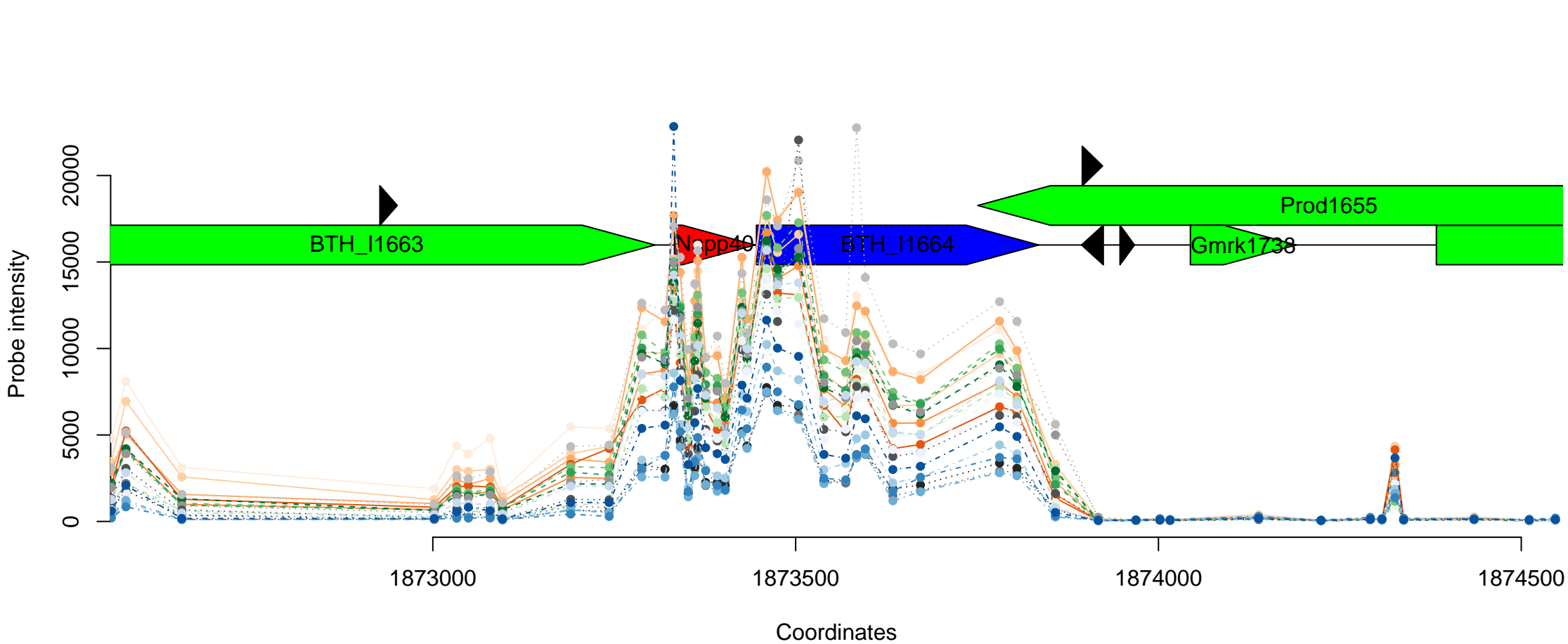

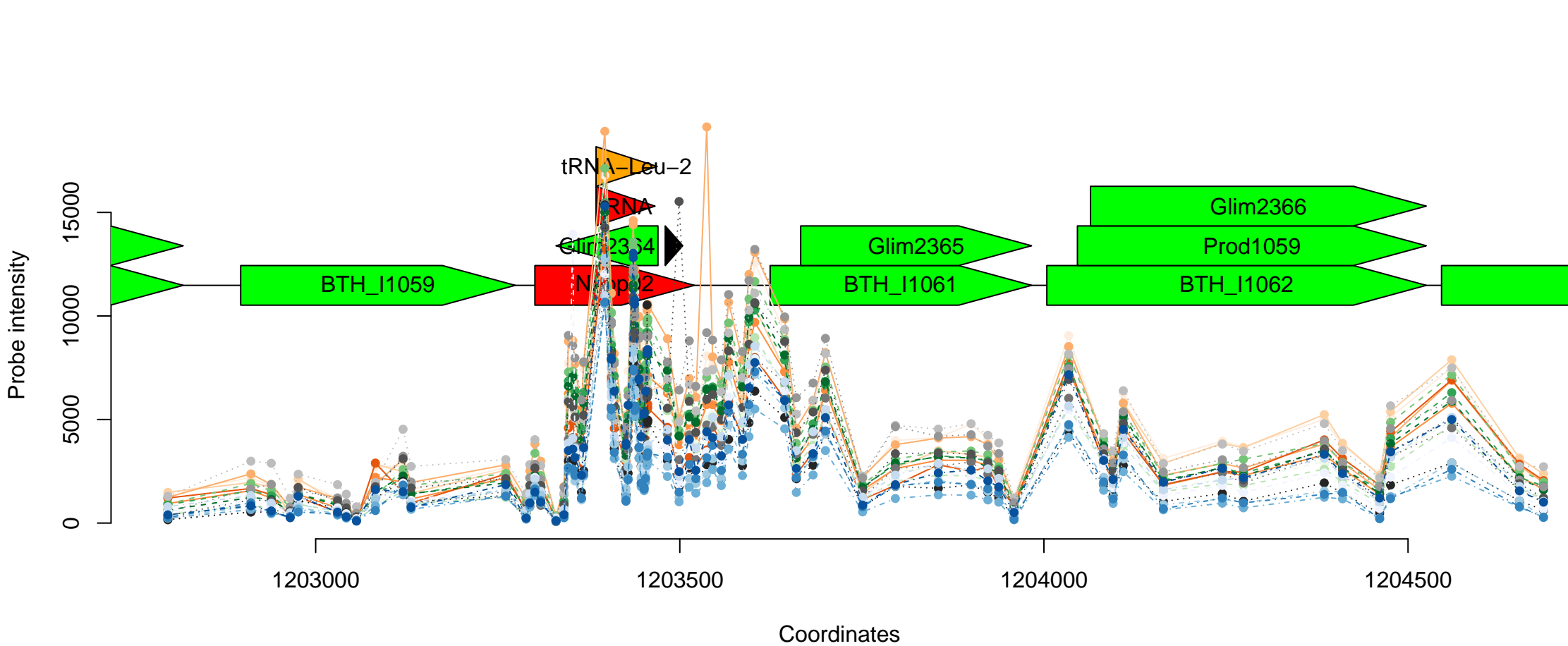

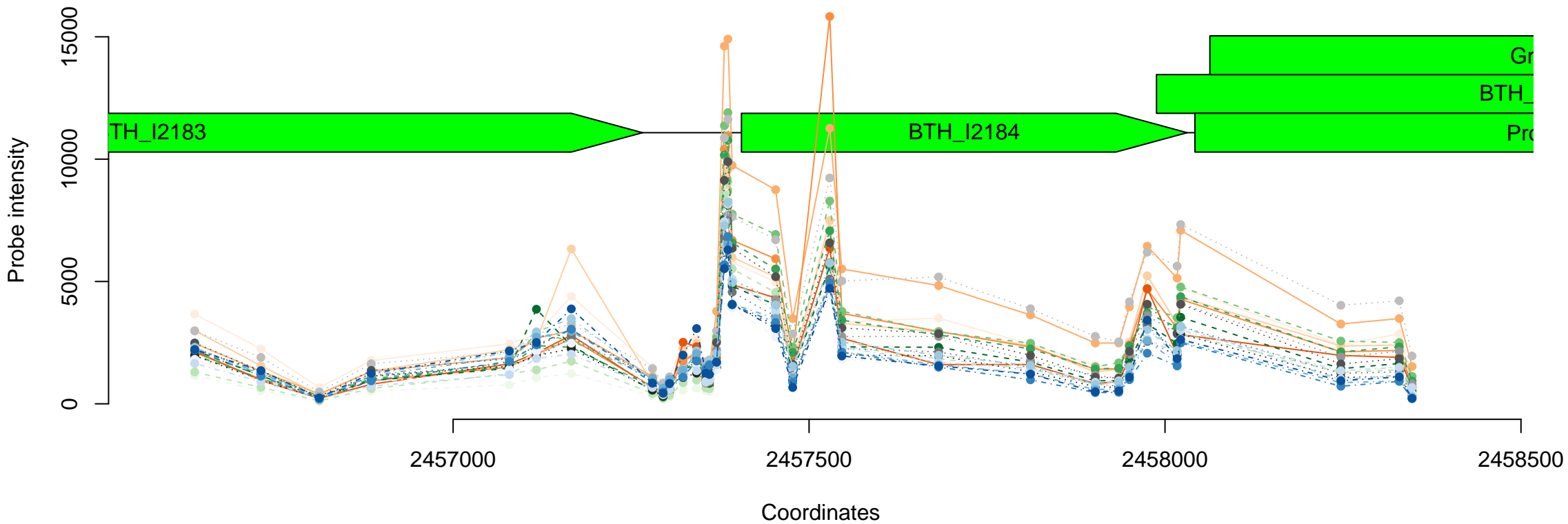

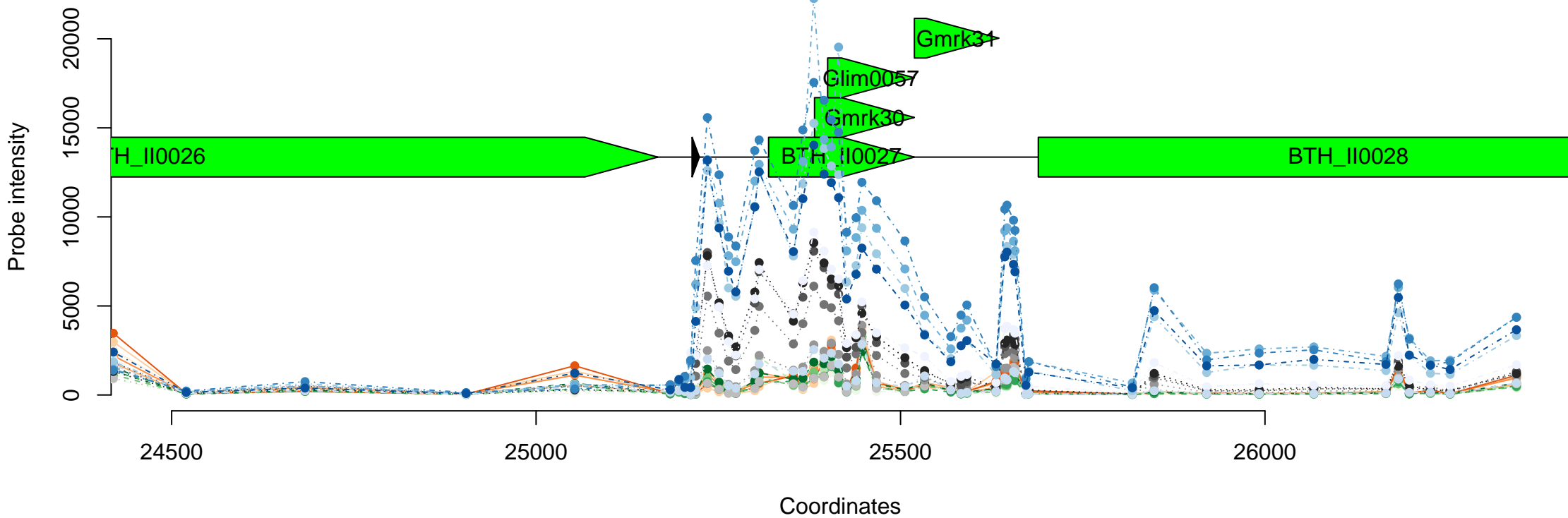

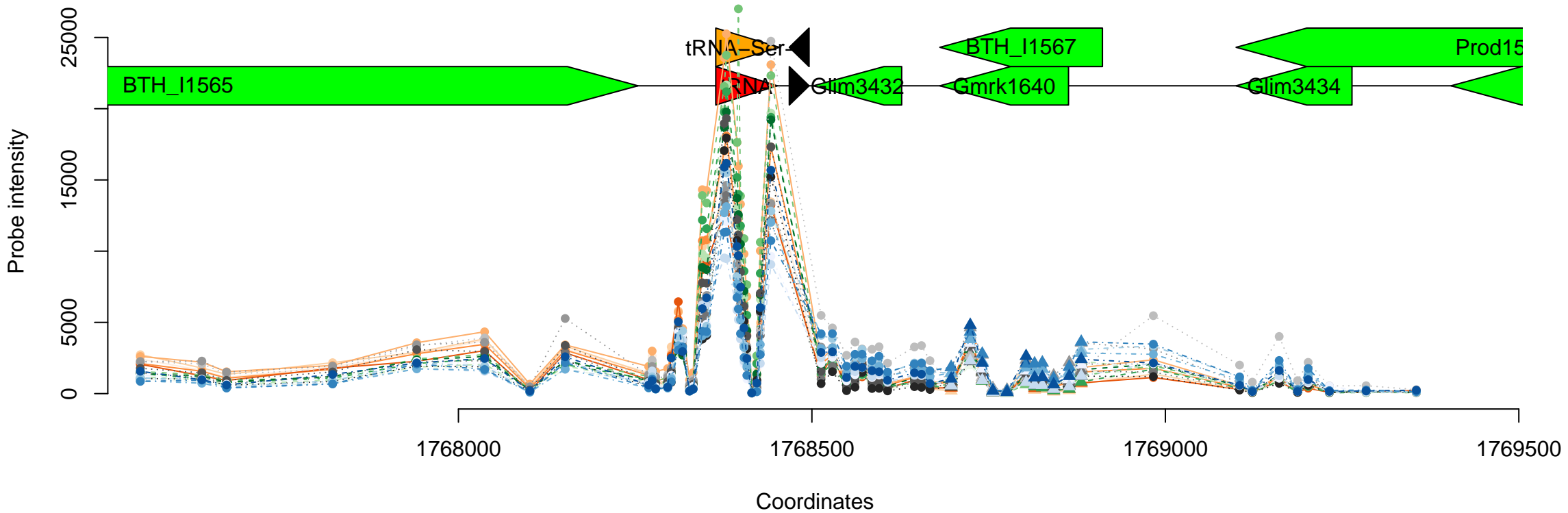

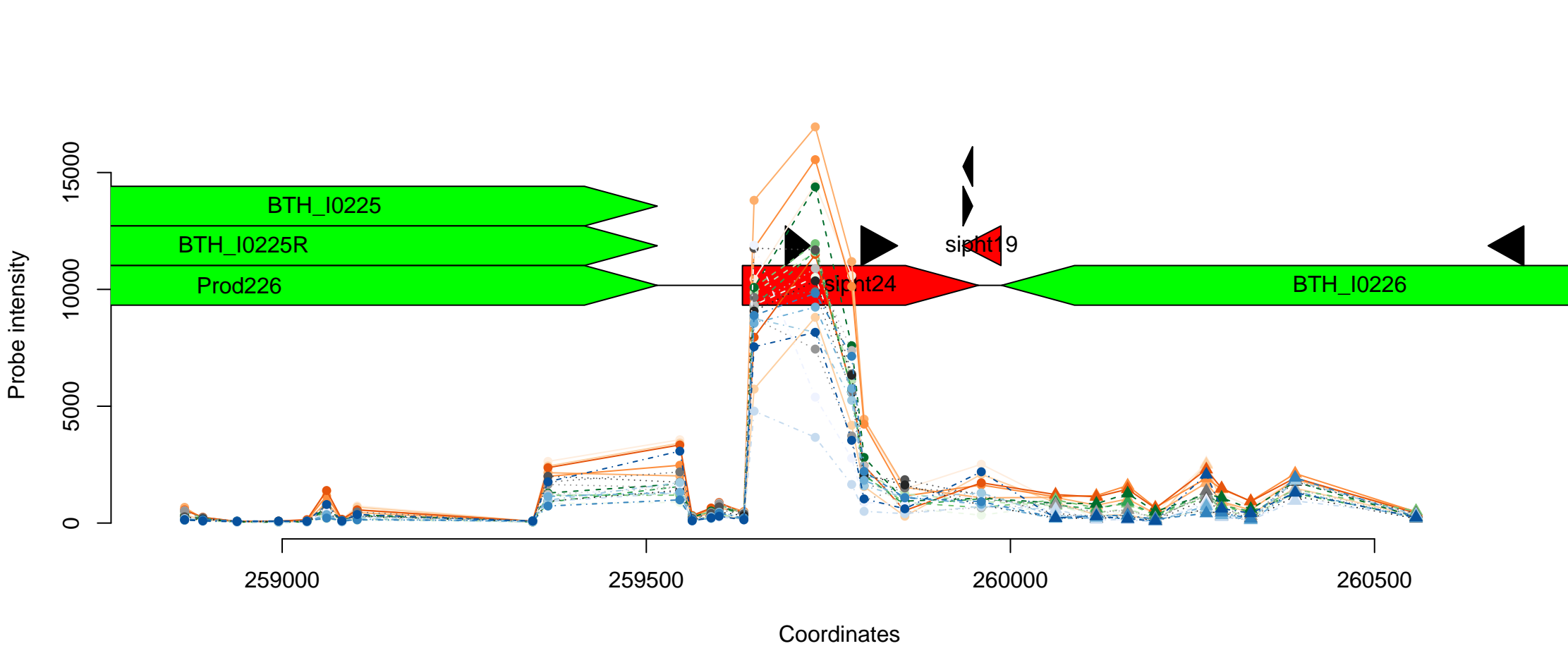

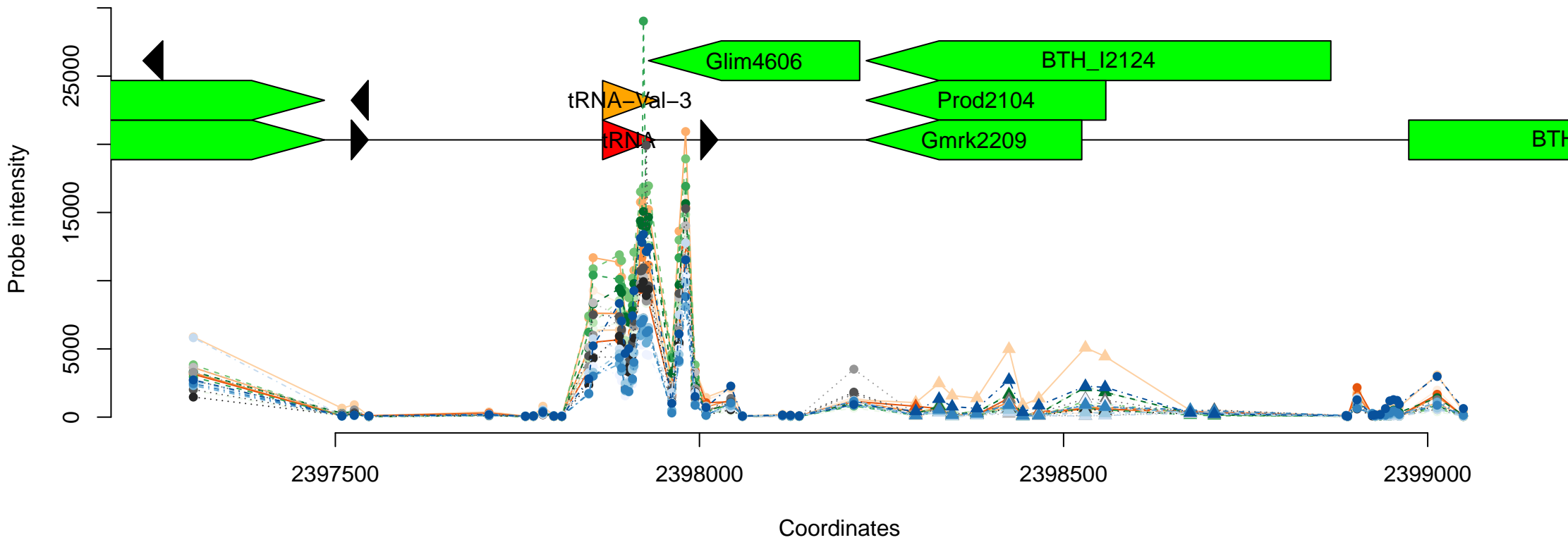

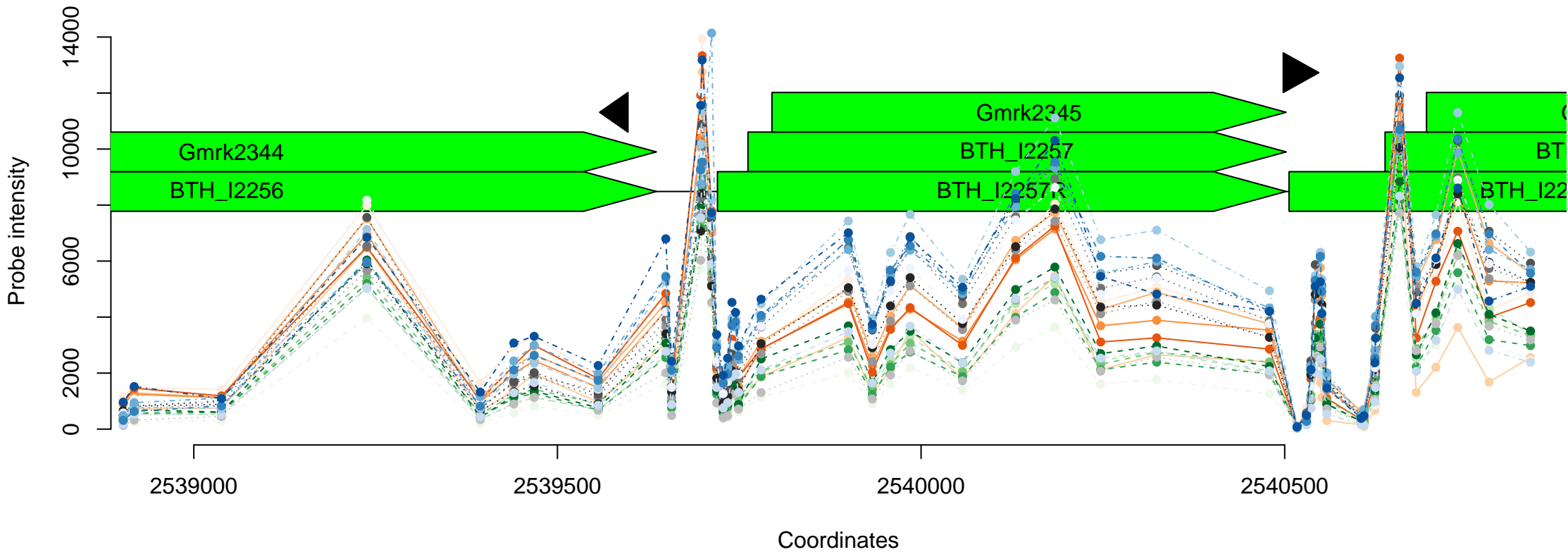

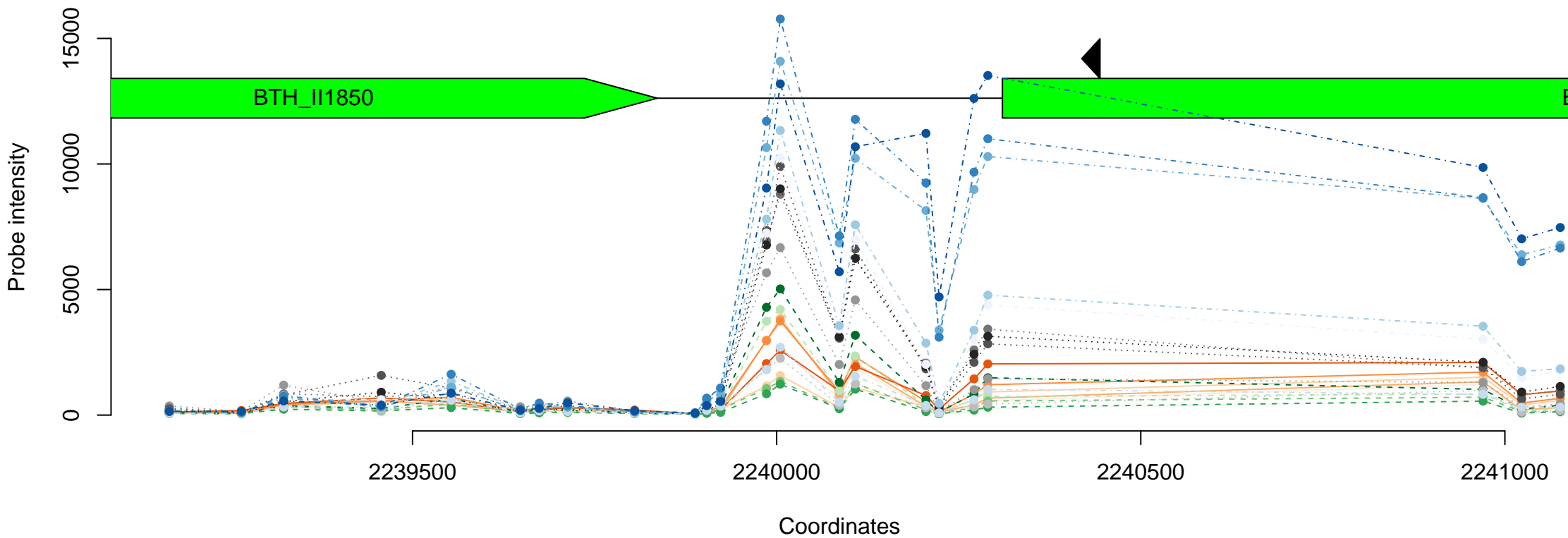

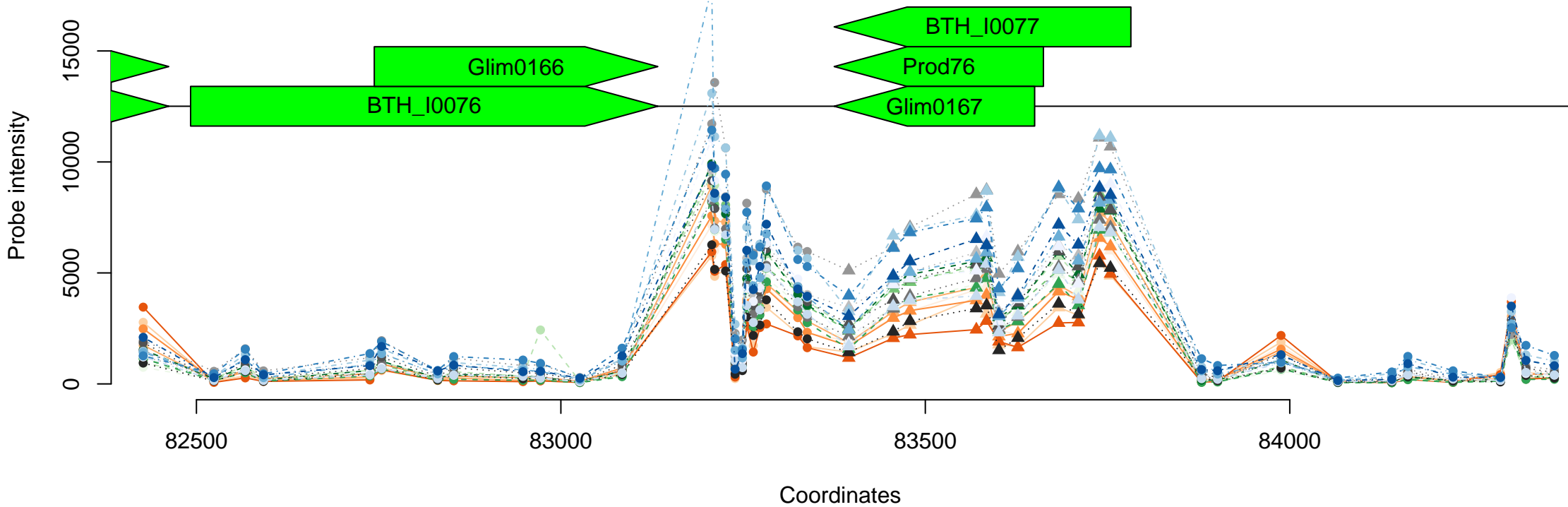

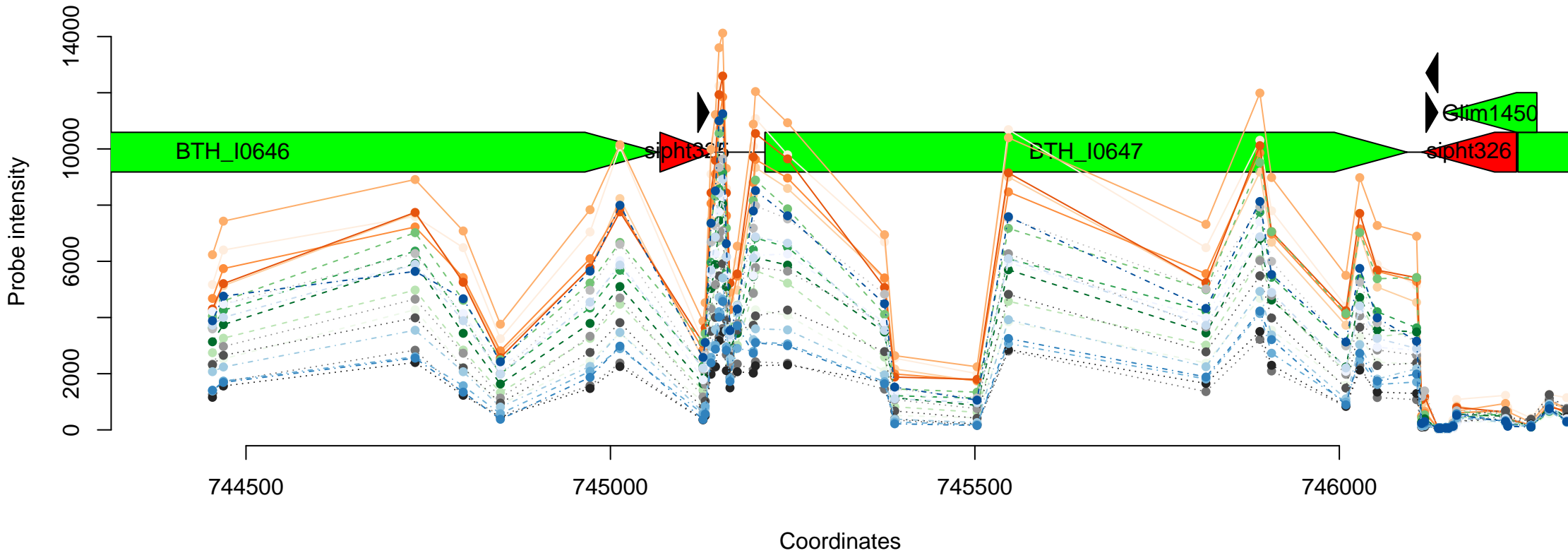

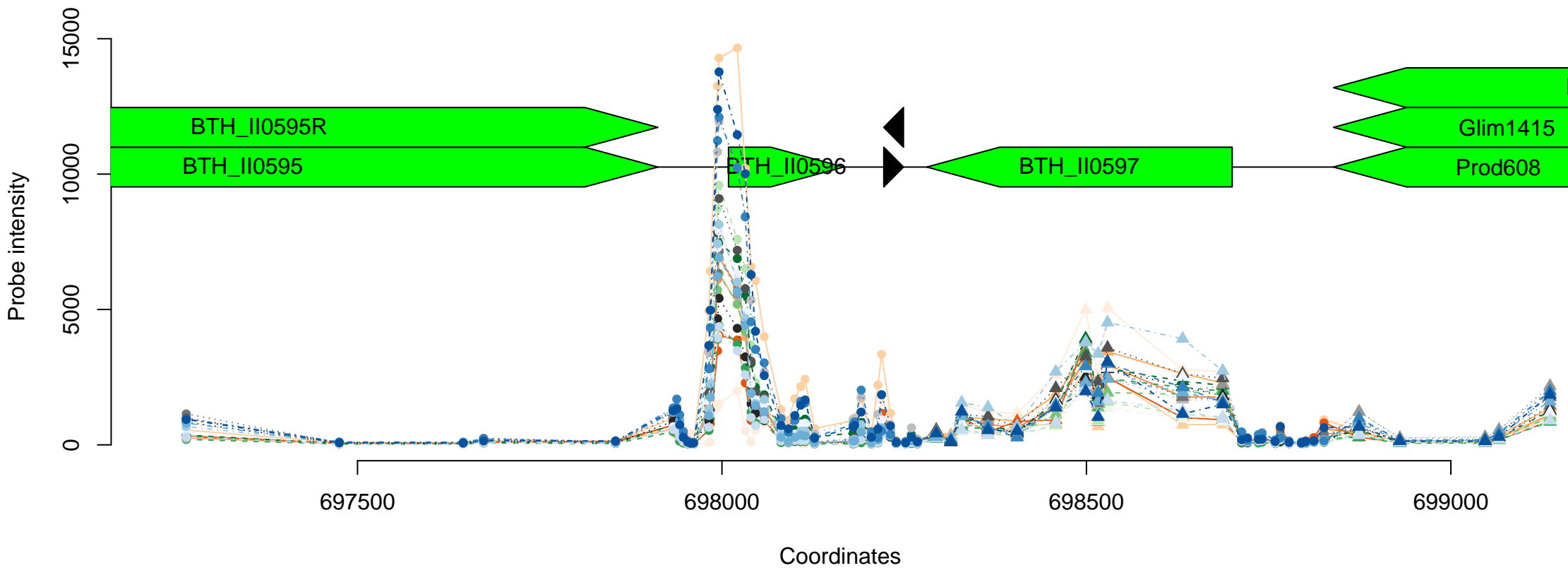

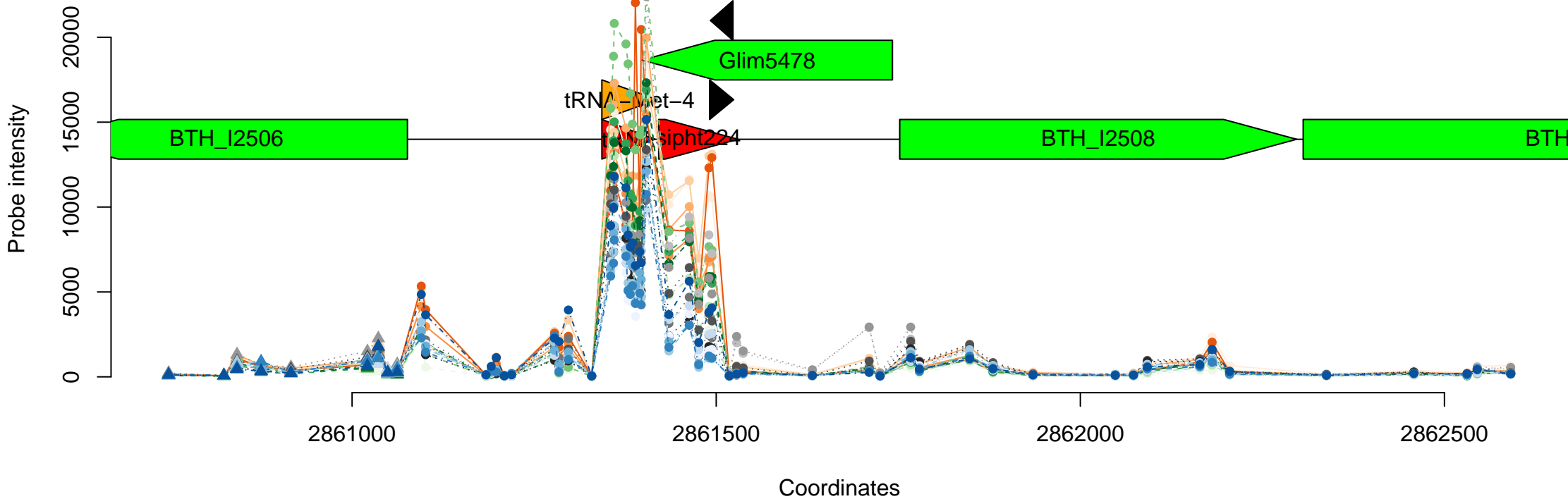

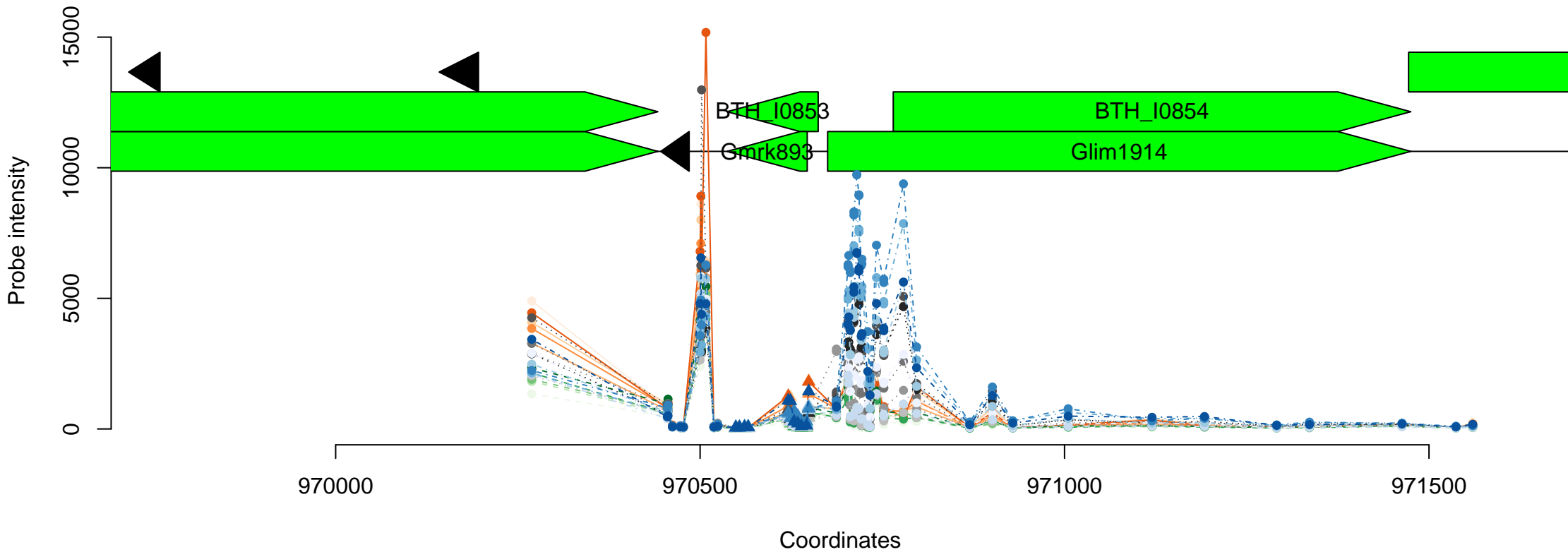

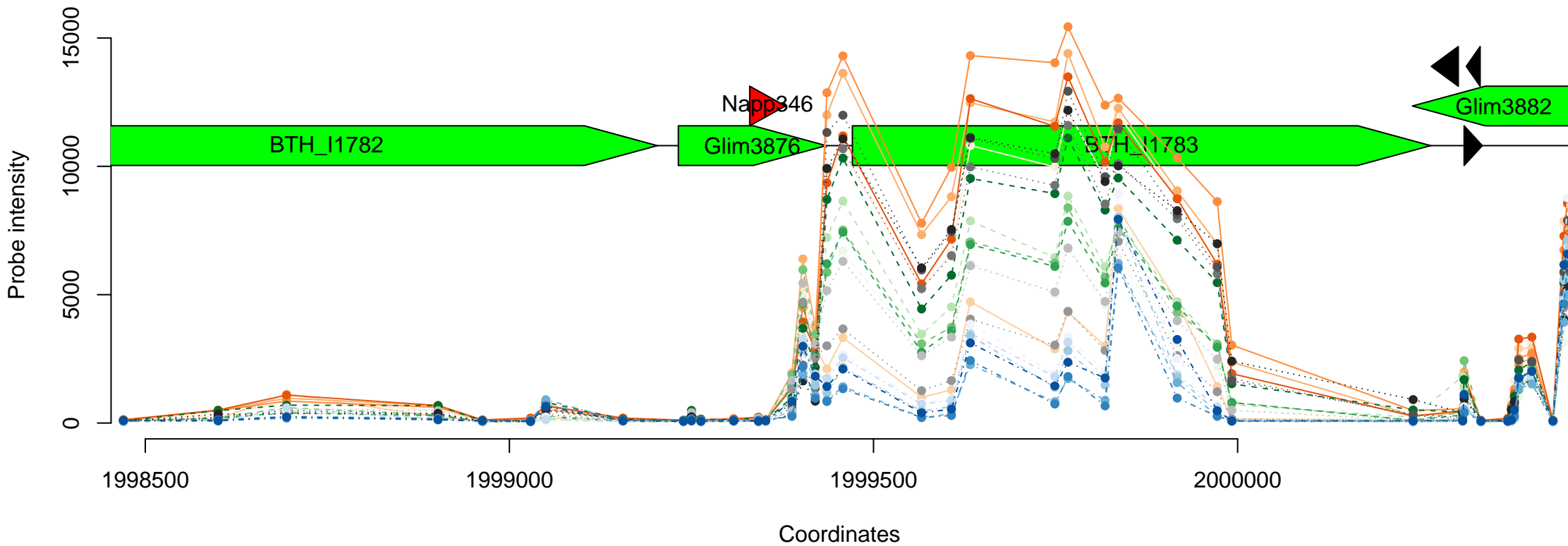

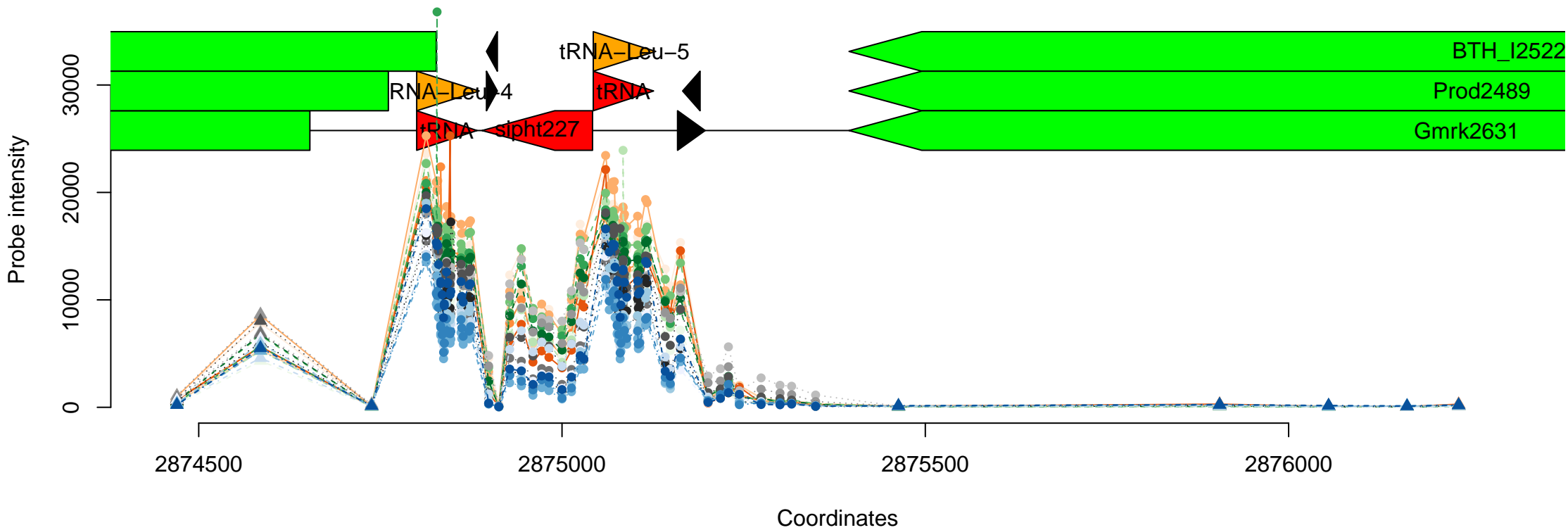

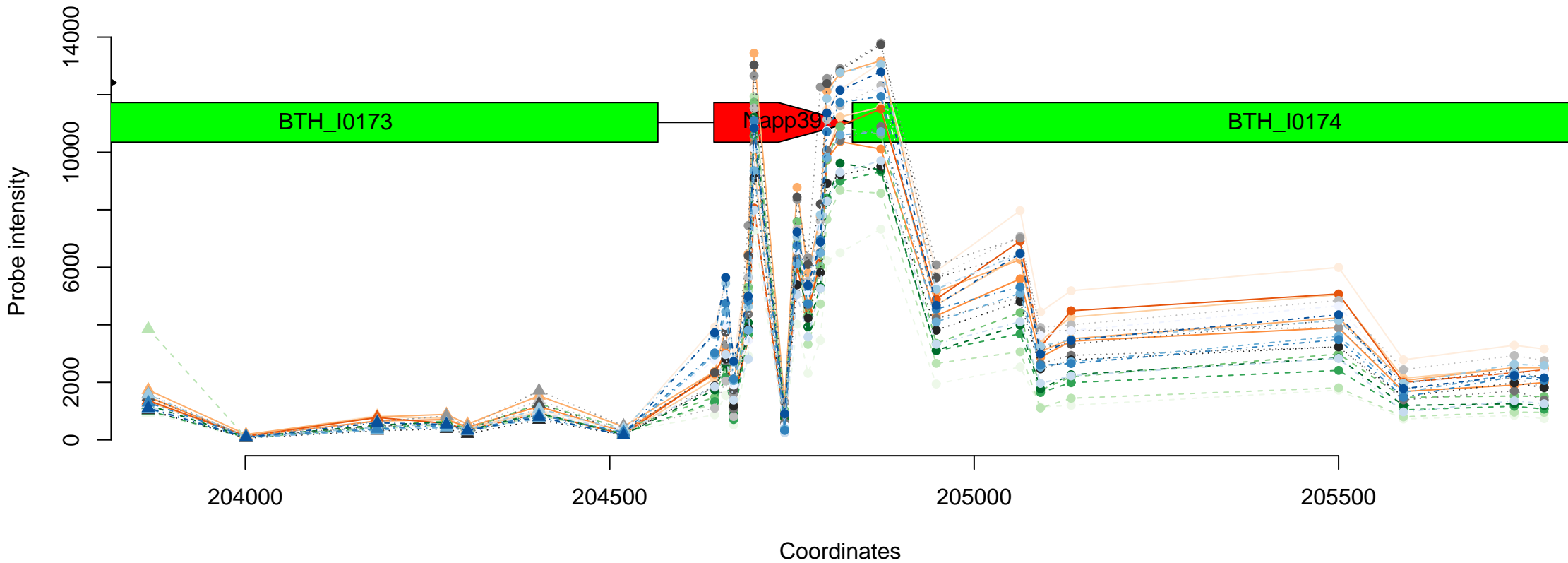

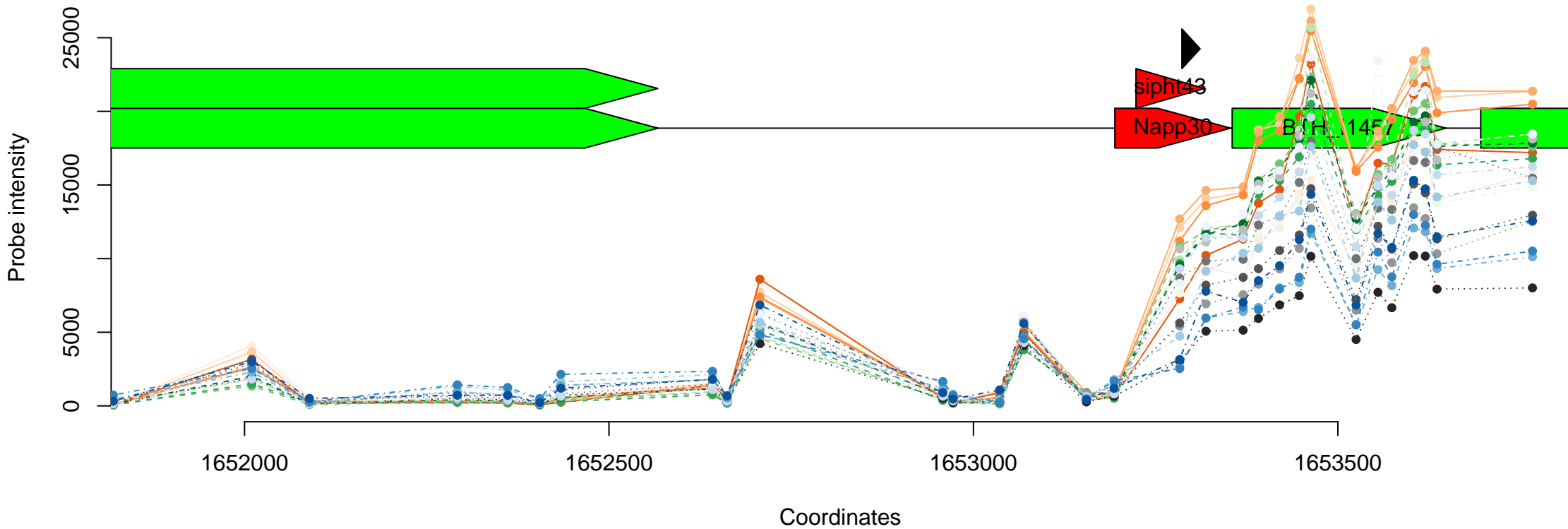

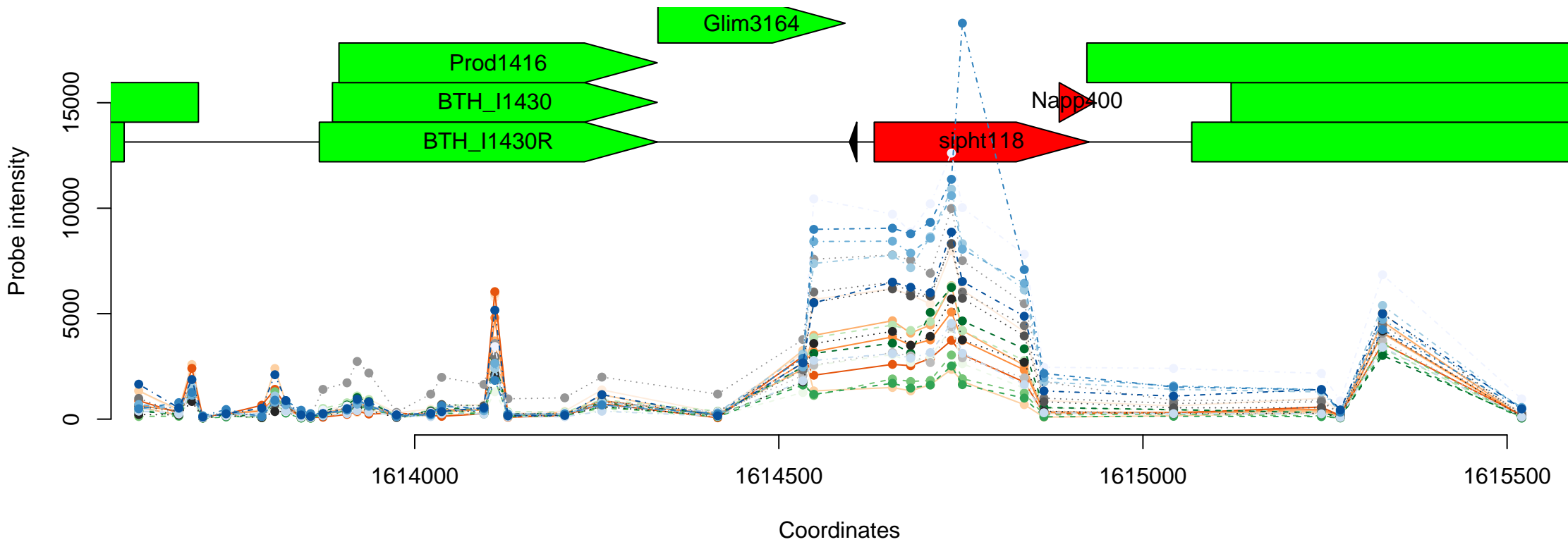

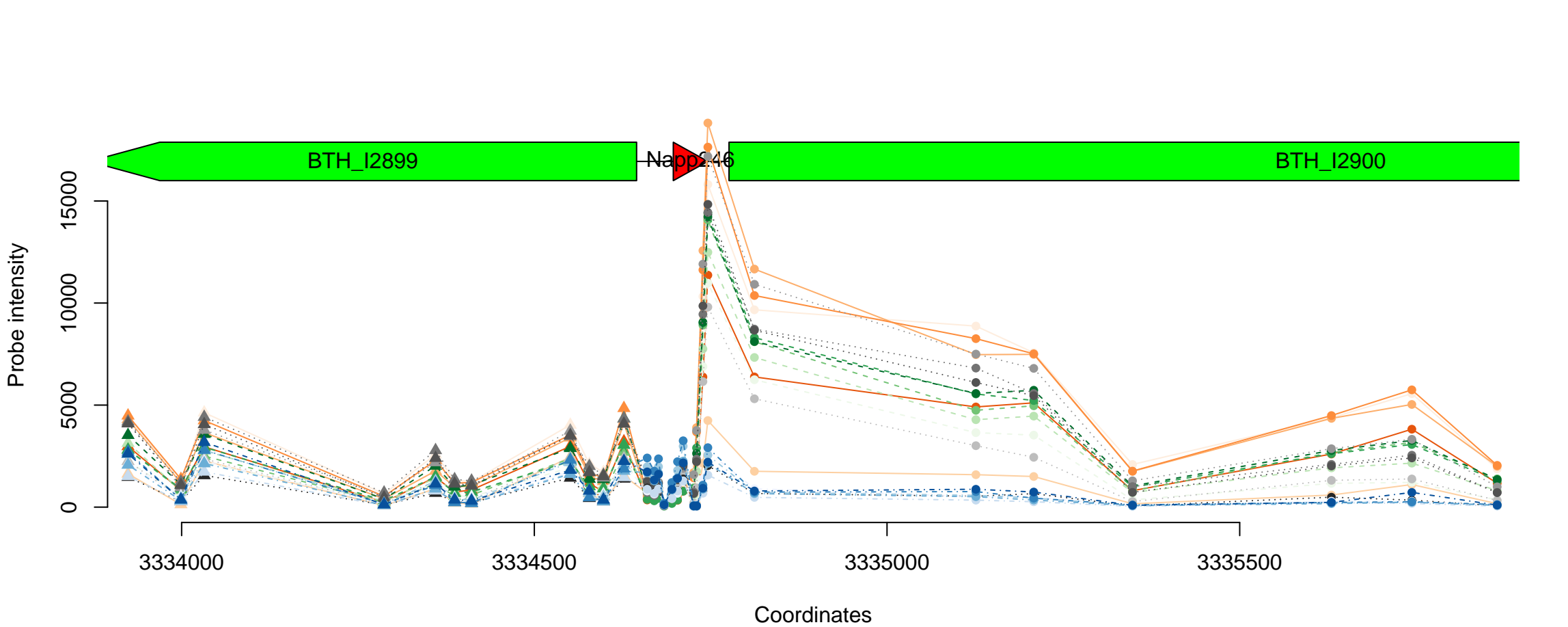

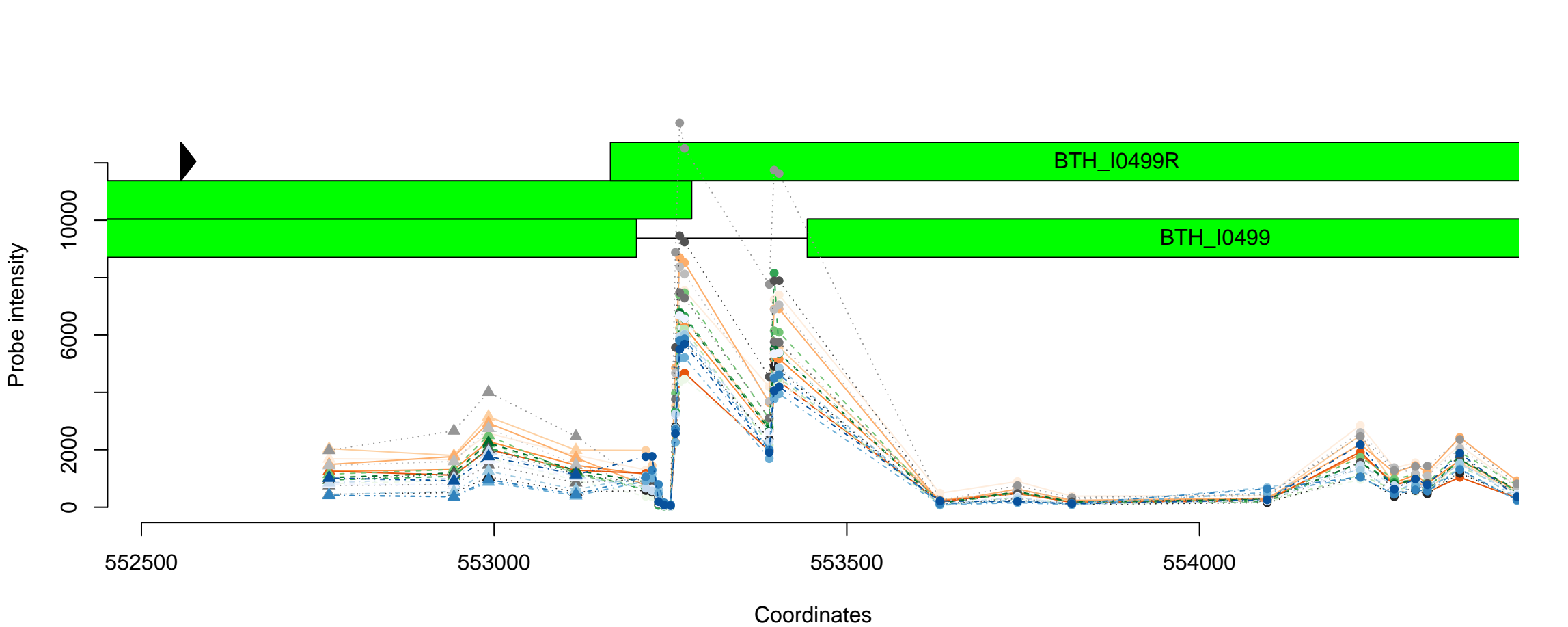

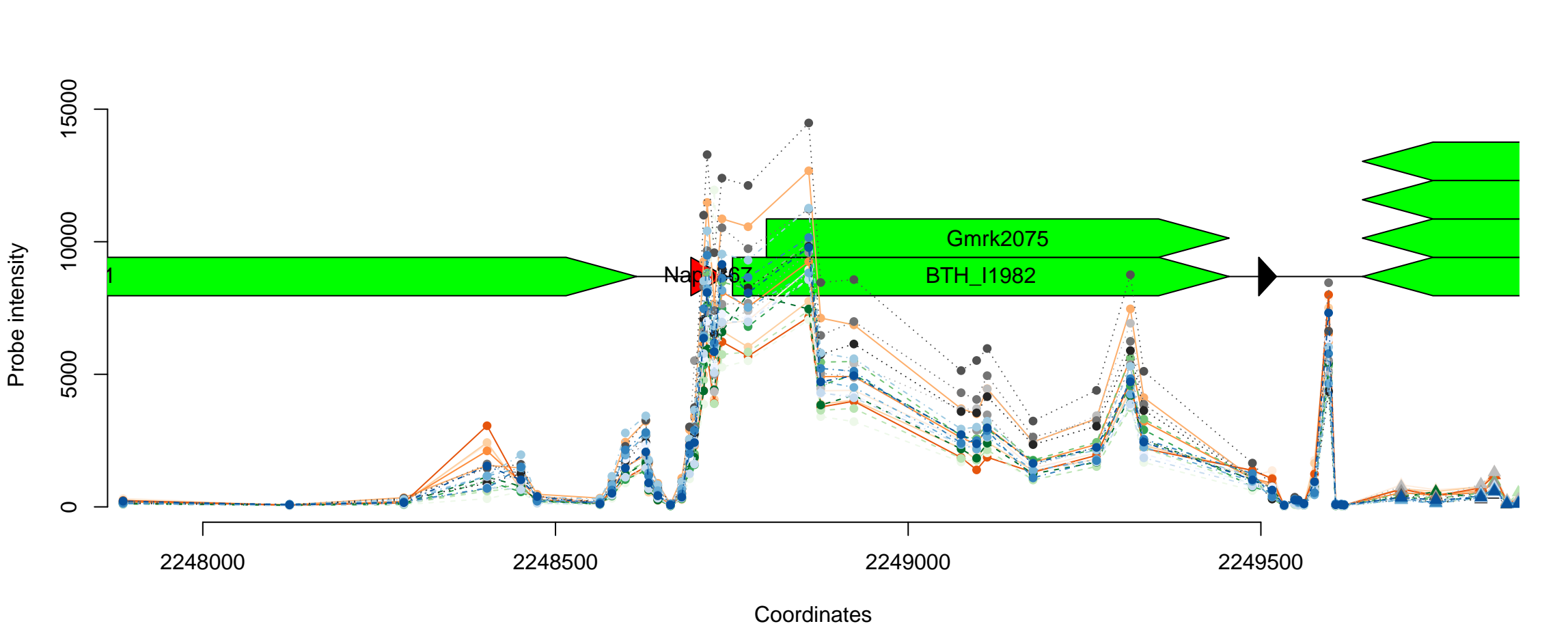

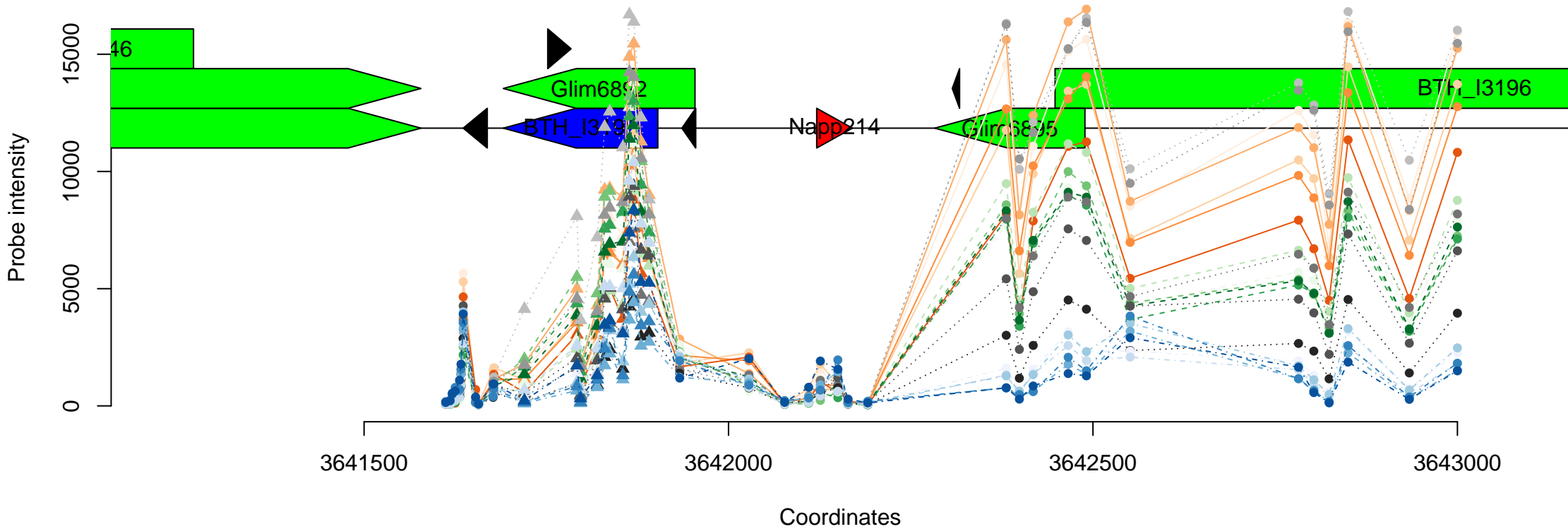

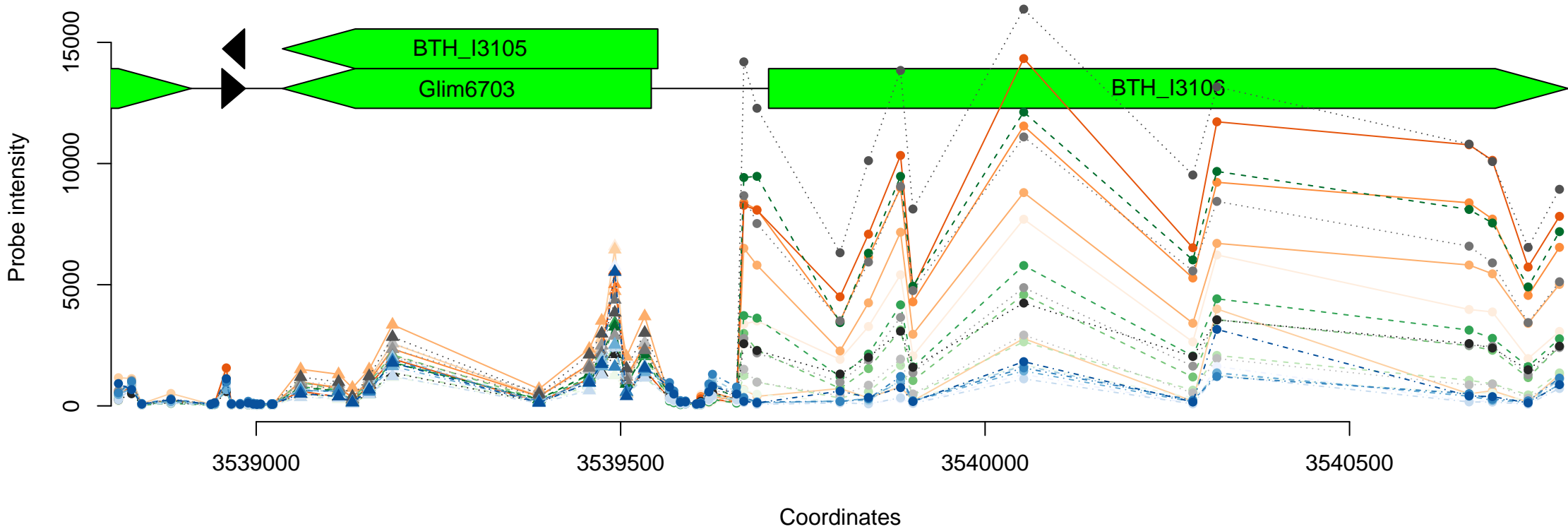

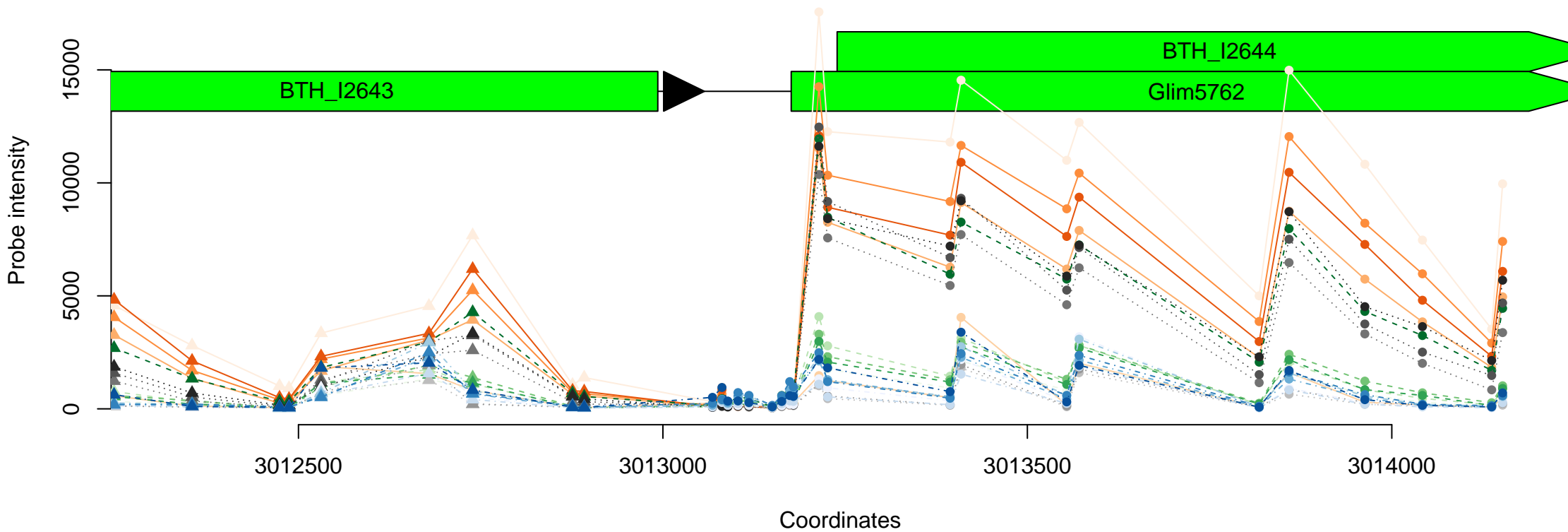

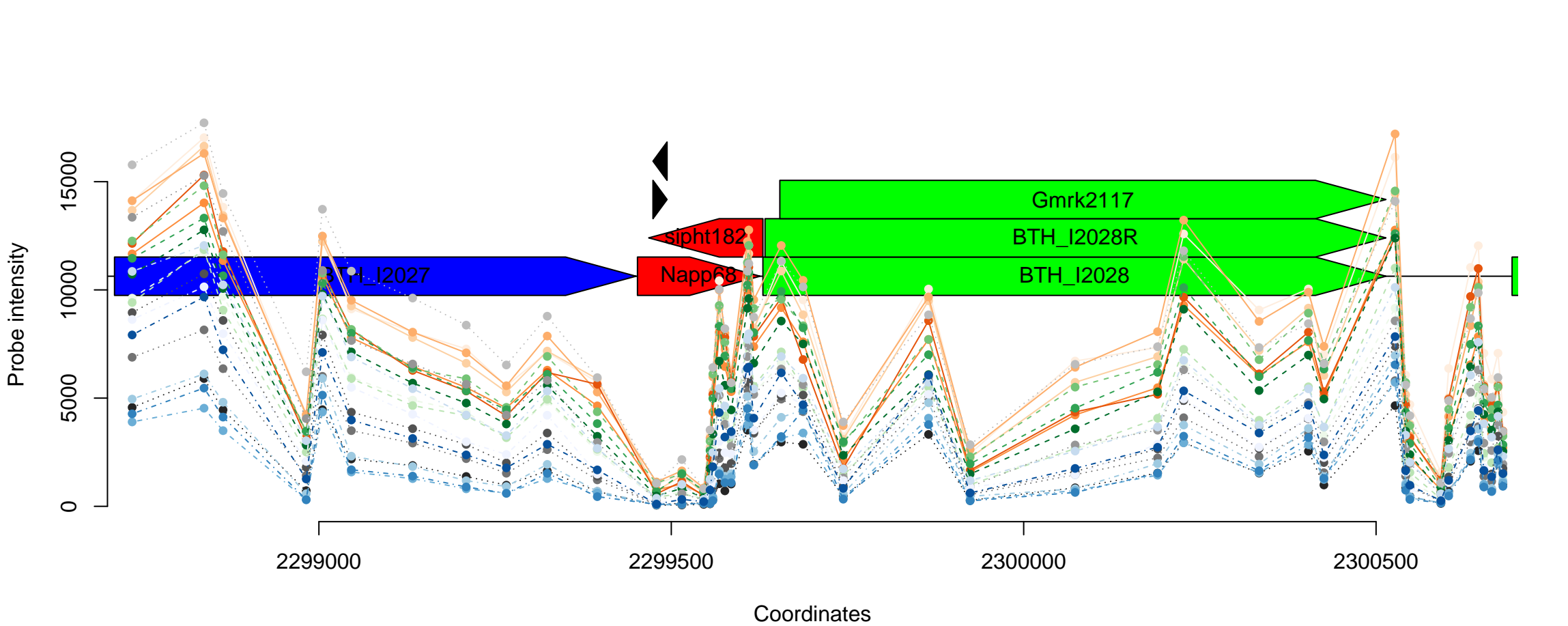

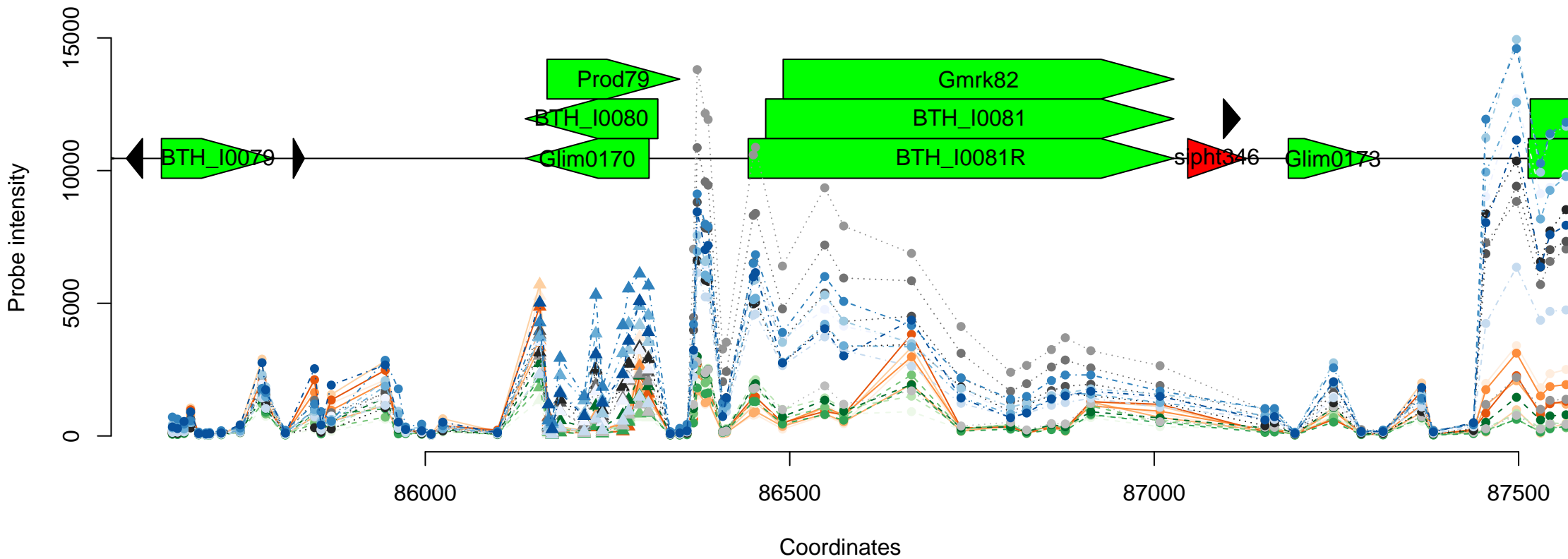

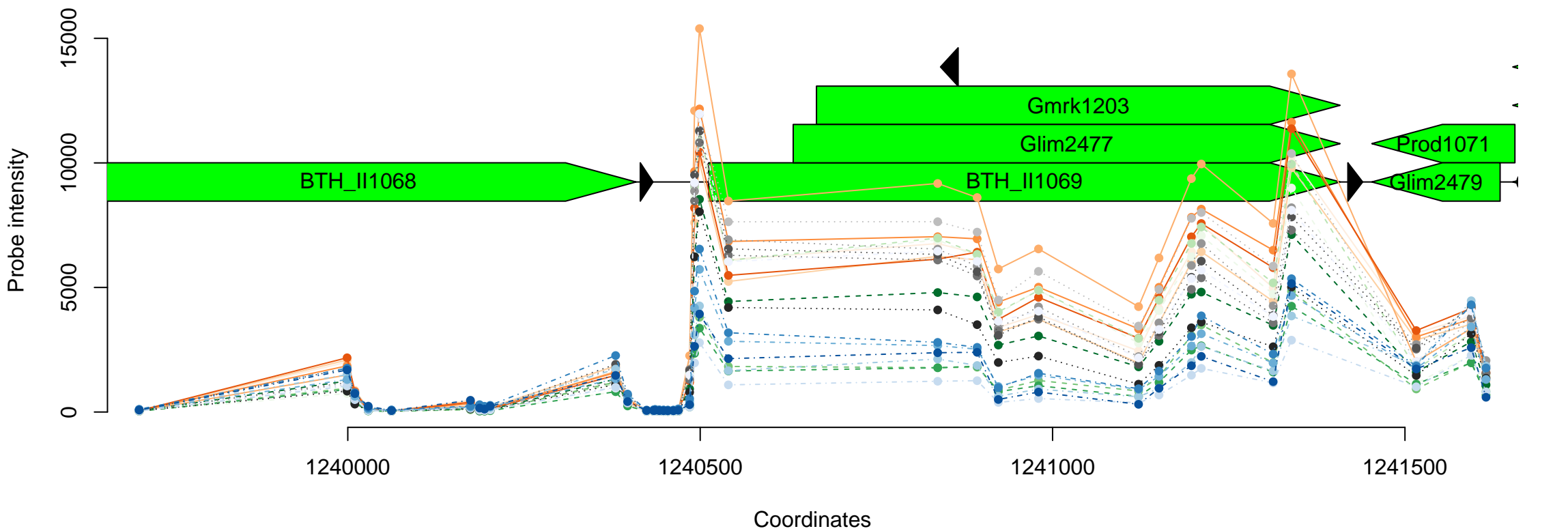

Probe intensity

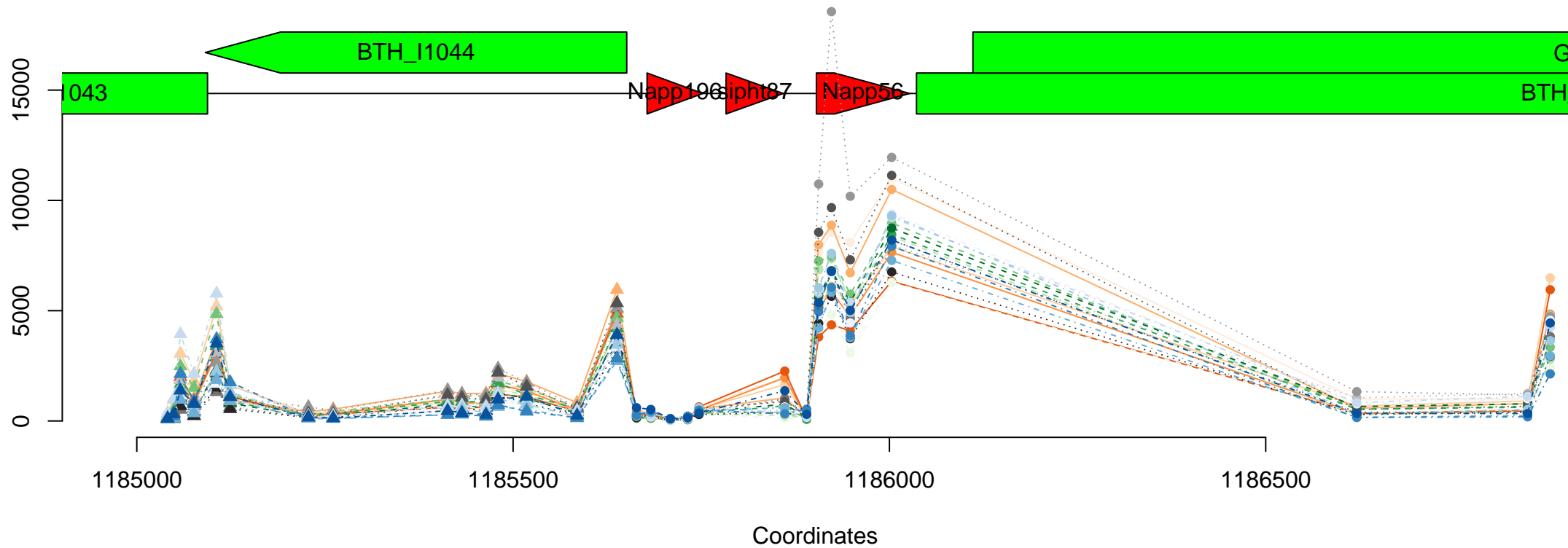

Probe intensity

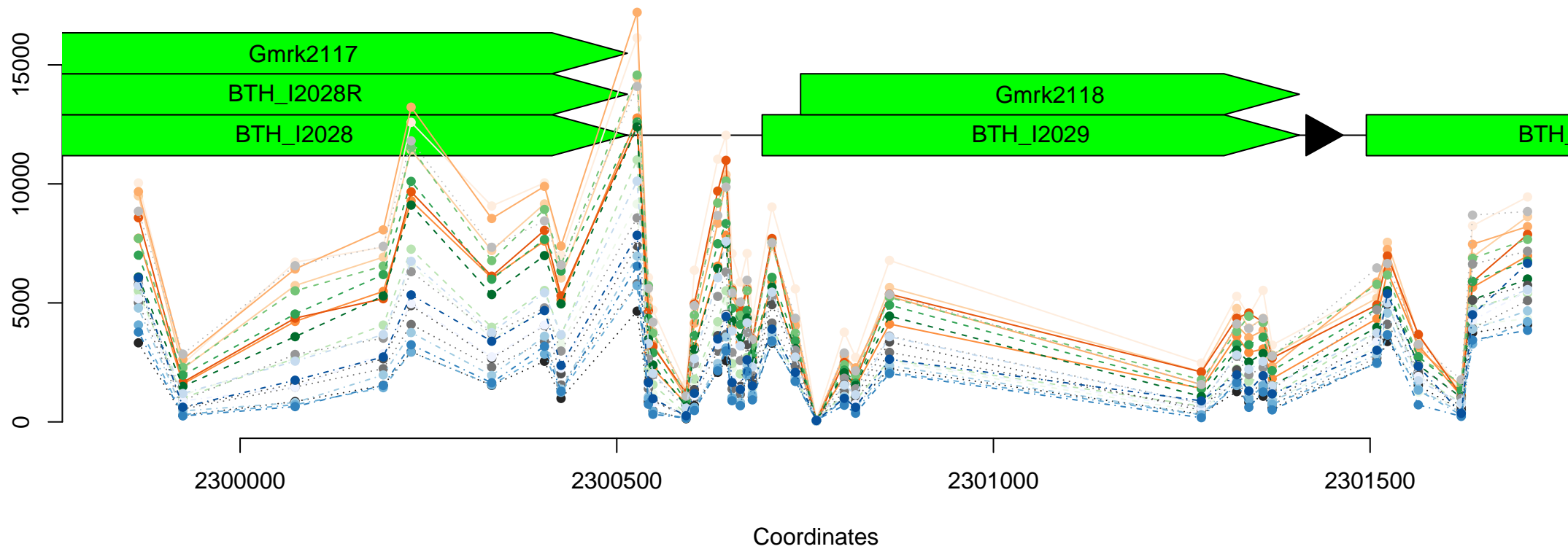

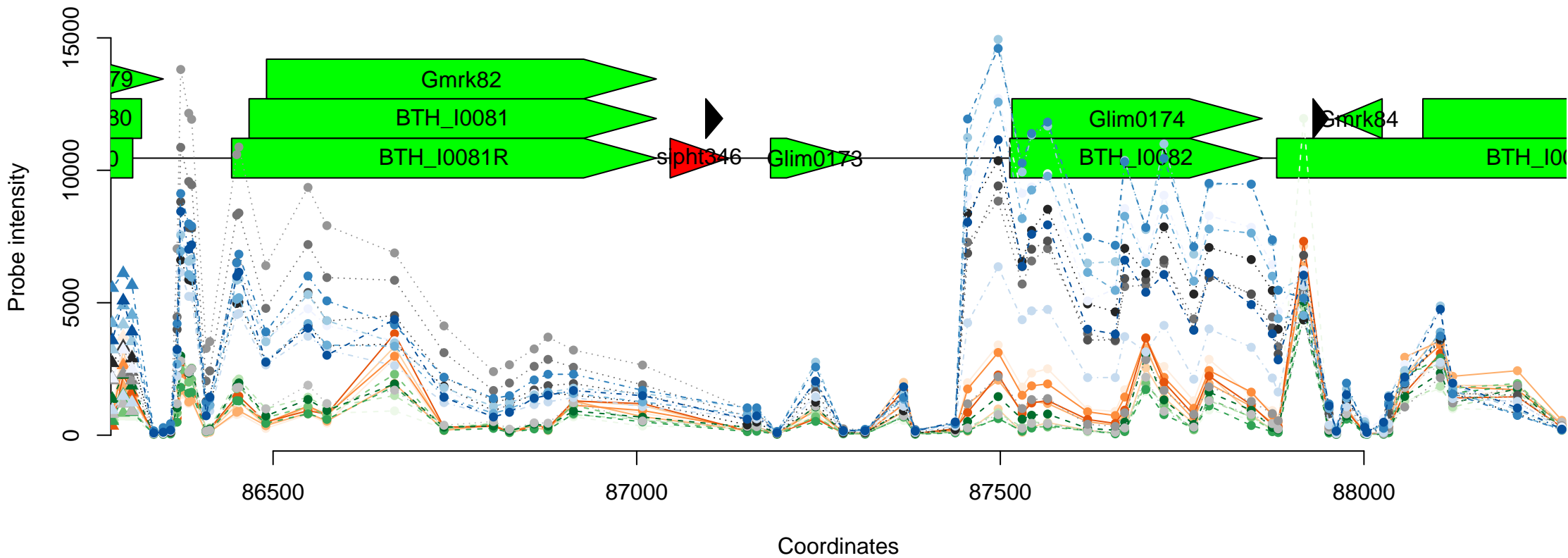

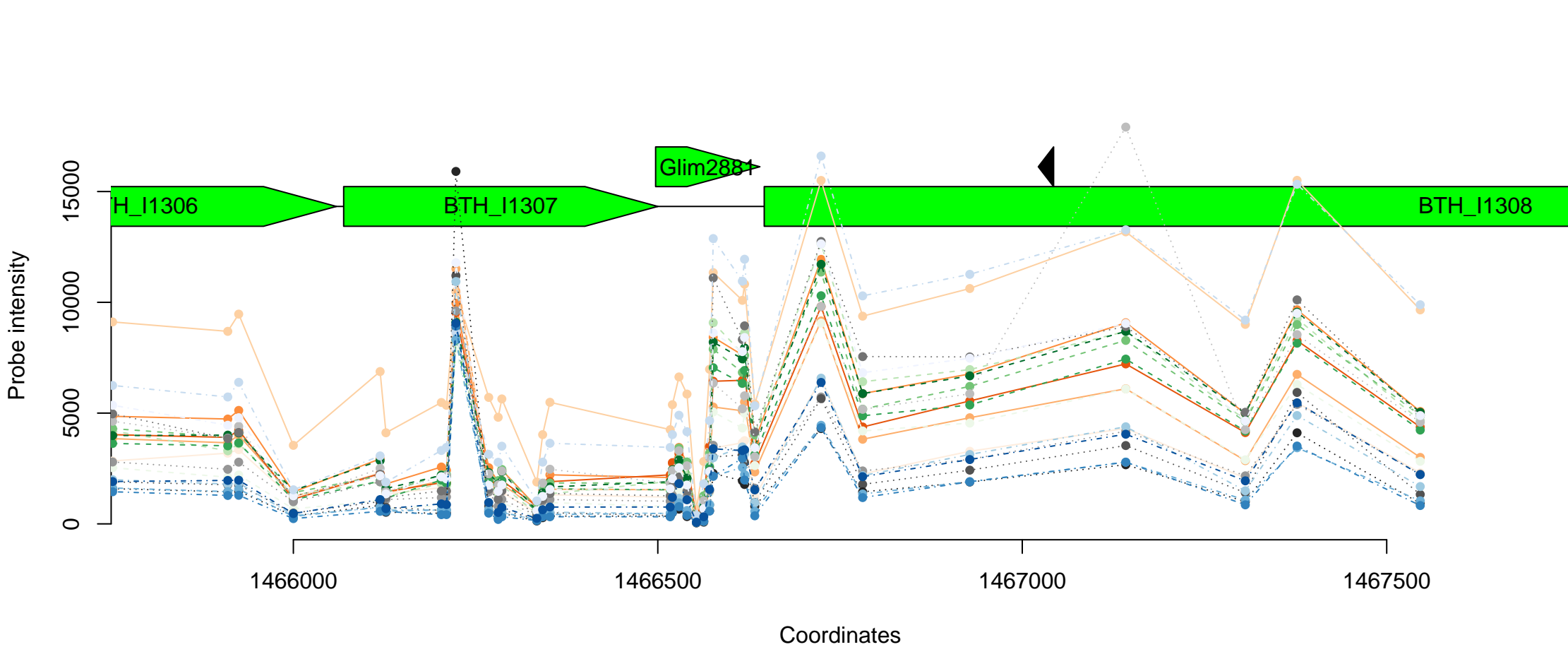

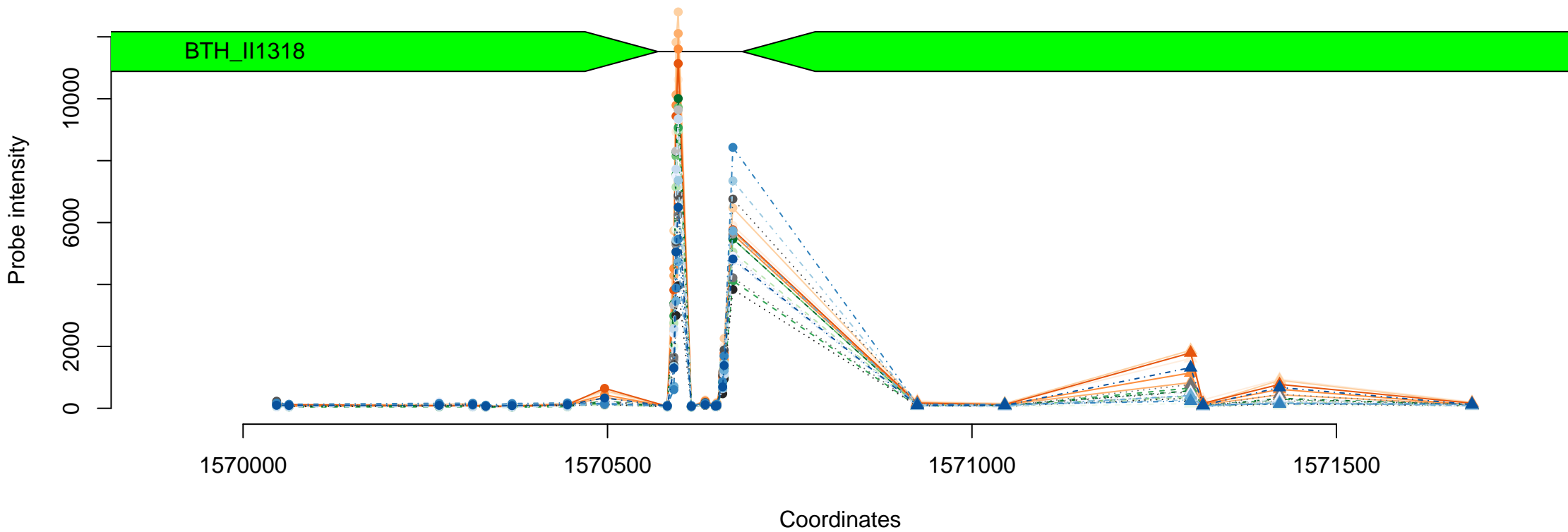

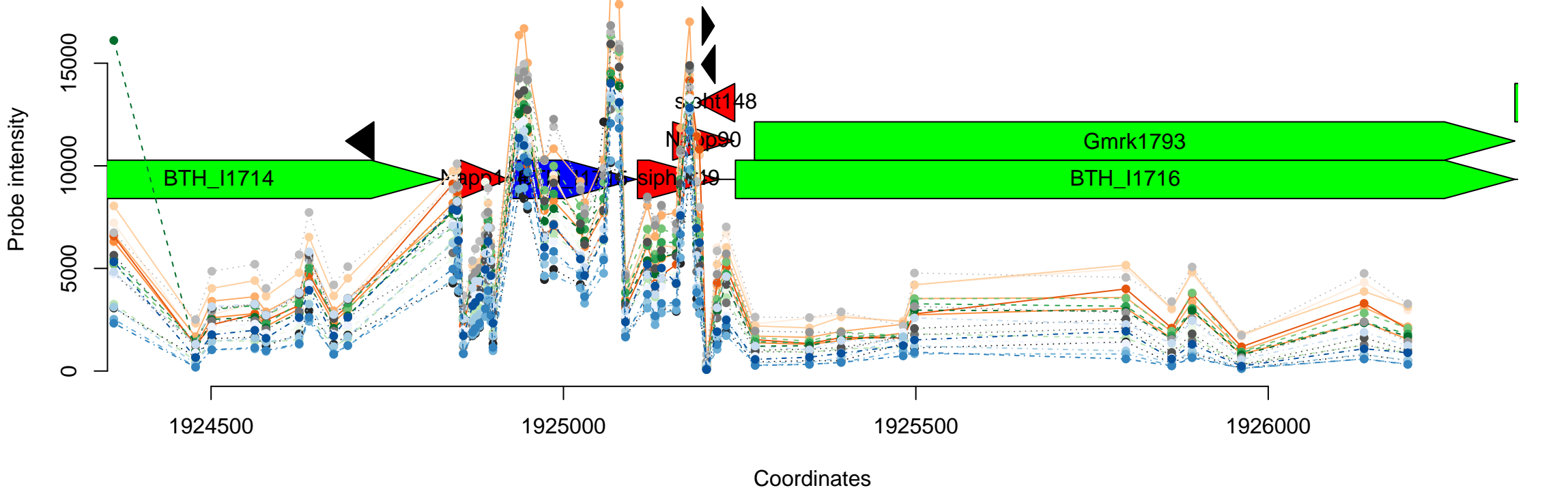

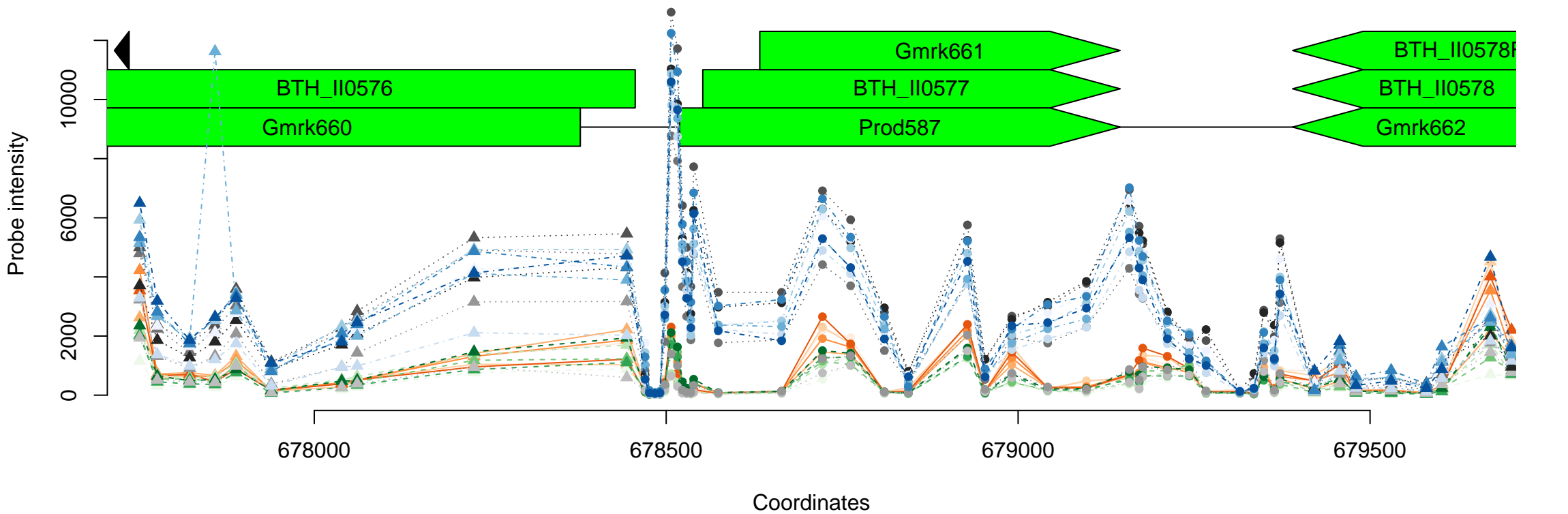

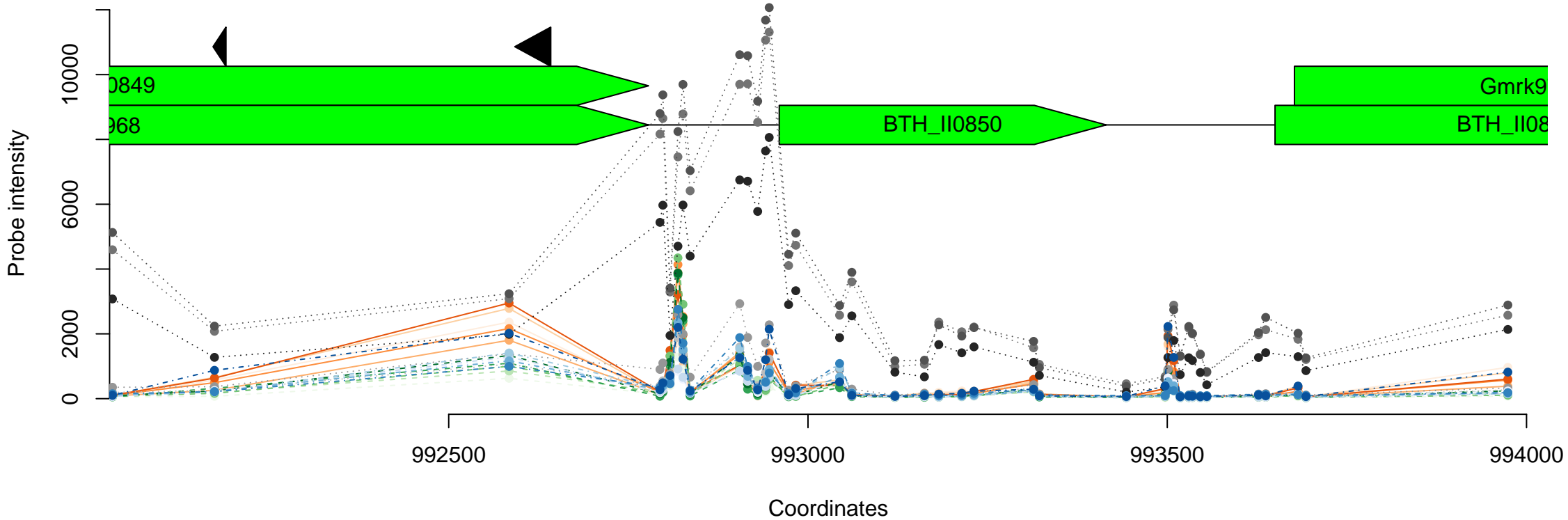

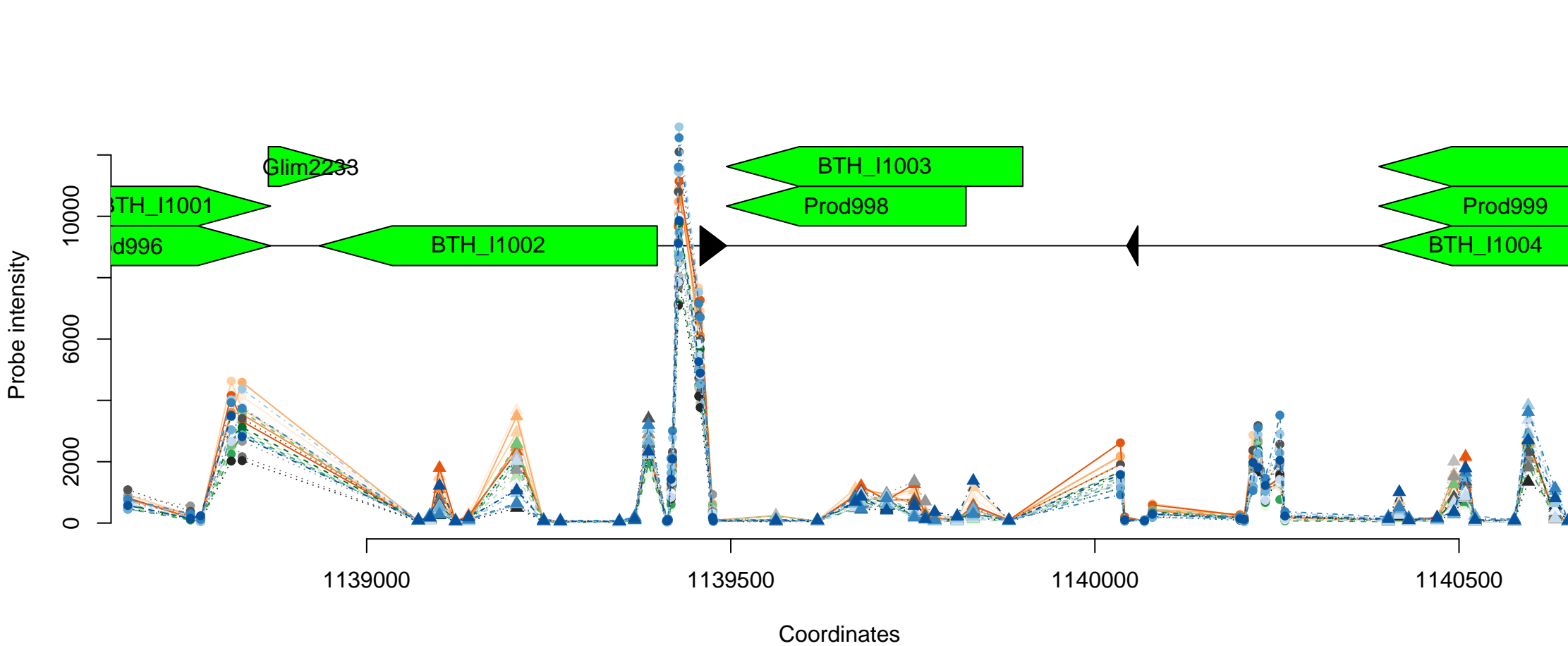

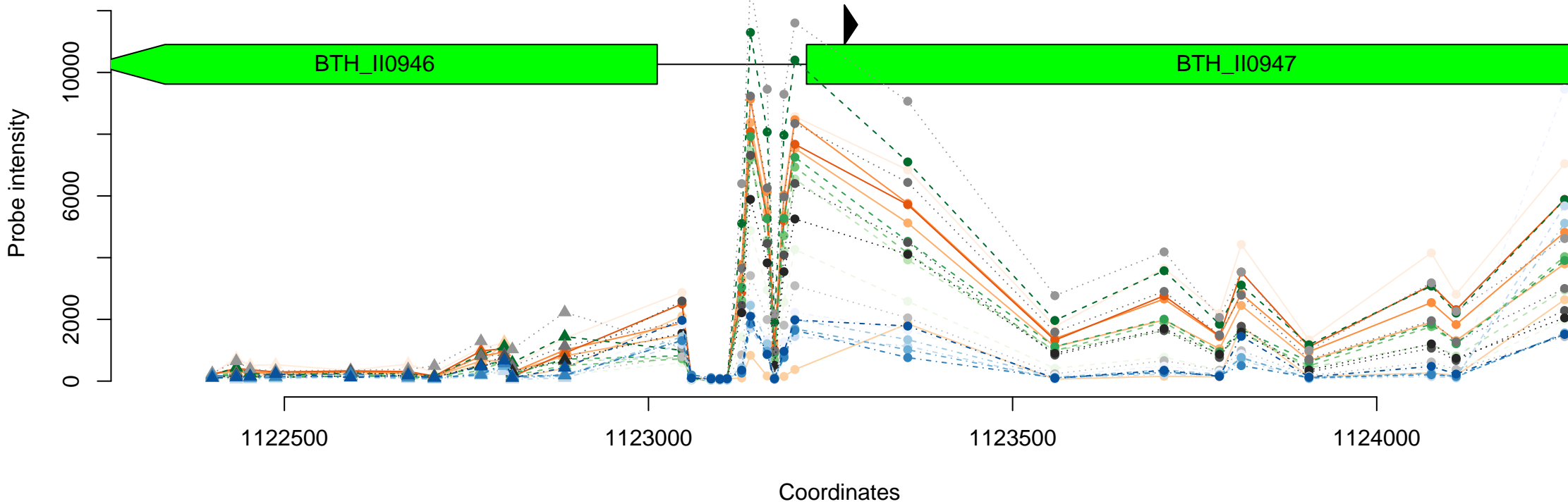

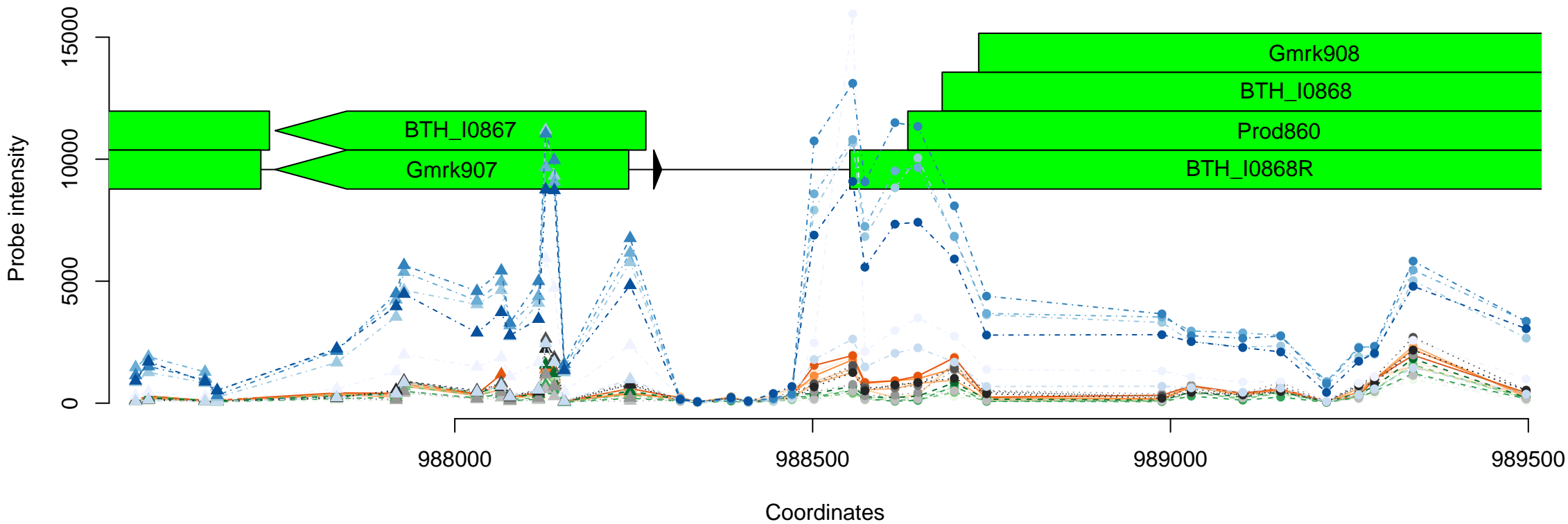

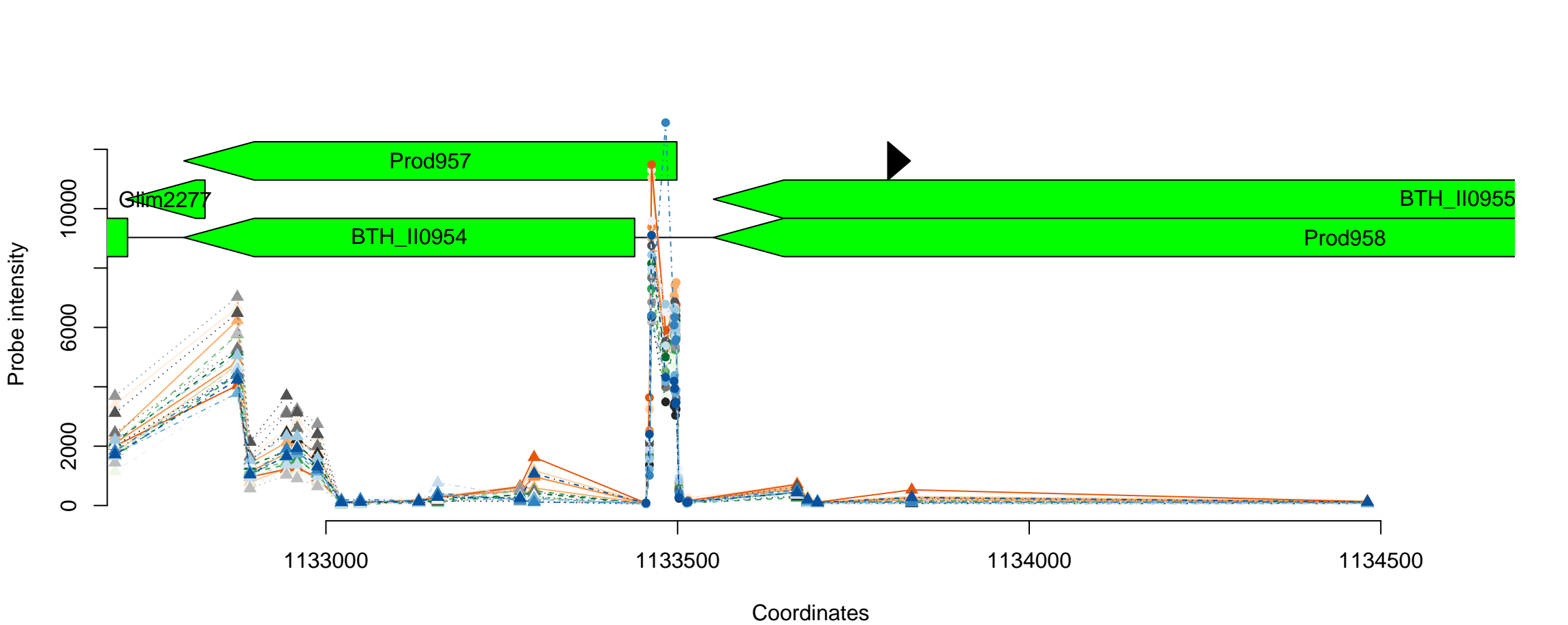

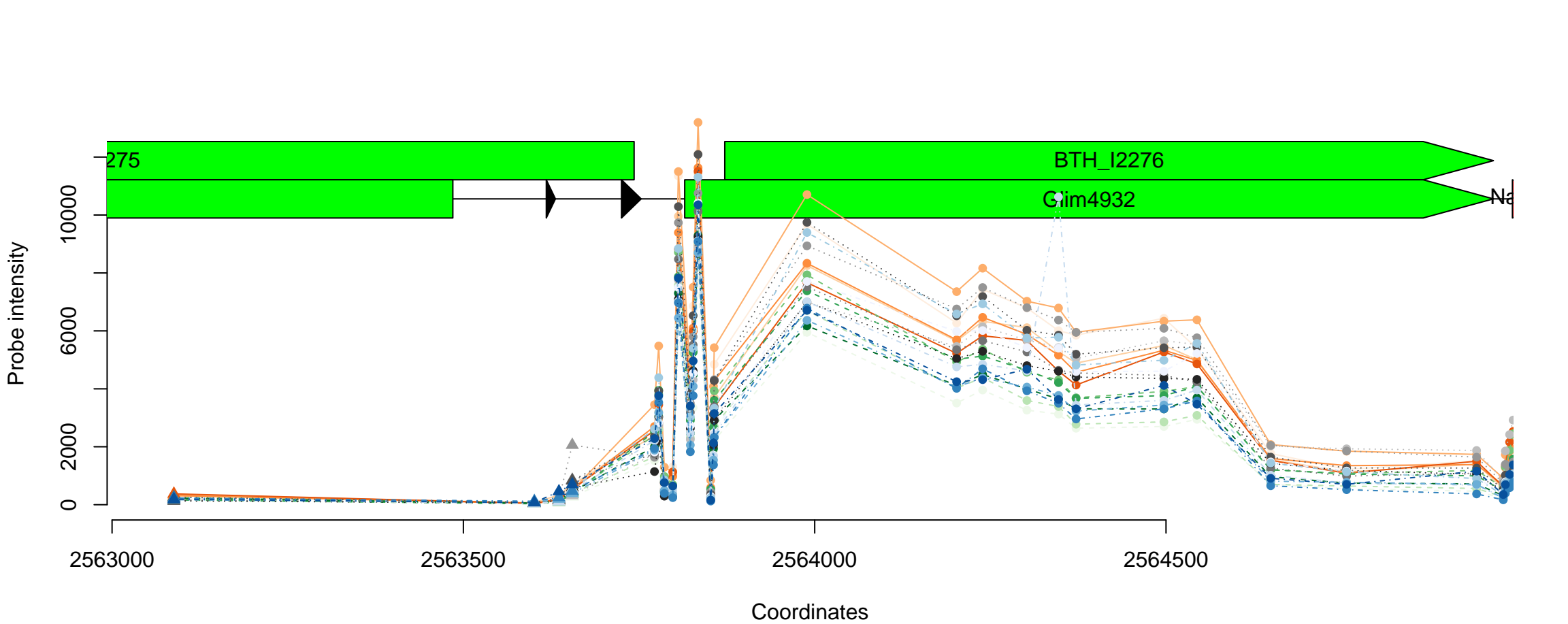

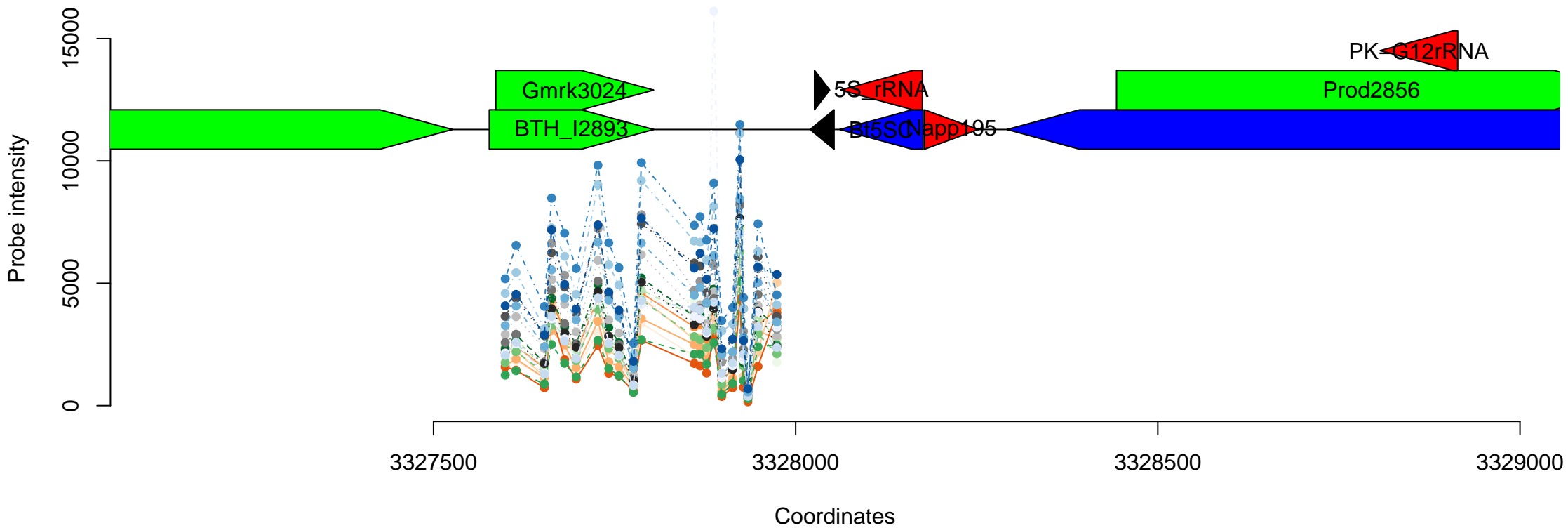

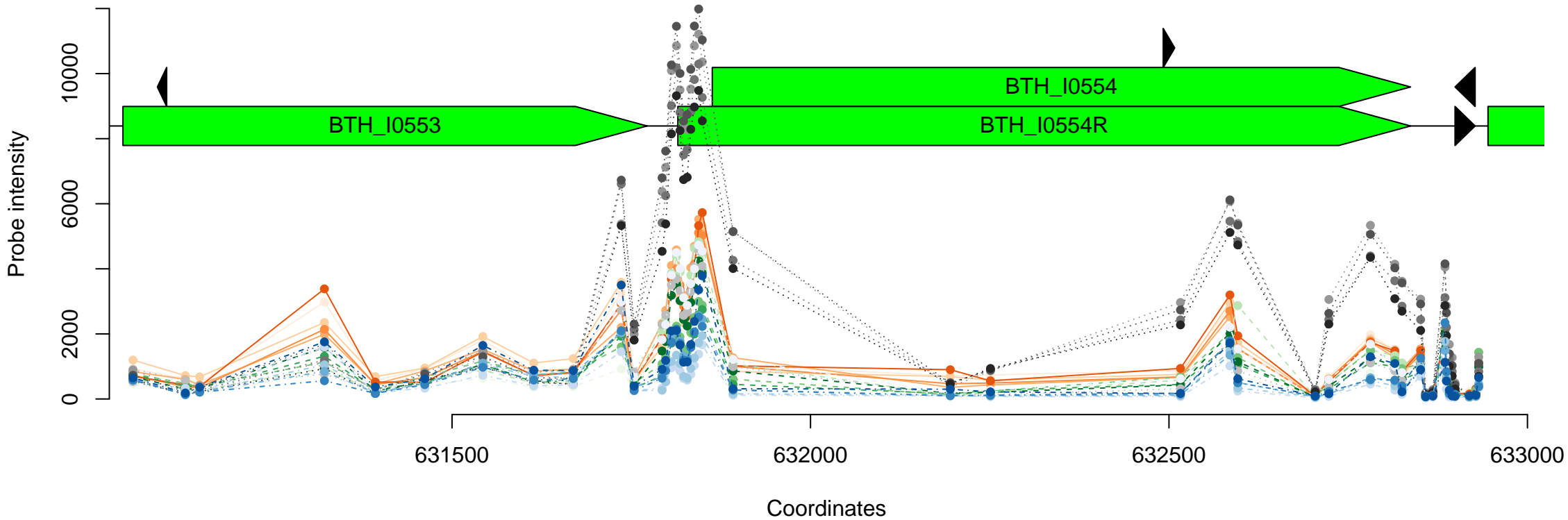

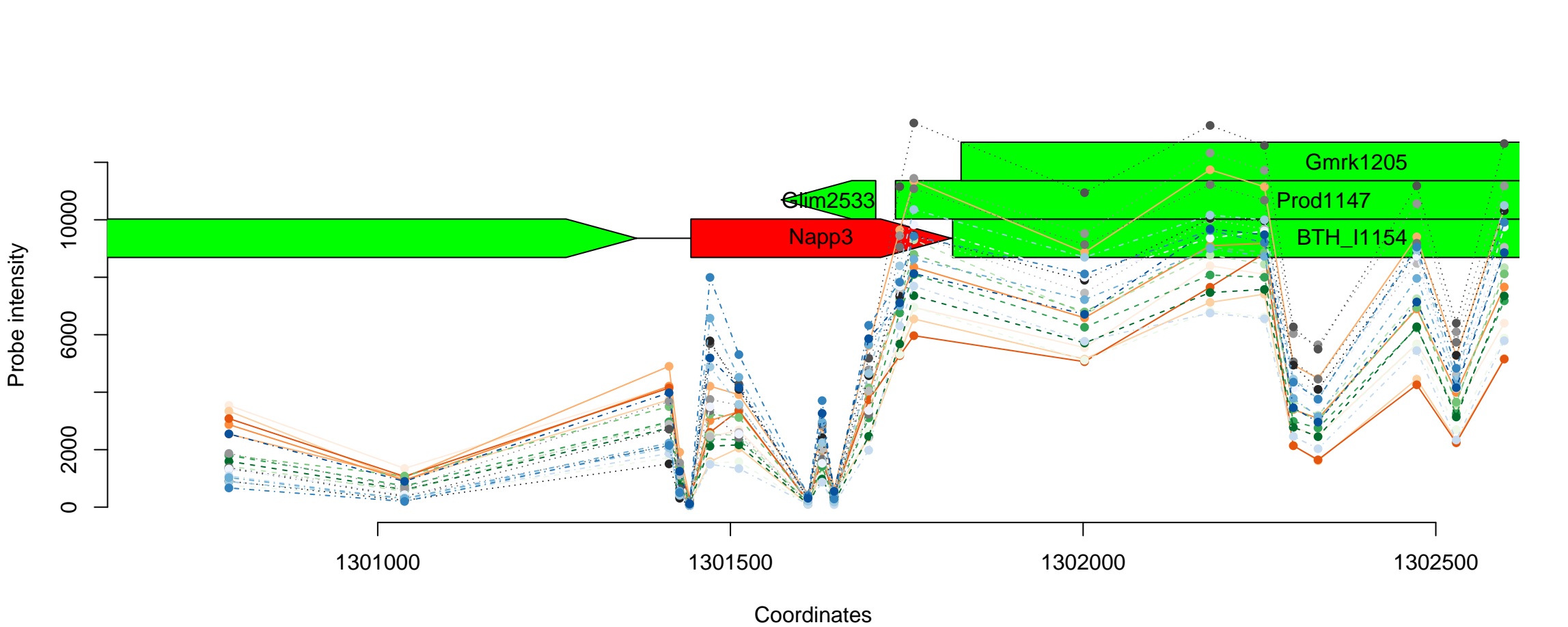

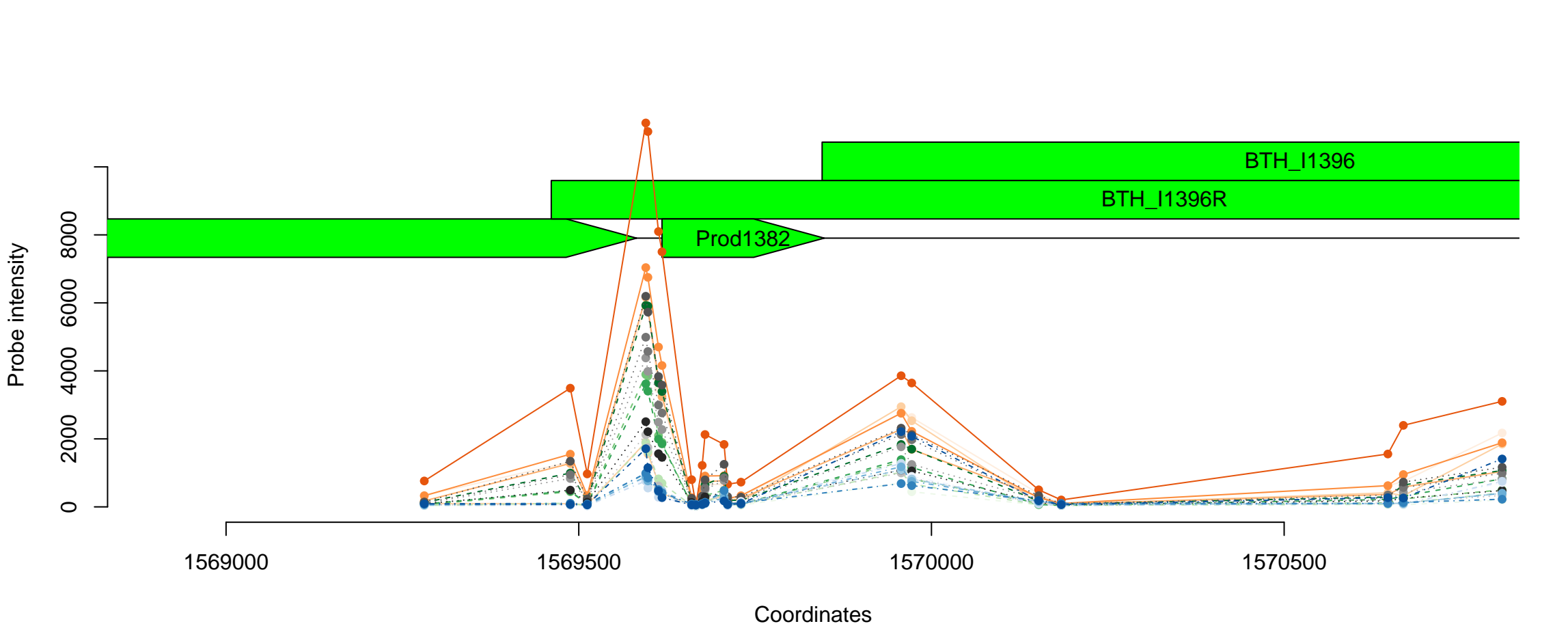

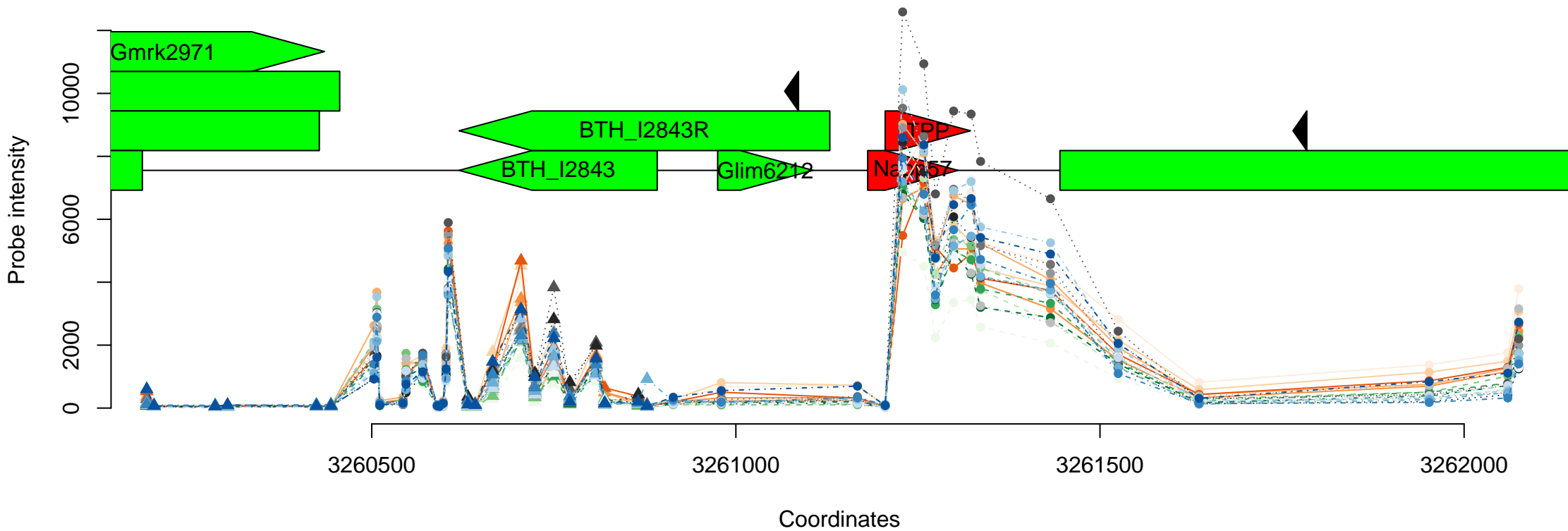

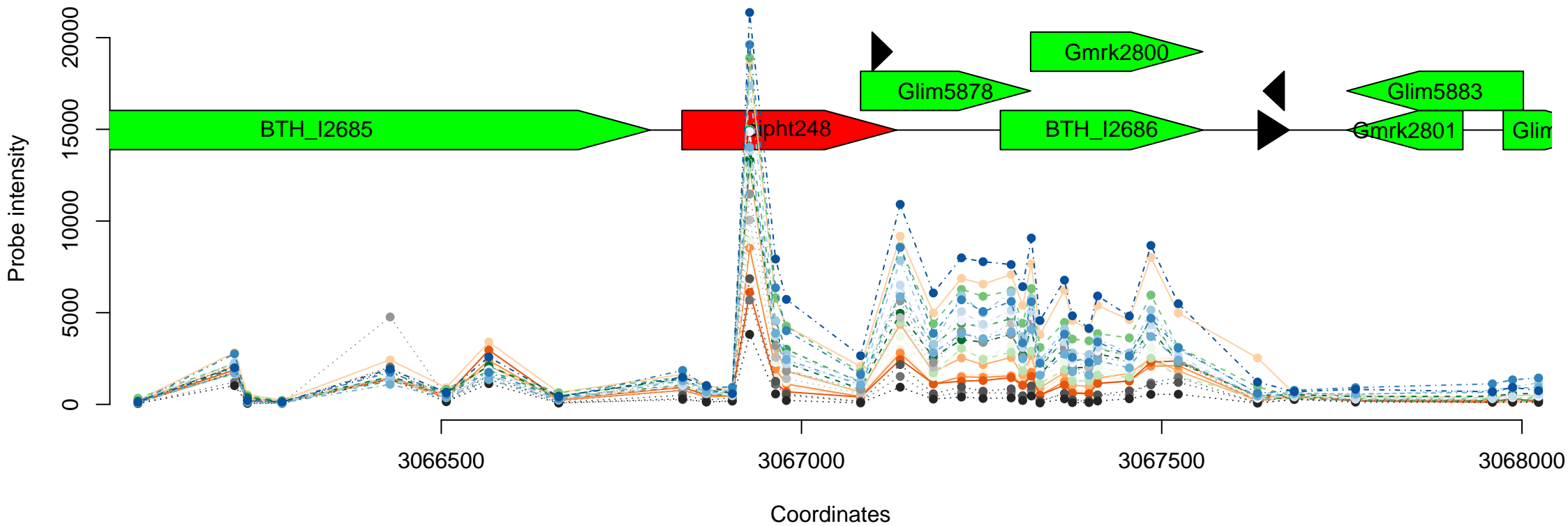

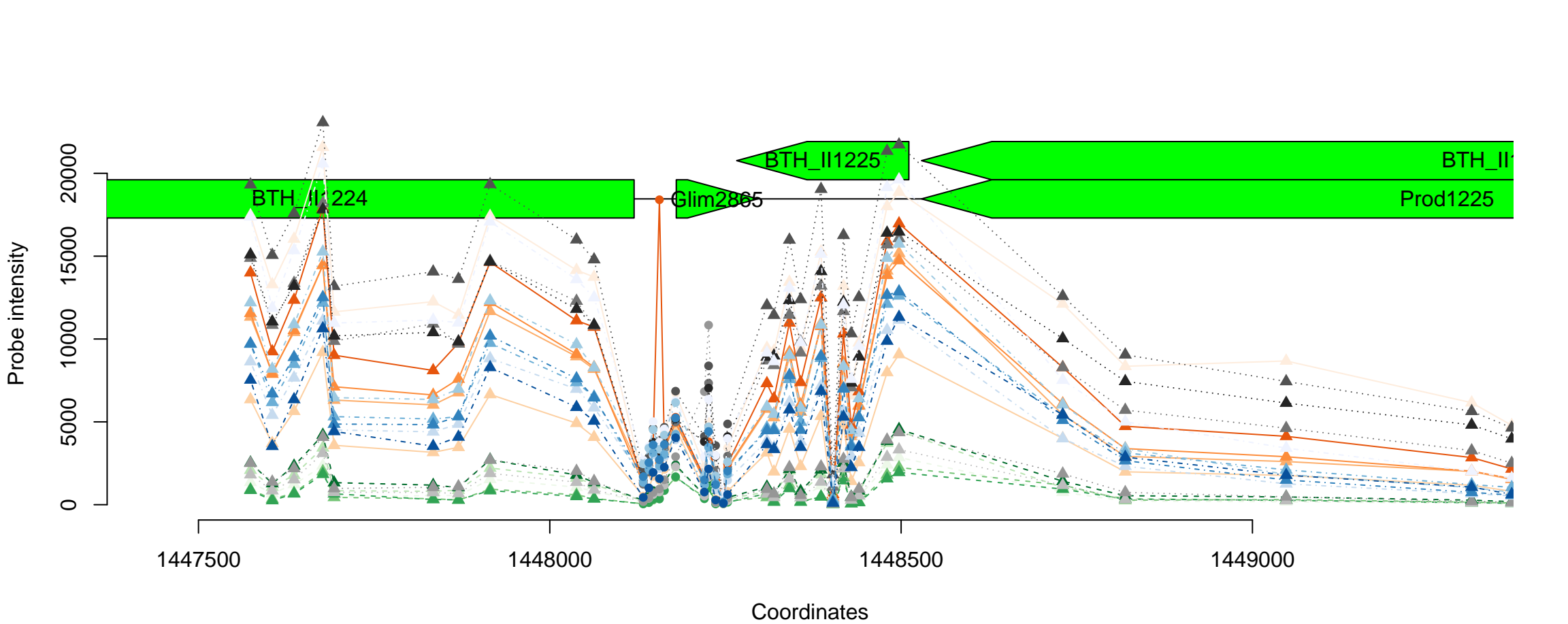

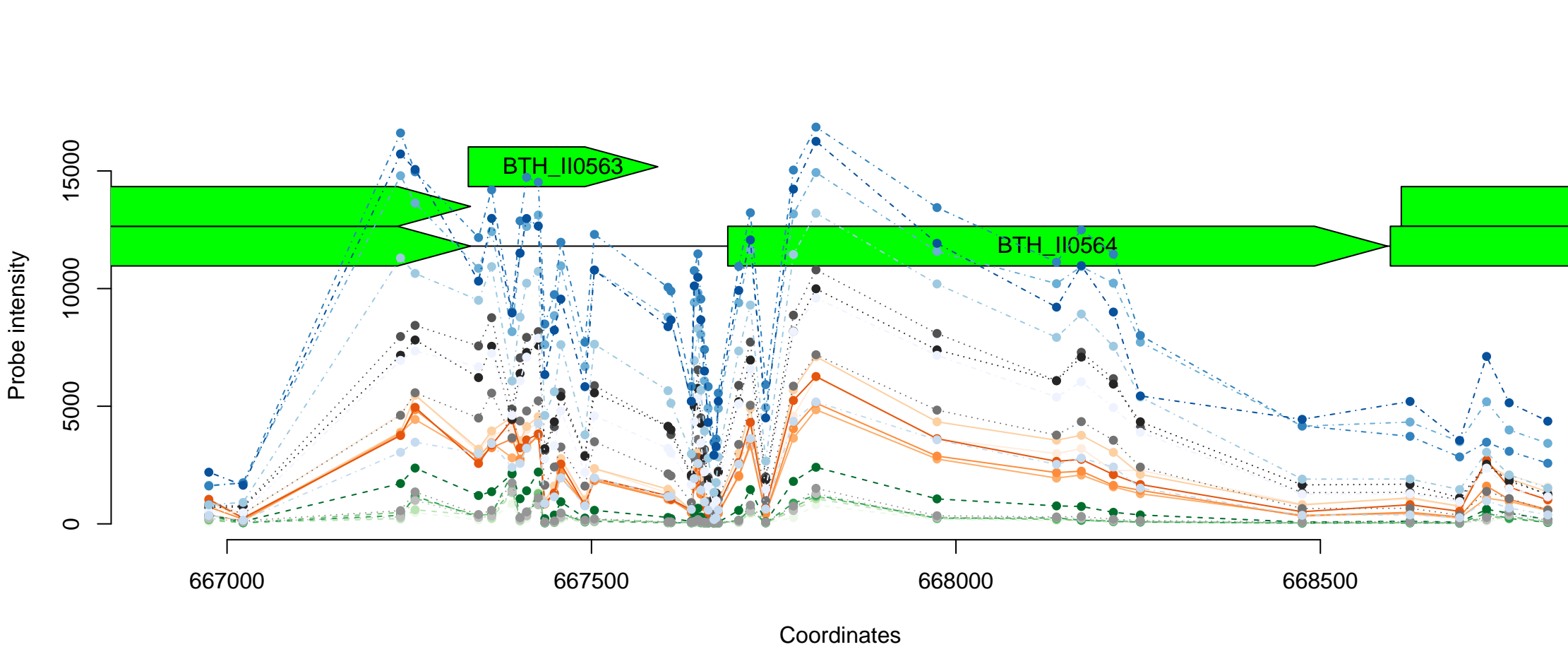

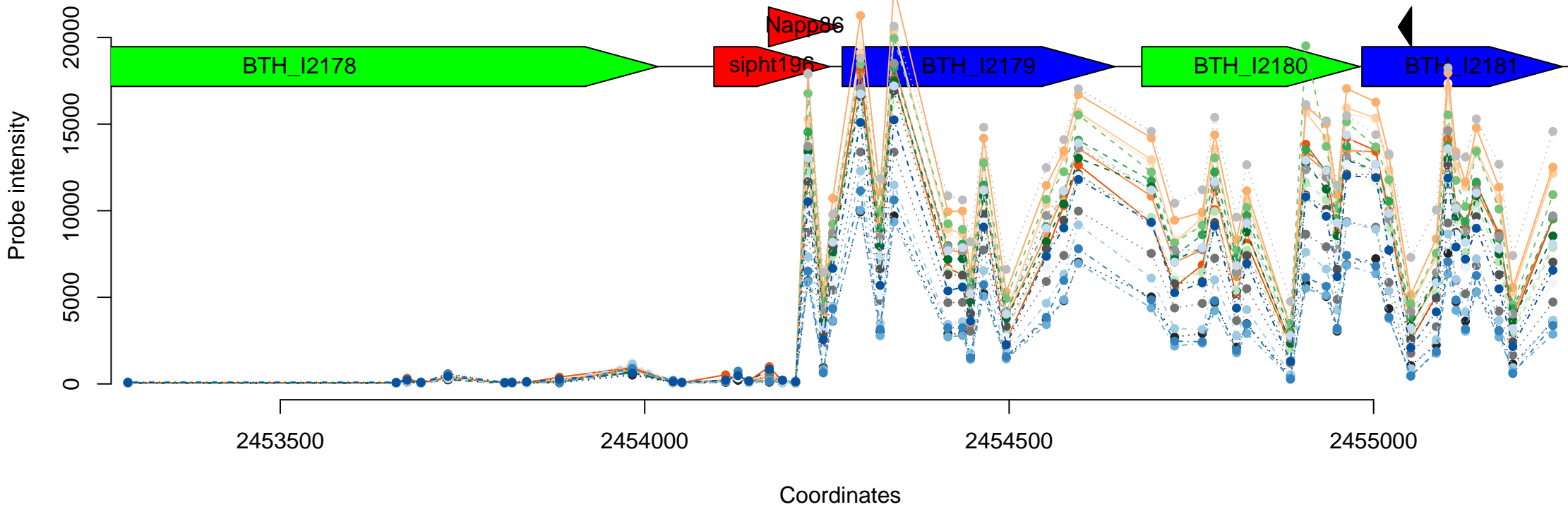

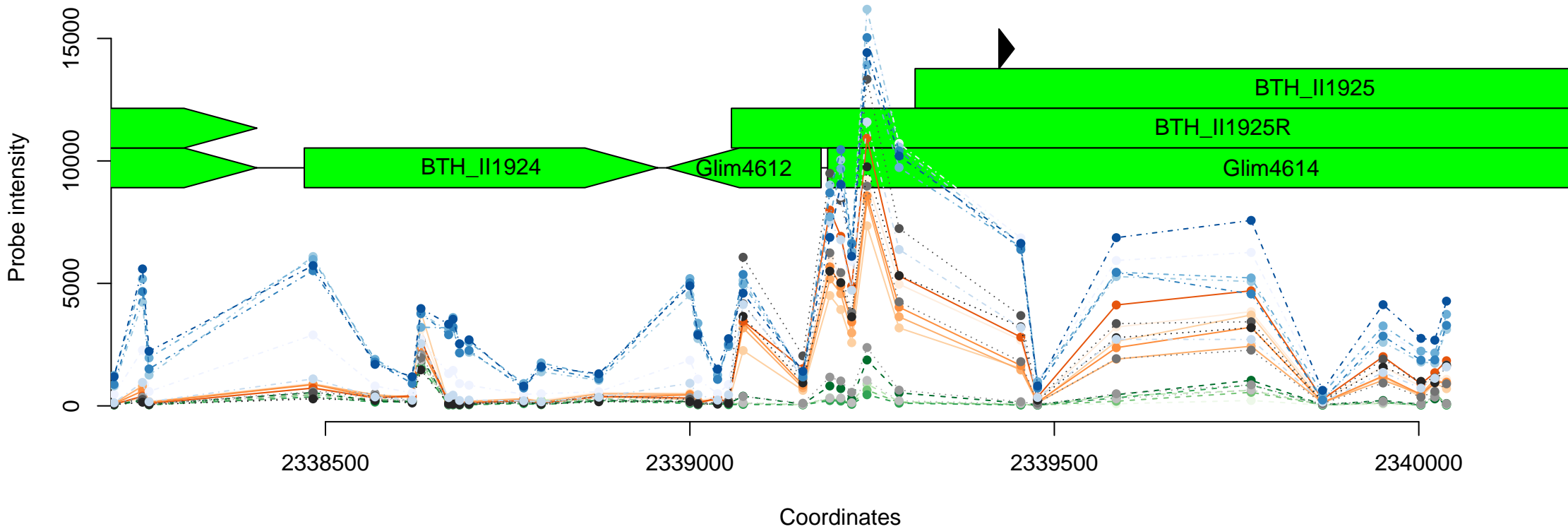

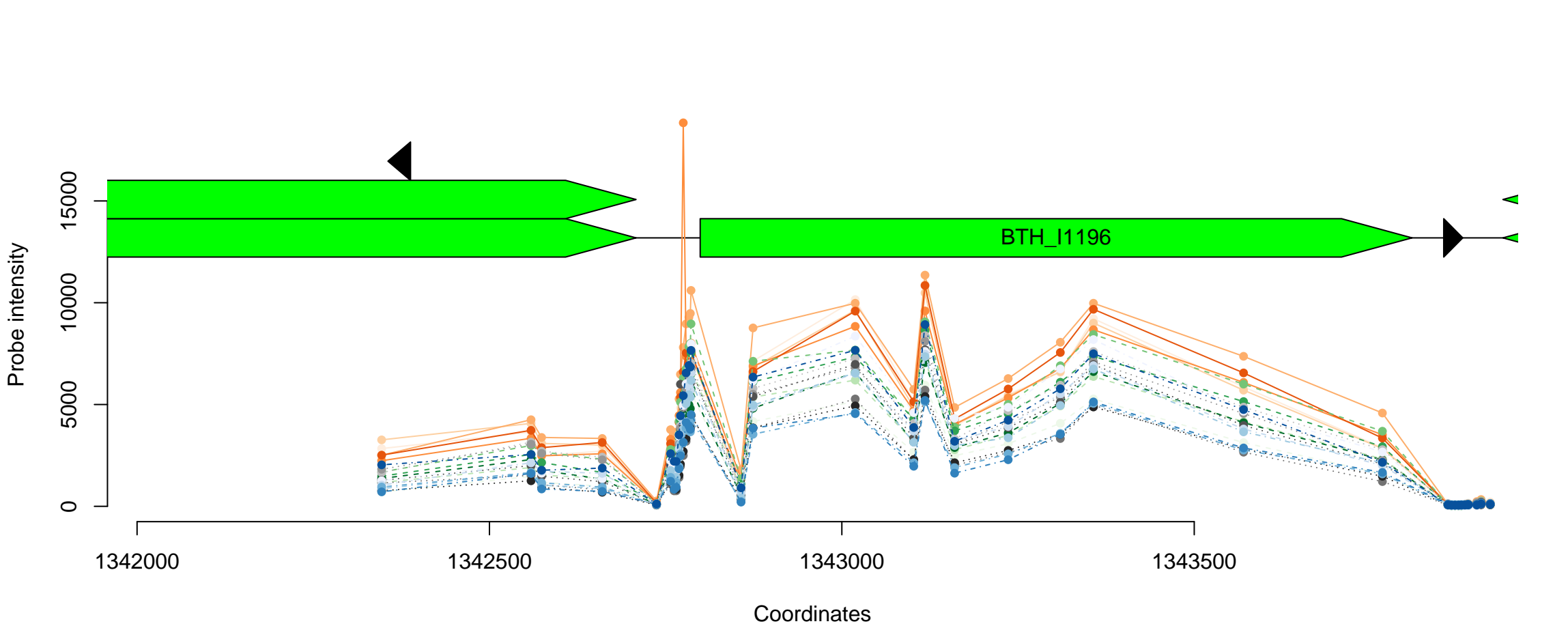

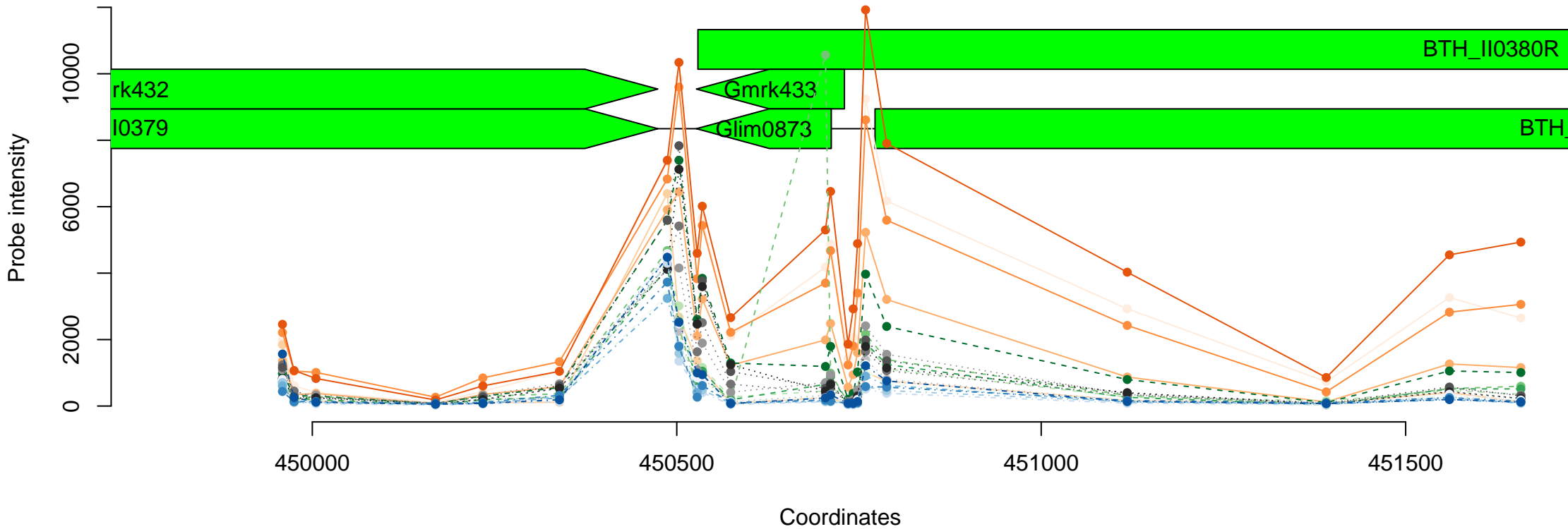

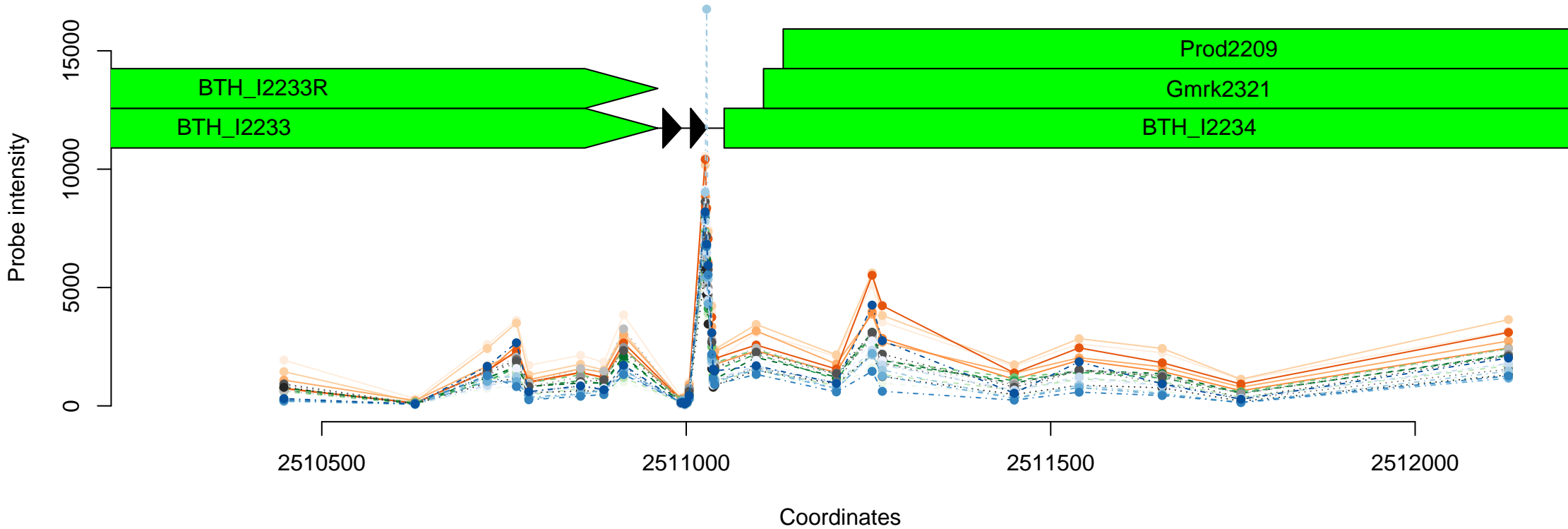

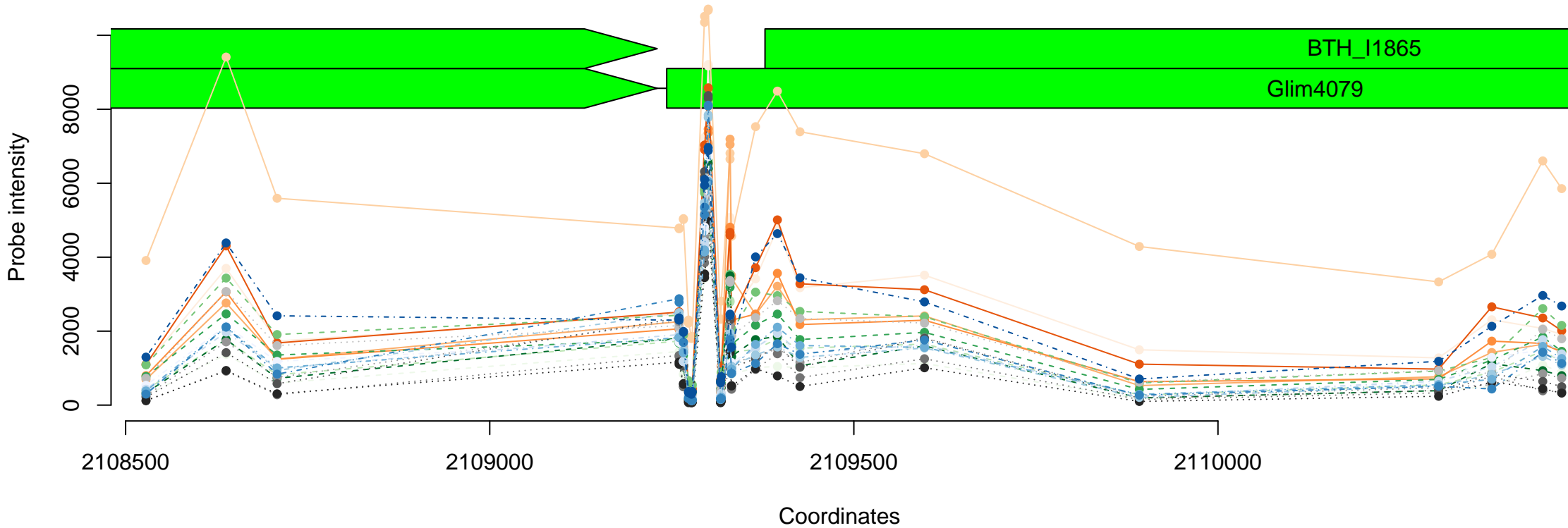

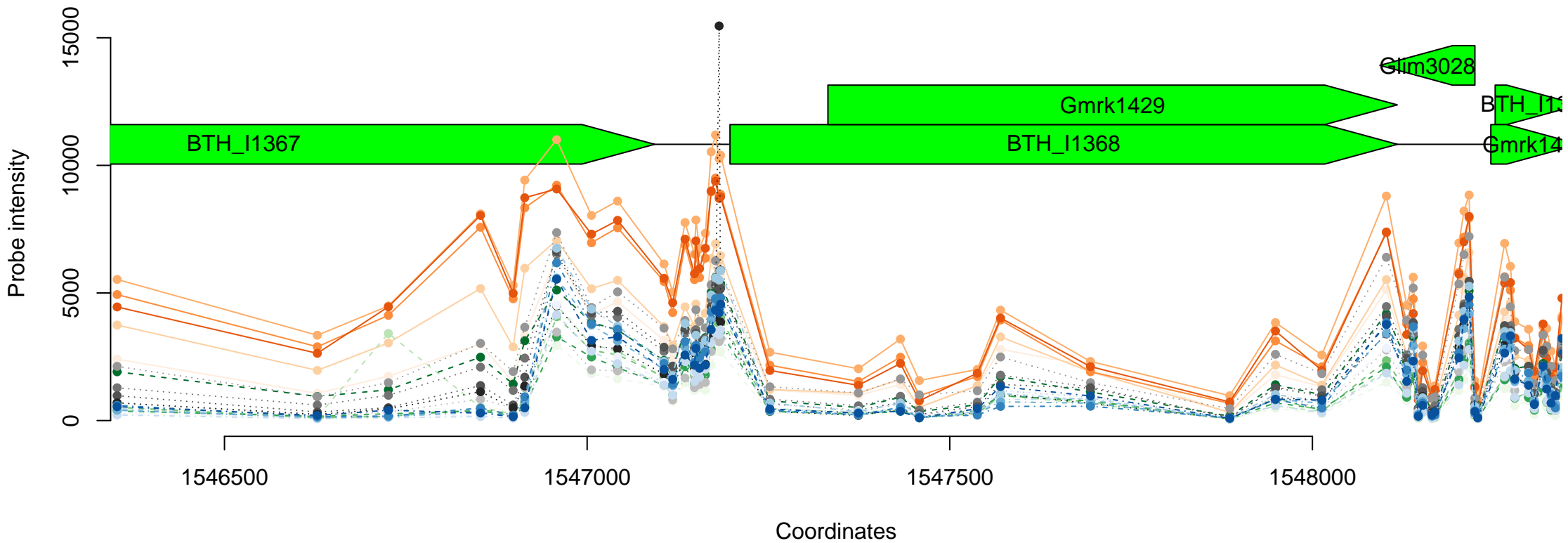

Probe intensity

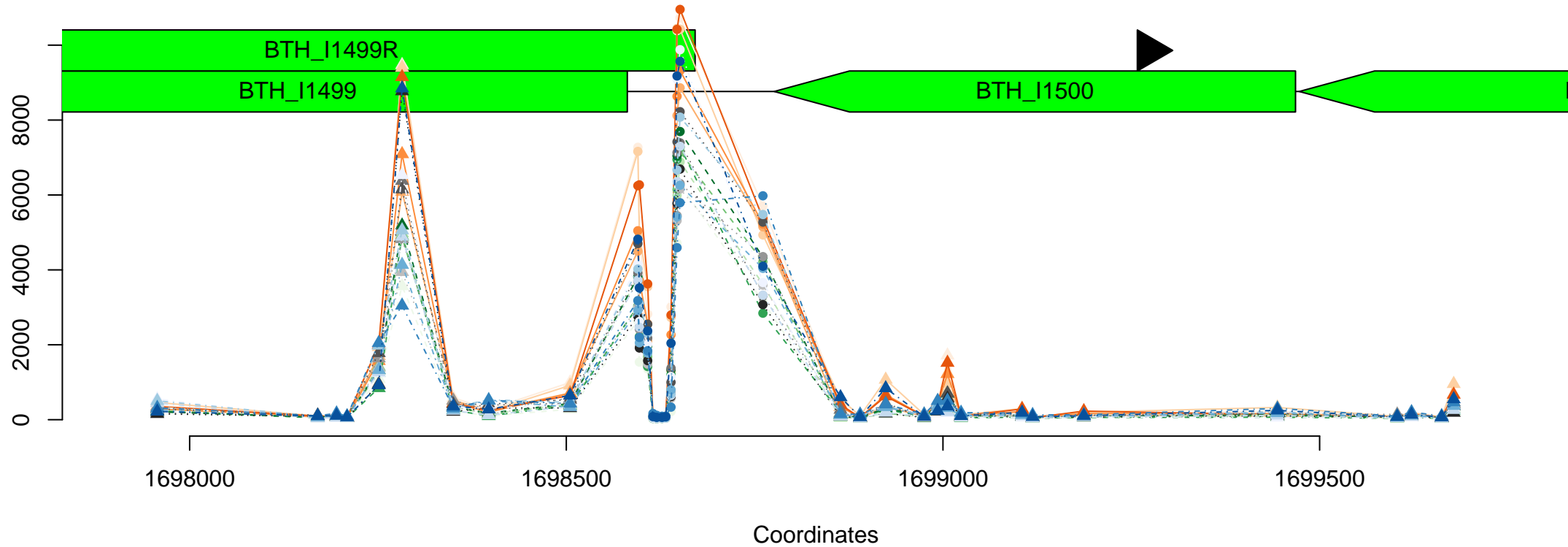

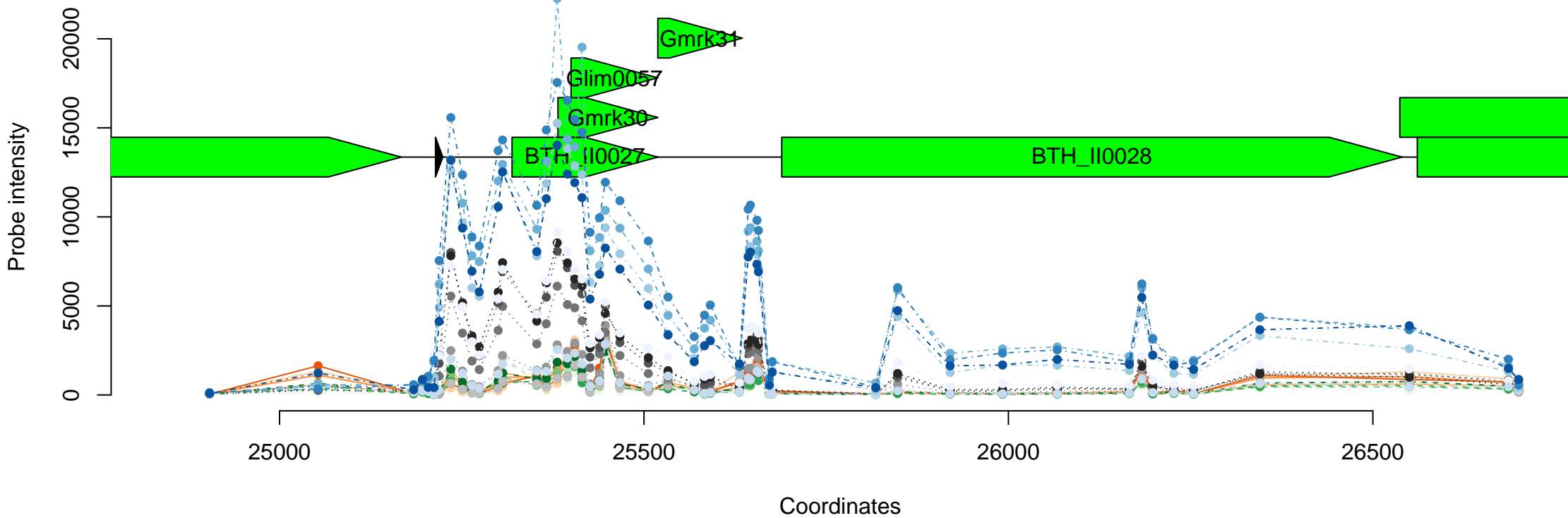

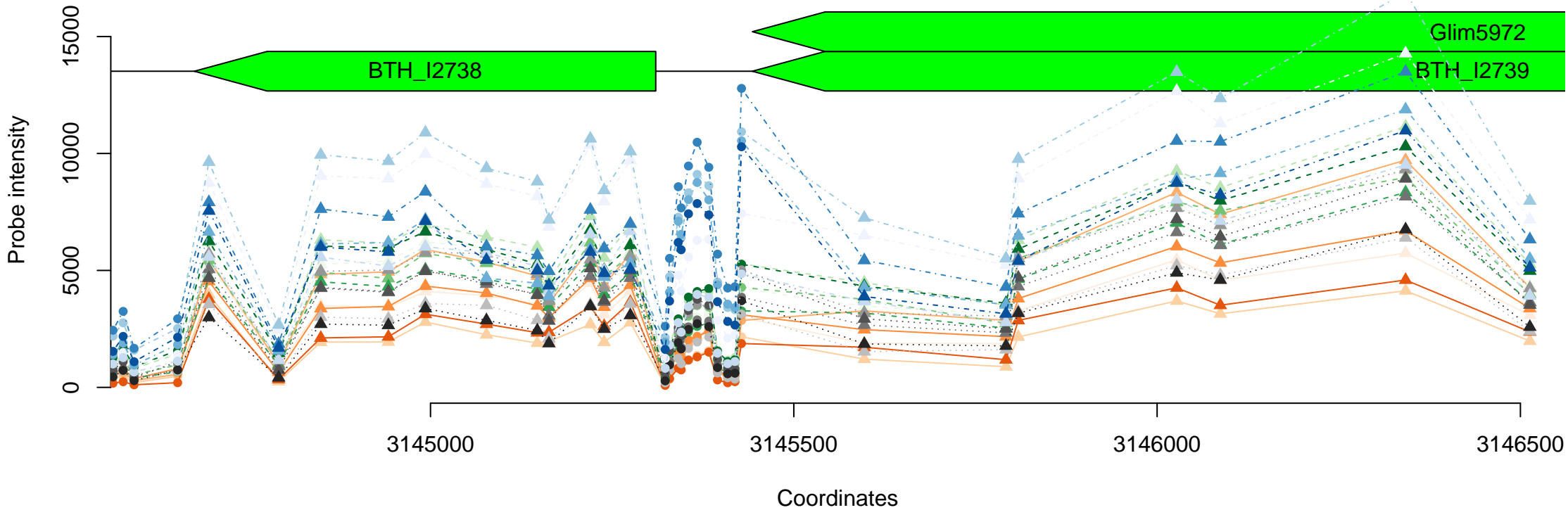

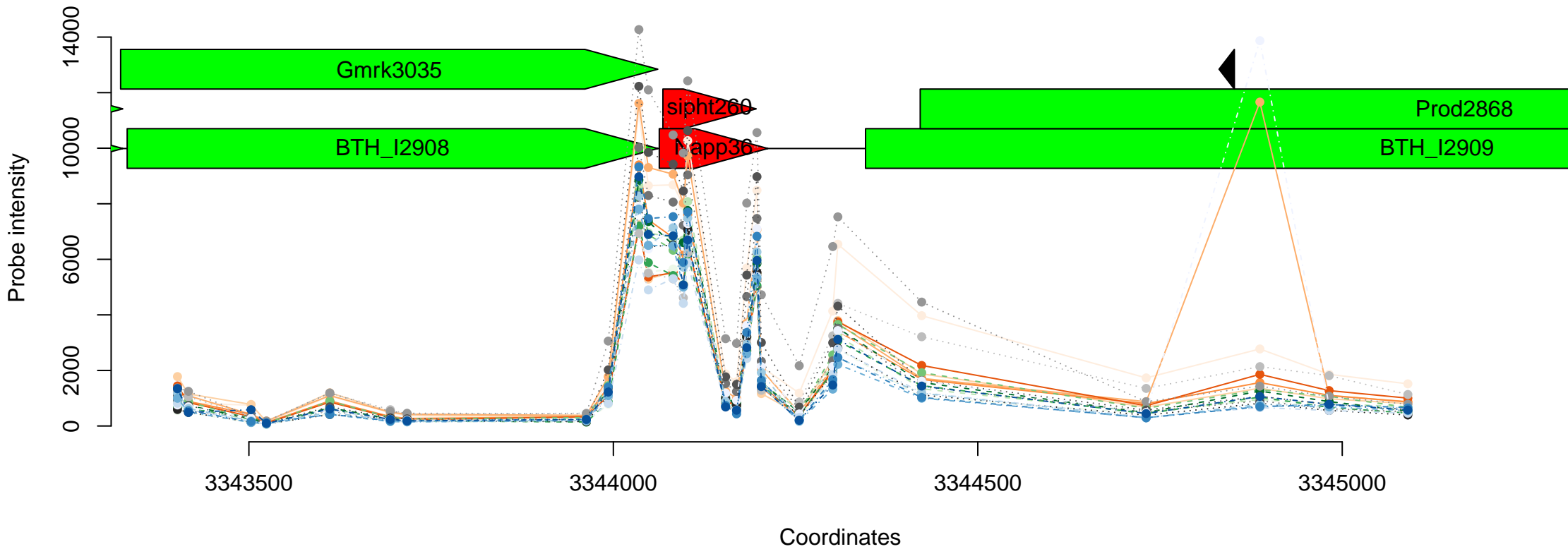

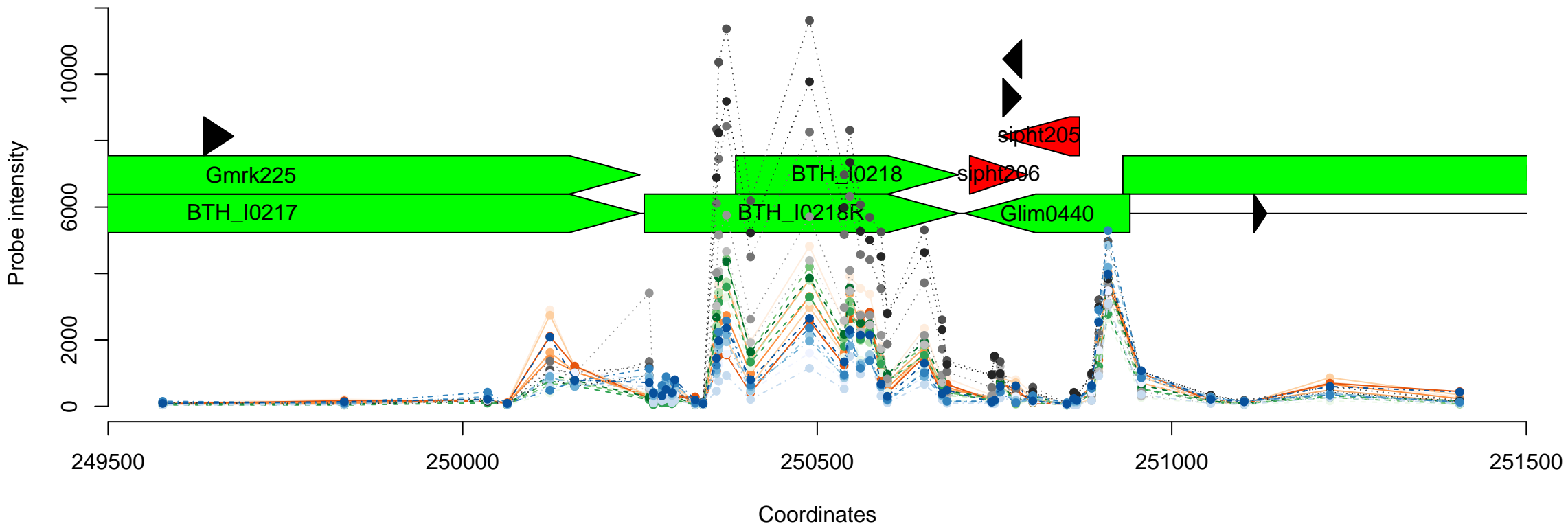

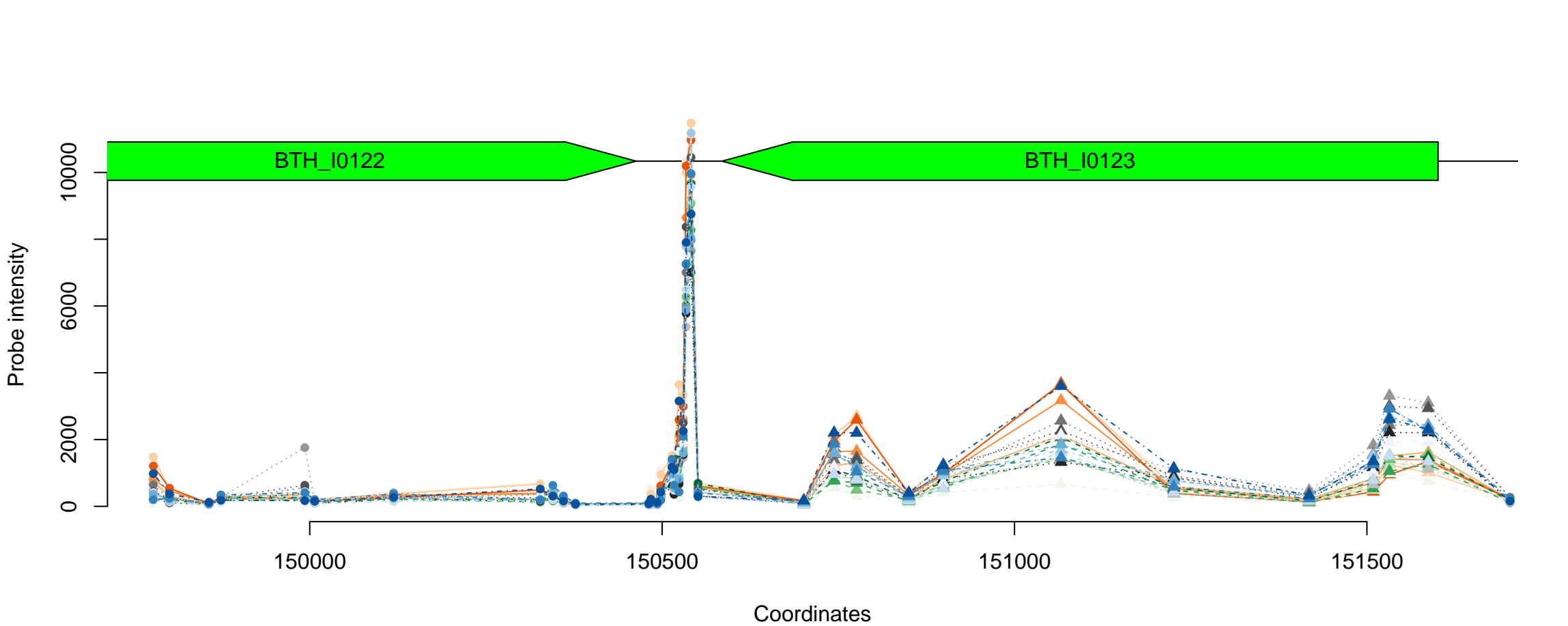

Probe intensity

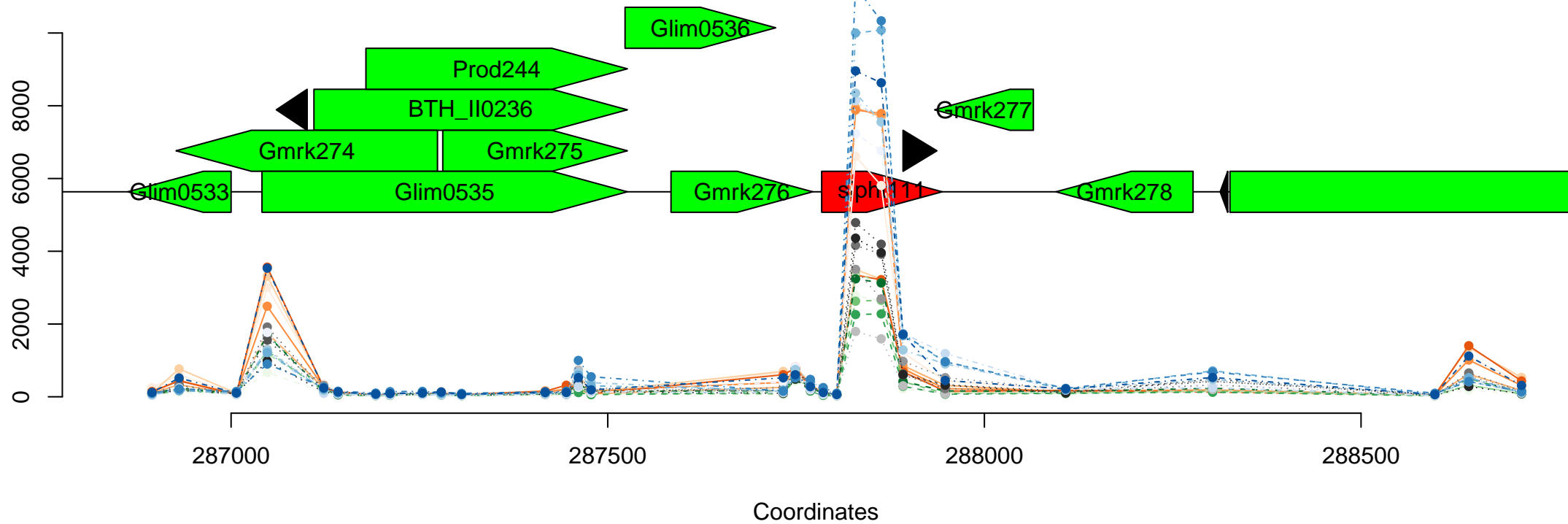

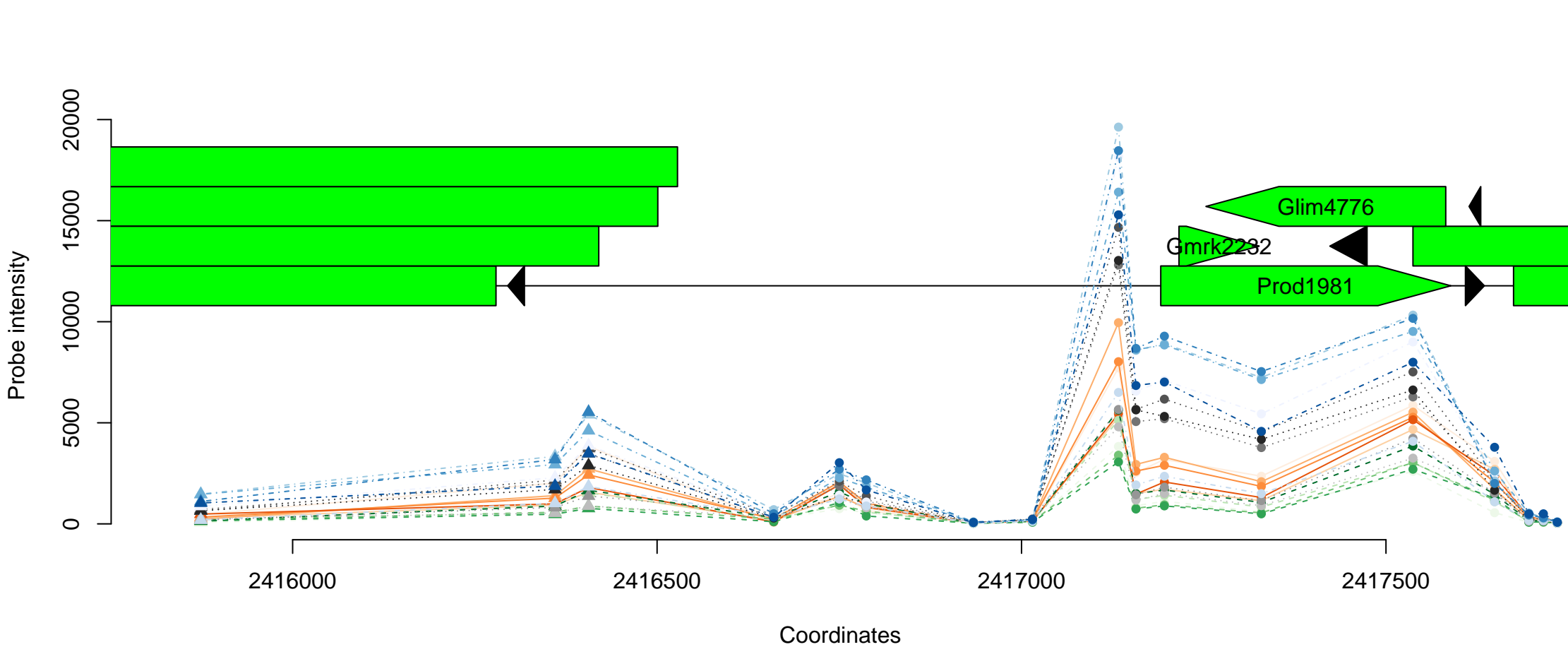

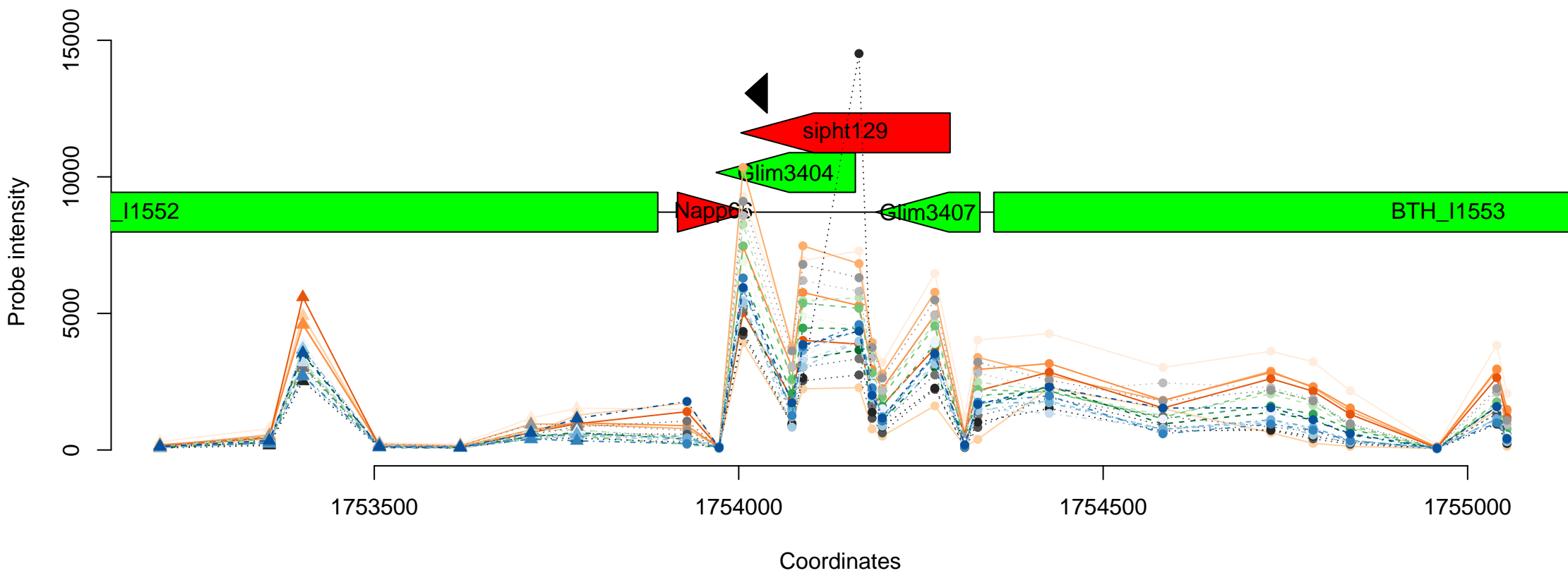

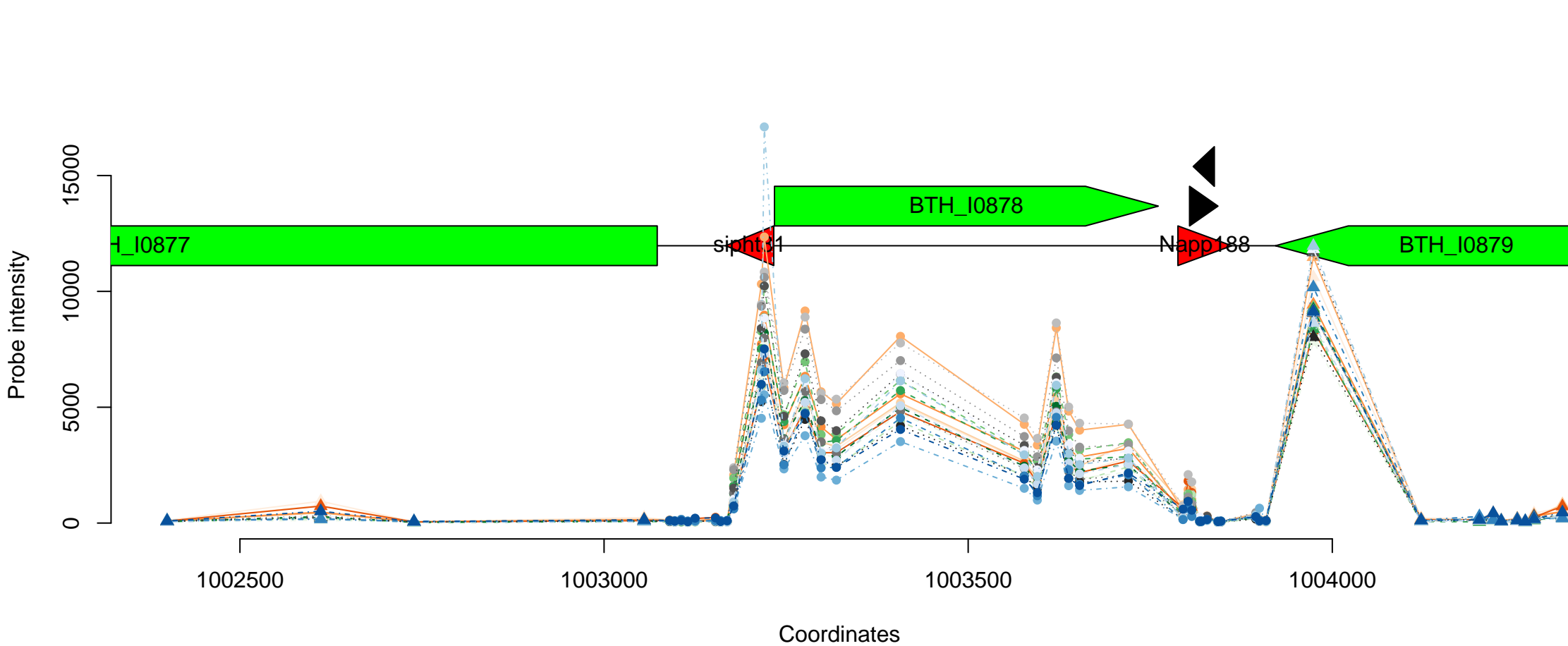

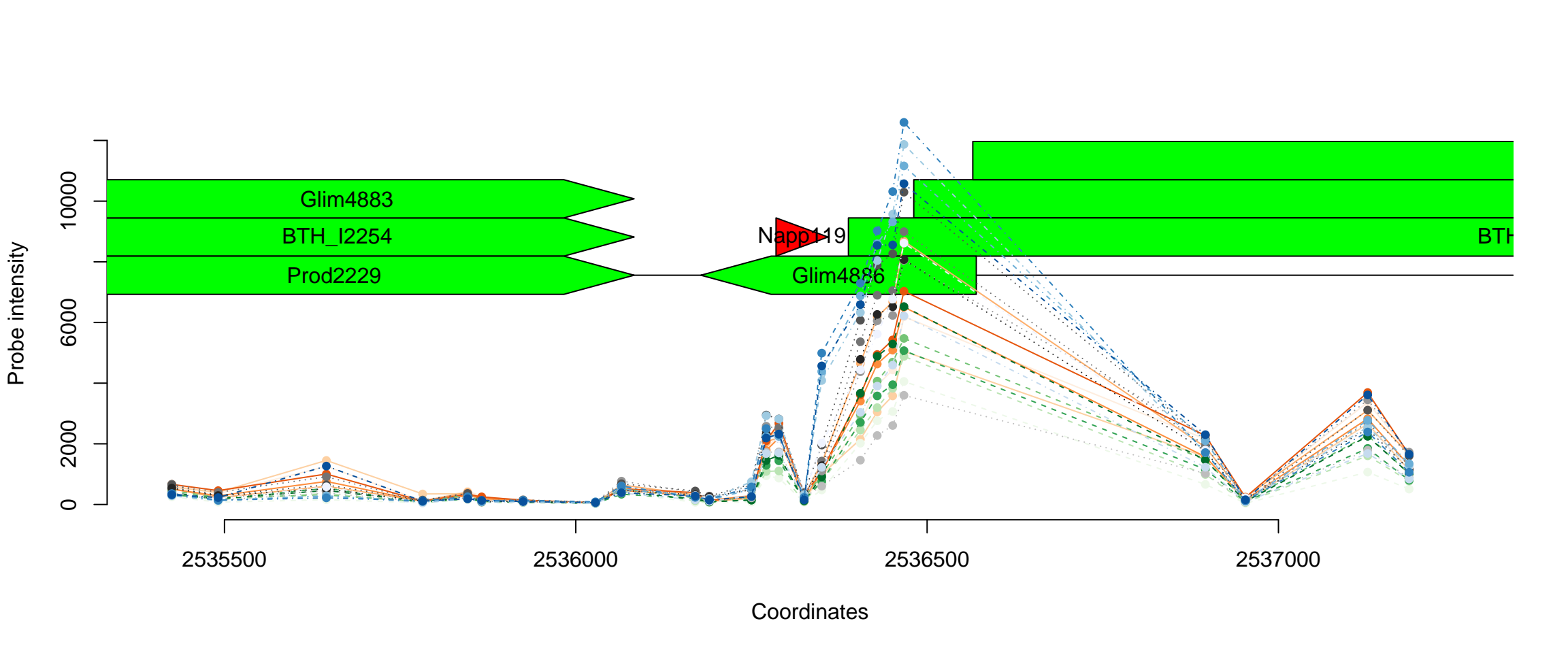

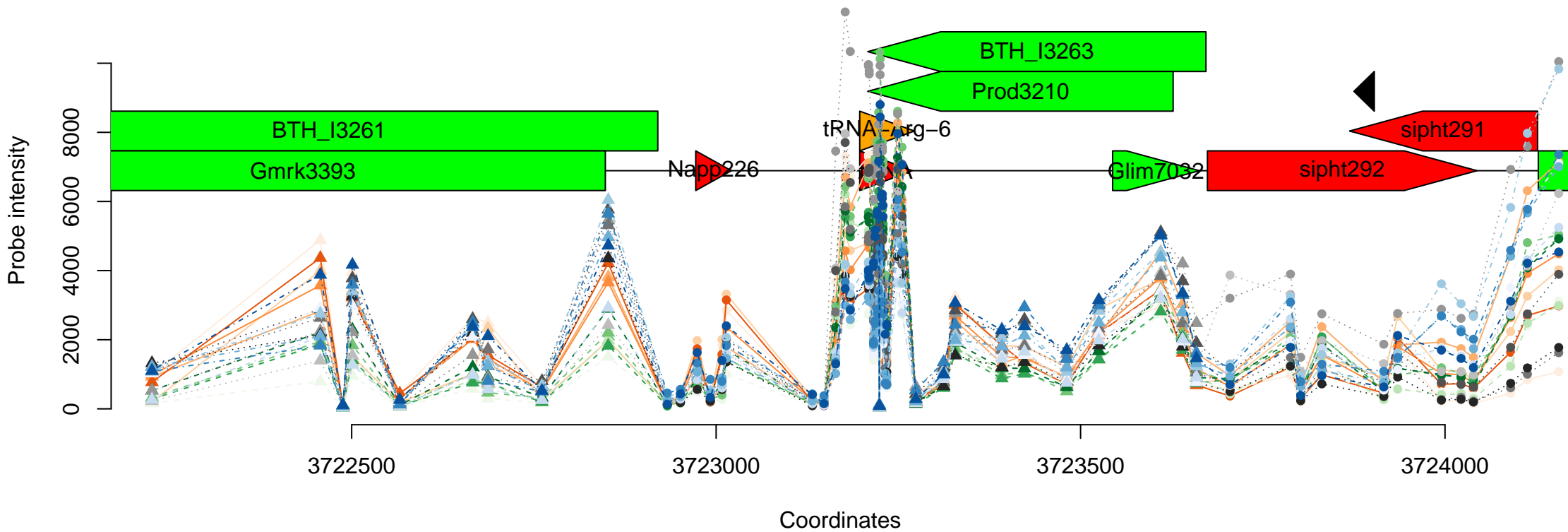

Supplement: Supplementary file 1 — Additional file 1: Expression plots from IGRs. The top 100 IGRs were sorted by the range between the probe with the lowest intensity and the probe with the second highest intensity using probe data from the 21 distinct conditions in the four time course arrays. Plot colors and labels are described in the Methods. (PDF 7 MB) [file 12864_2013_6069_MOESM1_ESM.pdf]

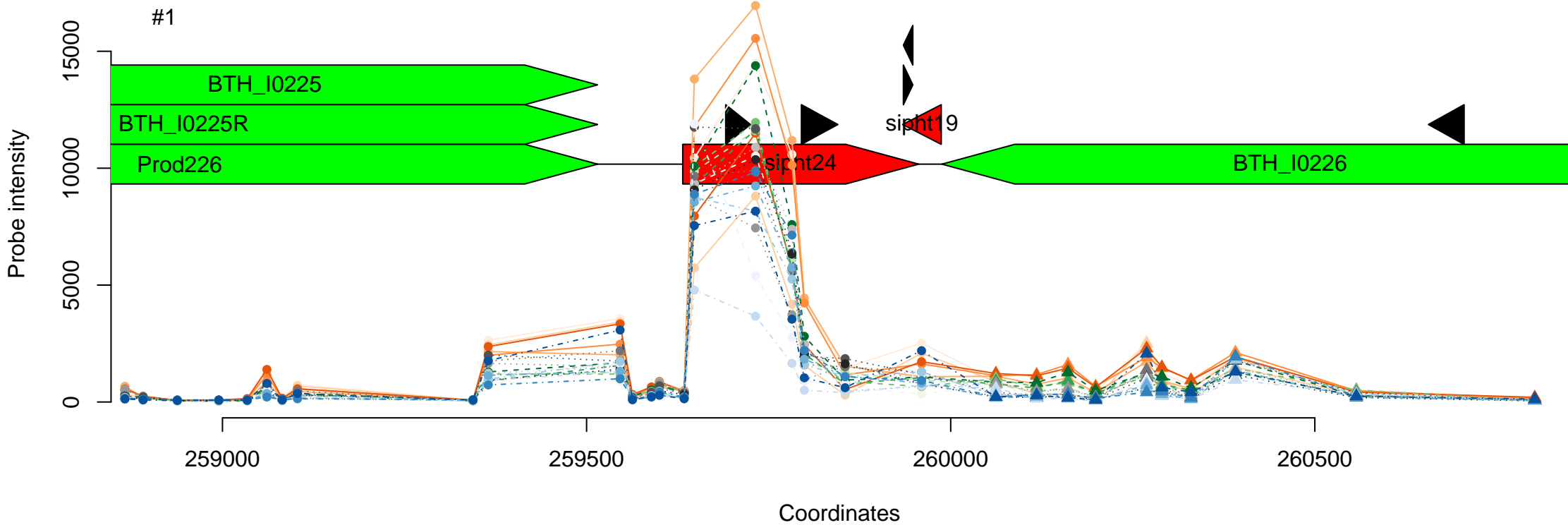

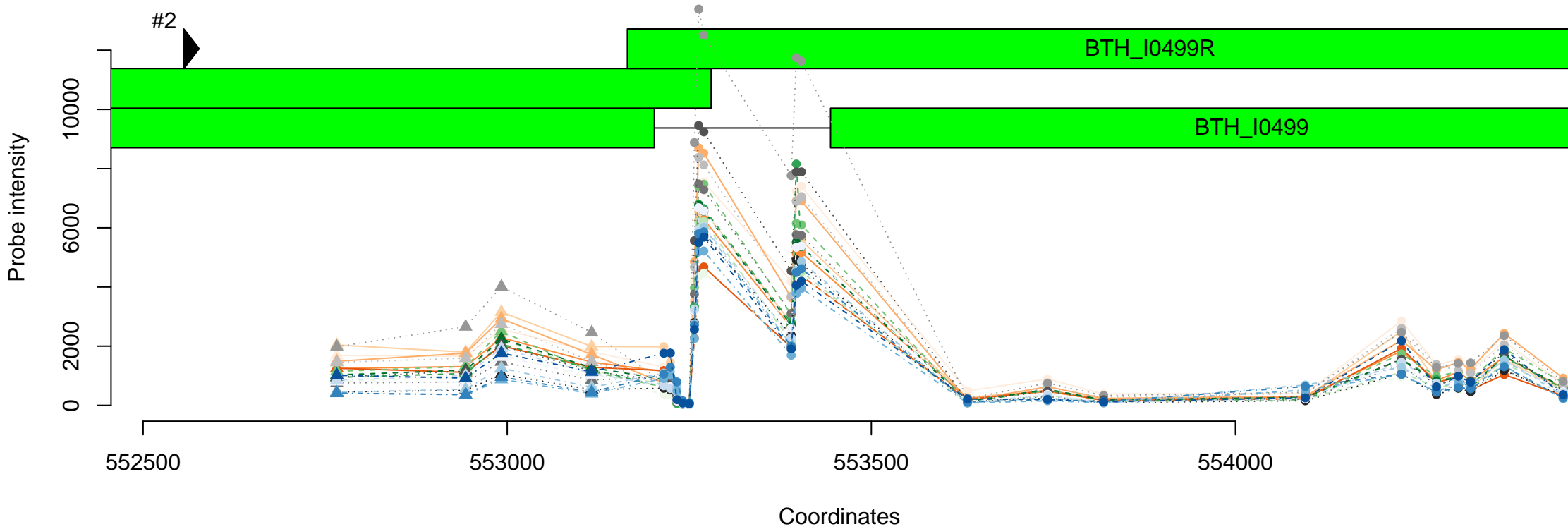

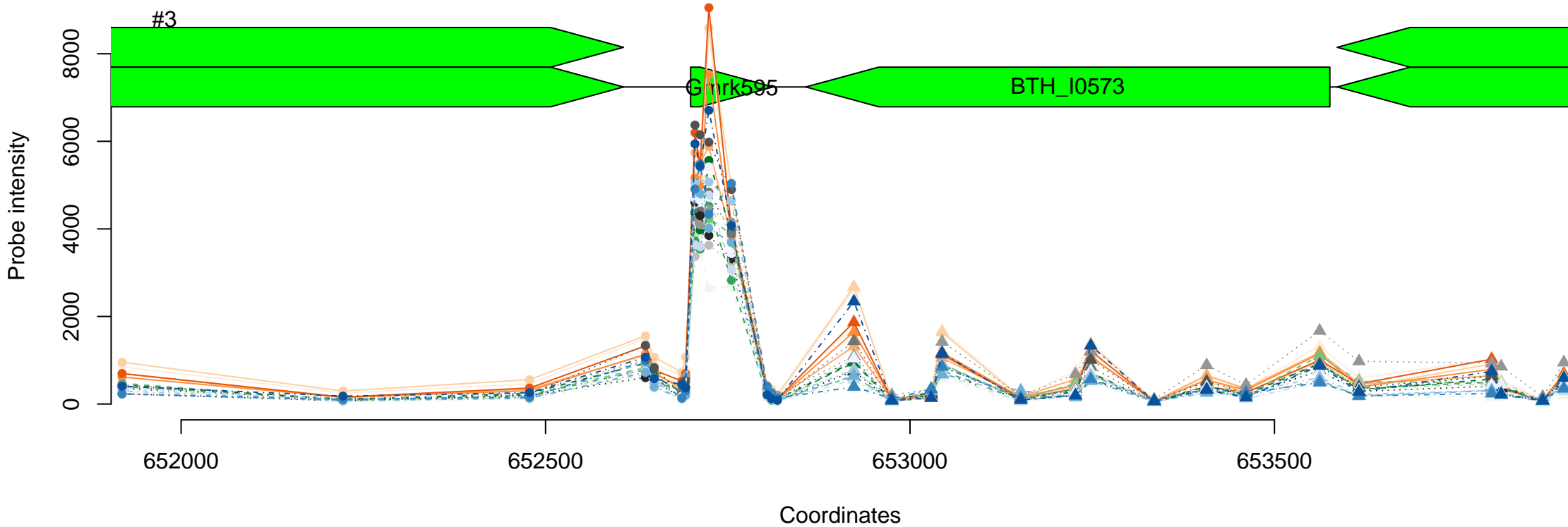

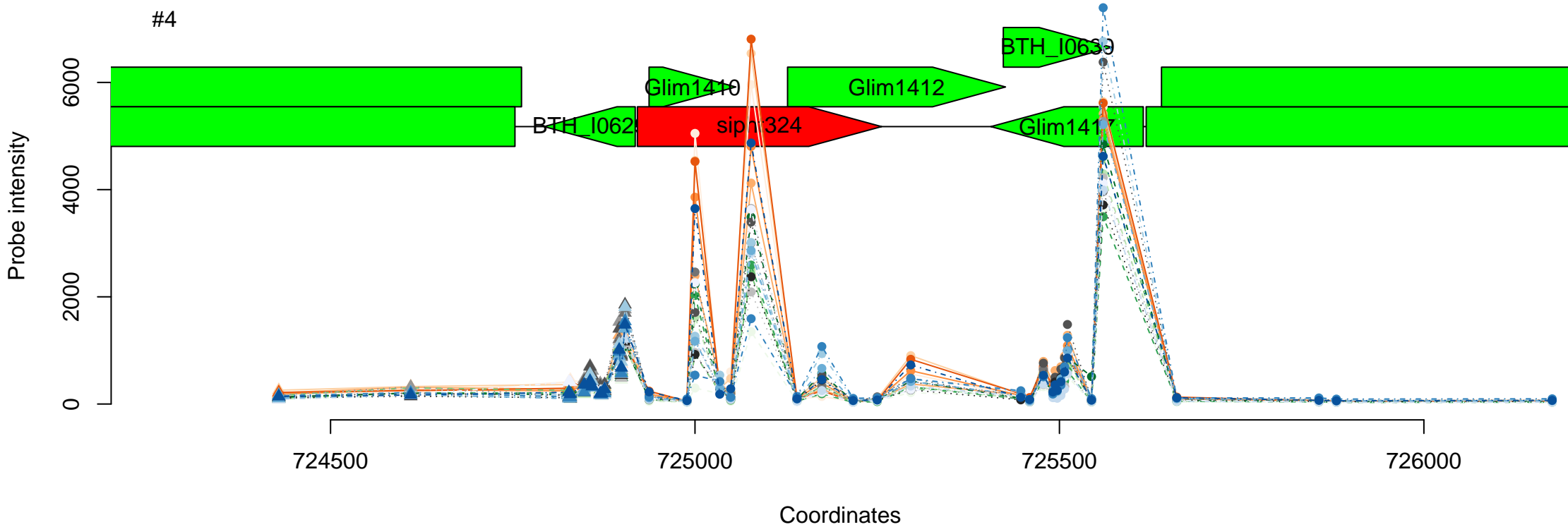

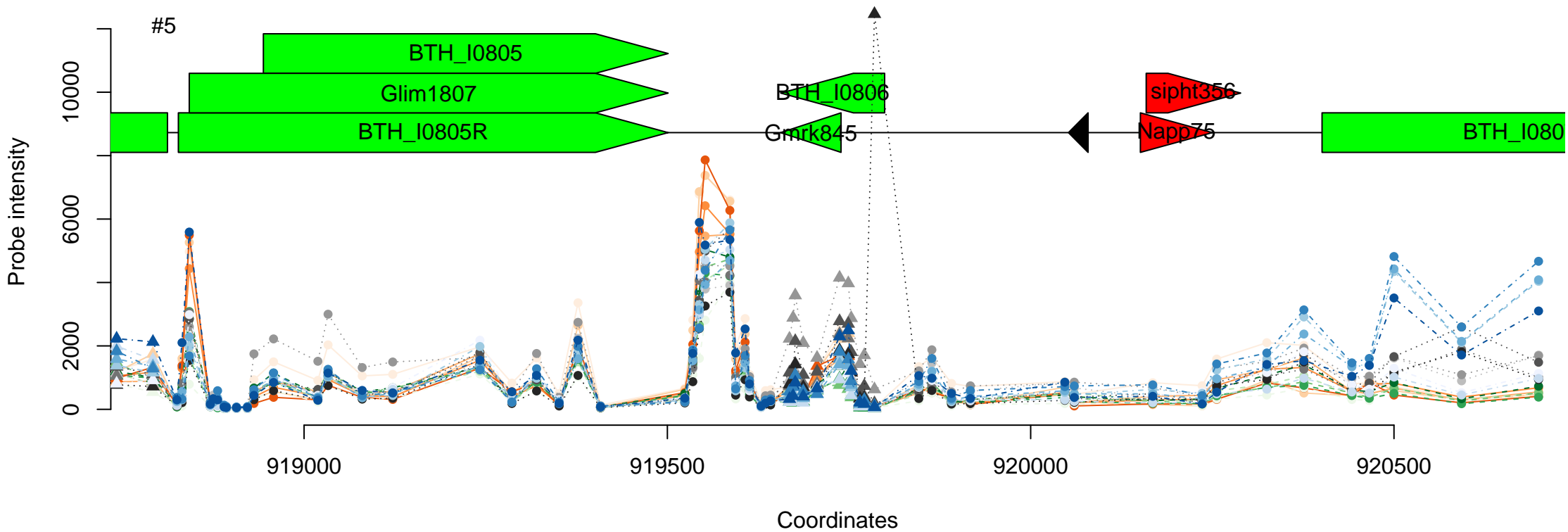

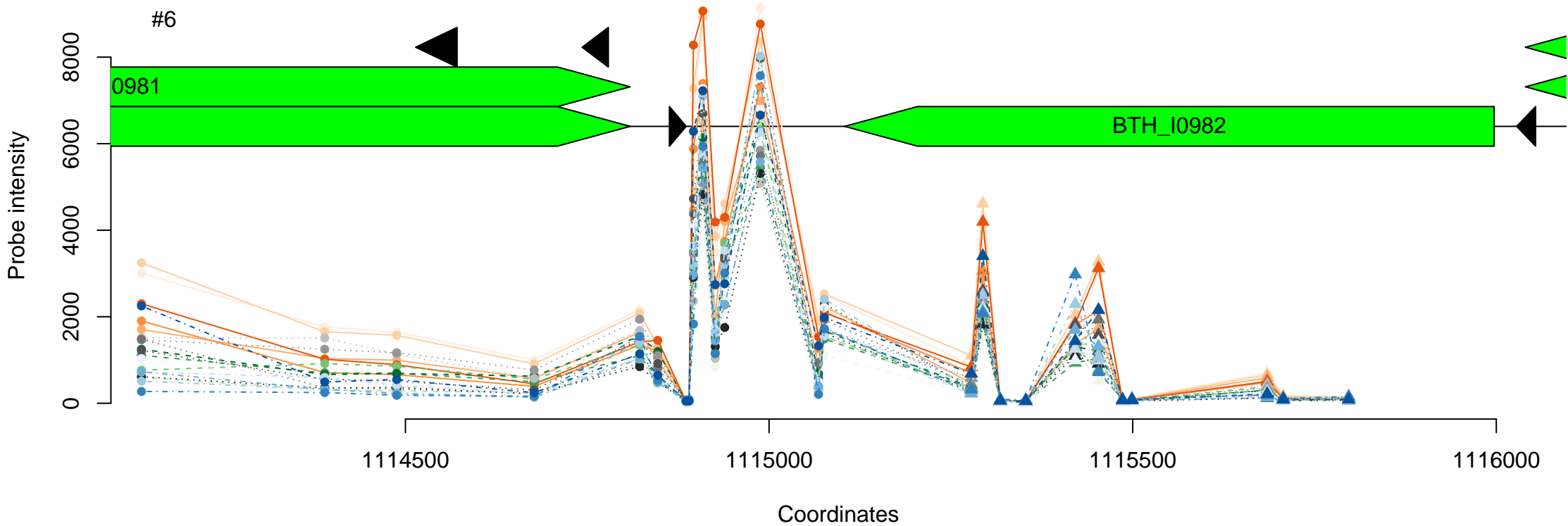

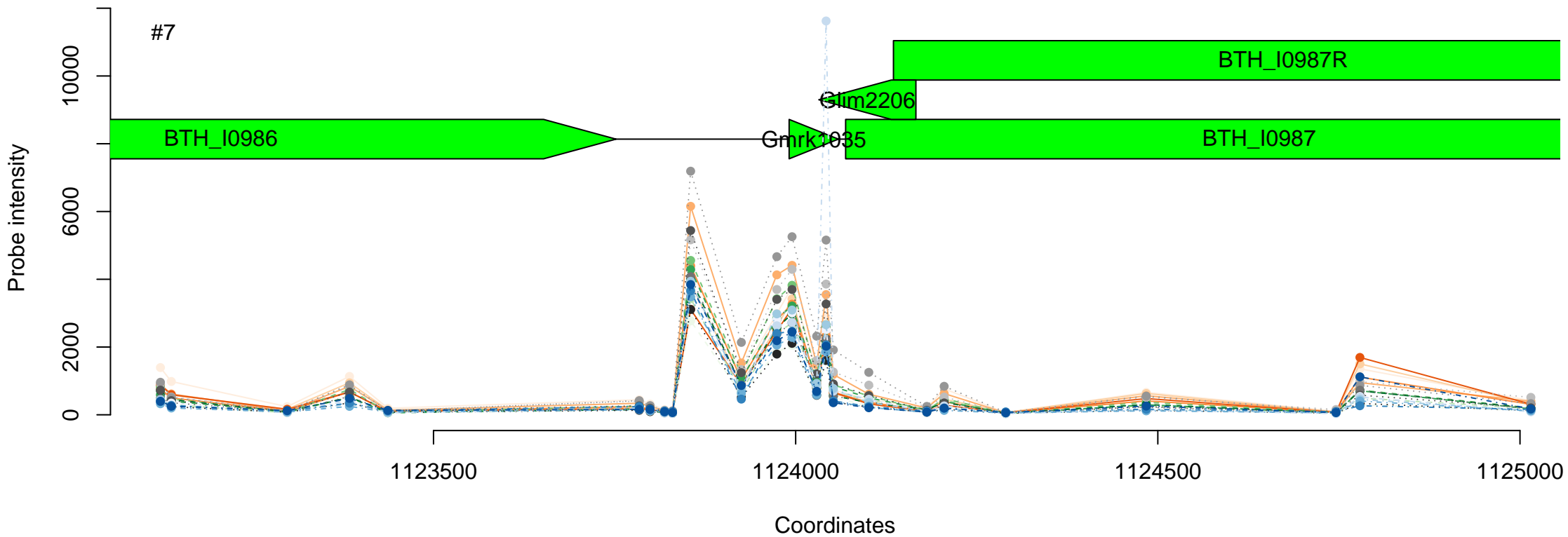

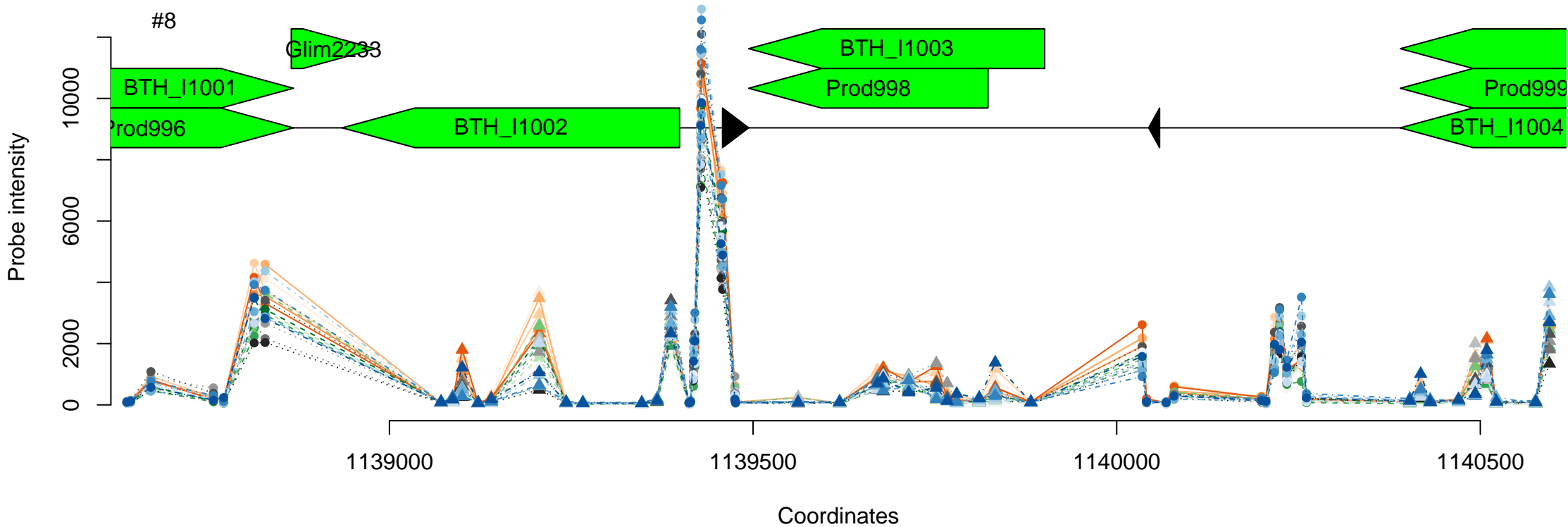

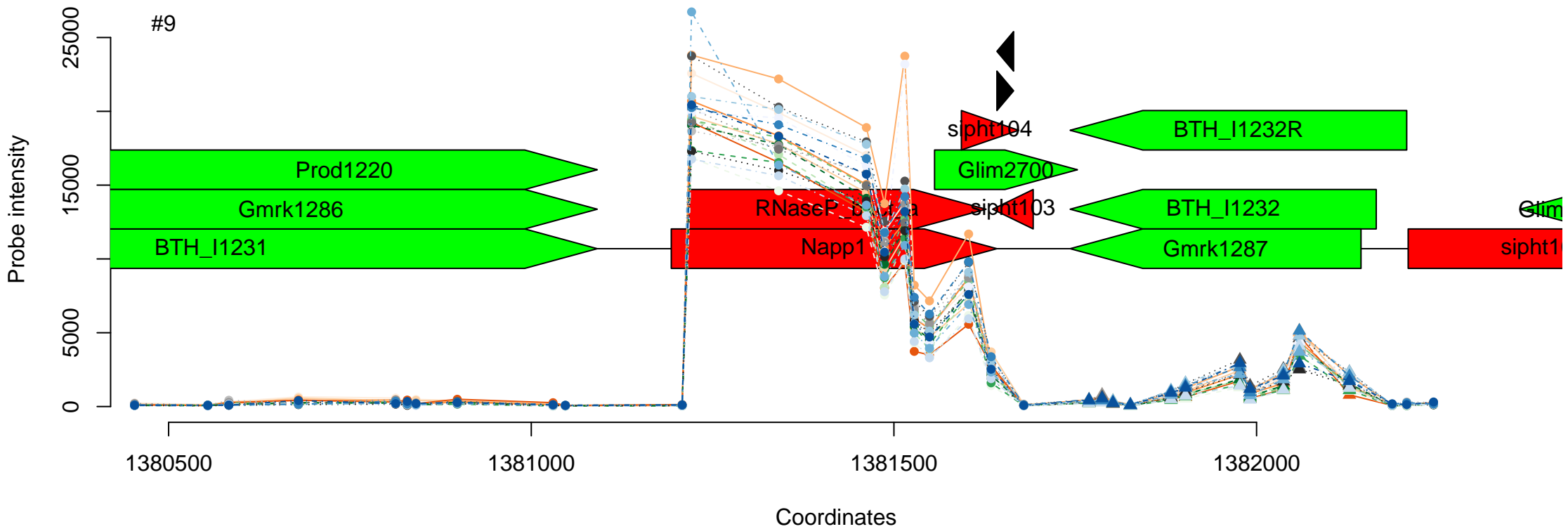

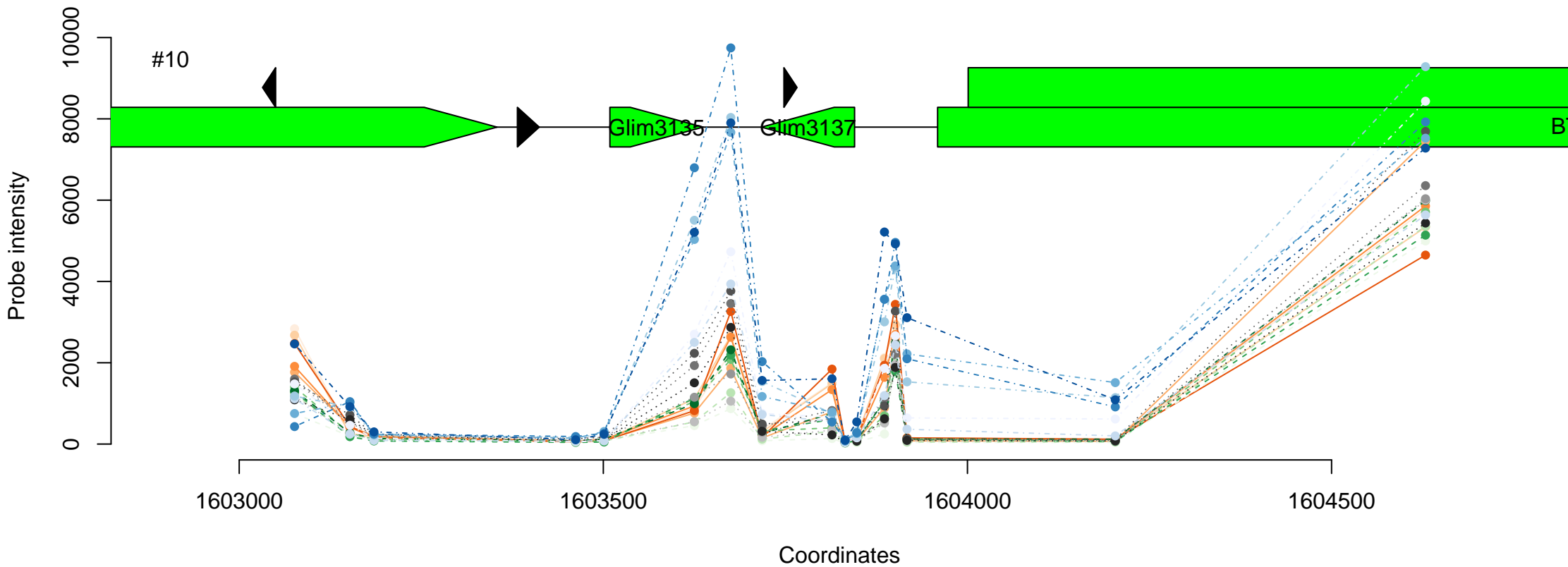

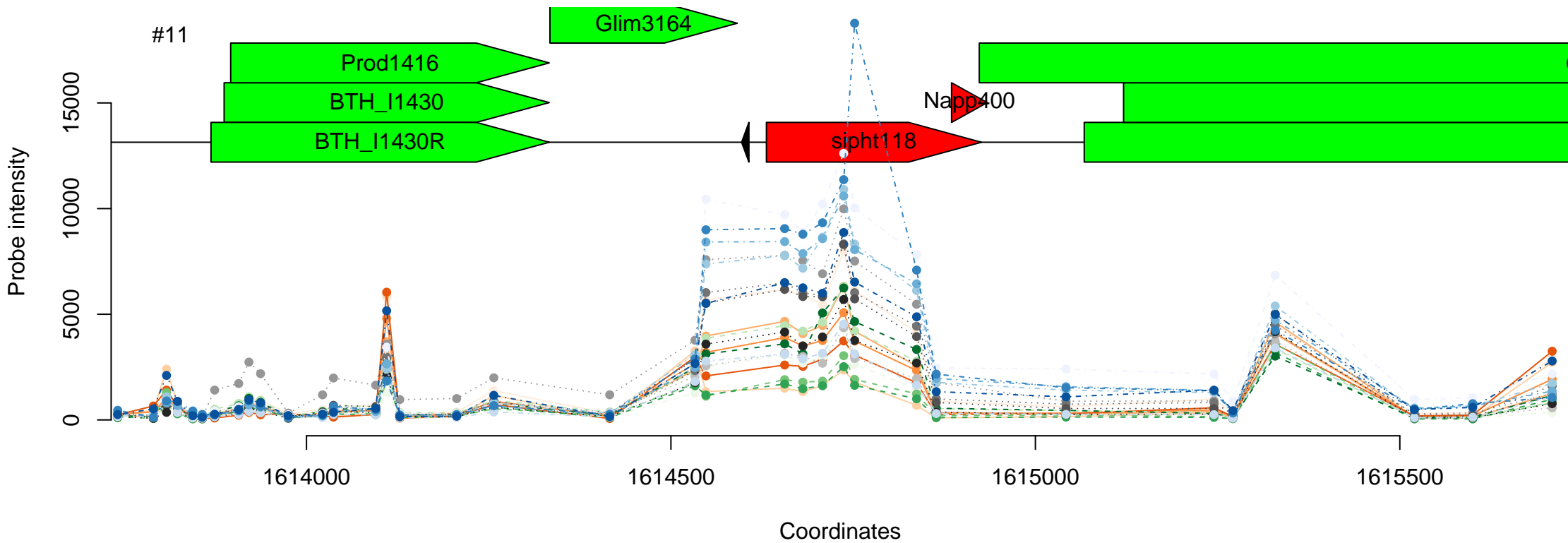

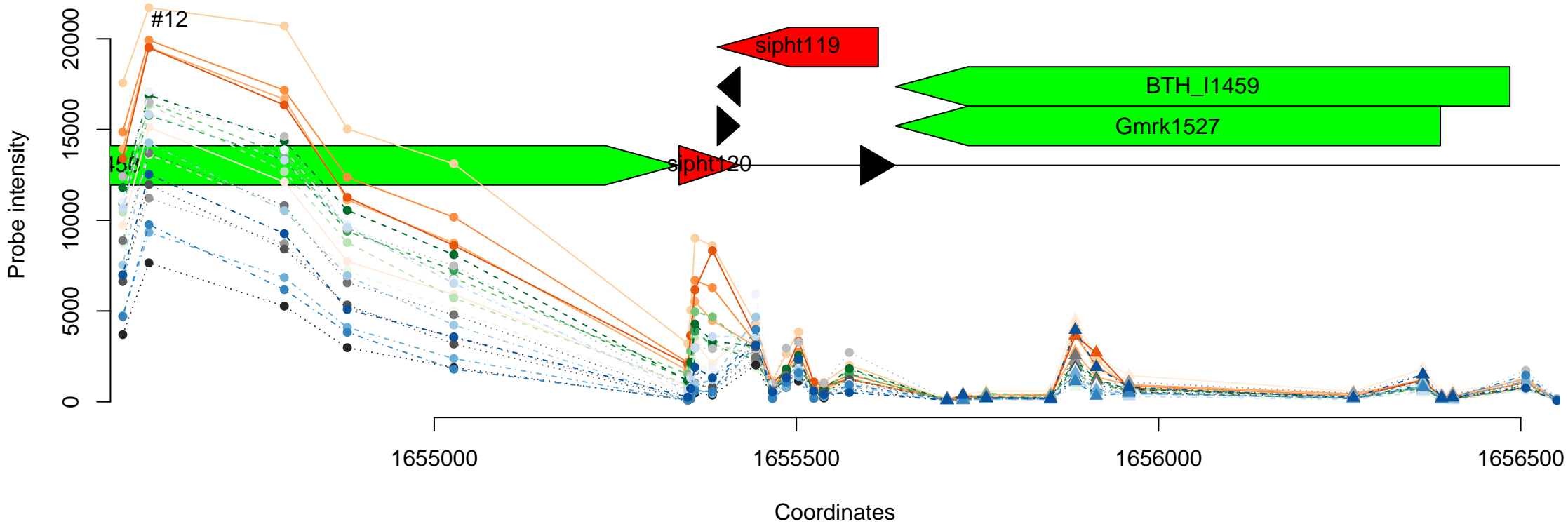

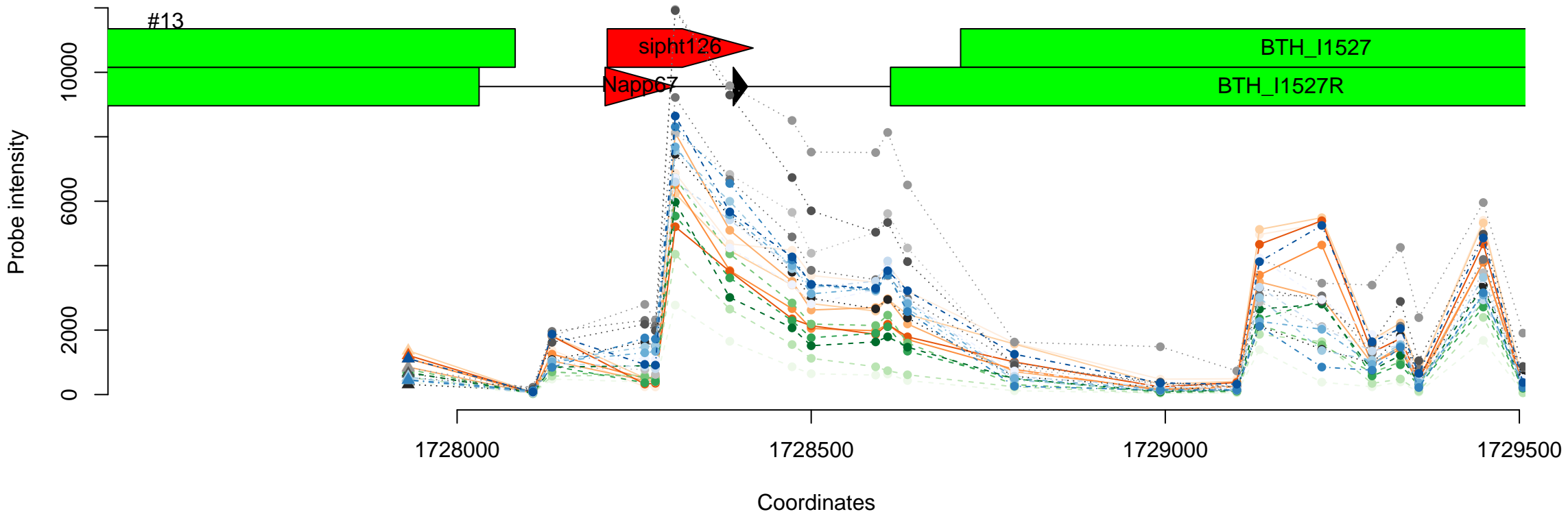

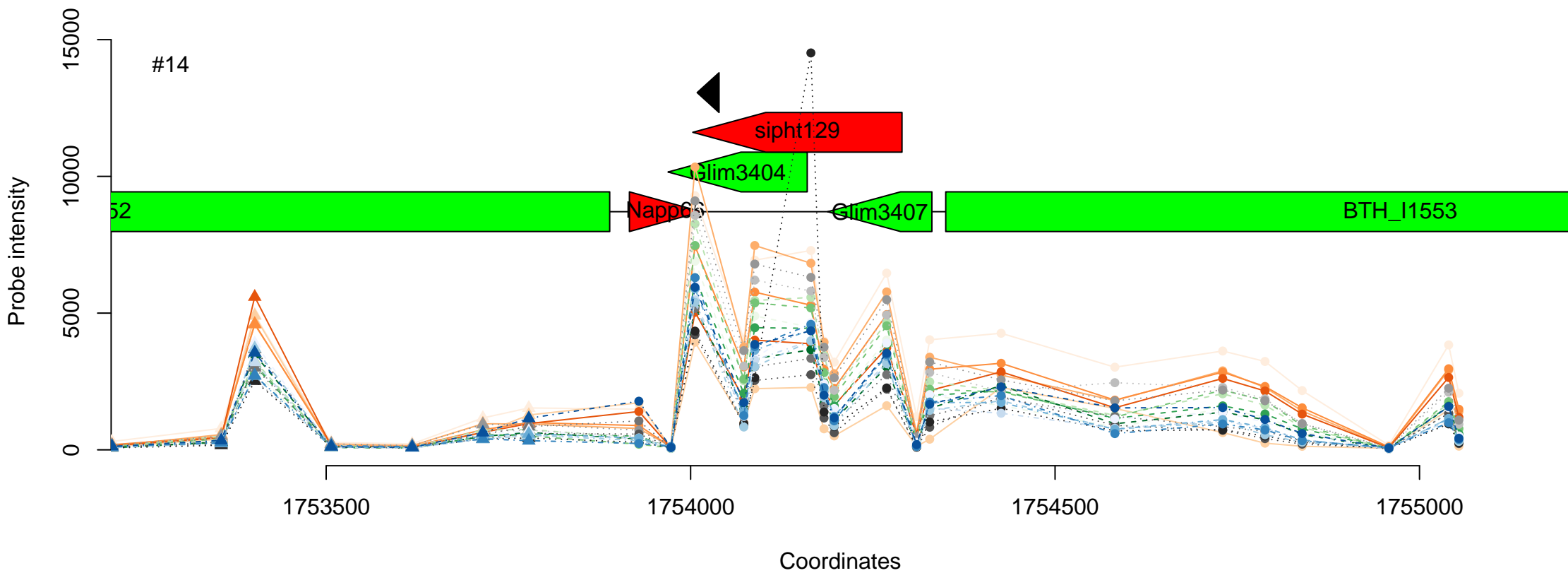

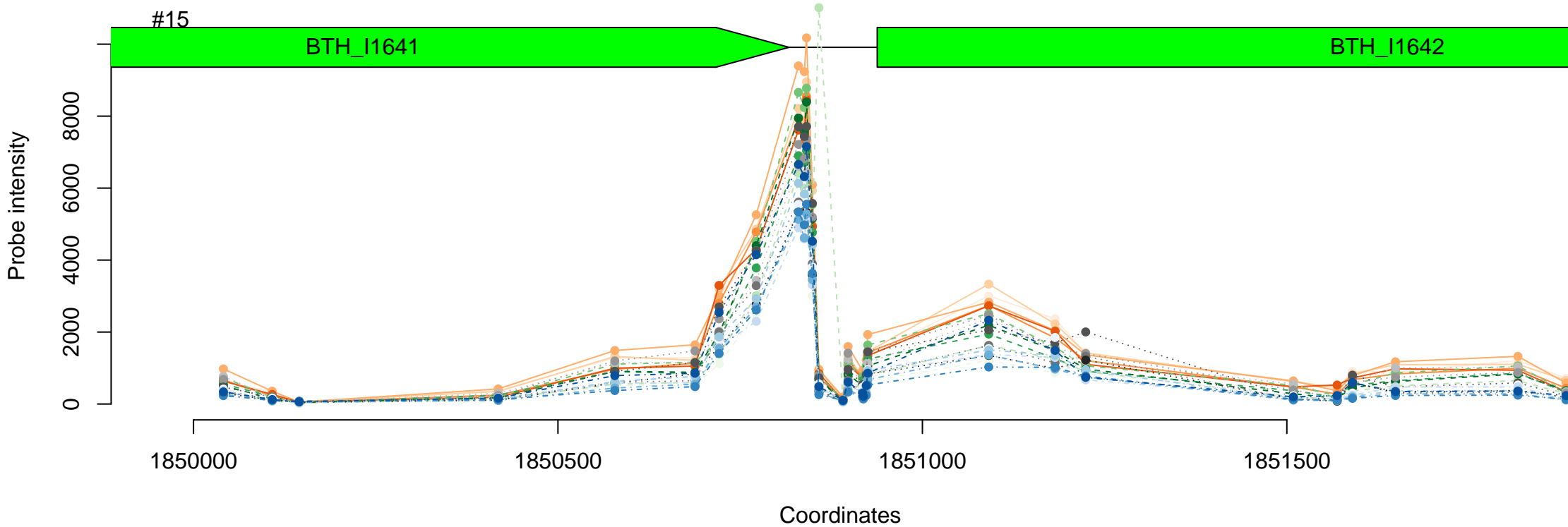

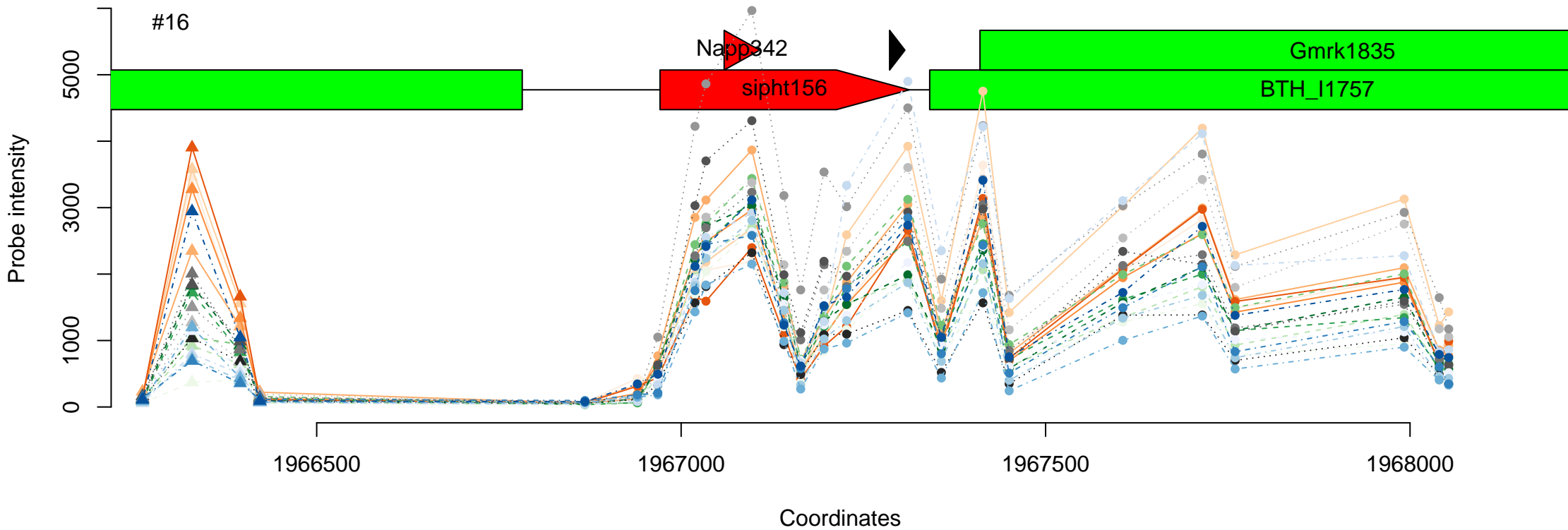

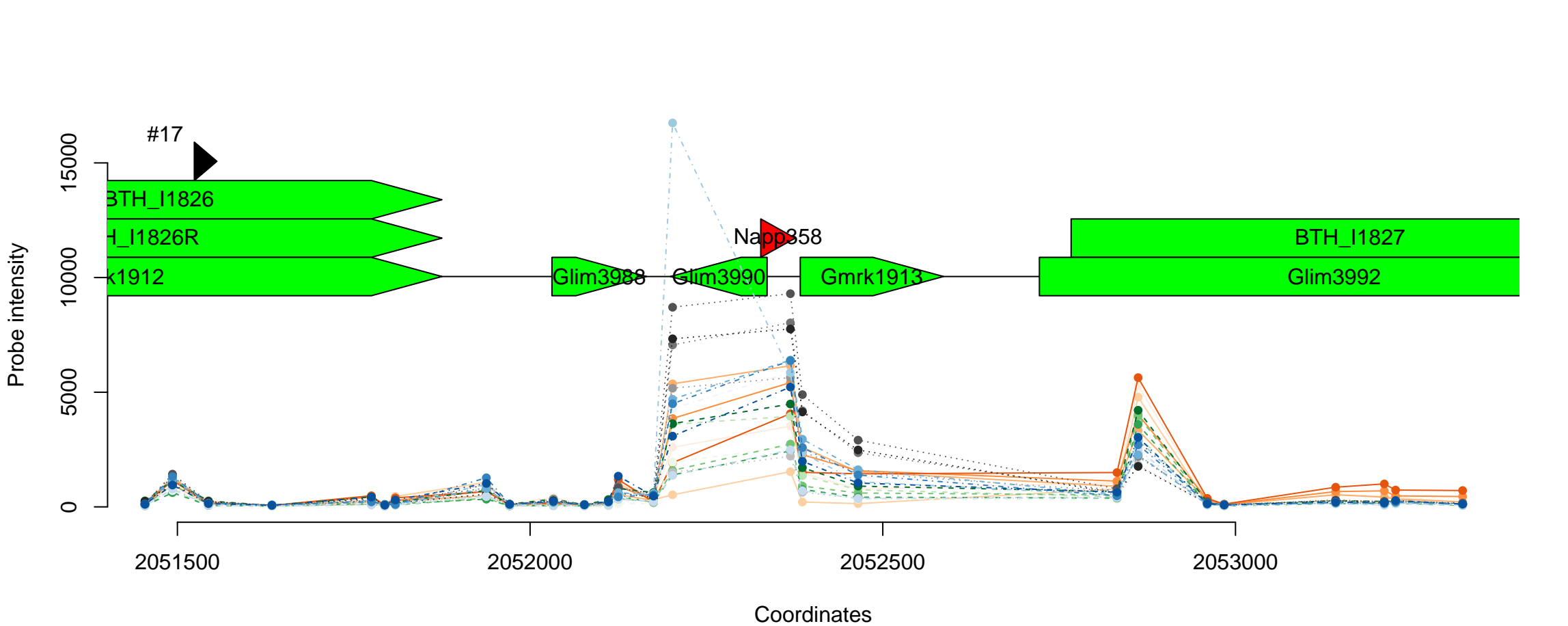

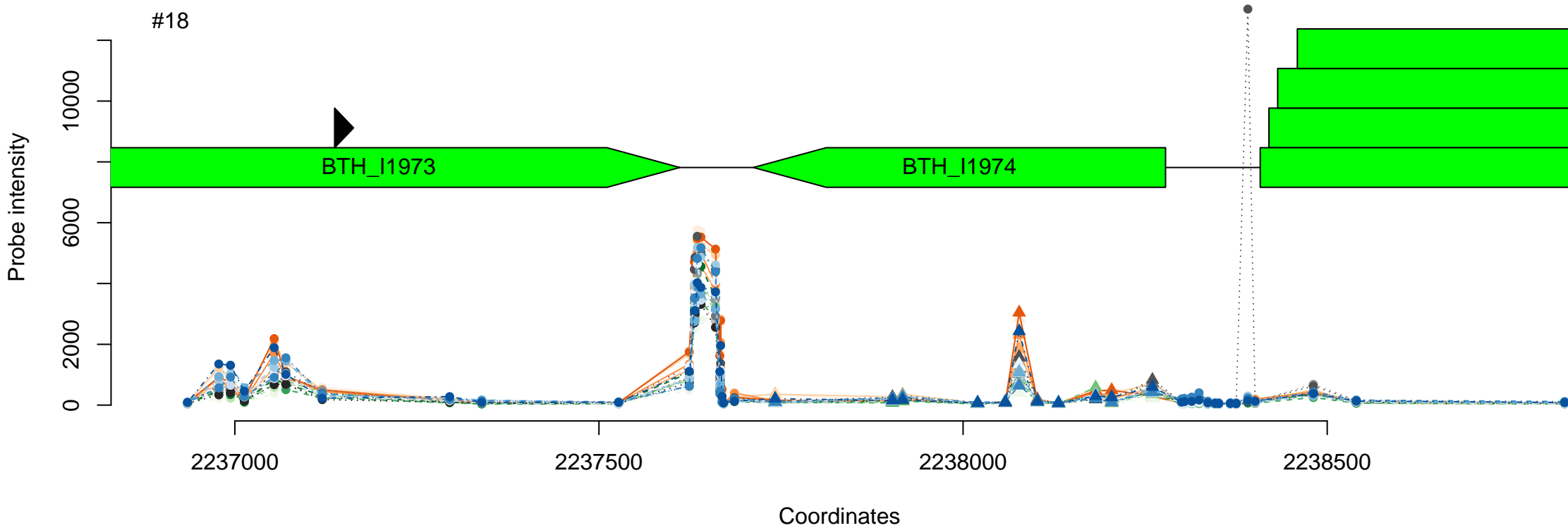

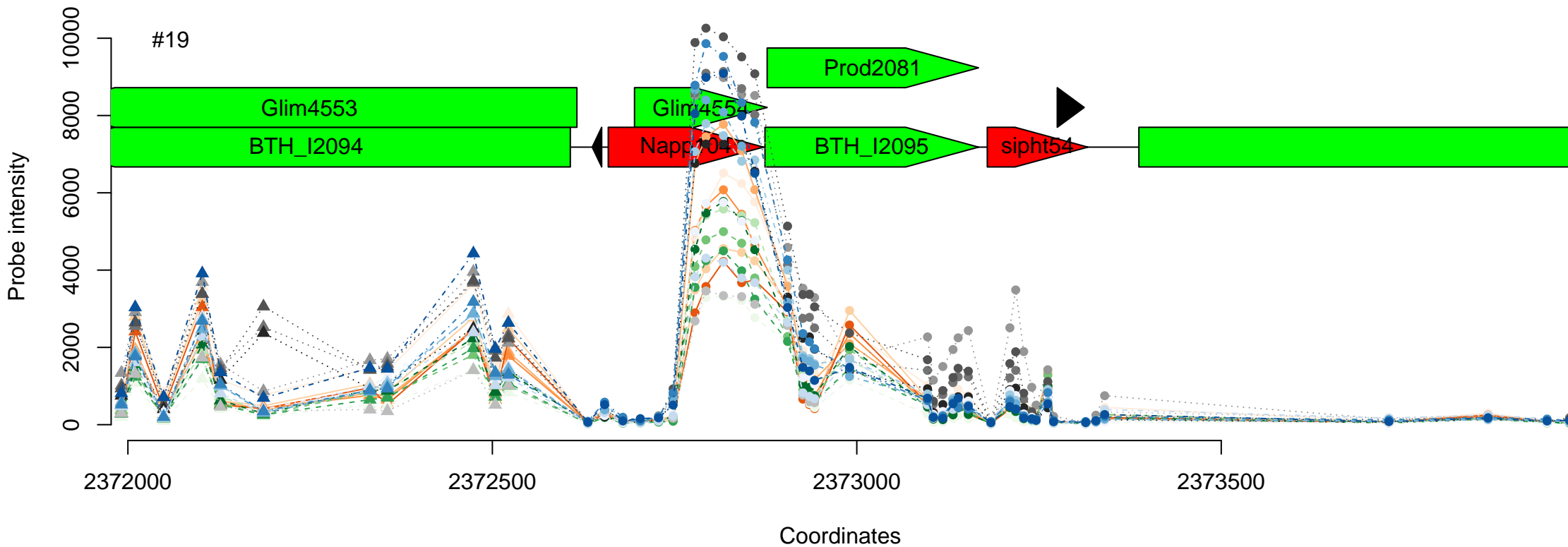

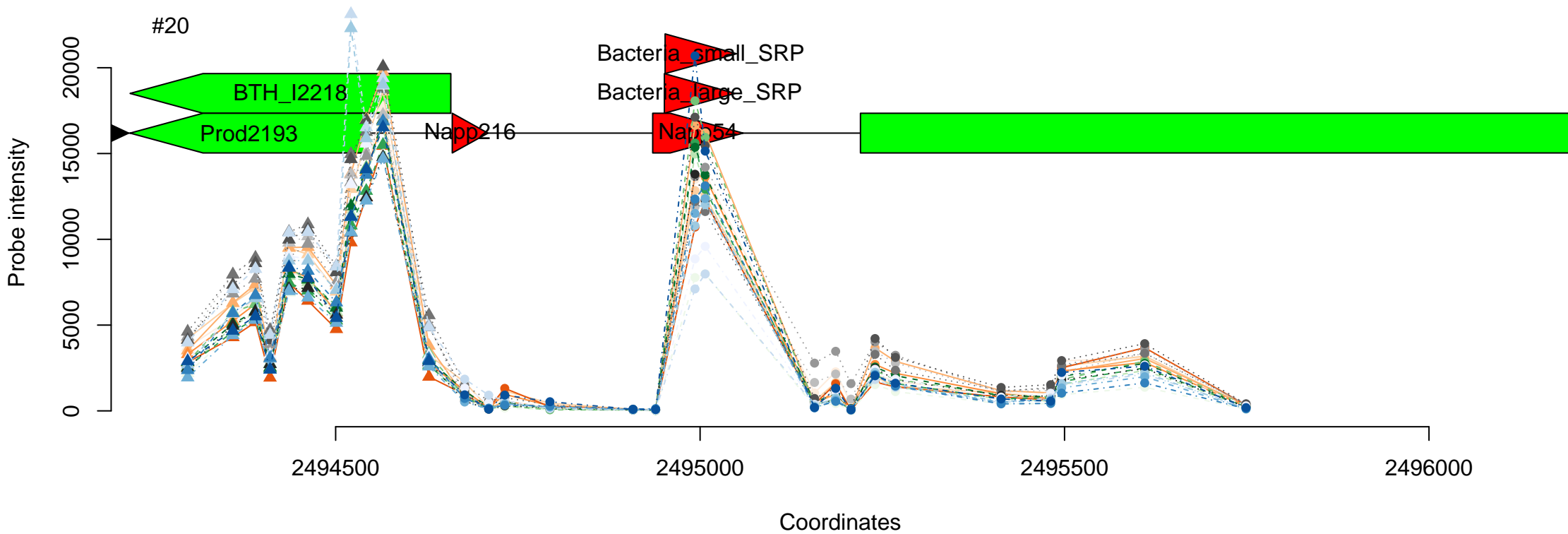

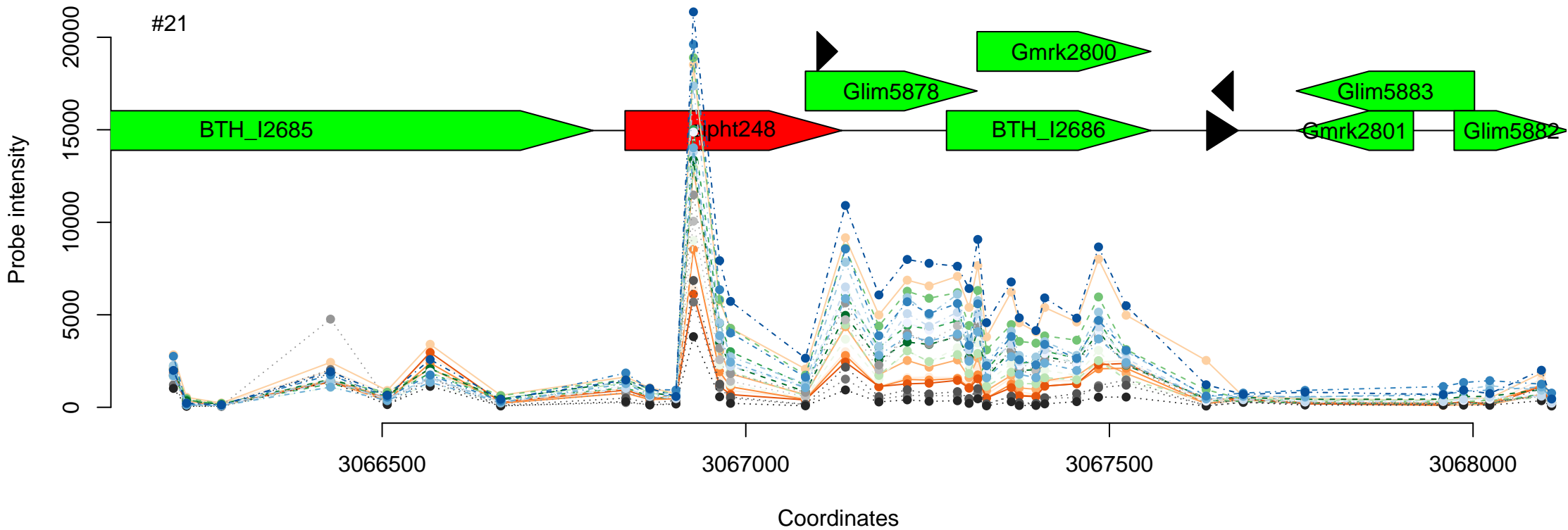

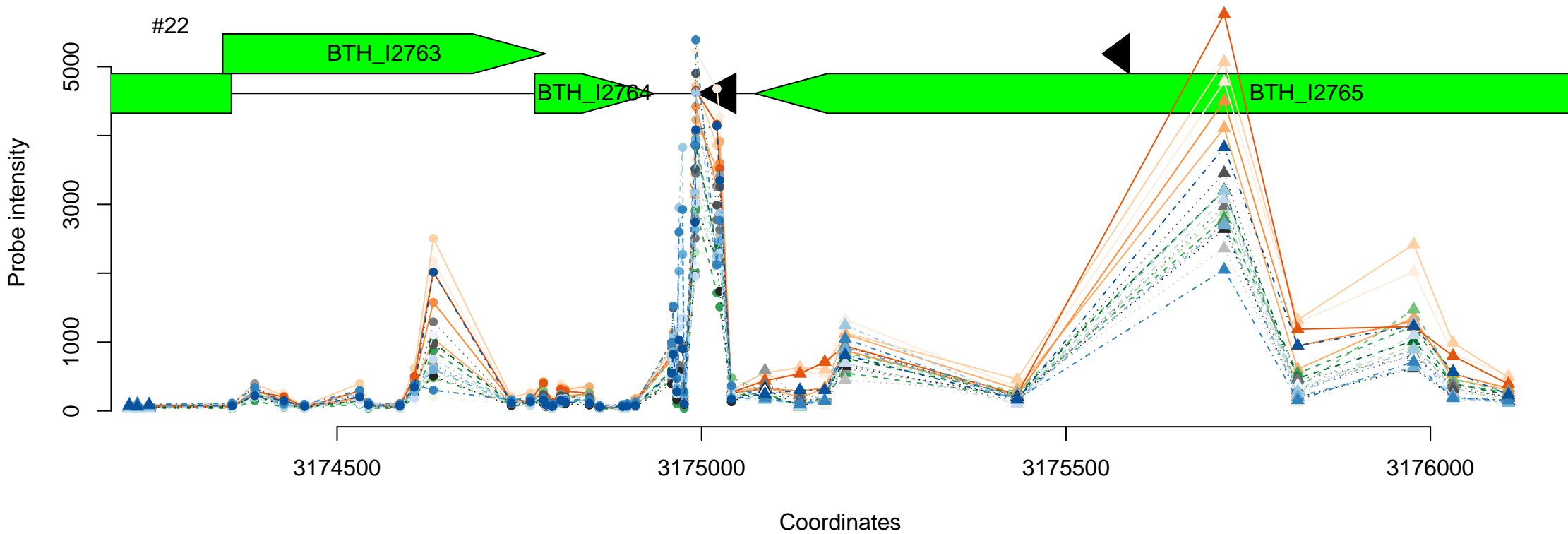

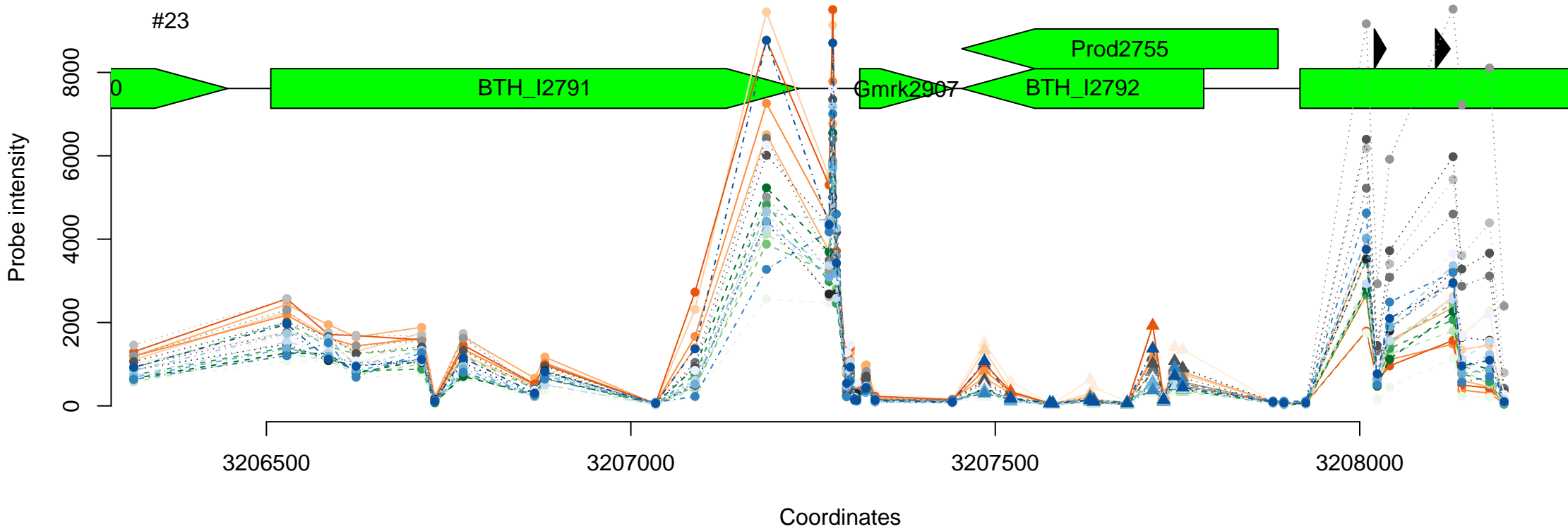

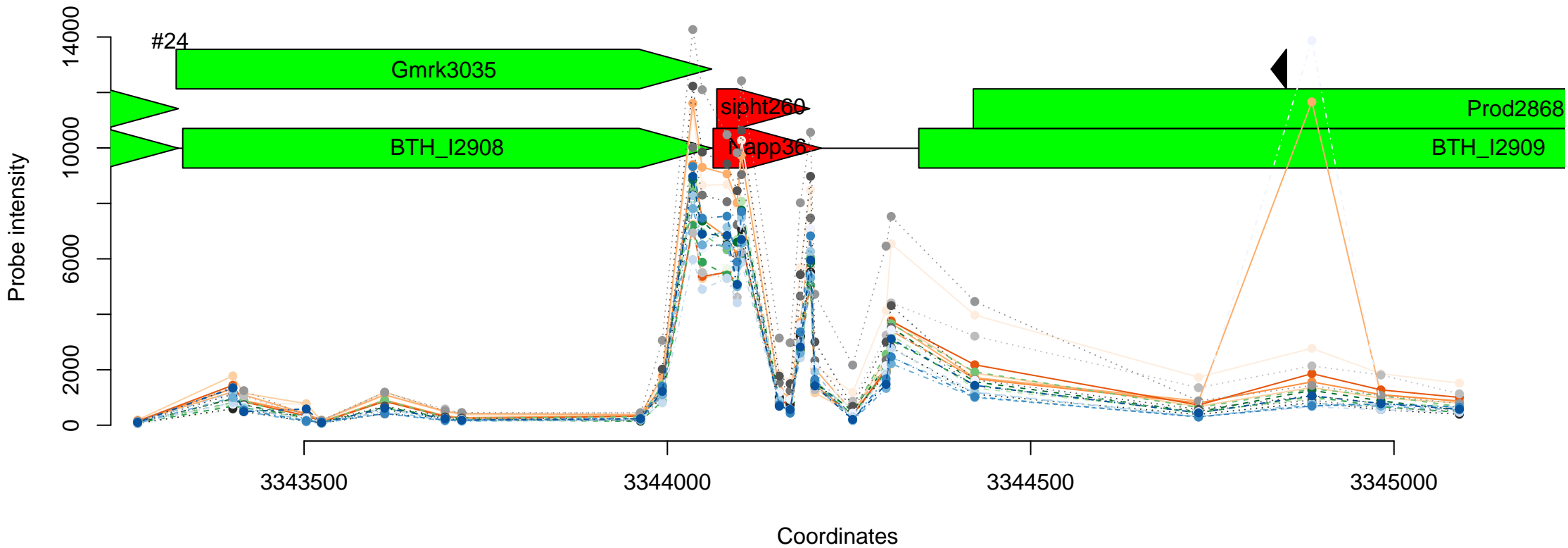

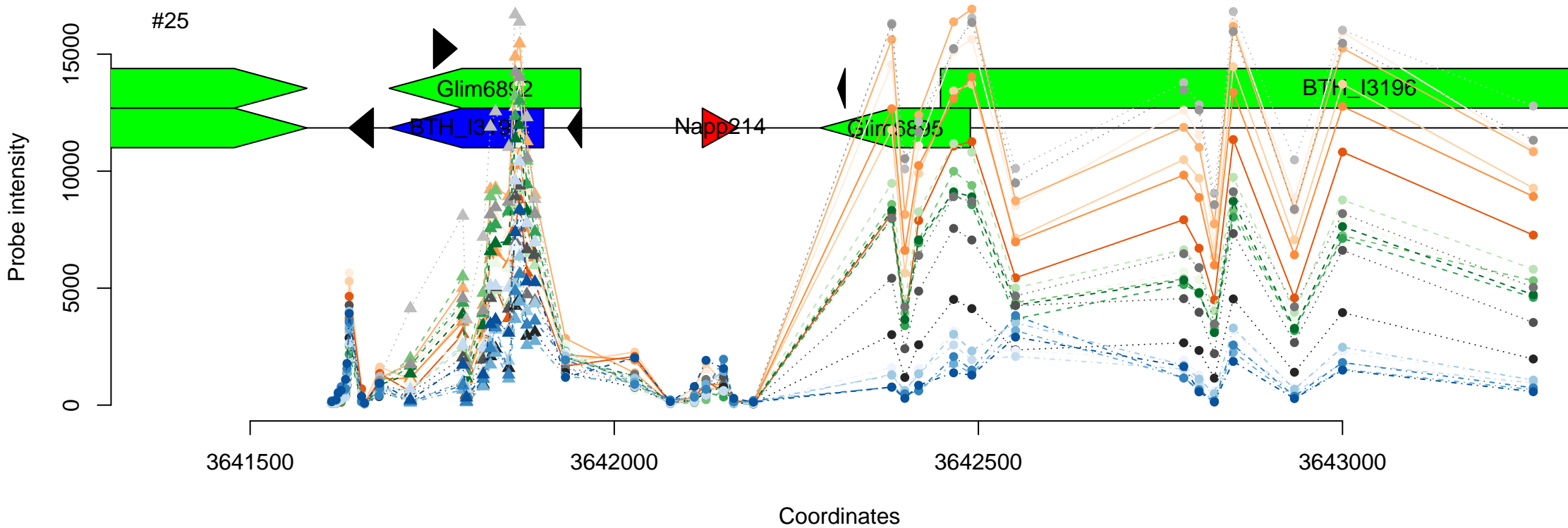

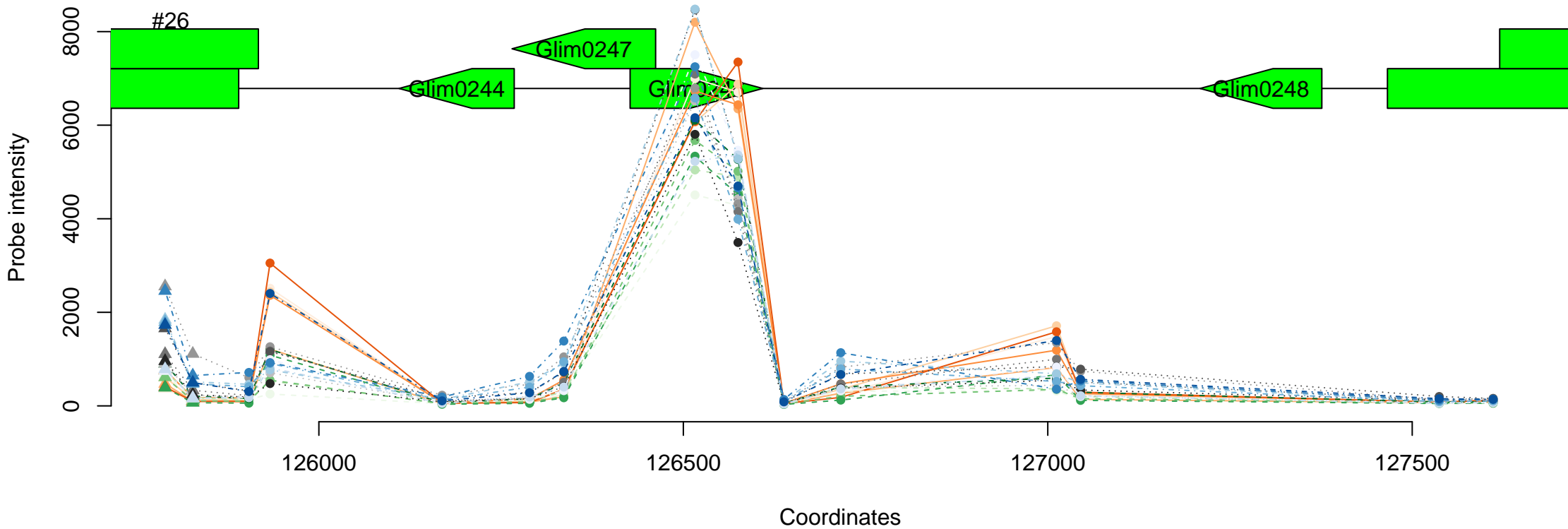

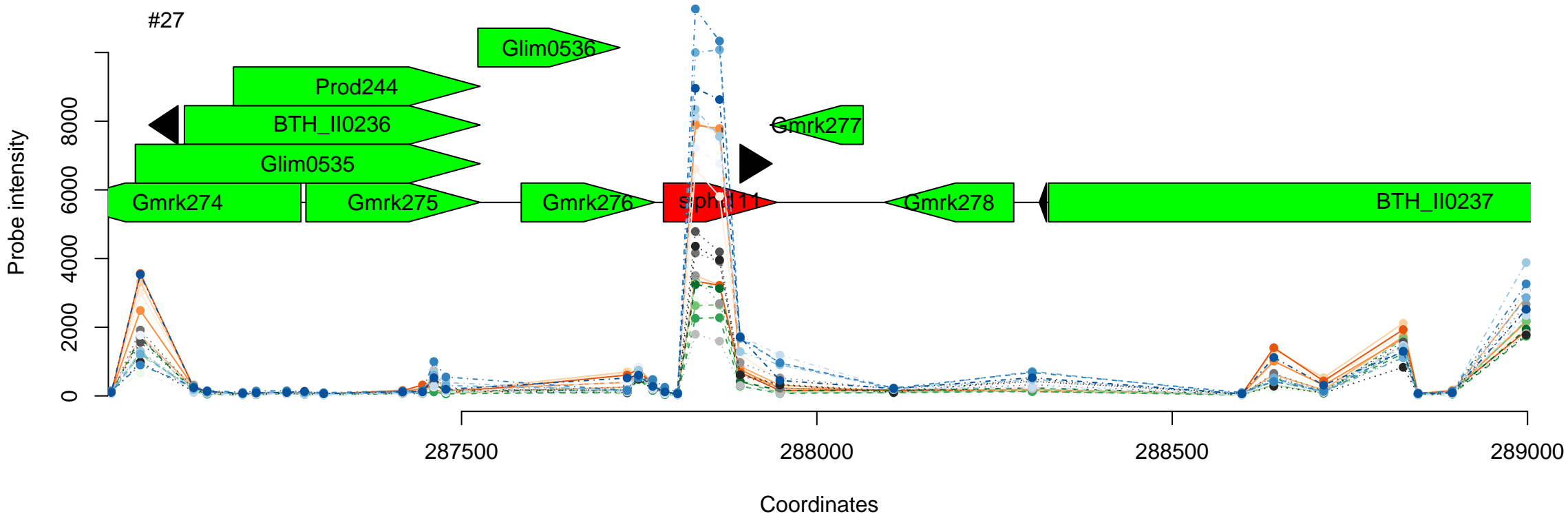

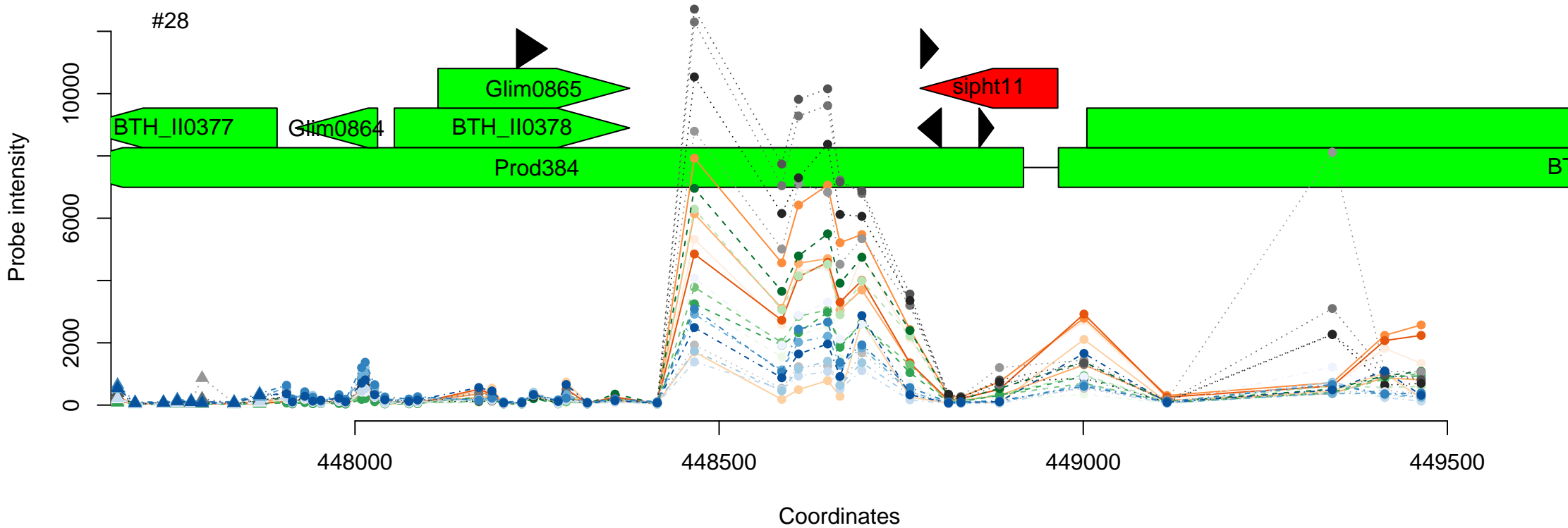

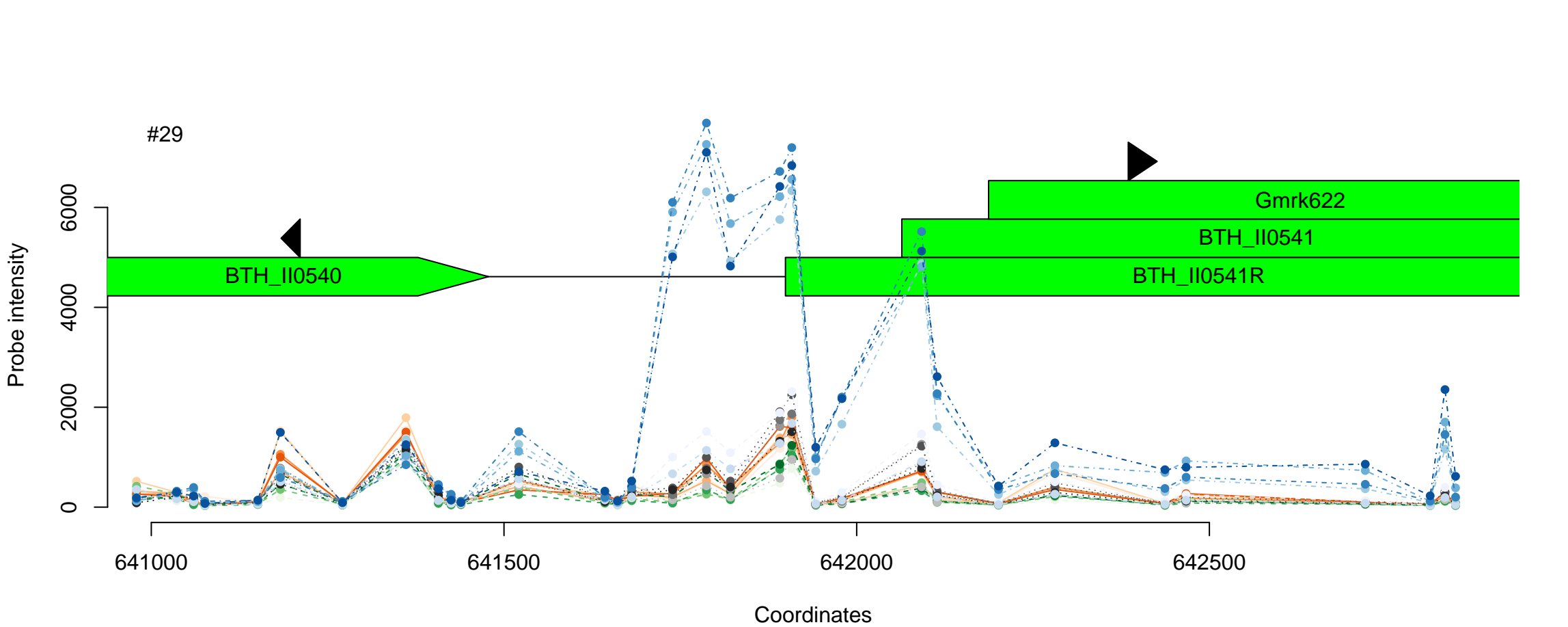

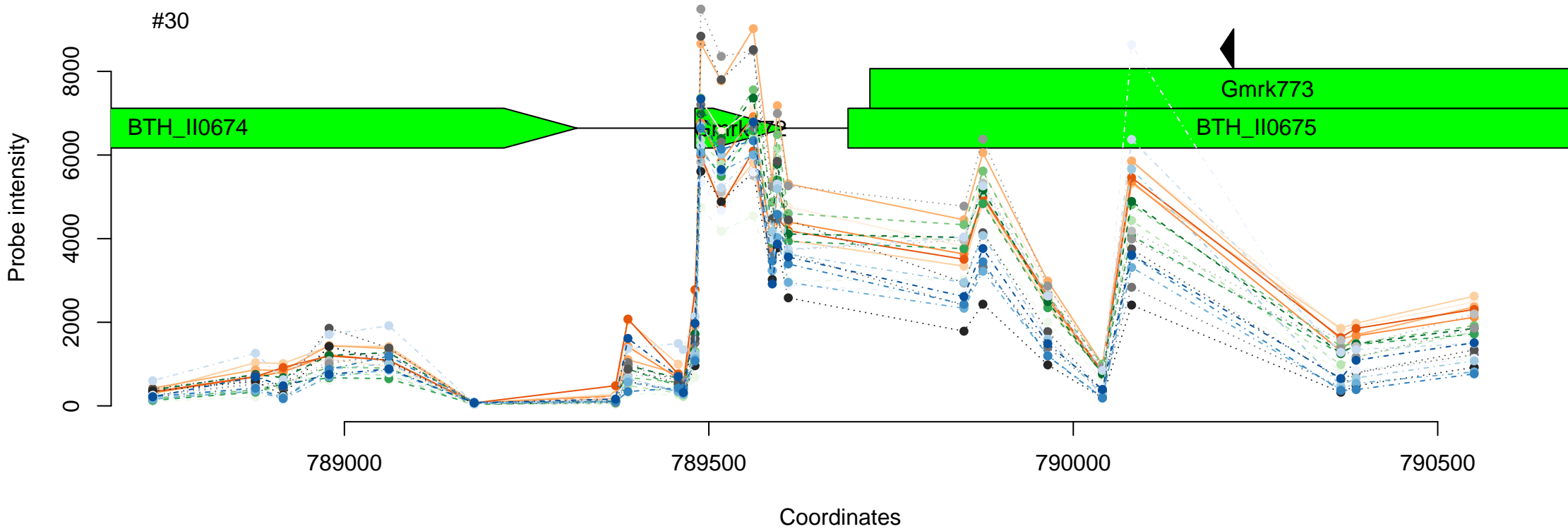

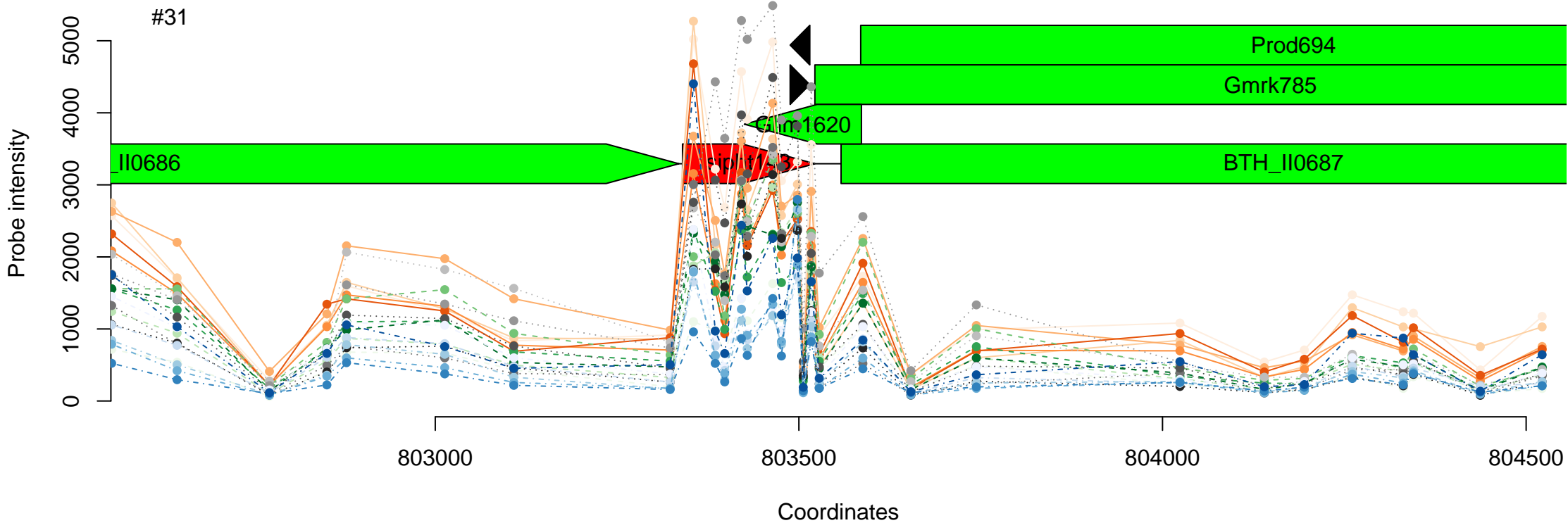

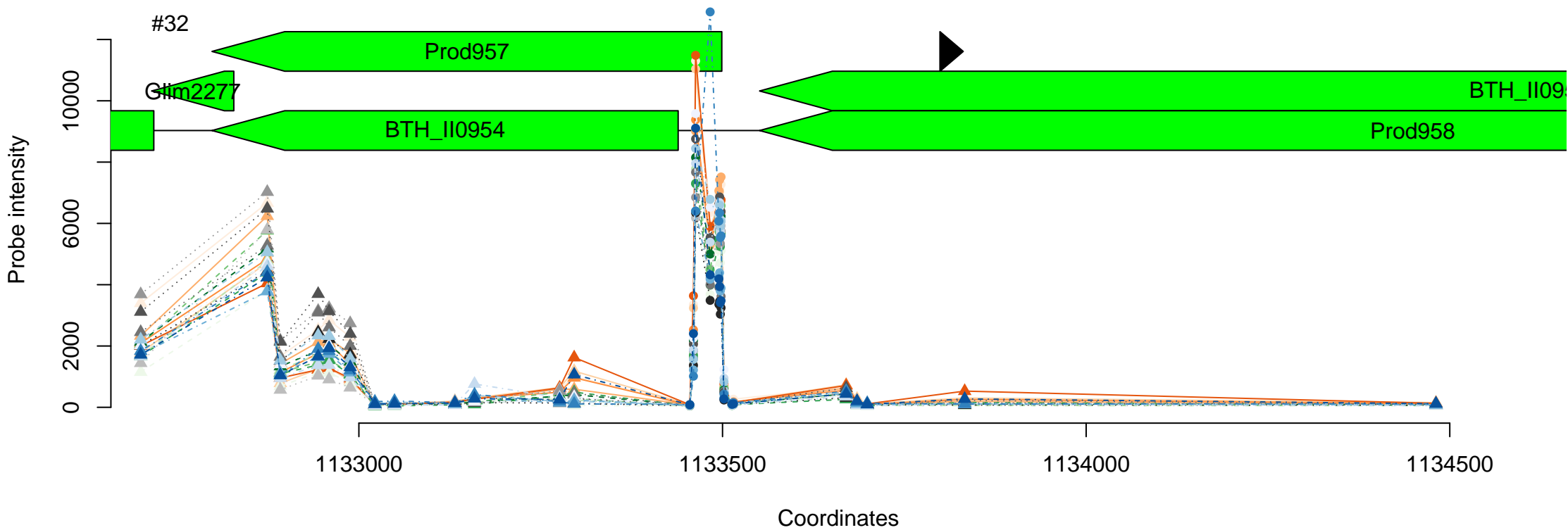

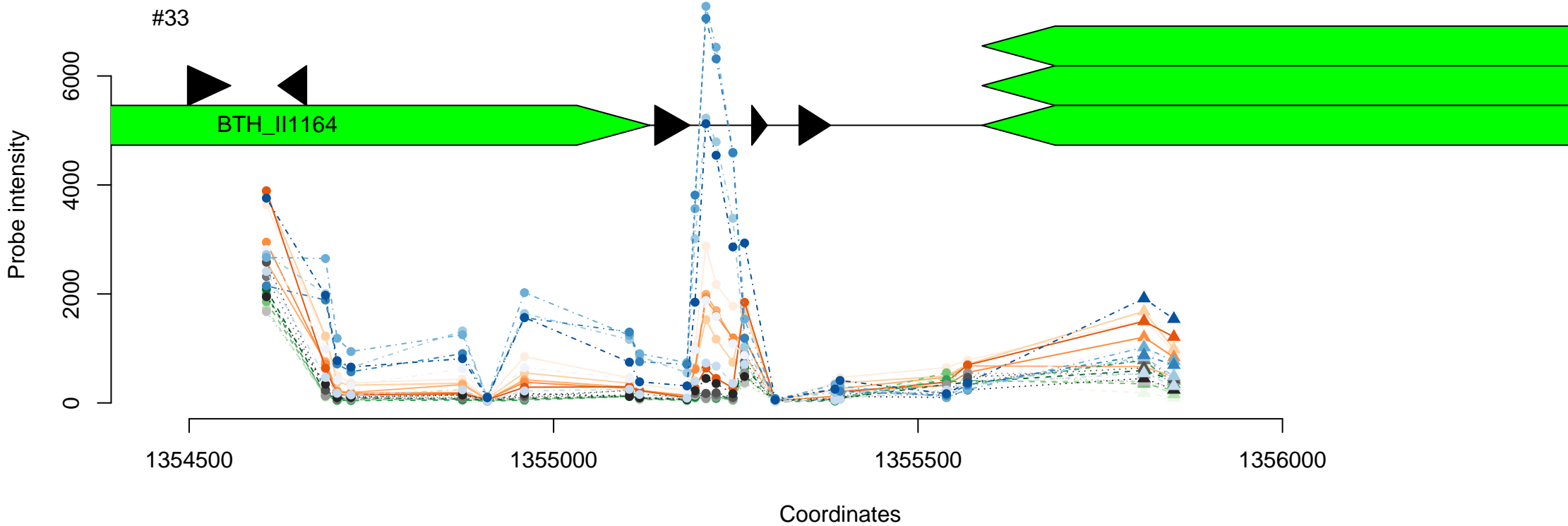

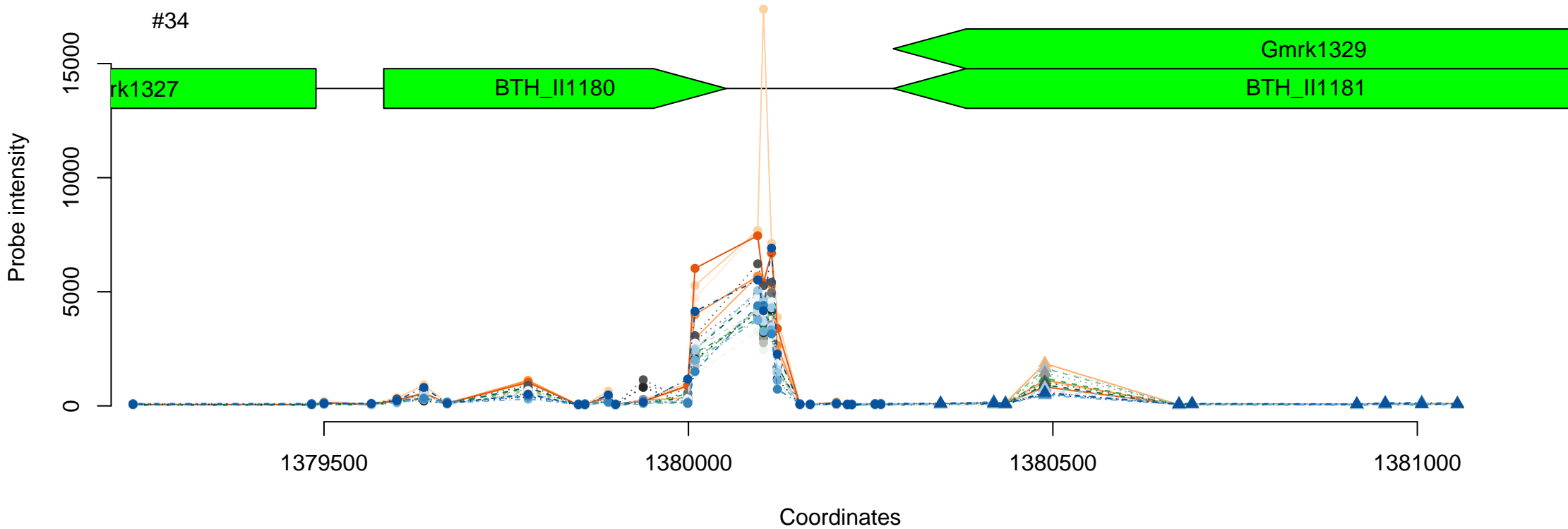

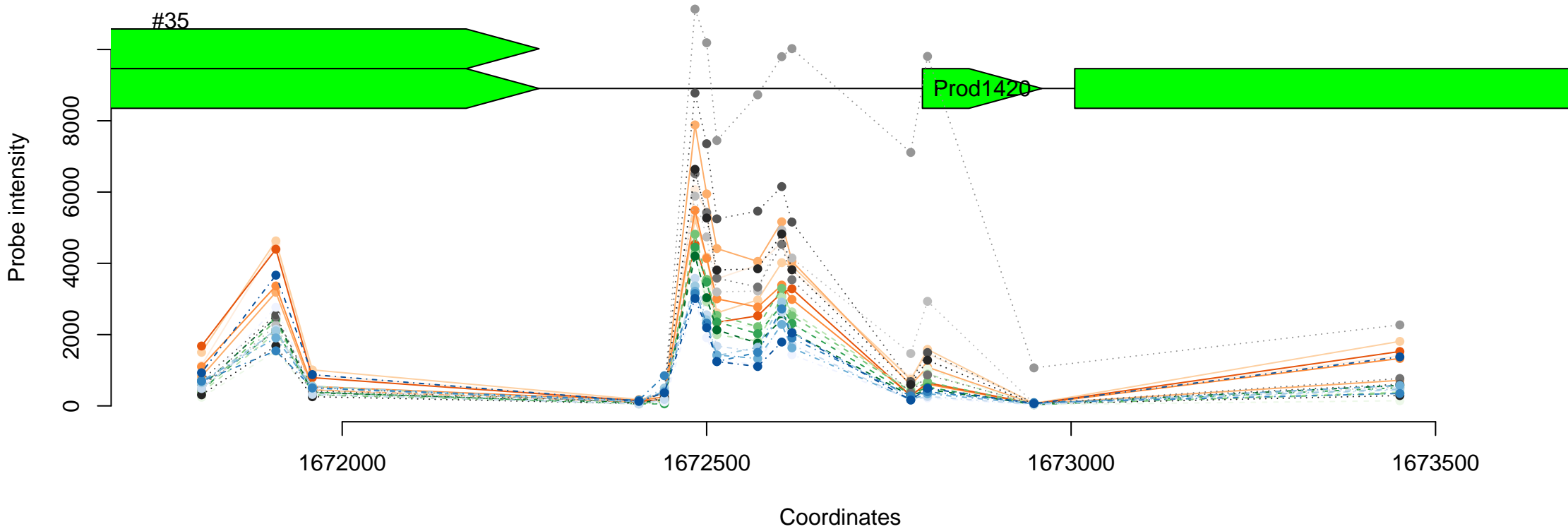

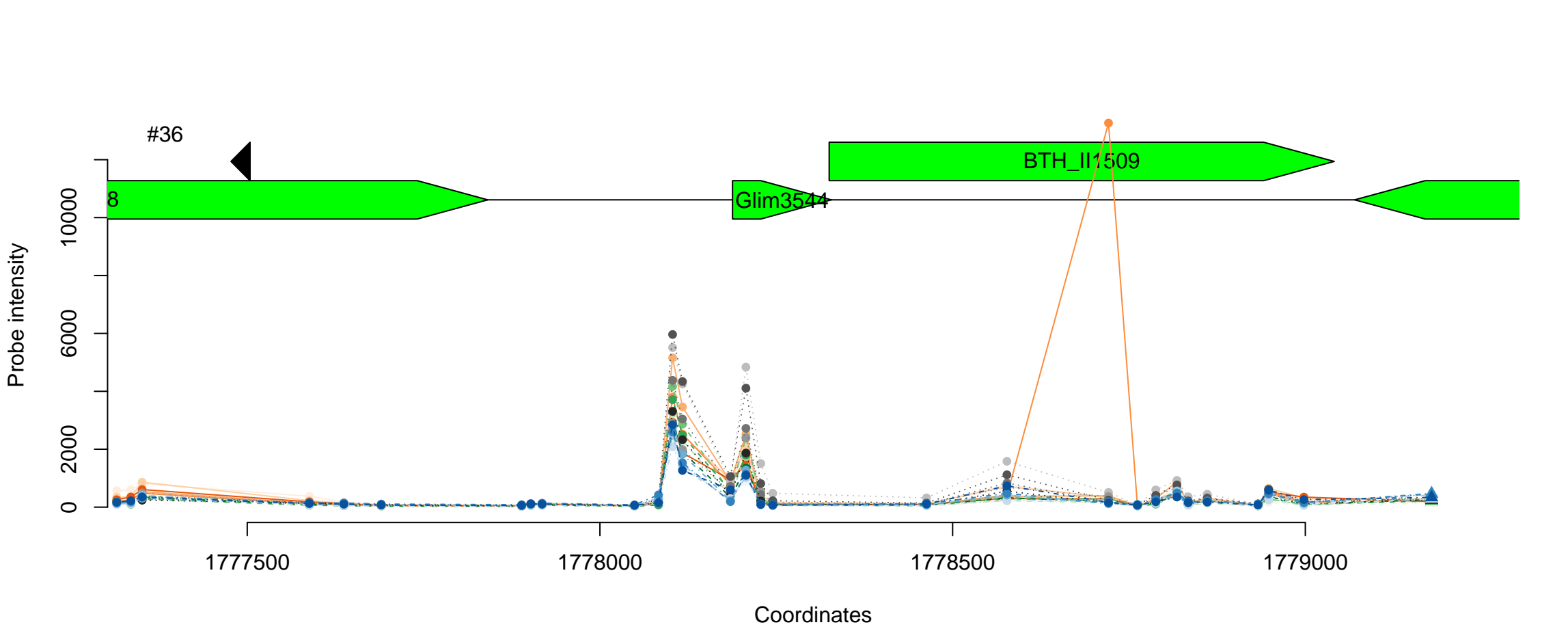

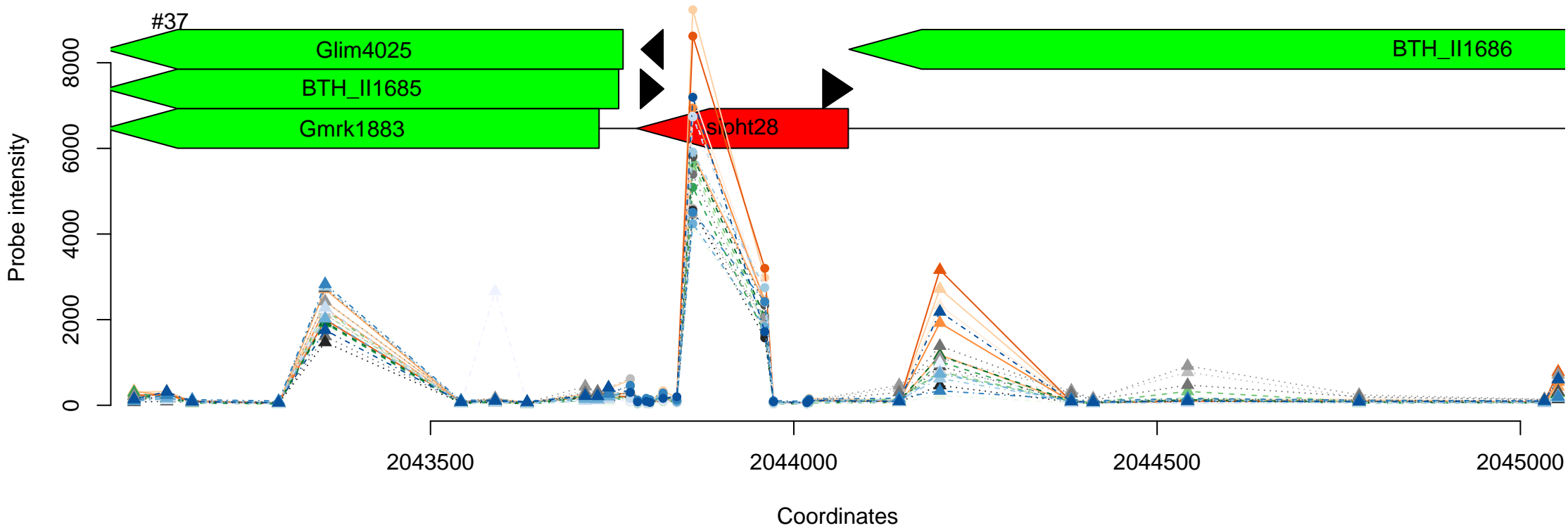

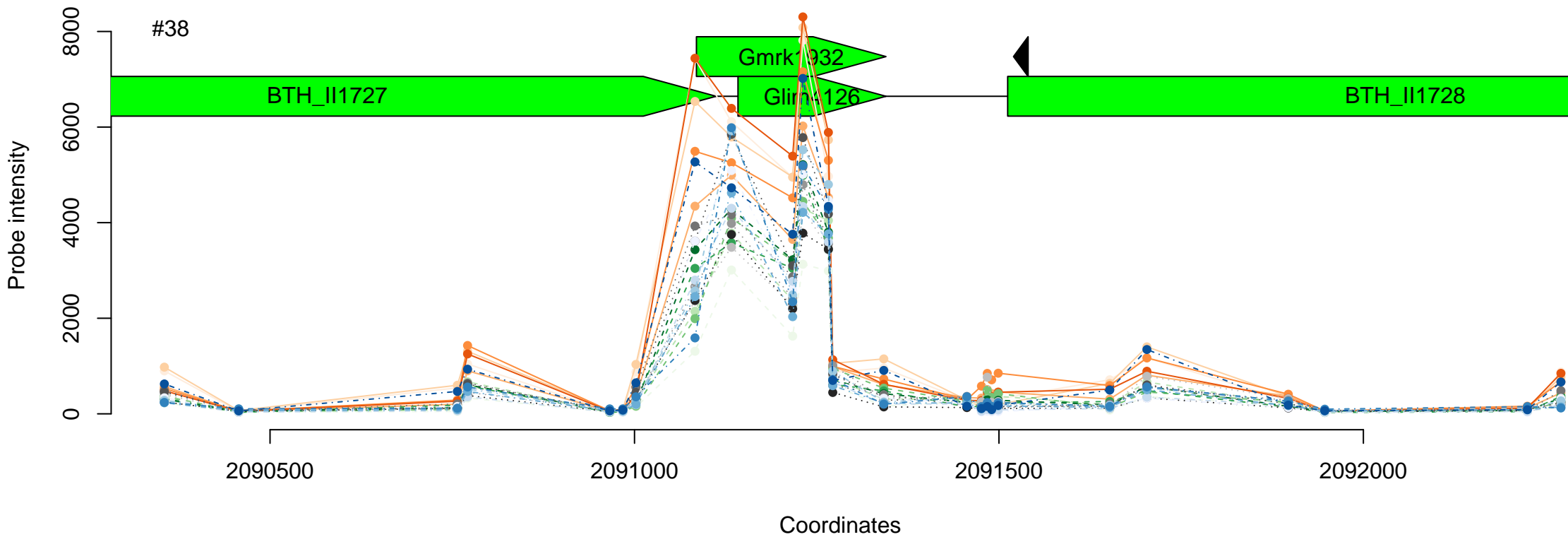

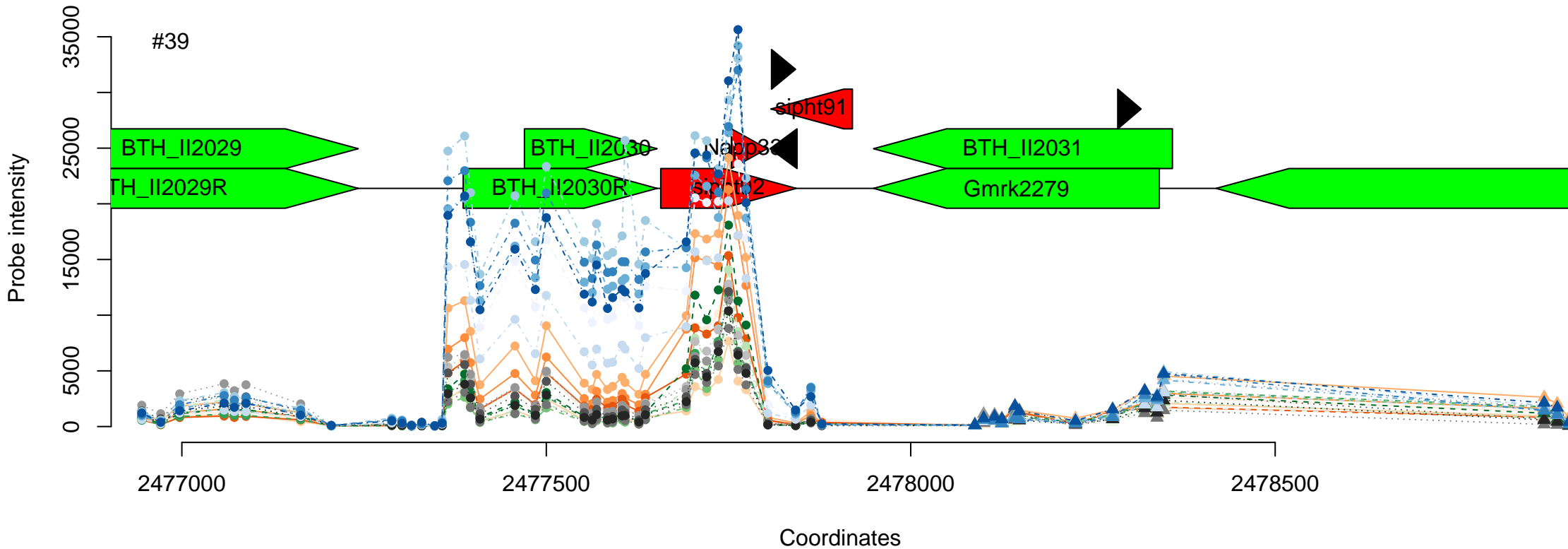

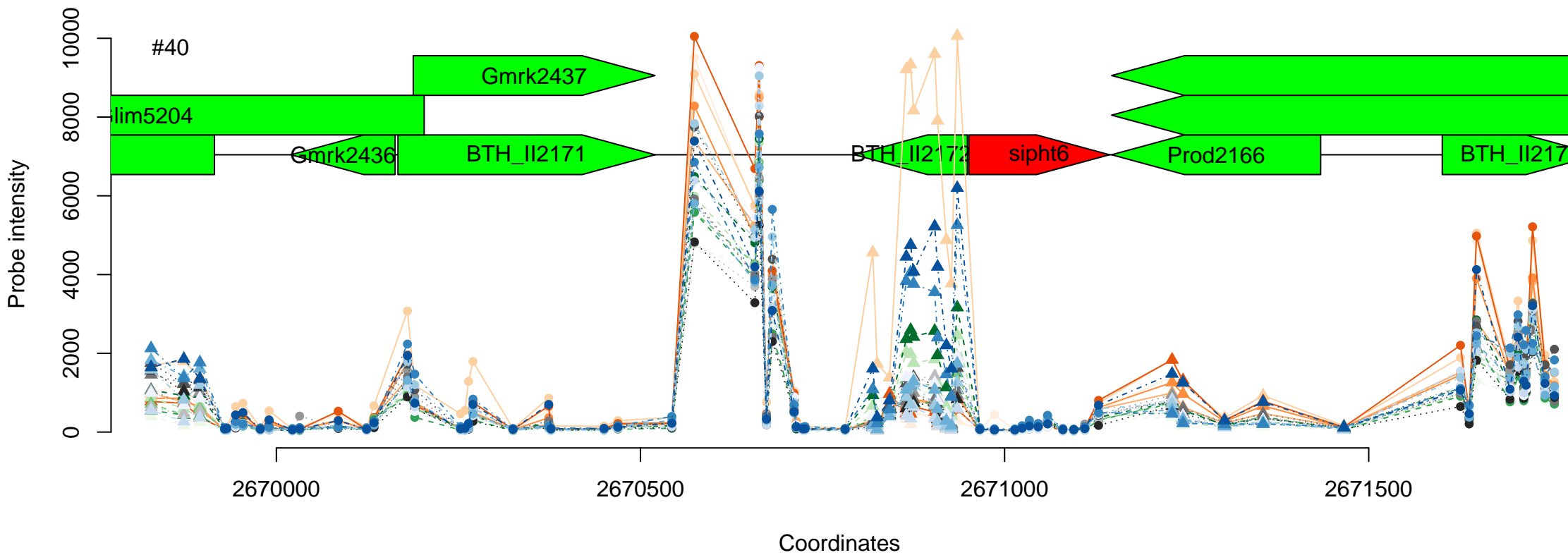

Supplement: Supplementary file 2 — Additional file 2: Expression plots of sRNAs. Expression plots are depicted of 40 sRNA candidates using probe data from 21 distinct conditions in the four time course arrays. Plot colors and labels are described in the Methods. (PDF 2 MB) [file 12864_2013_6069_MOESM2_ESM.pdf]
