# Supplementary material for: Differential expression of small RNAs from Burkholderia thailandensis in response to varying environmental and stress conditions
Source: BMC Genomics. 2014 May 19;15(1):385. doi: 10.1186/1471-2164-15-385 (PMC4035088; doi:10.1186/1471-2164-15-385)
Supplement: Supplementary file 3 — Additional file 3: sRNA sequences obtained from RACE. RACE sequences obtained from sequencing of BTH_s1, s13, s19, s27, s36, and s39. (DOCX 23 KB) [file 12864_2013_6069_MOESM3_ESM.docx]

**Additional file 3 - sRNA sequences obtained from RACE**

***BTH_s1*** (205nt, 97% similarity with *B.pseudomallei*)

AAATGCGCTACGGGGAACAATAAAAACAGCGCGGGGGTAGCGTTGCCGGCACGCTTTCGGCGTGCCGGCAGCGGTTGTCTGCATGCACGACGGGGCCAAATCACCCACAACAAGCCACCATGCAGGCACGTACGCCCATTCGATGGGCGTTCCGCCGTCCGGCCGGGGGAAGCCAGATGCGGGCGGCGGCGGACGCATTTTTTGT

***BTH_s13-cl1*** (284 nt, 98% similarity with *B.pseudomallei*)

TGTCCTAGCCAGCGCCCAGTCGACGCTGGTTTTTTGCGTGGGAGCCTCGCGCGTCCGCCTGCCGCAAGTGCAGGCCCTGTTTCGACGTGGCTCCGAACGGTACTTATACCGTTTCTCCGATACCGATGCGGTCCGACCAAGACGCTCGGTGTCTTGATTCCGCGGCAGCGCGCCATGTCTCGCTTCGAGCAACGAGCGAGCCGCACGAATCATGTCCATGGAGGATCTGAATGAACGCTTGGTTAACGTGGCGTCCCAATGAGCGGCATGCGCAGATGTTGCGC

***BTH_s13-cl2*** (218 nt, 99% similarity with *B.pseudomallei*))

TGTCCTAGCCAGCGCCCAGTCGACGCTGGTTTTTTGCGTGGGAGCCTCGCGCGTCCGCCTGCCGCAAGTGCAGGCCCTGTTTCGACGTGGCTCCGAACGGTACTTATACCGTTTCTCCGATACCGATGCGGTCCGACCAAGACGCTCGGTGTCTTGATTCCGCGGCAGCGCGCCATGTCTCGCTTCGAGCAACGAGCGAGCCGCACGAATCATGCTCC

***BTH_s13-cl3*** (209 nt, 99% similarity with *B.pseudomallei*)

GTTTTTTGCGTGGGAGCCTCGCGCGTCCGCCTGCCGCAAGTGCAGGCCCTGTTTCGACGTGGCTCCGAACGGTACTTATACCGTTTCTCCGATACCGATGCGGTCCGACCAAGACGCTCGGTGTCTTGATTCCGCGGCAGCGCGCCATGTCTCGCTTCGAGCAACGAGCGAGCCGCACGAATCATGTCCATGGAGGATCTGAATGAACG

***BTH_s13-cl4*** (388 nt, 96% similarity with *B.pseudomallei*)

TGTTGTCCTAGCCAGCGCCCAGTCGACGCTGGTTTTTTGCGTGGGAGCCTCGCGCGTCCGCCTGCCGCAAGTGCAGGCCCTGTTTCGACGTGGCTCCGAACGGTACTTATACCGTTTCTCCGATACCGATGCGGTCCGACCAAGACGCTCGGTGTCTTGATTCCGCGGCAGCGCGCCATGTCTCGCTTCGAGCAACGAGCGAGCCGCACGAATCATGTCCATGGAGGATCTGAATGAACGCTTGGTTAACGTGGCGTCCCAATGAGCGGCATGCGCAGATGTTGCGCGCAGCGCTGCGTCGCGGAACGCGTGTCAGTCACCATCTATTCAGCGTGGTCGGTTGCTGTGCGGTAGCGATCGCGCTGGCGCTGTGGCTCCTGCCCAATGT

***BTH_s13-cl5*** (333 nt, 95% similarity with *B.pseudomallei*)

GCTCCGAACGGTACTTATACCGTTTCTCCGATACCGATGCGGTCCGACCAAGACGCTCGGTGTCTTGATTCCGCGGCAGCGCGCCATGTCTCGCTTCGAGCAACGAGCGAGCCGCACGAATCATGTCCATGGAGGATCTGAATGAACGCTTGGTTAACGTGGCGTCCCAATGAGCGGCATGCGCAGATGTTGCGCGCAGCGCTGCGTCGCGGAACGCGTGTCAGTCACCATCTATTCAGCGTGGTCGGTTGCTGTGCGGTAGCGATCGCGCTGGCGCTGTGGCTCCTGCCCAATGTGCGCGGCACGCTTGCGGCGAAAGTGATGCCGTTCGTG

***BTH_s13-1,2*** [400nt, 1728381- 1728780]

TTGTCCTAGCCAGCGCCCAGTCGACGCTGGTTTTTTGCGTGGGAGCCTCGCGCGTCCGCCTGCCGCAAGTGCAGGCCCTGTTTCGACGTGGCTCCGAACGGTACTTATACCGTTTCTCCGATACCGATGCGGTCCGACCAAGACGCTCGGTGTCTTGATTCCGCGGCAGCGCGCCATGTCTCGCTTCGGGCAACGAGCGAGCCGCACGAATCATGTCCATGGAGGATCTGAATGAACGCTTGGTTAACGTGGCGTCCCAATGAGCGGCATGCGCAGATGTTGCGCGCAGCGCTGCGTCGCGGAACGCGTGTCAGTCACCATCTATTCAGCGTGGTCGGTTGCTGTGCGGTAGCGATCGCGCTGGCGCTGTGGCTCCTGCCCAATGTGCGCGGCACGCTTG

***BTH_s13-3*** [388nt, 1728471- 1728858]

GCTCCGAACGGTACTTATACCGTTTCTCCGATACCGATGCGGTCCGACCAAGACGCTCGGTGTCTTGATTCCGCGGCAGCGCGCCATGTCTCGCTTCGAGCAACGAGCGAGCCGCACGAATCATGTCCATGGAGGATCTGAATGAACGCTTGGTTAACGTGGCGTCCCAATGAGCGGCATGCGCAGATGTTGCGCGCAGCGCTGCGTCGCGGAACGCGTGTCAGTCACCATCTATTCAGCGTGGTCGGTTGCTGTGCGGTAGCGATCGCGCTGGCGCTGTGGCTCCTGCCCAATGTGCGCGGCACGCTTGCGGGGAAAGTGATGCCGTTCGTGTCGGCCGCGGTTCAGGCCGGCACGGCGCGCCTGCTGAGCGGCCATCCGCTGCCGA

***BTH_s13-4*** [307nt, 1728471- 1728779]

GCTCCGAACGGTACTTATACCGTTTCTCCGATACCGATGCGGTCCGACCAAGACGCTCGGTGTCTTGATTCCGCGGCAGCGCGCCATGTCTCGCTTCGAGCAACGAGCGAGCCGCACGAATCATGTCCATGGAGGATCTGAATGAACGCTTGGTTAACGTGGCGTCCCAATGAGCGGCATGCGCAGATGTTGCGCGCAGCGCTGCGTCGCGGAACGCGTGTCAGTCACCATCTATTCAGCGTGGTCGGTTGCTGTGCGGTAGCGATCGCGCTGGCGCTGTGGCTCCTGCCCAATGTGCGCGGCACGCTT

***BTH_s13-5*** [280nt, **1728279**- **1728560**]

CATTGGTTGCAGGCCCCTGTAGTTGAACCATTAATCCCCGGCCCTATAGACGCAACGCGTCACCGTCCGCCGATACCTGCACGGCCTTCTGACGGTTGTTTGTTGTCCTAGCCAGCGCCCAGTCGACGCTGGTTTTTTGCGTGGGAGCCTCGCGCGTCCGCCTGCCGCAGGTGCAGGCCCTGTTTCGACGTGGCTCCGAACGGTACTTATACCGTTTCTCCGATACCGATGCGGTCCGACCAAGACGCTCGGTGTCTTGATTCCGCGGCAGCGCGCCATGTC

***BTH_s13-6*** [300nt, 1728381- 1728682]

***BTH_s13-7*** [286nt, **1728278**- 1728543]

***BTH_s13-8*** [300nt, 1728417- 1728722]

***BTH_s13-9*** [320nt, 1728383- 1728704]

***BTH_s19-c1*** (223 nt, 96% similarity with *B.pseudomallei*)

CACTTAACATTTACATGGTTTGCACGCCCGATTCTCGCAGGGTTTGTTCGTGTGCCAAAGGTAGCCGGTTGCCGCGTGACGAAGGGAACAAAACGACGAAAGAGGCGAGCCAGTGATGCGGCCGACGAAGATGGCGTTCGTGCTGGCGCTCGCTTGCGTCGGCTTCCTCGCTTCGGACTTCGCTTGCGCGCAGCGCCCGGAACATGGCGCCGCGCCGTCGCAC

***BTH_s19-c2*** (133 nt, 99% similarity with *B.pseudomallei*)

TTTGCACGCCCGATTCTCGCAGGGTTTGTTCGTGTGCCAAAGGTAGCCGGTTGCCGCGTGACGAAGGGAACAAACGACGAAAGAGGCGAGG CCAGTGATGCGGCCGACGAAGATGGCGTTCGTGCTGGCGCTC

***BTH_s27-c1-4*** (101 nt, 94% similarity with B.pseudomallei)

GGAAAGGCTATTCGACGTCCGGCCTTTTCCGATCTCGAGAAAATGCAGCAATCGGCCCATGTTGTATGGTTGATCGACCTGCCGCACTGCGTCATGACTGT

***BTH_s36-cl3*** (81 nt, 97% similarity with B.pseudomallei)

TTCAGGAACCGTCGTGGACAGTAGCAGCACAACGTCCGCACGCCGCTTCGCCTGAACGCCTGCCGGCCGATTGCCGCCACA

***BTH_s36-cl4*** (170 nt, 96% similarity with B.pseudomallei)

ATCCATTCTTTCAGGAACCGTCGTGGACAGTAGCAGCACAACGTCCGCACGCCGCTTCGCCTGAACGCCTGCCGGCCGATTGCCGCCGCACGCGTTCGTCGGAACGCGTGCGATTTCCCGTTTCACACAATCGCATCGATTCGCGCGCTAAAGTGTCGCGCGAGTATGTA

***BTH_s39-c1*** (127 nt, 99% similarity with *B.pseudomallei*)

GTATTGTGGGGACCACCTCTACGAGAGGTGTTAGGCATTAGCCAGCCACAACGGCTTGCACGCGAGTCTAGACCCCTTCGTGCAAAACATATTCAAGCAGCCGTTGCAGCAAGCCAAGCCGCTTTTT

***BTH_s39-c2*** the same as c1, two sequences ligated one after another

GTATTGTGGGGACCACCTCTACGAGAGGTGTTAGGCATTAGCCAGCCACAACGGCTTGCA CGCGAGTCTAGACCCCTTCGTGCAAACATATTCAAGCAGCCGTTGCAGCAAGCCAAGCCG

CTTTTTT

***BTH_s39-c3*** (130 nt, 99% similarity with *B.pseudomallei*)

GTATTGTGGGGACCACCTCTACGAGAGGTGTTAGGCATTAGCCAGCCACAACGGCTTGCA CGCGAGTCTAGACCCCTTCGTGCAAAACATATTCAAGCAGCCGTTGCAGCAAGCCAAGCCGCTTTTTTTG

***BTH_s39-c4*** is the same as BTH_sR39-c1
