# Supplementary material for: Differential expression of small RNAs from Burkholderia thailandensis in response to varying environmental and stress conditions
Source: BMC Genomics. 2014 May 19;15(1):385. doi: 10.1186/1471-2164-15-385 (PMC4035088; doi:10.1186/1471-2164-15-385)
Supplement: Supplementary file 5 — Additional file 5: B. thailandensis annotations from Pathema. Pathema gene annotations downloaded on Sep 20, 2012 from the Pathosystems Resource Intergration Center (http://www.patricbrc.org). (DOCX 195 KB) [file 12864_2013_6069_MOESM5_ESM.docx]

**Additional file 5. R code for B. thailandensis arrays**

# Install CDF library once
setwd("~/path/to/array/files")

library(makecdfenv)

make.cdf.package("thaiLANLa520655F.CDF", species = "Burkholderia thailandensis")

install.packages("thailanla520655fcdf", repos=NULL)

# LOAD time course for temperature
# IF *.CEL files in directory called "temp"
# create covdesc file by reading files and assigning timepoints
x <- data.frame(file= list.files("temp", pattern="CEL$"), time=paste("t", rep(0:4, each=5), sep=""))
write.table(x, file="temp/covdesc", quote=FALSE, sep="\t", row.names=FALSE)

# Load CEL files
library(simpleaffy)
x <- read.affy( path="temp")
sampleNames(x) <- gsub(".CEL", "",sampleNames(x))

# QUALITY assessment
# plots of normalized unscaled standard error (NUSE) and relative log expression (RLE).
library(affyPLM)
xPLM <- fitPLM(x)
boxplot(xPLM, main="NUSE", ylim=c(.95, 1.25), outline = FALSE, col="lightblue", names=NA, whisklty=0, staplelty=0)
Mbox(xPLM, main="RLE", ylim = c(-0.45, 0.45), outline = FALSE, col="mistyrose", las=3, whisklty=0, staplelty=0)
# DROP SAMPLE 25
x <- x[, -25]

# BACKGROUND correction
x1 <- bg.correct.rma(x)
# combine NORMALIZED arrays
x2 <- combine(normalize(x1[, 1:5]), normalize(x1[,6:10]),

normalize(x1[,11:15]), normalize(x1[,16:20]), normalize(x1[,21:24]))

# Summarize perfect match intensity values

pm1 <- pm(x2)

pm2<-data.frame(id= as.numeric(rownames(pm1)), name=probeNames(x2),

t0=rowMeans(pm1[, 1:5]), t1=rowMeans(pm1[,6:10]),

t2=rowMeans(pm1[,11:15]), t3=rowMeans(pm1[,16:20]),

t4=rowMeans(pm1[,21:24]), stringsAsFactors=FALSE)

Fig 1. NUSE and RLE plot from temperature increase. Sample 25 was excluded.

Fig 2. NUSE and RLE plot from pH drop. Samples 16 and 19 were excluded.

Fig 3. NUSE and RLE plot from salt increase. Sample 1 was excluded.

Fig 4. NUSE and RLE plot from phosphate starvation. Sample 28 was excluded.
