# Supplementary material for: Differential expression of small RNAs from Burkholderia thailandensis in response to varying environmental and stress conditions
Source: BMC Genomics. 2014 May 19;15(1):385. doi: 10.1186/1471-2164-15-385 (PMC4035088; doi:10.1186/1471-2164-15-385)
Supplement: Supplementary file 7 — Additional file 7: Primer sequences. The primer sequences for sRNAs and the internal control gene 5S rRNA for Northern blots and qPCR. (PPTX 80 KB) [file 12864_2013_6069_MOESM7_ESM.pptx]

## Slide 1
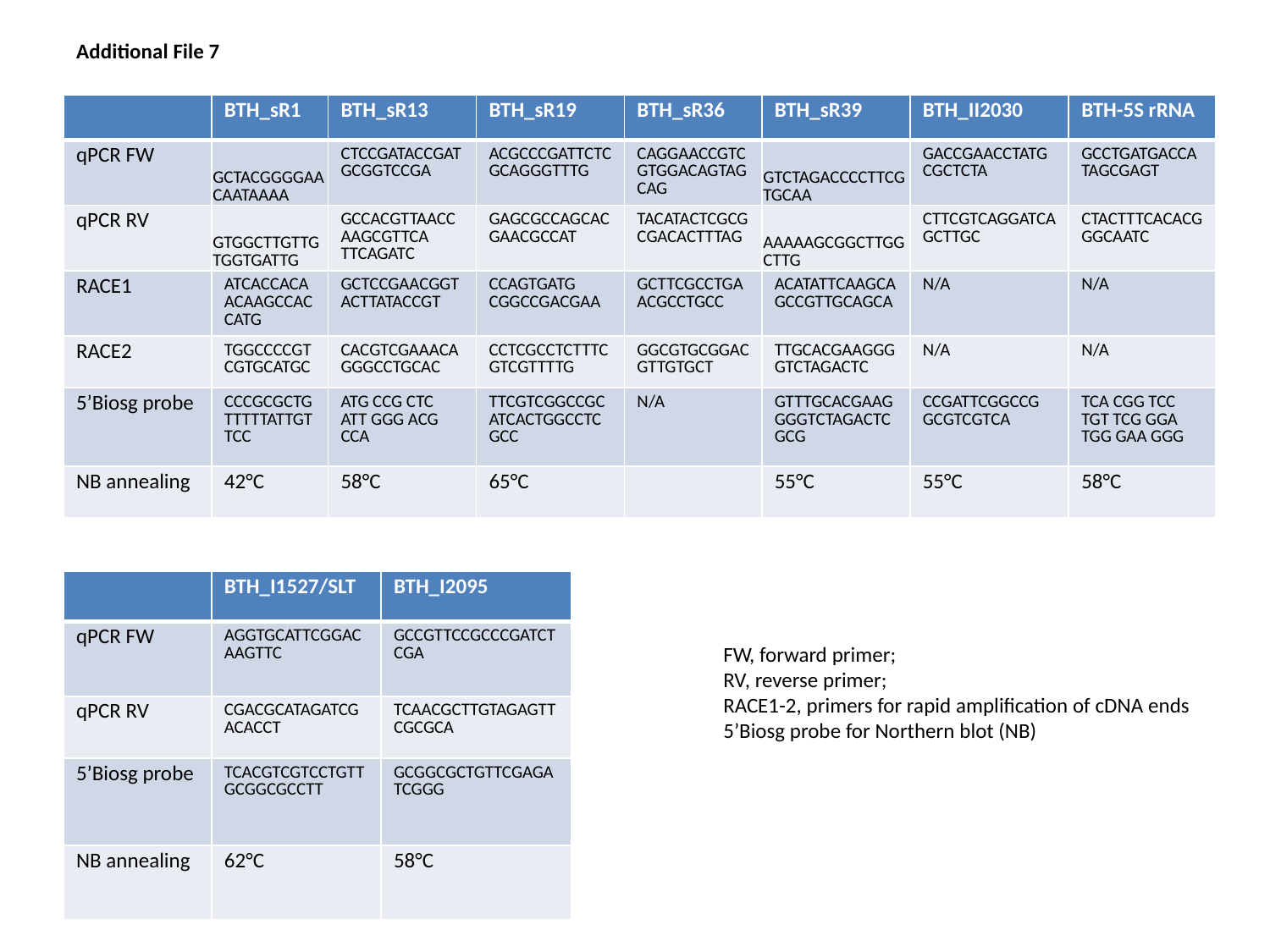

Additional File 7
| | BTH\_sR1 | BTH\_sR13 | BTH\_sR19 | BTH\_sR36 | BTH\_sR39 | BTH\_II2030 | BTH-5S rRNA |
| --- | --- | --- | --- | --- | --- | --- | --- |
| qPCR FW | GCTACGGGGAACAATAAAA | CTCCGATACCGATGCGGTCCGA | ACGCCCGATTCTCGCAGGGTTTG | CAGGAACCGTCGTGGACAGTAGCAG | GTCTAGACCCCTTCGTGCAA | GACCGAACCTATGCGCTCTA | GCCTGATGACCATAGCGAGT |
| qPCR RV | GTGGCTTGTTGTGGTGATTG | GCCACGTTAACCAAGCGTTCA TTCAGATC | GAGCGCCAGCACGAACGCCAT | TACATACTCGCGCGACACTTTAG | AAAAAGCGGCTTGGCTTG | CTTCGTCAGGATCAGCTTGC | CTACTTTCACACGGGCAATC |
| RACE1 | ATCACCACAACAAGCCACCATG | GCTCCGAACGGTACTTATACCGT | CCAGTGATG CGGCCGACGAA | GCTTCGCCTGAACGCCTGCC | ACATATTCAAGCAGCCGTTGCAGCA | N/A | N/A |
| RACE2 | TGGCCCCGTCGTGCATGC | CACGTCGAAACAGGGCCTGCAC | CCTCGCCTCTTTCGTCGTTTTG | GGCGTGCGGACGTTGTGCT | TTGCACGAAGGGGTCTAGACTC | N/A | N/A |
| 5’Biosg probe | CCCGCGCTGTTTTTATTGTTCC | ATG CCG CTC ATT GGG ACG CCA | TTCGTCGGCCGCATCACTGGCCTCGCC | N/A | GTTTGCACGAAGGGGTCTAGACTCGCG | CCGATTCGGCCG GCGTCGTCA | TCA CGG TCC TGT TCG GGA TGG GAA GGG |
| NB annealing | 42°C | 58°C | 65°C | | 55°C | 55°C | 58°C |
| | BTH\_I1527/SLT | BTH\_I2095 |
| --- | --- | --- |
| qPCR FW | AGGTGCATTCGGACAAGTTC | GCCGTTCCGCCCGATCTCGA |
| qPCR RV | CGACGCATAGATCGACACCT | TCAACGCTTGTAGAGTTCGCGCA |
| 5’Biosg probe | TCACGTCGTCCTGTTGCGGCGCCTT | GCGGCGCTGTTCGAGATCGGG |
| NB annealing | 62°C | 58°C |
FW, forward primer;
RV, reverse primer;
RACE1-2, primers for rapid amplification of cDNA ends
5’Biosg probe for Northern blot (NB)
